# Efficient, Functional Group‐Tolerant, and Catalyst‐Free Nitrile Formation from Aldehydes

**DOI:** 10.1002/chem.202502629

**Published:** 2025-11-10

**Authors:** Simay Aydonat, Davide Campagna, Robert Göstl

**Affiliations:** ^1^ Department of Chemistry and Biology University of Wuppertal Gaußstr. 20 42119 Wuppertal Germany; ^2^ DWI – Leibniz Institute for Interactive Materials Forckenbeckstr. 50 52056 Aachen Germany; ^3^ Institute of Technical and Macromolecular Chemistry RWTH Aachen University Worringerweg 2 52074 Aachen Germany

**Keywords:** aldehydes, carbamates, cyanides, nucleophilic addition‐elimination, rearrangement

## Abstract

The development of efficient methods for nitrile syntheses is a challenge due to the current reliance on toxic cyanide sources, metal catalysts and their associated waste products, and harsh conditions that limit functional group compatibility. Here, we show a metal‐ and complex reagent‐free strategy for the conversion of aldehydes to nitriles through carbamoylaldoxime intermediates. Therefore, we synthesize aldoximes from several aldehydes using hydroxylamine, which in turn are reacted with dimethylcarbamoyl chloride (DMCC) to afford *N,N‐*dimethylcarbamoyloximes. Subsequent heating cleanly produces the desired nitriles as well as volatile CO_2_ and HNMe_2_ through a pericyclic *syn* elimination. This approach relies on widely available commercial chemicals, proceeds with broad functional group tolerance, and minimizes the need for extensive purification of the nitrile product.

## Graphical abstract

Metal‐ and cyanide‐free aldehyde‐to‐nitrile synthesis by carbamoylaldoxime intermediates is described. Aldoximes form *N*,*N*‐dimethylcarbamoyloximes, which undergo clean thermal *syn* elimination to give nitriles, CO_2_, and dimethylamine with broad functional‐group tolerance and minimal purification.

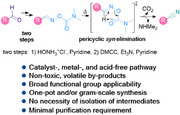

Nitriles are widely present as important structural motifs in pharmaceuticals,^[^ ^1^ ^]^ agrochemicals,^[^ ^2^ ^]^ materials,^[^ ^3^ ^]^ polymers,^[^ ^4^ ^]^ and natural products.^[^ ^5^, ^6^ ^]^ Thereby, they serve as versatile precursors for various functional groups, for example, amines, amides, amidines, tetrazoles, heterocycles, and other carboxylic derivatives.^[^ ^7^, ^8^, ^9^ ^]^ Numerous efforts have been devoted to introducing nitrile groups to organic molecules. Traditional synthetic routes include the Sandmeyer cyanation,^[^ ^10^ ^]^ Rosenmund‐von Braun reaction,^[^ ^11^ ^]^ and Kolbe nitrile synthesis,^[^ ^12^ ^]^ all proceeding by nucleophilic substitution requiring stoichiometric amounts of highly toxic copper or alkali cyanides under harsh conditions and producing equimolar amounts of metal waste and other environmentally hazardous by‐products, such as toxic HCN. Ammoxidations of toluene to benzonitrile have been implemented in large‐scale industrial manufacturing.^[^ ^13^, ^14^, ^15^, ^16^, ^17^ ^]^ However, they do not tolerate functionalized substrates due to the necessity of elevated temperatures, high pressures, and a substantial excess of ammonia. Additionally, the industrial availability of substituted toluenes is limited, restricting the applicability of this method predominantly to products such as benzonitrile and halobenzonitriles.^[^ ^14^, ^15^ ^]^ Transition metal‐catalyzed cyanation of aryl (pseudo)halides and organometallic reagents has been emerging as an alternative strategy for the synthesis of aryl nitriles. Yet, the majority of these procedures rely on expensive transition metal catalysts (e.g., Cu,^[^ ^18^, ^19^ ^]^ Pd,^[^ ^20^, ^21^, ^22^, ^23^, ^24^ ^]^ Rh,^[^ ^25^, ^26^ ^]^ or Ru^[^ ^27^, ^28^ ^]^) by using different substrates, including alkenes,^[^ ^29^ ^]^ amines,^[^ ^30^ ^]^ amides,^[^ ^31^ ^]^ carboxylic acids,^[^ ^32^ ^]^ primary alcohols,^[^ ^33^ ^]^ and aldehydes.^[^ ^34^, ^35^ ^]^ This strategy is frequently limited by high costs and toxicity concerns related to metal catalysts and the generation of stoichiometric amounts of metal waste, similar to conventional methodologies. Besides, precise control over the cyanide concentration is essential to preserve the catalyst activity, as it is often affected by the formation of inactive cyano‐transition metal complexes.^[^ ^36^ ^]^

Due to the availability of aldehydes, nitriles have been synthesized from them through aldoxime intermediates using various chemical dehydrating agents, including H‐zeolites,^[^ ^37^ ^]^ *N*‐thiocyanato‐dibenzenesulfonimide (NTSI),^[^ ^38^ ^]^ Castro's reagent (BOP),^[^ ^39^ ^]^ PhSe(O)OH,^[^ ^40^ ^]^ or by undergoing oxidative transformations using (COCl)_2_/DMSO/Et_3_N.^[^ ^41^ ^]^ In these strategies, the oxime is initially isolated, followed by the activation of the oxime hydroxyl group to afford the nitrile by elimination.^[^ ^42^, ^43^, ^44^ ^]^ The practicality and efficiency of this strategy have inspired the development of several one‐pot protocols for the direct conversion of aldehydes to nitriles, employing hydroxylamine or ammonia in conjunction with various activating agents.^[^ ^45^ ^]^ Yet, these protocols necessitate harsh conditions to proceed, or the employed agents, such as CuCl_2_/NaOMe/O_2_,^[^ ^46^ ^]^ Pb(OAc)_4_,^[^ ^47^ ^]^ H_2_O_2_,^[^ ^48^ ^]^ NBS,^[^ ^49^ ^]^ IBX,^[^ ^50^ ^]^ and NaICl_2_,^[^ ^51^ ^]^ are mostly intolerant of many functional groups. Recently, there have been attempts to find greener options to generate nitriles from aldehydes, including the use of an ionic liquid environment to promote the transformation^[^ ^52^ ^]^ as well as biphasic systems enabling the chemical conversion of aldehydes to aldoximes followed by enzymatic dehydration to nitriles in a one‐pot cascade, typically affording moderate yields (50–60%).^[^ ^53^, ^54^ ^]^ These strategies operate under mild conditions and produce minimal hazardous by‐products; however, challenges remain regarding enzyme stability and compatibility. Enzymatic processes often depend on cofactors, regeneration systems, or whole‐cell catalysts (e.g., *E. coli*), which can increase operational complexity, cost, and purification demands. Likewise, the practical application of ionic liquids can be limited by their high viscosity, mass‐transfer constraints, and restricted commercial availability for large‐scale processes. All these methodologies stimulate the development of nitrile syntheses from aldehydes further, highlighting the necessity for procedures that circumvent tedious workups, high costs, and toxicity of precursors; undesired side‐product formation; and limited versatility of functional groups.

In our prior work on oxime carbamates (also known as carbamoyloximes) as mechanophores,^[^ ^55^, ^56^, ^57^ ^]^ we coincidentally found that carbamoylaldoximes formed nitriles efficiently through pericyclic *syn* elimination. The mechanism was unraveled by a series of trapping experiments with 5,5‐dimethyl‐1‐pyrroline‐*N*‐oxide (DMPO), where the DMPO concentration remained constant during nitrile formation, and also by DFT calculations through a potential energy surface scan of the N − O stretching coordinate.^[^ ^56^ ^]^ Herein, we report a protocol for the alcoholysis of aldehyde‐derived aldoximes with DMCC to produce *N,N‐*dimethylcarbamoyloximes, which subsequently convert to nitriles (Scheme 1a). This approach relies on widely available commercial chemicals, proceeds with broad functional group tolerance, and minimizes the need for extensive purification of the nitrile product as the final step of the reaction proceeds as pericyclic *syn* elimination, generating only the desired nitrile, CO_2_, and HNMe_2_ (Scheme 1b). The oximes were first prepared by condensing aldehydes (**1**) with hydroxylamine hydrochloride (HONH_3_^+^Cl^−^), thereby introducing nitrogen at the carbonyl carbon center and forming aldoximes (**2**). Subsequently, oximes (**2**) were treated with a pyridine/Et_3_N base mixture and DMCC to afford desired carbamoylaldoximes (**3**) through nucleophilic addition‐elimination, resulting in good to excellent yields (80 to 98%). Deprotonation with pyridine or Et_3_N individually proved ineffective, and the reaction occurred only in the presence of their combined mixture, presumably due to pyridine's role in activating DMCC by forming a pyridinium adduct and Et_3_N's efficient scavenging of HCl, thus maintaining optimal basic reaction conditions (details can be found in the SI in Section 2.7, Figures S30 and S31). Subsequent heating in MeCN at 80 °C or DMSO‐*d*_6_ at 90 °C prompted the elimination of dimethylamine and formation of nitriles (**4**, Scheme 1). In the case of the volatile carbamoylaldoximes **3n** and **3o**, formation of the corresponding nitriles was detected under ambient conditions, indicating spontaneous conversion that circumvented the need for thermal activation.

**Scheme 1:**
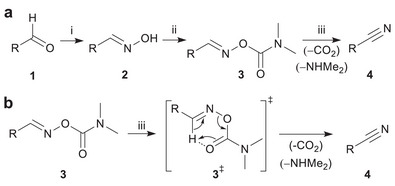
a) General procedure for the synthesis of nitriles. (i) HONH_3_^+^Cl^−^ (1.1 equiv.), pyridine (3 equiv.), EtOH, 80 °C, 24 hours. (ii) DMCC (1.5 equiv.), Et_3_N (1.5 equiv.), pyridine (1.5 equiv.), MeCN, r.t., overnight. (iii) 80 °C, 24 hours in MeCN or 90 °C, 24 hours in DMSO‐*d*_6_ or room temperature reaction conditions in step (ii) for volatile carbamoylaldoxime derivatives (**3n** and **3o**). b) Pericyclic *syn* elimination mechanism of carbamoylaldoximes (**3**) upon heating.

Benzaldehyde **1a** served as the model substrate to test firstly DMCC‐mediated formation of *O‐*dimethylcarbamoyloxime **3a** through benzaldehyde oxime **2a** and secondly elimination of **3a** to benzonitrile **4a** via formation of a leaving group (Table 1, Entry 1).

**Table 1:** Substrate scope for the conversion of aldehydes to nitriles.^[^ ^a^ ^]^

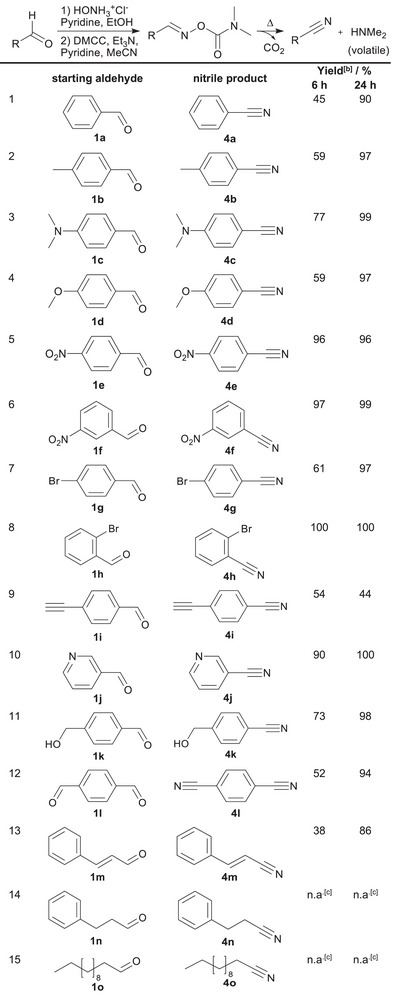

^[a]^ Nitrile conversions were performed in DMSO‐*d*_6_ at 90 °C for 6 hours and 24 hours. NMR tubes of each substrate in an approximate final concentration of 0.1 m were prepared using 0.5 mL of DMSO‐*d*_6_, sealed, and put in a thermostat bath at 90 °C. The tubes were removed, cooled down to r.t., and ^1^H NMR spectra were recorded both in the presence and absence of 8.35 µL of DMF to avoid possible peak overlapping of DMF and diagnostic aromatic peaks of nitriles and/or *O*‐dimethylcarbamoyloximes.

^[b]^ Yields were determined by ^1^H NMR analysis.

^[c]^ Formation of **3n** and **3o** consequently produced corresponding nitriles **4n** and **4o** at room temperature without further need to heat in DMSO‐*d*_6_. **4n** and **4o** were isolated via vacuum distillation due to their low boiling point.

A kinetic analysis of the thermal conversion was conducted by variable temperature (VT) NMR measurements, with temperatures ranging from 50 °C to 90 °C to monitor the transformation of **3a** to **4a**, obtaining the reactant concentrations through the peak integrals (Table S3 and Figure S2). The elimination was found to proceed on useful timescales at temperatures ≥ 80 °C, and thus subsequent experiments were conducted at or above this threshold. These results were consistent with related electrocyclic rearrangement reactions, such as those described by Nantz and coworkers,^[^ ^58^ ^]^ and also by us.^[^ ^56^ ^]^

The synthetic protocol for **1a** was applied to a broad range of aldehydes, including aromatic, heterocyclic, saturated, unsaturated, and aliphatic derivatives. Phenyl substrates bearing electron‐withdrawing substituents **3e‐h** formed nitriles in shorter reaction times (Table 1, Entries 5–8) relative to those with electron‐donating groups **3b‐d, 3k**. (Table 1, Entries 2–4,11).

Phenyl substitutions consistently facilitated the rearrangement, outperforming the core derivative **3a**. Thermal reactivity studies were performed for all derivatives to underline the general applicability of the method. As a more specific example, 4‐methoxybenzaldehyde *O*‐dimethylcarbamoyloxime **3d** converted to the corresponding nitrile **4d** with 59% yield when heated for 6 hours and 97% for 24 hours at 90 °C in DMSO‐*d*_6_, separately (Figure 1).

**Figure 1:**
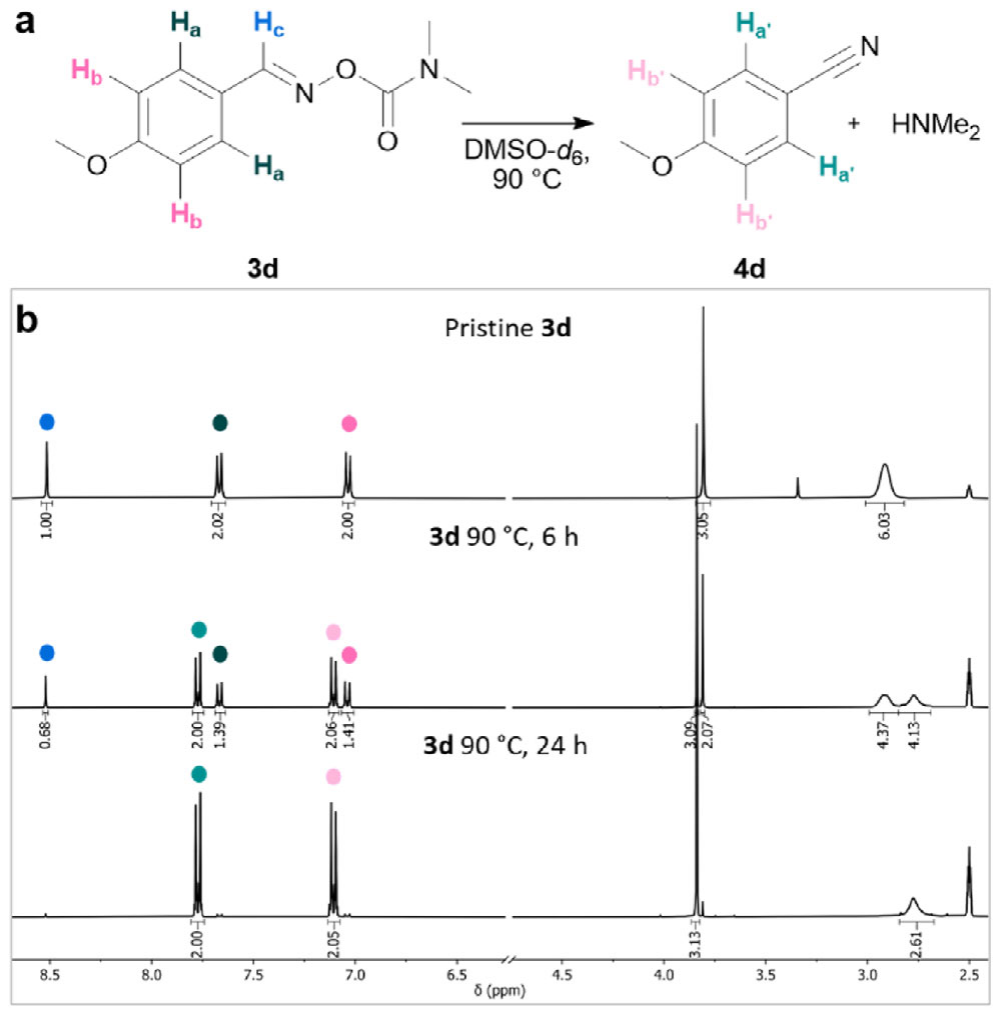
Thermal reactivity analysis of **3d**. a) Scheme denoting all measured protons. b) From top to bottom: ^1^H NMR spectra corresponding to pristine 4‐methoxybenzaldehyde *O‐*dimethylcarbamoyloxime **3d** and 4‐methoxybenzonitrile **4d** formed after heating **3d** for 6 hours and 24 hours at 90 °C in DMSO‐*d*_6_. *δ* = 2.77 ppm appears, as the *δ* = 2.92 ppm disappears due to the formation and evaporation of HNMe_2_ over time. Details can be found in the SI in Section 2.2.

4‐Methoxy substrates **1d** and **2d** were selected as the test precursors for further studies, given moderate nitrile formation from **3d** to **4d** within 6 hours compared to most of the other analogues (Table 1, entry 4).

Among the substrates, the alkynyl derivative **3i** afforded **4i** with the lowest efficiency, attributed to competing side reactions of the alkynyl group adjacent to the nitrile functionality (Table 1, entry 9). Vinyl species were likely generated through hydrogen transfer from the released dimethylamine, as indicated by the gradual appearance of new ^1^H signals between 5.00 and 7.50 ppm with prolonged heating. Cyano‐substituted heterocycles represent important scaffolds in numerous medicinal compounds, and the application of our methodology enabled the efficient synthesis of heterocycle **4j** from **3j** in excellent yield (Table 1, entry 10). The alkyl alcohol substrate **2k** did not exhibit competing reactivity, as the p*K*_a_ difference between the oxime and the aliphatic hydroxyl group was sufficient to prevent interference of undesired side reactions (Table 1, entry 11). α,β‐Unsaturation showed a significant influence on the conversion due to the volatile character of **3n** compared to **3m**, surpassing an additional heating step (Table 1, entries 13 and 14); however, it proceeded smoothly for both derivatives **4m** and **4n**. The alkyl derivative **3o** exhibited similar volatile behavior to **3n** (Table 1, entry 15). Upon vacuum distillation, both **3n** and **3o** resulted in full conversion to the corresponding nitriles **4n** and **4o**, eliminating the need to isolate the intermediate carbamoylaldoximes.

Seeking a protocol enhancing the step economy, we next examined the possibility of transforming aldoximes into nitriles without isolation of the carbamoyloxime adducts. A semi‐one‐pot protocol was first employed, transforming aldoxime **2d** into nitrile **4d** through intermediate **3d**, considering the differing solvent conditions for oxime and carbamoyloxime formation. Syntheses of **2d** and **3d** were monitored by thin‐layer chromatography (TLC) to control how to proceed with the next step, and no work‐up was performed in between. Heating the reaction mixture at 80 °C for 6 hours provided **4d** in 17% yield, whereas prolonged heating for 24 hours afforded **4d** in full conversion (Figure S16). Product **4d** was readily isolated by simple extraction with CH_2_Cl_2_ to remove Et_3_N·HCl, obviating the need for chromatography.

Subsequent studies focused on the direct conversion of aldehydes to nitriles, bypassing the isolation of both oxime and carbamoyloxime intermediates. The one‐pot transformation was conducted by performing all reaction steps in MeCN, including the synthesis of oxime **2d** from aldehyde **1d**. Only the target product **4d** was isolated and redissolved in CDCl_3_ to track the conversion. **4d** was obtained in 93% NMR yield after 6 hours and in quantitative NMR yield upon extending the reaction time to 24 hours (Figure 2).

**Figure 2:**
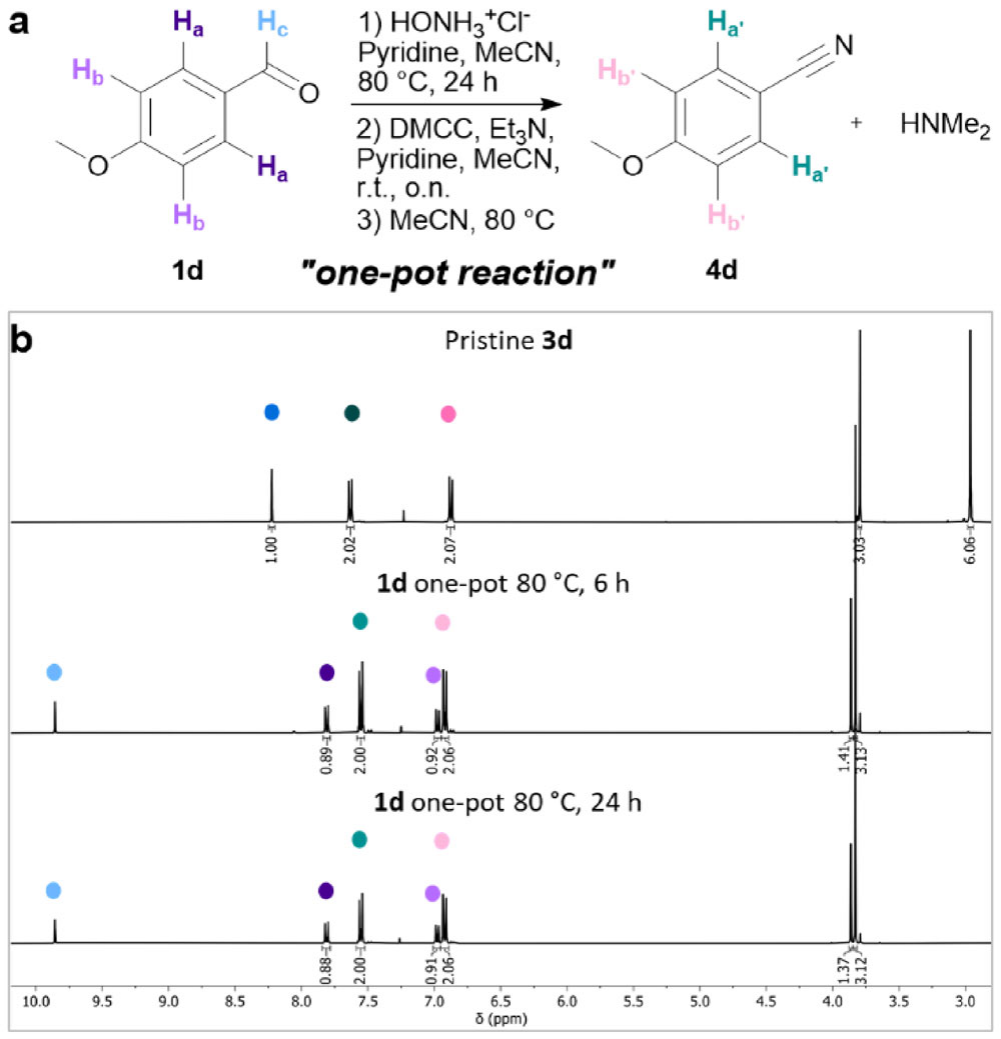
One‐pot synthesis of 4‐methoxybenzonitrile **4d** starting from 4‐methoxybenzaldehyde **1d**. a) Reaction scheme denoting all measured protons. b) From top to bottom: ^1^H NMR spectra corresponding to pristine 4‐methoxybenzaldehyde *O*‐dimethylcarbamoyloxime **3d**, isolated 4‐methoxybenzonitrile **4d** formed via one‐pot synthesis starting from **1d,** and after heating intermediate **3d** for 6 hours and 24 hours at 80 °C in MeCN (Spectra were taken in CDCl_3_, and the middle and bottom ^1^H NMRs consist of 4‐methoxybenzaldehyde **1d** that remains unreacted further. Detailed ^1^H and ^13^C NMR analyses can be found in Figures S19 and S20 in the SI).

However, the conversion of aldehyde **1d** to oxime **2d** under reflux conditions remained incomplete, affording approximately 70% NMR yield even with prolonged reaction time. This limitation was addressed in our microwave‐assisted synthesis trials, where increasing the excess of hydroxylamine hydrochloride proved highly effective in improving conversion.

Microwave (MW) irradiation allows for rapid, direct, and uniform heating of reaction mixtures, making it an attractive method that has been widely employed as a heating source in various chemical processes. Encouraged by the high conversion according to the NMR yields obtained in the semi‐one‐pot and one‐pot protocols for 4‐methoxybenzonitrile **4d**, we aimed to shorten the reaction time while maintaining or improving efficiency. Notably, under microwave‐assisted conditions, **4d** was synthesized from **1d** in approximately 3 hours overall, representing a significant improvement over the conventional protocols if needed (Figure 3). Full conversion to **4d** was achieved in 60 minutes, while ^1^H NMR investigations demonstrated 80% NMR yield for the synthesis of **2d** from **1d** in 30 minutes. Extending the reaction time had no further effect on the yield of **2d**, whereas doubling the equivalency of hydroxylamine hydrochloride resulted in full conversion, affording **2d** in quantitative yield during our parallel trials. The formation of carbamoyloxime **3d** was initially monitored by TLC, and intermediate **3d** was neither isolated nor recorded separately, as the reaction was conducted directly to obtain **4d** in a one‐pot process. During the synthesis of **3d**, the reaction temperature was adjusted from room temperature to 40 °C, the lowest setting compatible with the microwave synthesizer. Complete conversion to nitrile **4d** was achieved after 60 minutes; notably, 55% NMR yield was obtained within just 30 minutes.

**Figure 3:**
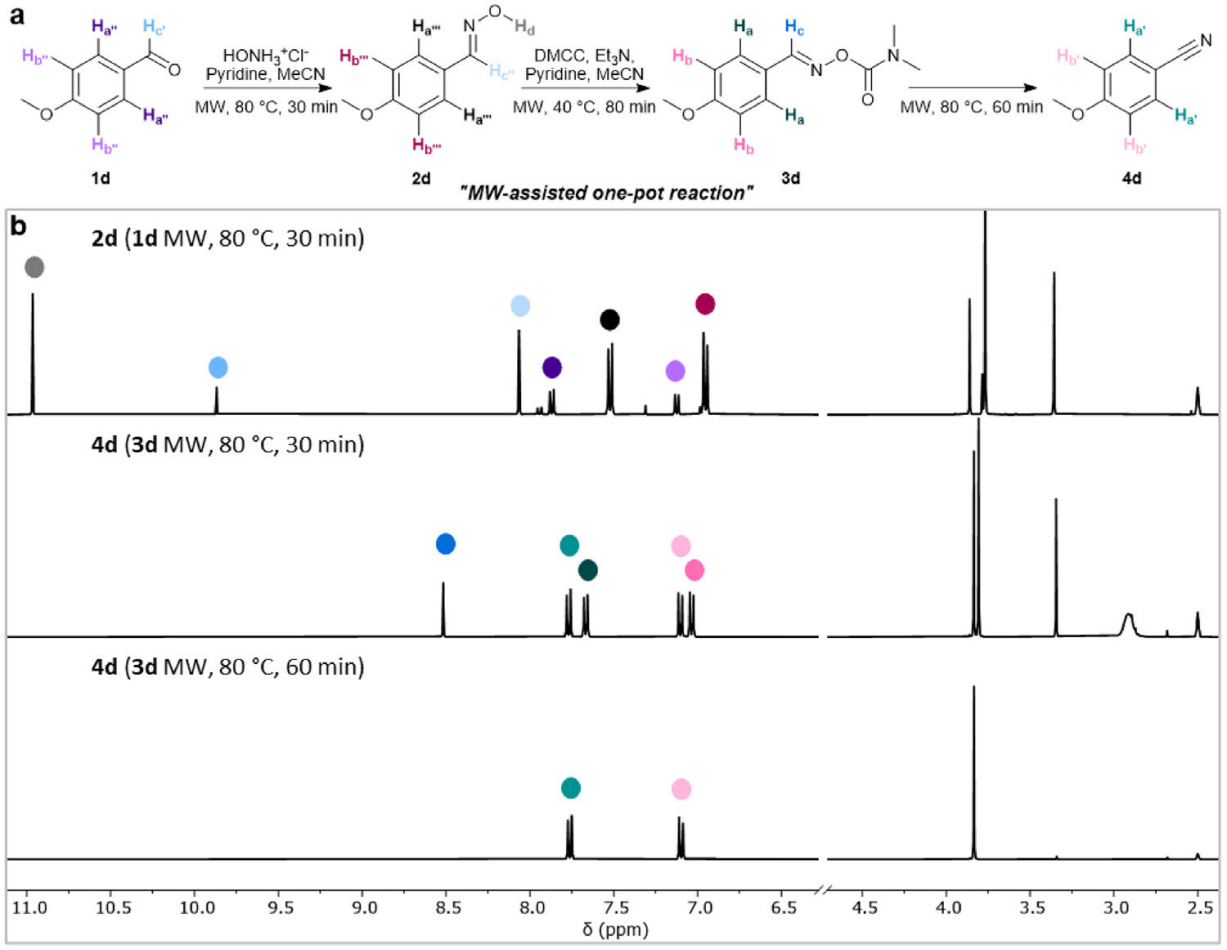
MW‐assisted one‐pot synthesis of 4‐methoxybenzonitrile **4d** starting from 4‐methoxybenzaldehyde **1d**. a) Reaction scheme denoting all measured protons. b) From top to bottom: Stacked ^1^H NMR spectra showing the formation of oxime **2d** from aldehyde **1d** after microwave irradiation at 80 °C for 30 minutes in MeCN, partial conversion to **4d** after MW‐assisted heating of intermediate **3d** for 30 minutes at 80 °C in MeCN, and complete conversion to **4d** after 60 minutes in identical conditions (spectra were recorded in DMSO‐*d*_6_. ^1^H NMRs consist of residual 4‐methoxybenzaldeyde **1d** (top) and also incomplete formation of **4d**, consistent with intermediate **3d** formation (middle). The middle spectrum was reported to show the formation of **3d**. Detailed ^1^H and ^13^C NMR analyses can be found in Figures S22 and S23).

A scale‐up one‐pot synthesis of **4d** from precursor **1d** was carried out to further show the practicality of this method. The preparation of 16.2 g of *N,N*‐dimethylcarbamoyloxime **3d** necessitated a 2.6‐fold increase in the equivalents of DMCC, pyridine, and Et_3_N; however, following completion of the one‐pot reaction, only the nitrile **4d** was isolated through a straightforward work‐up. The conversion was investigated by ^1^H NMR spectroscopy in CDCl_3,_ and 9.7 g of **4d** were obtained in 98% NMR yield after 24 hours, starting from 10.0 g of **1d** (Figures S24‐26). These results demonstrate that nitrile formation can be effectively scaled up without the need to isolate any intermediates, even under large‐scale conditions.

As a conventional approach, Rosenmund‐von Braun cyanation was also employed to convert 4‐bromoanisole to **4d**, using CuCN under standard conditions to demonstrate the advantages of our method. The reaction was carried out on a small scale to minimize the generation of excess metal waste and toxic HCN gas. After refluxing in DMF for 24 hours, **4d** was isolated and purified by column chromatography, resulting in a moderate to good yield (68%) (Figures S27‐29). However, this method still presents safety concerns, requires careful handling, and involves an additional purification step.

We have developed an operationally simple protocol for the synthesis of nitriles from aldehydes via carbamoylaldoxime intermediates, avoiding the use of toxic cyanide sources, precious metal catalysts, and harsh conditions. This metal‐free and complex reagent‐free approach employs the alcoholysis of readily accessible aldoximes with DMCC to furnish *N,N‐*dimethylcarbamoyloximes, which undergo clean conversion to nitriles. In contrast to previously reported methods that rely on acidic conditions (e.g., TFA, CSA, TfOH),^[^ ^59^, ^60^, ^61^ ^]^ which may be problematic for acid‐sensitive groups to convert *O‐*acyl oximes into nitriles, our approach achieves this transformation under acid‐free conditions simply by heating. The method demonstrates broad functional group compatibility and can be executed in a one‐pot, solvent‐independent approach, significantly reducing purification steps to only a simple extraction in the final step. Importantly, this transformation proceeds cleanly with minimal by‐product formation, releasing only volatile CO_2_ and HNMe_2_, underscoring its potential as an efficient, scalable, benign alternative for established nitrile syntheses. The microwave‐one‐pot approach improved the synthesis of nitrile derivatives from aldehydes, achieving completion in roughly 3 hours, marking a substantial reduction in reaction time while maintaining high efficiency.

## Conflict of Interest

There are no conflicts to declare.

## Supporting information



Supporting Information

## Data Availability

The data that support the findings of this study including experimental procedures, molecular characterization data, and metadata, are openly available in Chemotion‐Repository athttp://doi.org/10.14272/collection/ASI_2025‐09‐12.^[^ ^62^ ^]^
