# Supplementary material for: Efficient, Functional Group‐Tolerant, and Catalyst‐Free Nitrile Formation from Aldehydes
Source: Chemistry. 2025 Nov 10;31(71):e02629. doi: 10.1002/chem.202502629 (PMC12734648; doi:10.1002/chem.202502629)
Supplement: Supplementary file 1 — Supporting Information [file CHEM-31-e02629-s001.pdf]

Supporting Information

for

Efficient, functional group-tolerant, and catalyst-free nitrile formation from aldehydes

Simay Aydonat,[a,b,c] Davide Campagna,[b,c] and Robert Göstl[a,b]*

[a]Department of Chemistry and Biology, University of Wuppertal, Gaußstr. 20, 42119 Wuppertal, Germany

[b]DWI – Leibniz Institute for Interactive Materials, Forckenbeckstr. 50, 52056 Aachen, Germany

[c]Institute of Technical and Macromolecular Chemistry, RWTH Aachen University, Worringerweg 2, 52074 Aachen, Germany

*goestl@uni-wuppertal.de

Table of Contents

1. Materials and methods 3
1.1. Materials 3
1.2. Methods 3
2. Experimental procedures 4
2.1. General synthesis procedures 4
2.1.1. General procedure for the synthesis of aldoximes (**2a-o**) (step 1) 4
2.1.2. General procedure for the synthesis of carbamoyloximes (**3a-o**) (step 2) 5
2.1.3. General procedure for the formation of nitriles (**4a-o**) (step 3) 5
2.2. Thermal reactivity experiments 6
2.2.1. Nitrile formation monitoring (benzaldehyde *O*-dimethylcarbamoyloxime **3a** to benzonitrile **4a**) 6
2.2.2. Temperature monitoring (benzaldehyde *O*-dimethylcarbamoyloxime **3a** to benzonitrile **4a**) 6
2.2.3. Thermal exposure time evaluation (benzaldehyde *O*-dimethylcarbamoyloxime **3a** to benzonitrile **4a**) 8
2.2.4. Solvent evaluation (benzaldehyde *O*-dimethylcarbamoyloxime **3a** to benzonitrile **4a**) 8
2.2.5. Substrate scope of diverse *O*-dimethylcarbamoyloximes 8
2.3. Semi-one-pot and one-pot transformation of aldehydes to nitriles 17
2.3.1. Semi-one-pot synthesis of nitriles (4-methoxybenzaldehyde oxime **2d** to 4-methoxybenzonitrile **4d**) 17
2.3.2. One-pot synthesis of nitriles (4-methoxybenzaldehyde **1d** to 4-methoxybenzonitrile **4d**) 19
2.4. One-pot formation of nitriles *via* microwave-assisted synthesis 21
2.5. Large-scale one-pot formation of nitriles (4-methoxybenzaldehyde **1d** to 4-methoxybenzonitrile **4d**) 23
2.6. Rosenmund-von Braun cyanation (4-methoxybenzonitrile **4d**) 25
2.7. Initial control experiments on the role of the base mixture (pyridine/Et_3N) for the formation of benzaldehyde *O*-dimethylcarbamoyloxime **3a** from benzaldehyde oxime **2a** 27
3. Molecular characterization data 29
3.1. Characterization data of products and intermediates 29
3.2. ^{1}H NMR, ^{13}C NMR, and mass spectra 37
4. Supporting references 81

1. Materials and methods

1.1. Materials

Solvents and reagents were purchased from different suppliers (Sigma Aldrich, TCI, Acros Organics, Oakwood Chemicals, Alfa Aesar) and were used as delivered without further purification unless otherwise noted. Thin-layer chromatography (TLC) was performed on Merck TLC silica gel 60 F254 TLC plates or ALUGRAM® SIL G/UV254 plates with a fluorescent indicator. Purification by flash chromatography was performed manually on silica gel (40-63 µm) or on a Reveleris X2 Flash Chromatography System (Büchi), equipped with UV and ELSD detectors with commercially available FlashPure ID cartridges (particle size: 35-45 µm, pore size: 53-80 Å) from the same provider, if needed.

1.2. Methods

Microwave-assisted one-pot synthesis was performed using a Biotage® Initiator EXP EU microwave reactor with a maximum power of 400 W.

General ^{1}H and ^{13}C NMR spectra were recorded on a 400 MHz Bruker Avance 400 spectrometer (^{13}C: 101 MHz) or a 600 MHz Bruker Avance III 600 spectrometer (^{13}C: 151 MHz). Variable temperature (VT) NMR experiments were performed on a Bruker AV III 300 MHz Spectrometer. Reported chemical shifts are given in δ units with respect to the residual protonated signal in deuterated solvents: δ = 2.50 ppm for DMSO-d_6 and δ = 7.26 ppm for $CDCl_3$. The multiplicities of the peaks were indicated as follows: s = singlet, d = doublet, t = triplet, q = quartet, p = pentet, m = multiplet, ddd = doublet of doublet of doublets, dd = doublet of doublets, dt = doublet of triplets. ^{1}H NMR was recorded applying 32 scans on a 400 MHz Bruker Avance 400 spectrometer for all thermal reactivity experiments to achieve higher sensitivity to monitor the nitrile formation. No internal standard (IS) was used unless otherwise noted, and integrals were normalized to a non-overlapping proton signal during NMR yield calculations in Sections 2.2, 2.3, and 2.4. Additionally, ^{1}H NMR spectra in Section 2.2.5 were recorded in the presence of dimethylformamide (DMF) with a known concentration as an IS to increase the precision. NMR yields were determined using ^{1}H NMR spectroscopy with DMF as an IS. Integrals were corrected based on the number of protons and compared to the known amount of IS added, if needed.

Electrospray ionization-high-resolution mass spectrometry (ESI-HRMS) spectra were recorded on a microTOF-QII instrument from Bruker Daltonik. Field desorption (FD) mass spectrometry measurements were conducted on a JEOL AccuTOF-GCX spectrometer, while atmospheric pressure chemical ionization (APCI) mass spectrometry measurements were performed on a Bruker microTOF instrument.

2. Experimental procedures

2.1. General synthesis procedures

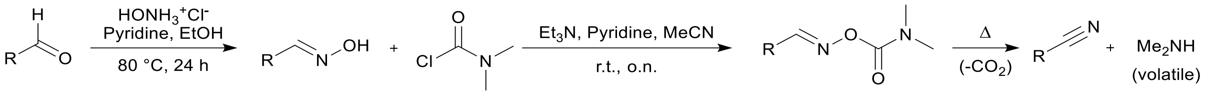

Figure S1. General reaction scheme followed for the formation of nitriles through the syntheses of aldoximes and carbamoyloximes.

2.1.1. General procedure for the synthesis of aldoximes (**2a-o**) (step 1)

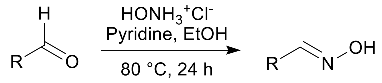

Aldehyde (1.0 equiv.) and hydroxylamine hydrochloride ($HONH_3^+Cl^-$, 1.1 equiv.) were dissolved in abs. EtOH. Pyridine (3.0 equiv.) was added, and the mixture was stirred and refluxed for 24 h. Afterwards, all volatiles were removed *in vacuo*. H_2O (20 mL) was added to the oily residue and extracted with Et_2O (2×20 mL) and washed with brine (1×20 mL). The combined organic layers were dried over $MgSO_4$, filtered, and the solvent was removed *in vacuo*. Aldoximes were obtained without further purification with the isolated yields reported in Table S1 (terephthalaldehyde dioxime **2l** was exceptionally synthesized with the following equivalencies: aldehyde:$HONH_3^+Cl^-$:pyridine = 1.0 equiv.:2.2 equiv.:3.0 equiv.).

Table S1. List of aldoximes synthesized ([a] isolated yield was recalculated after impurity % was reduced, [b] purity % of the compound was calculated according to the ^{1}H NMR signals of unreacted starting materials, trace amount of by-products, etc.).

entry	compound name	substituent / R–C=N–OH	yield / %[a]	purity / %[b]	^{1}H NMR, ^{13}C NMR, and MS
1	**2a**	Ph (C_6H_5)	83	100	Figure S32, Figure S33, Figure S34
2	**2b**	4-Me-C_6H_4	88	92	Figure S35, Figure S36, Figure S37
3	**2c**	4-Me_2N-C_6H_4	85	85	Figure S38, Figure S39, Figure S40
4	**2d**	4-MeO-C_6H_4	85	100	Figure S41, Figure S42, Figure S43
5	**2e**	4-NO_2-C_6H_4	91	93	Figure S44, Figure S45, Figure S46
6	**2f**	3-NO_2-C_6H_4	80	100	Figure S47, Figure S48, Figure S49
7	**2g**	4-Br-C_6H_4	89	94	Figure S50, Figure S51, Figure S52
8	**2h**	2-Br-C_6H_4	87	100	Figure S53, Figure S54, Figure S55
9	**2i**	4-Alkynyl	91	96	Figure S56, Figure S57, Figure S58
10	**2j**	3-Pyridyl	87	100	Figure S59, Figure S60, Figure S61
11	**2k**	4-CH_2OH-C_6H_4	80	95	Figure S62, Figure S63, Figure S64
12	**2l**	4-NOH-C_6H_4	98	100	Figure S65, Figure S66, Figure S67
13	**2m**	4-Cinnamyl-C_6H_4	85	94	Figure S68, Figure S69, Figure S70
14	**2n**	4-Propyl-C_6H_4	90	93	Figure S71, Figure S72, Figure S73
15	**2o**	*n*-$C_{11}H_{24}$	99	100	Figure S74, Figure S75, Figure S76

2.1.2. General procedure for the synthesis of carbamoyloximes (**3a-o**) (step 2)

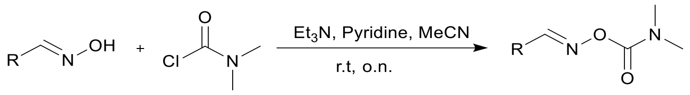

Aldoxime (1.0 equiv.), dry Et_3N (1.5 equiv.), and pyridine (1.5 equiv.) were dissolved in dry MeCN in a Schlenk flask under an Ar atmosphere. Subsequently, *N,N*-dimethylcarbamoyl chloride (1.5 equiv.) was injected, whereupon the color of the solution turned from transparent to light yellow. The mixture was then stirred overnight at r.t. All volatiles were removed *in vacuo*, the residue was dissolved in CH_2Cl_2 (20 mL), washed with H_2O (2×25 mL), and brine (1×25 mL). The organic layer was dried over $MgSO_4$ and filtered. The solvent was removed *in vacuo,* and carbamoyloximes were mostly obtained without further purification. Column chromatography was employed only when necessary, particularly to prevent possible overlapping signals in the ^1H NMR spectra during subsequent thermal studies, as reported in Table S2.

Table S2. List of *O*-dimethylcarbamoyloximes synthesized ([a] isolated yield).

entry	compound name	substituent / R–C=N–O–CO–NMe$_2$	yield / %[a]	^1H NMR, ^{13}C NMR, and MS
1	**3a**	Ph (C_6H_5)	93	Figure S77, Figure S78, Figure S79
2	**3b**	4-Me-C_6H_4	92	Figure S80, Figure S81, Figure S82
3	**3c**	4-Me$_2$N-C_6H_4	89	Figure S83, Figure S84, Figure S85
4	**3d**	4-MeO-C_6H_4	90	Figure S86, Figure S87, Figure S88
5	**3e**	4-NO_2-C_6H_4	90	Figure S89, Figure S90, Figure S91
6	**3f**	3-NO_2-C_6H_4	85	Figure S92, Figure S93, Figure S94
7	**3g**	4-Br-C_6H_4	90	Figure S95, Figure S96, Figure S97
8	**3h**	2-Br-C_6H_4	80	Figure S98, Figure S99, Figure S100
9	**3i**	4-Alkynyl	90	Figure S101, Figure S102, Figure S103
10	**3j**	3-Pyridyl	98	Figure S104, Figure S105, Figure S106
11	**3k**	4-CH_2OH-C_6H_4	90	Figure S107, Figure S108, Figure S109
12	**3l**	4-NOH-C_6H_4	98	Figure S110, Figure S111, Figure S112
13	**3m**	4-Cinnamyl-C_6H_4	85	Figure S113, Figure S114, Figure S115
14	**3n**	4-Propyl-C_6H_4	n.a.	n.a.
15	**3o**	*n*-$C_{11}H_{24}$	n.a.	n.a.

2.1.3. General procedure for the formation of nitriles (**4a-o**) (step 3)

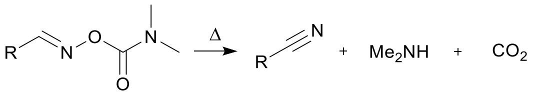

The solutions of carbamoyloximes were subjected to heat to initiate the nitrile generation. After temperature scanning (*cf.* Section 2.2.2), 80 °C or 90 °C was chosen as the temperature, depending on the temperature requirements of the solvent of choice (80 °C for MeCN, and 90 °C for DMSO-*d*$_6$). Detailed information is provided in the next Section 2.2.

2.2. Thermal reactivity experiments

2.2.1. Nitrile formation monitoring (benzaldehyde *O*-dimethylcarbamoyloxime **3a** to benzonitrile **4a**)

The initial heating experiment was carried out with benzaldehyde *O*-dimethylcarbamoyloxime **3a** in DMSO-d_6 for qualitative analysis. **3a** and DMSO-d_6 were chosen as the preliminary trial substrate and solvent of choice, respectively, to eliminate the substituent effect and the overlap of the diagnostic ^{1}H NMR peaks of **3a** and the corresponding benzonitrile **4a** formed. For qualitative analysis, the two NMR tubes of **3a** in 0.6 mL of DMSO-d_6 were sealed and put in a thermostat bath at the defined temperature of 90 °C. After 6 and 24 h, the tubes were removed, respectively, cooled down, and the ^{1}H NMR spectra were recorded for both. Later, 10 µL of DMF was added as an internal standard, reaching a final concentration of 0.1 M, and the ^{1}H NMR spectra were additionally recorded. Similarly, 10 µL of DMF was added to a parallel pristine sample **3a** in 0.6 mL of DMSO-d_6 that was not heated, and directly submitted to ^{1}H NMR to obtain the starting spectrum, and the NMR yield was calculated (Figure S3, Table S4, Entry 1) The details of this experiment, characterization data, and metadata are available under the reaction DOI: https://dx.doi.org/10.14272/reaction/SA-FUHFF-UHFFFADPSC-JFDZBHWFFU-UHFFFADPSC-NUHFF-NUHFF-NUHFF-ZZZ.

2.2.2. Temperature monitoring (benzaldehyde *O*-dimethylcarbamoyloxime **3a** to benzonitrile **4a**)

The temperature was varied using freshly prepared substrate **3a** at the same concentration to identify the onset temperature for the formation of product **4a**, aiming to maximize efficiency at the lowest possible temperature while ensuring sufficient product yield. The NMR tube was then placed inside the NMR spectrometer, and ^{1}H NMR spectra were recorded by VT NMR. Then, the sample was heated to the defined temperature inside the spectrometer, and multiple spectra were recorded at specific intervals. Four spectra were obtained for every temperature scanned, including the initial spectrum at that particular temperature; at 50 °C every 20 min, 60 °C every 20 min, 70 °C every 20 min, 80 °C every 20 min, and 90 °C every 20 min (Table S3, Figure S2). By measuring the NMR integrals, the concentrations of the decreasing **3a** and of the increasing nitrile **4a** were calculated at every time interval. Due to overlapped NMR shifts of **3a** at 7.50 ppm for 2H and 1H (multiplet instead of two separate triplets), the integral for 2H of **3a** was calculated by dividing the integral of 3H ($I_{\text{cox, ar, avg., (3H)}}$) by 1/3, and subsequently, multiplying by 2/3. Thus, the NMR yield was calculated according to the integrals of 2H of **3a** at 7.50 ppm and **4a** at 7.58 ppm. Since the heating took place continuously in the same NMR tube, DMF was not added to the system as an internal standard. VT NMR measurements indicated a pronounced acceleration in benzonitrile formation above 80 °C, and with a noticeable increase in rate at 90 °C. Benzonitrile (**4a**): **^{1}H NMR** (300 MHz, DMSO-d_6 [2.50 ppm]): δ_H / ppm = 7.83–7.76 (m, 2H), 7.76–7.69 (m, 1H), 7.63–7.54 (m, 2H). The details of this experiment, characterization data, and metadata are available under the reaction DOI: https://dx.doi.org/10.14272/reaction/SA-FUHFF-UHFFFADPSC-JFDZBHWFFU-UHFFFADPSC-NUHFF-NUHFF-NUHFF-ZZZ.1 and analysis DOI: https://dx.doi.org/10.14272/SA-FUHFF-UHFFFADPSC-JFDZBHWFFU-UHFFFADPSC-NUHFF-NUHFF-NUHFF-ZZZ/CHMO0000593.1.

Table S3. List of temperatures scanned and recorded by VT NMR experiments of **3a** heated starting from 50 °C to 90 °C, and their corresponding NMR yields, respectively. Yields were calculated according to the integrals of aromatic shifts of nitrile formed ($I_{\text{nitrile, aromatic}} = I_{\text{nt, ar.}}$) and *O*-dimethylcarbamoyloxime consumed ($I_{\text{carbamoyloxime, aromatic}} = I_{\text{cox, ar.}}$) in ^{1}H NMR spectra. Integrals of 2H protons of **4a** were calculated by normalizing the integral of the ^{1}H NMR signal of 1H of **3a** at 8.58 ppm to 1.00.

entry	*T* / °C	*t* / min	$I_{\text{cox, ar. (2H)}}$	$I_{\text{cox, ar. (3H)}}$	$I_{\text{nt, ar. (2H)}}$	$I_{\text{nt, ar. (2H)}}$	NMR yield / %
1	25	0	2.03	3.05	0.00	0.00	0
2	50	20	2.07	3.05	0.00	0.00	0
3	50	40	2.07	3.06	0.00	0.00	0
4	50	60	2.07	3.06	0.00	0.00	0
5	60	20	2.07	3.07	0.01	0.01	0.49
6	60	40	2.07	3.06	0.02	0.02	0.97
7	60	60	2.08	3.06	0.02	0.02	0.97
8	70	0	2.08	3.06	0.03	0.03	1.45
9	70	20	2.09	3.05	0.06	0.05	2.40
10	70	40	2.10	3.05	0.07	0.08	3.79
11	70	60	2.11	3.05	0.09	0.10	4.69
12	80	0	2.13	3.06	0.13	0.13	5.99
13	80	20	2.17	3.06	0.21	0.22	9.73
14	80	40	2.23	3.02	0.30	0.31	13.3
15	80	60	2.27	3.02	0.38	0.41	16.9
16	90	0	2.36	3.03	0.63	0.64	24.1
17	90	20	2.61	3.03	1.10	1.10	35.3
18	90	40	2.88	3.01	1.58	1.62	44.7
19	90	60	3.25	3.03	2.30	2.32	53.5

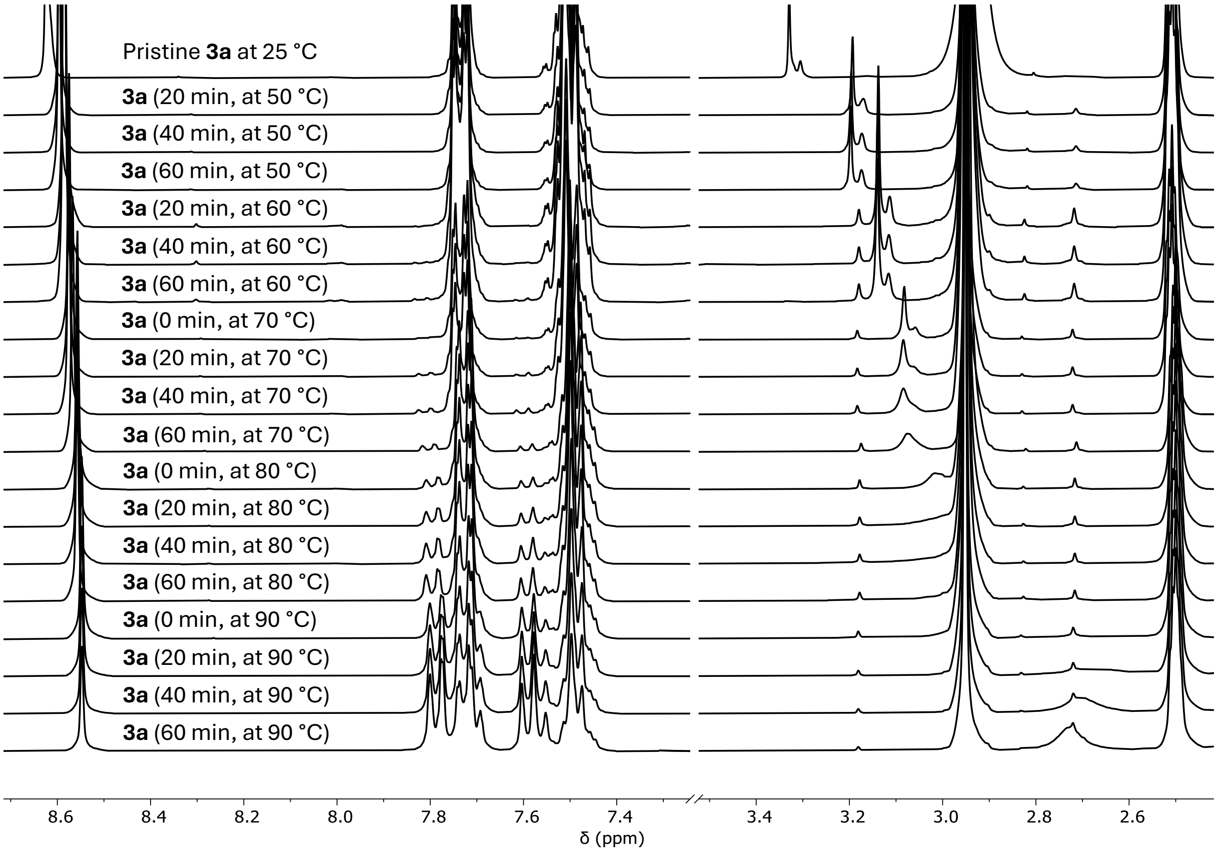

Figure S2. ^{1}H NMR spectra of pristine **3a**, and **3a** heated starting from 50 °C to 90 °C every 20 min recorded by VT NMR.

2.2.3. Thermal exposure time evaluation (benzaldehyde *O*-dimethylcarbamoyloxime **3a** to benzonitrile **4a**)

The heating duration was scanned at 80 °C according to the VT NMR experiment results obtained in the previous Section 2.2.2. **3a** in 4.0 mL of dry DMSO was heated in a Schlenk flask with a concentration of 0.3 M. Every time interval, 0.1 mL of the sample was removed and diluted with 0.5 mL of DMSO-d_6. Samples were removed at 1, 2, 4, 7, 9, 24, and 48 h, and ^{1}H NMR spectra were recorded for both before and after adding 10 µL of DMF.

2.2.4. Solvent evaluation (benzaldehyde *O*-dimethylcarbamoyloxime **3a** to benzonitrile **4a**)

Final heating experiments of **3a** were conducted in 0.6 mL of MeCN-d_3 instead of DMSO-d_6 to evaluate the influence of solvent on the reaction. Two NMR tubes containing **3a** in 0.6 mL of MeCN-d_3 were sealed and put in a thermostat bath maintained at 80 °C. After 6 and 24 h, the tubes were removed, cooled to room temperature, and 10 µL of DMF was added as an internal standard, resulting in an approximate final concentration of 0.1 M. Subsequently, ^{1}H NMR spectra were recorded for both before and after adding 10 µL of DMF.

2.2.5. Substrate scope of diverse *O*-dimethylcarbamoyloximes

Heating experiments were performed with *O*-dimethylcarbamoyloximes **3a-o** in DMSO-d_6, and every thermal treatment was carried out in standard conditions for ^{1}H NMR analysis. Two NMR tubes of each substrate in an approximate final concentration of 0.1 M were charged with 0.5 mL of DMSO-d_6, sealed, and put in a thermostat bath at 90 °C. After 6 and 24 h, the tubes were removed, cooled down, and the ^{1}H NMR spectra were recorded for both in the presence and absence of 8.35 µL of DMF to avoid possible peak overlapping of DMF and diagnostic aromatic peaks of nitriles and/or *O*-dimethylcarbamoyloximes.

^{1}H NMR spectra of pristine samples of **3a-o** were also recorded in 0.5 mL of DMSO-d_6 that was not heated, and directly submitted to ^{1}H NMR to obtain the starting spectrum with and without adding 8.35 µL of DMF, and NMR yields were calculated (Table S4).

At a selected temperature of 90 °C, the decreasing integrals of carbamoyloximes ($I_{cox,t}$) and increasing integrals of nitriles ($I_{nt,t}$) were quantified at t = 6 and 24 h intervals by ^{1}H NMR spectroscopy. For more quantitative analysis, DMF with known concentration as an internal standard was used in the cases where the distinctive peaks do not overlap with the diagnostic peaks of DMF. These values were then used to calculate the NMR yield of newly formed corresponding nitriles as described by the following equation:

$$\text{NMR yield}_t(\%) = \left(\frac{I_{\text{nt},t}}{I_{\text{cox},t} + I_{\text{nt},t}}\right) \cdot 100 \quad (1)$$

Table S4. List of the thermal reactivity experiments of different *O*-dimethylcarbamoyloximes **3a-o** heated at 90 °C for t_1 = 6 h and t_2 = 24 h, and their corresponding NMR yields, respectively. Yields were calculated according to the integrals of nitriles formed $I_{nt,t}$ (more specifically: $I_{\text{nitrile, aromatic, average}} = I_{\text{nt, ar, avg}}$), and *O*-dimethylcarbamoyloximes consumed $I_{cox,t}$ (more specifically: $I_{\text{carbamoyloxime, aromatic, average}} = I_{\text{cox, ar, avg}}$), in ^{1}H NMR spectra if no other further information is given. [a] Integrals of 2H protons of **3a** were calculated by normalizing the ^{1}H NMR signal of DMF at 7.95 ppm. [b] Complete consumption of **3e** was observed; however, the appearance of an additional set of signals was detected, with an integral value of I = 0.04, and the $I_{\text{nt, ar, avg.}}$ was subsequently calculated based on this observation. [c] Average of aromatic protons was denoted for 1H instead of 2H due to non-symmetric aromatic protons. [d] **3i** led to nitrile formation; however, the NMR yield decreased over time due to the occurrence of side reactions. Two additional aromatic signals were detected, with integrals of I = 0.60 and I = 0.30 for 6 h, and I = 1.68 and I = 0.84 for 24 h, respectively, indicating progressive formation of side products. [e] NMR yield of **4m** formed from **3m** was calculated from neighbor α-alkenyl 1H that changes from 7.06 (dd) to 6.48 ppm (d) instead of aromatic 2H owing to overlapping new emerging peak.

entry	compound name	$I_{\text{cox, ar, avg, 6 h (2H)}}$	$I_{\text{nt, ar, avg, 6 h (2H)}}$	$I_{\text{cox, ar, avg, 24 h (2H)}}$	$I_{\text{nt, ar, avg, 24 h (2H)}}$	NMR yield at t_1 / %	NMR yield at t_2 / %	^{1}H NMR
1	**3a**	0.30[a]	0.25[a]	0.04[a]	0.36[a]	45	90	Figure S3
2	**3b**	1.39	2.02	0.06	2.02	59	97	Figure S4
3	**3c**	0.60	2.00	0.02	2.04	77	99	Figure S5
4	**3d**	1.40	2.03	0.07	2.03	59	97	Figure S6
5	**3e**	0.00[b]	2.00	0.00[b]	2.01	96	96	Figure S7
6	**3f**	0.03[c]	1.09[c]	0.01[c]	1.11[c]	97	99	Figure S8
7	**3g**	1.30	2.00	0.06	2.00	61	97	Figure S9
8	**3h**	0.00	1.04[c]	0.00	1.05[c]	100	100	Figure S10
9	**3i**	2.03[d]	3.38[d]	0.00[d]	2.01[d]	54	44	Figure S11
10	**3j**	0.12	1.08	0.00	1.04	90	100	Figure S12
11	**3k**	0.77	2.03	0.03	2.03	73	98	Figure S13
12	**3l**	2.00	12.76	2.00	126.4	52	94	Figure S14
13	**3m**	1.00[e]	0.62[e]	1.03[e]	6.81[e]	38	86	Figure S15
14	**3n**	n.a.	n.a.	n.a.	n.a.	n.a.	n.a.	Figure S116
15	**3o**	n.a.	n.a.	n.a.	n.a.	n.a.	n.a.	Figure S118

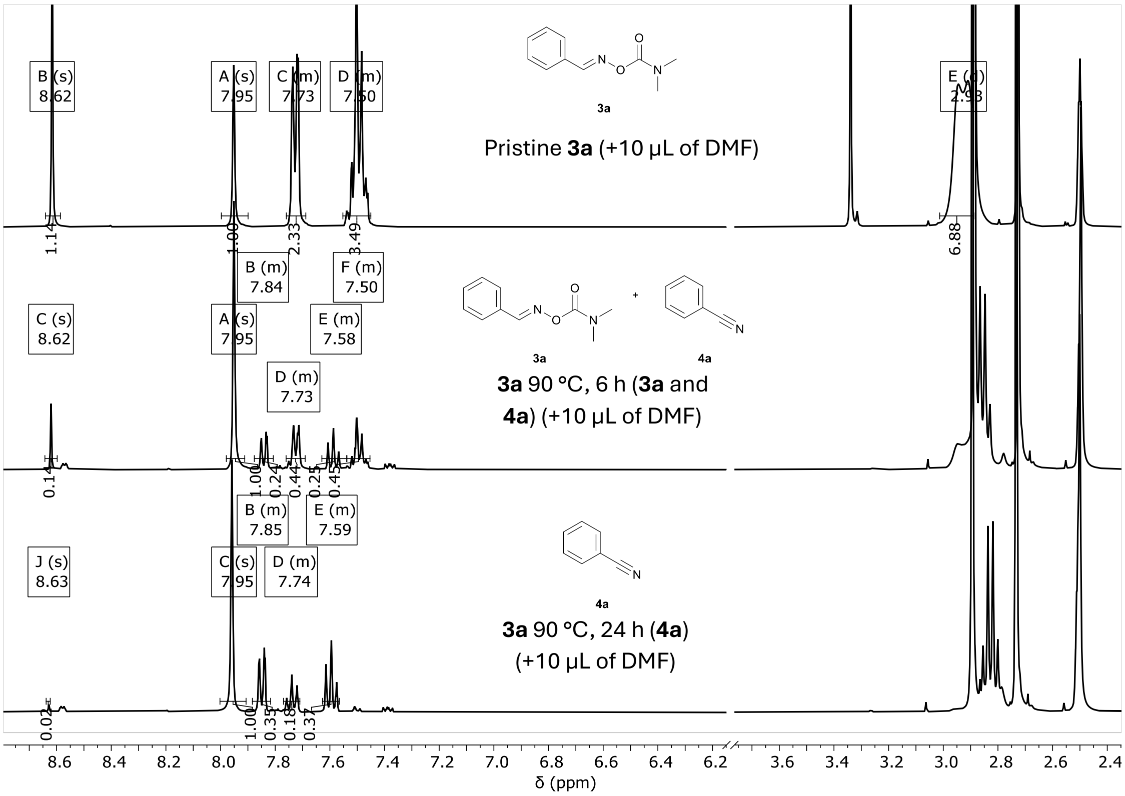

Figure S3. Thermal reactivity of benzaldehyde *O*-dimethylcarbamoyloxime **3a**, **3a** heated for 6 h, and 24 h at 90 °C in DMSO-d_6, respectively (10 µL of DMF is present with the integrated peak (I = 1.0) at 7.95 ppm) (NMRs were cut between 3.7 and 6.2 ppm to visualize the multiplicity and integrals of the aromatic region protons). Reaction DOI: https://dx.doi.org/10.14272/reaction/SA-FUHFF-UHFFFADPSC-JFDZBHWFFU-UHFFFADPSC-NUHFF-NUHFF-NUHFF-ZZZ.

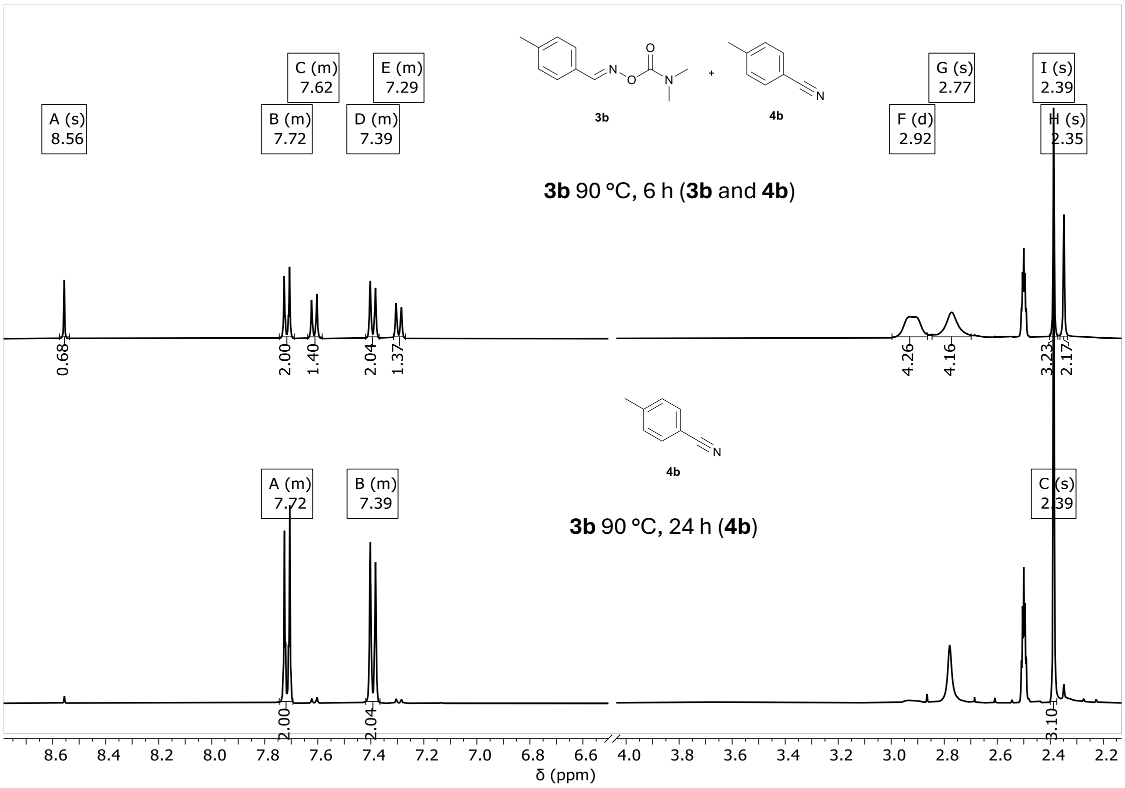

Figure S4. Thermal reactivity of 4-methylbenzaldehyde *O*-dimethylcarbamoyloxime **3b**, **3b** heated for 6 h, and 24 h at 90 °C in DMSO-d_6, respectively (NMRs were cut between 4.0 and 6.5 ppm to visualize the multiplicity and integrals of the aromatic region protons). Reaction DOI: https://dx.doi.org/10.14272/reaction/SA-FUHFF-UHFFFADPSC-VCZNNAKNUV-UHFFFADPSC-NUHFF-NUHFF-NUHFF-ZZZ.

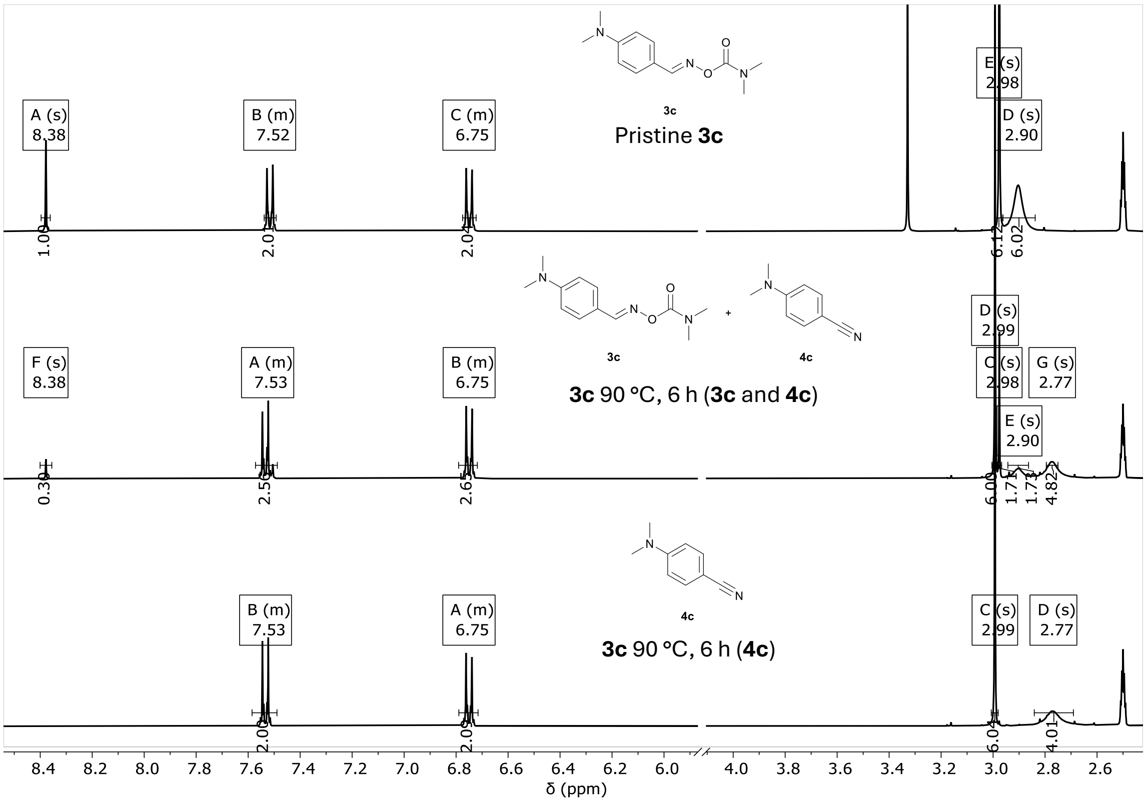

Figure S5. Thermal reactivity of 4-(dimethylamino)benzaldehyde *O*-dimethylcarbamoyloxime **3c**, **3c** heated for 6 h, and 24 h at 90 °C in DMSO-d_6, respectively (NMRs were cut between 4.5 and 5.5 ppm to visualize the multiplicity and integrals of the aromatic region protons). Reaction DOI: https://dx.doi.org/10.14272/reaction/SA-FUHFF-UHFFFADPSC-JYMNQRQQBJ-UHFFFADPSC-NUHFF-NUHFF-NUHFF-ZZZ.

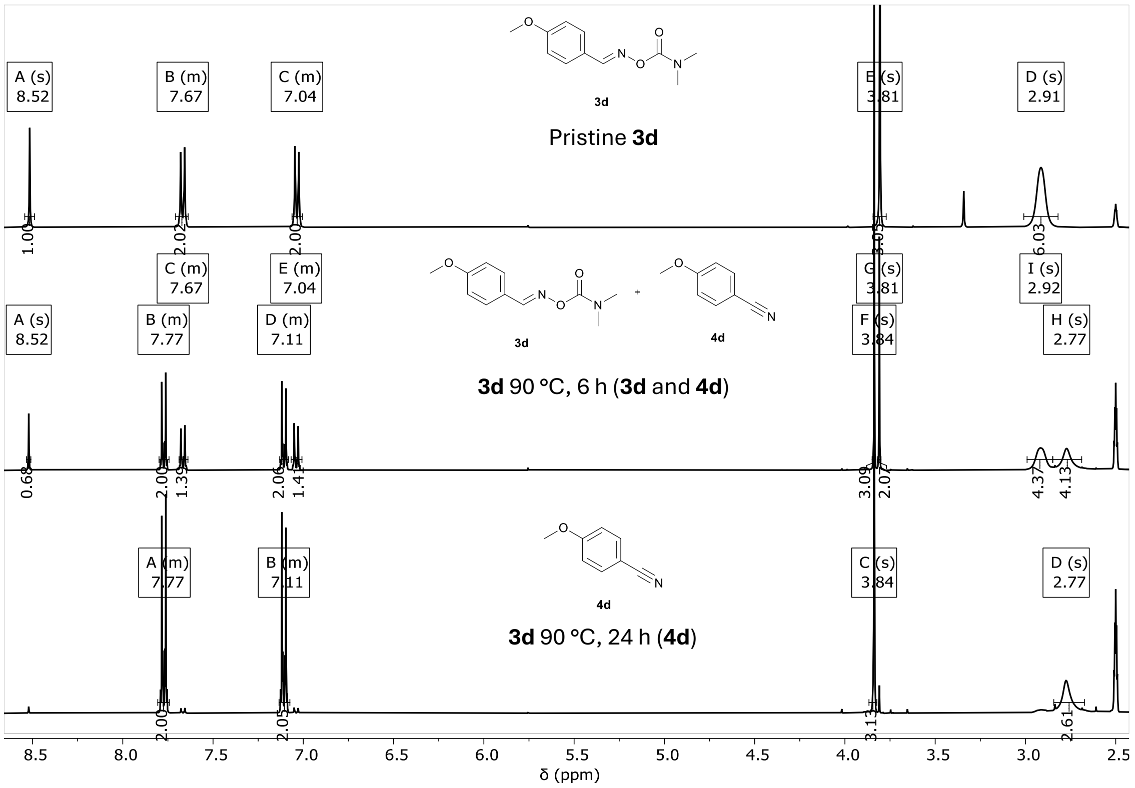

Figure S6. Thermal reactivity of 4-methoxybenzaldehyde *O*-dimethylcarbamoyloxime **3d**, **3d** heated for 6 h, and 24 h at 90 °C in DMSO-d_6, respectively. Reaction DOI: https://dx.doi.org/10.14272/reaction/SA-FUHFF-UHFFFADPSC-XDJAAZYHCC-UHFFFADPSC-NUHFF-NUHFF-NUHFF-ZZZ.3.

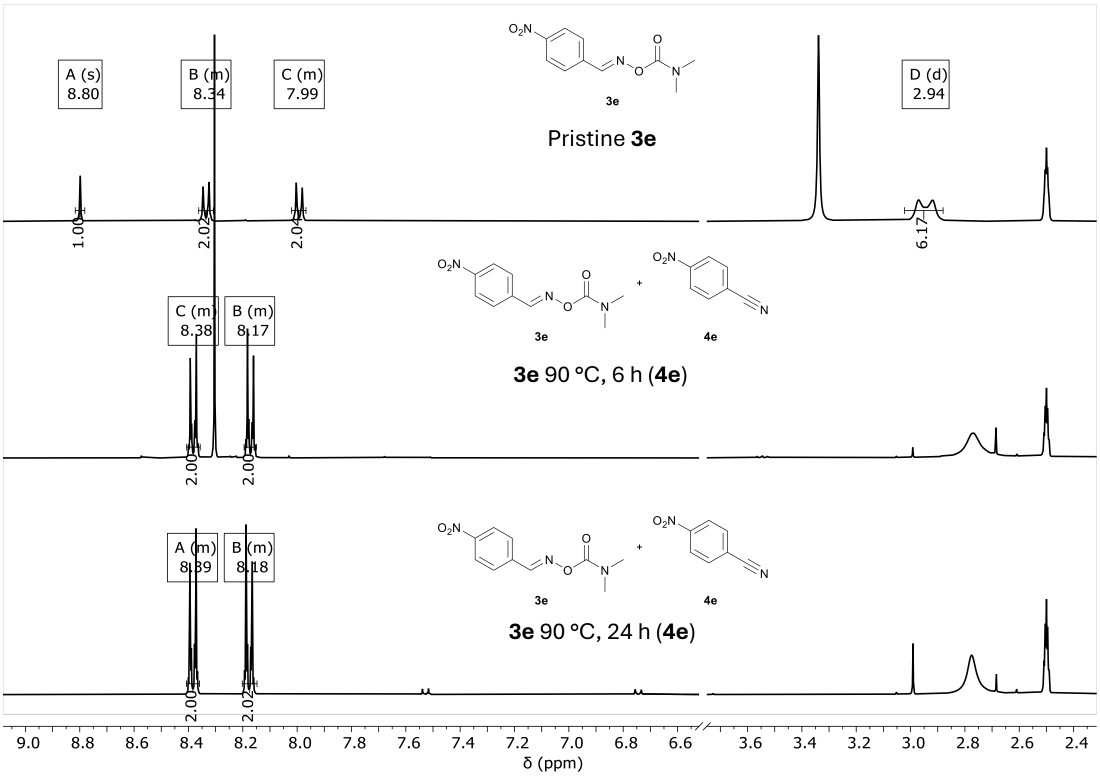

Figure S7. Thermal reactivity of 4-nitrobenzaldehyde *O*-dimethylcarbamoyloxime **3e**, **3e** heated for 6 h, and 24 h at 90 °C in DMSO-d_6, respectively (NMRs were cut between 3.7 and 6.5 ppm to visualize the multiplicity and integrals of the aromatic region protons). Reaction DOI: https://dx.doi.org/10.14272/reaction/SA-FUHFF-UHFFFADPSC-NKJIFDNZPG-UHFFFADPSC-NUHFF-NUHFF-NUHFF-ZZZ.

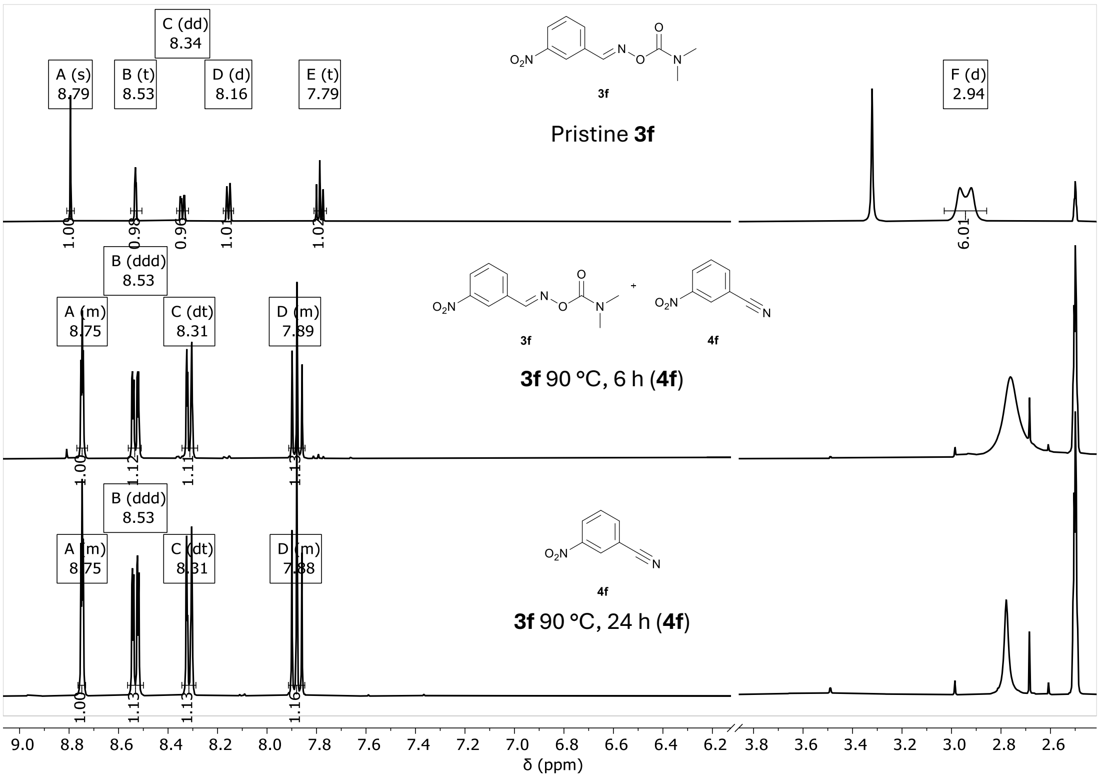

Figure S8. Thermal reactivity of 3-nitrobenzaldehyde *O*-dimethylcarbamoyloxime **3f**, **3f** heated for 6 h, and 24 h at 90 °C in DMSO-d_6, respectively (NMRs were cut between 3.8 and 6.2 ppm to visualize the multiplicity and integrals of the aromatic region protons). Reaction DOI: https://dx.doi.org/10.14272/reaction/SA-FUHFF-UHFFFADPSC-RUSAWEHOGC-UHFFFADPSC-NUHFF-NUHFF-NUHFF-ZZZ.

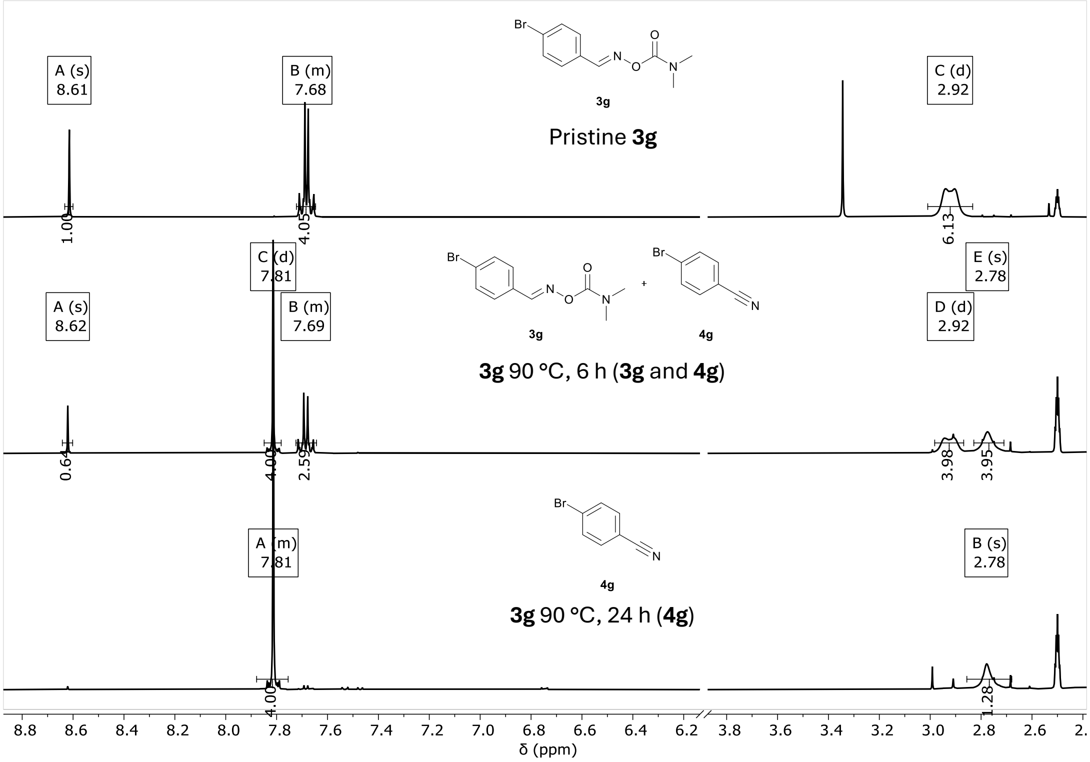

Figure S9. Thermal reactivity of 4-bromobenzaldehyde *O*-dimethylcarbamoyloxime **3g**, **3g** heated for 6 h, and 24 h at 90 °C in DMSO-d_6, respectively (NMRs were cut between 3.8 and 6.2 ppm to visualize the multiplicity and integrals of the aromatic region protons). Reaction DOI: https://dx.doi.org/10.14272/reaction/SA-FUHFF-UHFFFADPSC-HQSCPPCMBM-UHFFFADPSC-NUHFF-NUHFF-NUHFF-ZZZ.

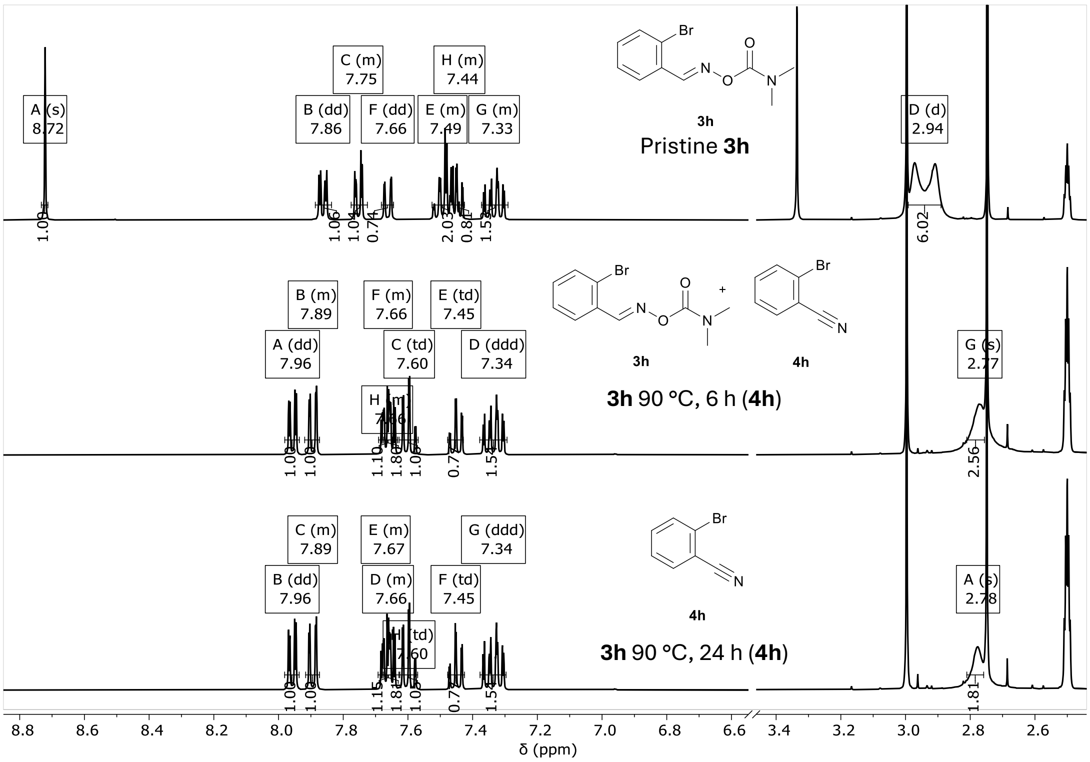

Figure S10. Thermal reactivity of 2-bromobenzaldehyde *O*-dimethylcarbamoyloxime **3h**, **3h** heated for 6 h, and 24 h at 90 °C in DMSO-d_6, respectively (NMRs were cut between 3.5 and 6.5 ppm to visualize the multiplicity and integrals of the aromatic region protons). Spectrum contains the signals that are present in the ^{1}H NMR of the pristine sample (7.66 (overlapping with the newly formed signal), 7.46–7.42, 7.38–7.29, 3.00, and 2.75 ppm) since the sample was used with present impurities. Reaction DOI: https://dx.doi.org/10.14272/reaction/SA-FUHFF-UHFFFADPSC-AFMPMSCZPV-UHFFFADPSC-NUHFF-NUHFF-NUHFF-ZZZ.

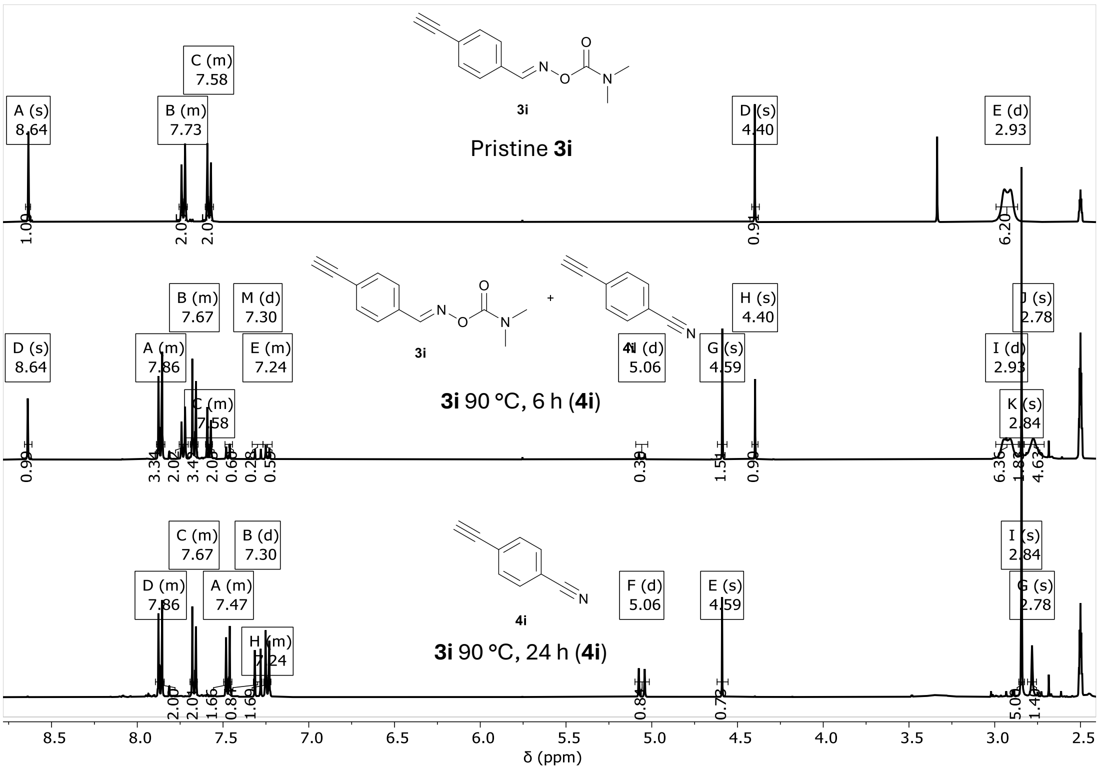

Figure S11. Thermal reactivity of 4-ethynylbenzaldehyde *O*-dimethylcarbamoyloxime **3i**, **3i** heated for 6 h, and 24 h at 90 °C in DMSO-d_6, respectively. Reaction DOI: https://dx.doi.org/10.14272/reaction/SA-FUHFF-UHFFFADPSC-LAGNMUUUMQ-UHFFFADPSC-NUHFF-NUHFF-NUHFF-ZZZ.

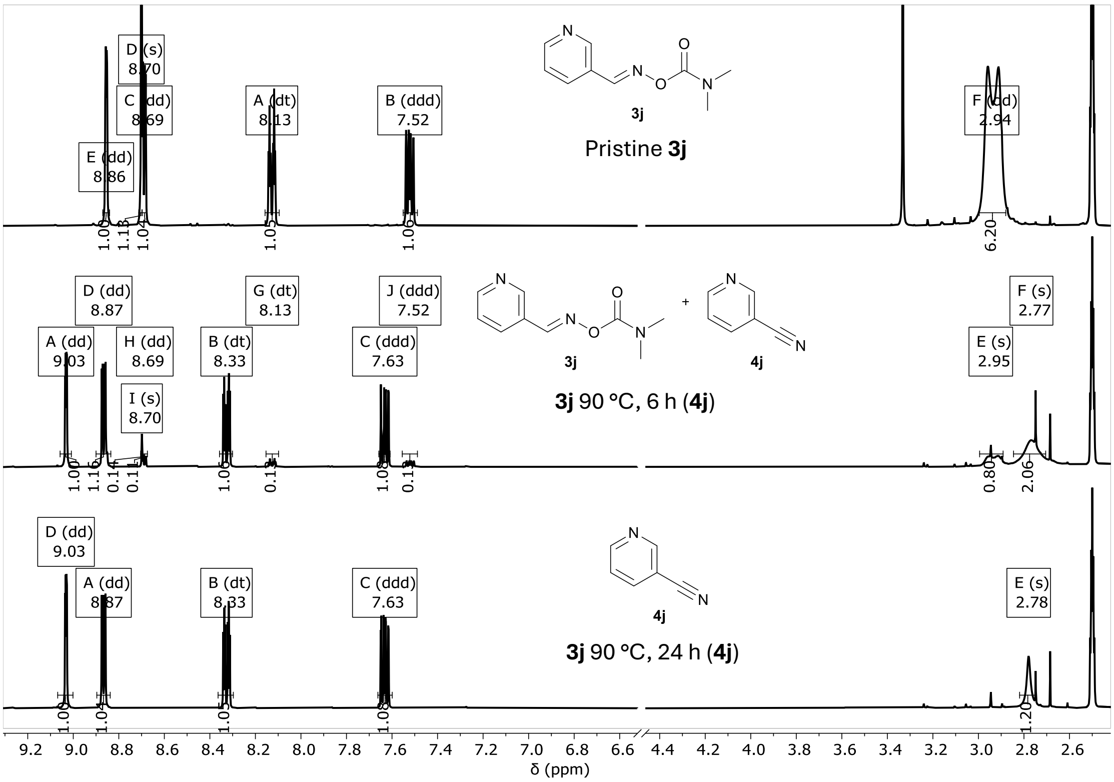

Figure S12. Thermal reactivity of nicotinaldehyde *O*-dimethylcarbamoyloxime **3j**, **3j** heated for 6 h, and 24 h at 90 °C in DMSO-d_6, respectively (NMRs were cut between 4.5 and 6.5 ppm to visualize the multiplicity and integrals of the aromatic region protons). Reaction DOI: https://dx.doi.org/10.14272/reaction/SA-FUHFF-UHFFFADPSC-GZPHSAQLYP-UHFFFADPSC-NUHFF-NUHFF-NUHFF-ZZZ.

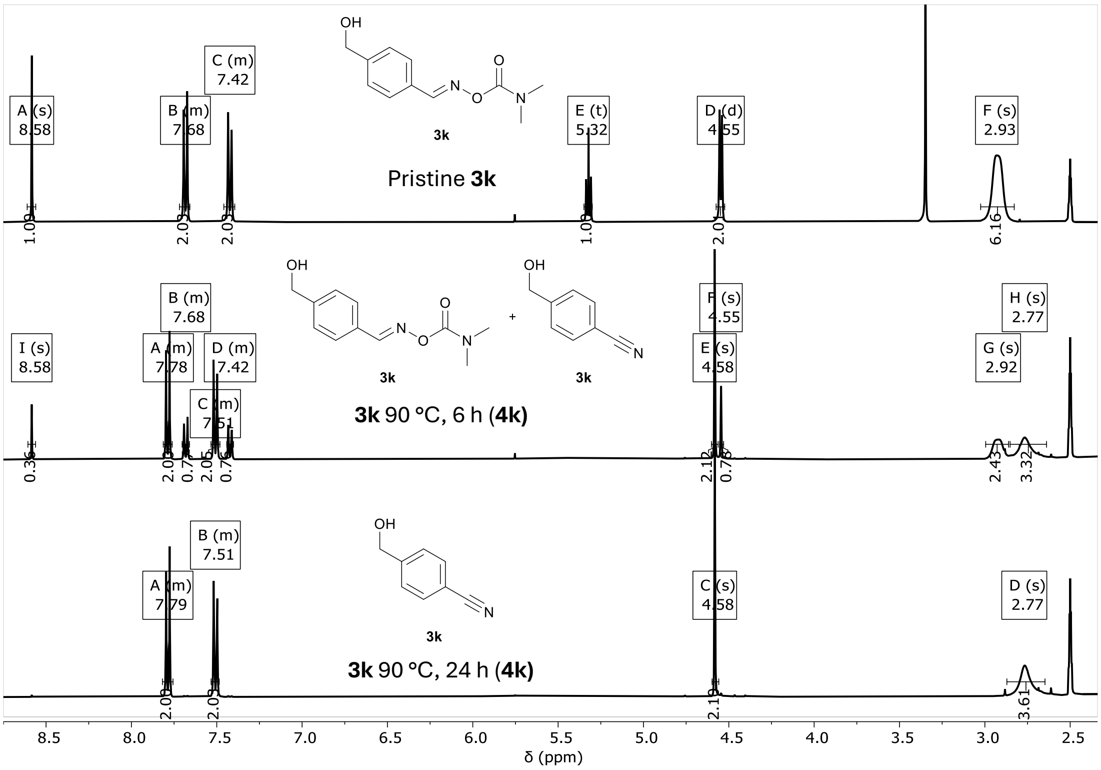

Figure S13. Thermal reactivity of 4-(hydroxymethyl)benzaldehyde *O*-dimethylcarbamoyloxime **3k**, **3k** heated for 6 h, and 24 h at 90 °C in DMSO-d_6, respectively. Reaction DOI: https://dx.doi.org/10.14272/reaction/SA-FUHFF-UHFFFADPSC-XAASLEJRGF-UHFFFADPSC-NUHFF-NUHFF-NUHFF-ZZZ.

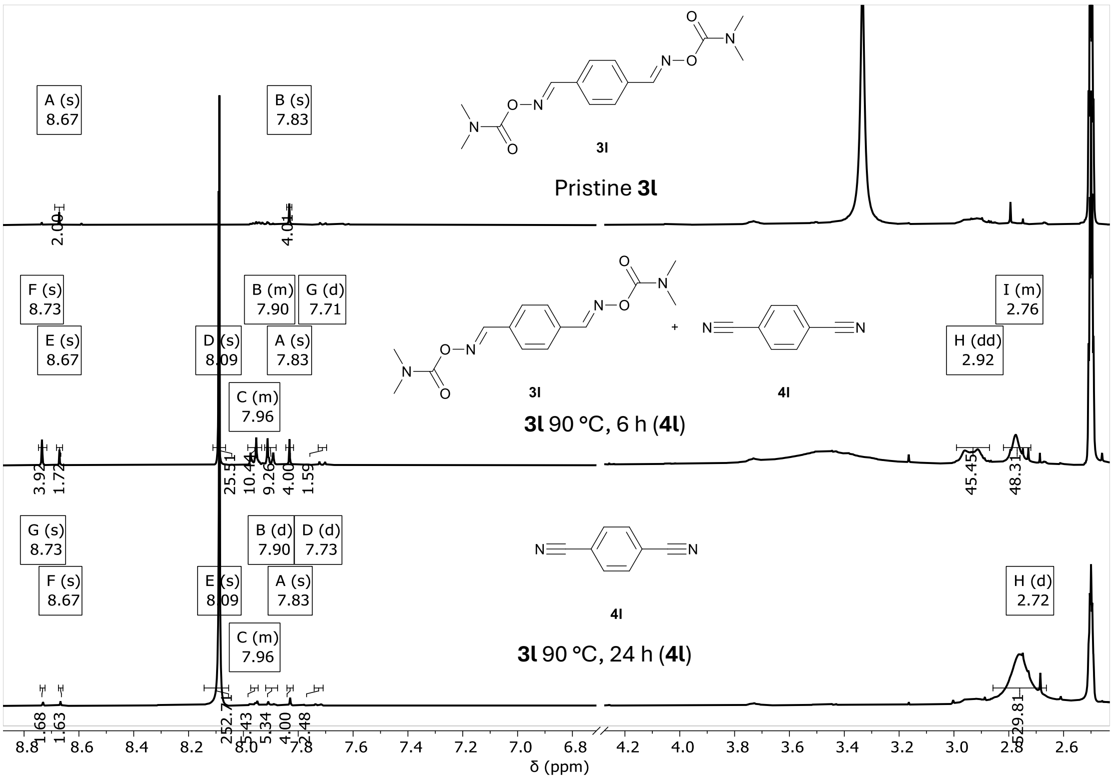

Figure S14. Thermal reactivity of terephthalaldehyde *O,O*-didimethylcarbamoyl dioxime **3l**, **3l** heated for 6 h, and 24 h at 90 °C in DMSO-d_6, respectively (NMRs were cut between 4.2 and 6.7 ppm to visualize the multiplicity and integrals of the aromatic region protons. Additionally, the solubility of **3l** was low in DMSO-d_6, and it increased by heating; therefore, it led to poor resolution for the NMR of pristine **3l**). Reaction DOI: https://dx.doi.org/10.14272/reaction/SA-FUHFF-UHFFFADPSC-BHXFKXOIOD-UHFFFADPSC-NUHFF-NUHFF-NUHFF-ZZZ.

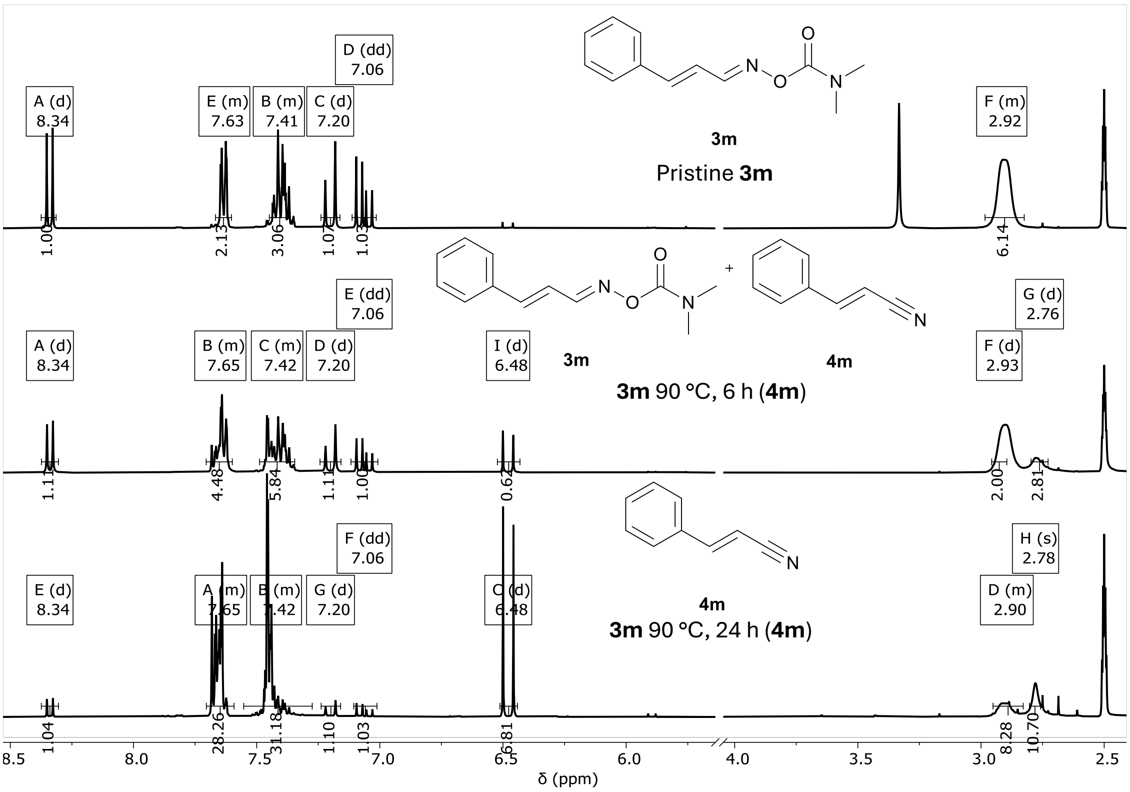

Figure S15. Thermal reactivity of cinnamaldehyde *O*-dimethylcarbamoyloxime **3m**, **3m** heated for 6 h, and 24 h at 90 °C in DMSO-d_6, respectively (NMRs were cut between 4.1 and 5.3 ppm to visualize the multiplicity and integrals of the aromatic region protons). Reaction DOI: https://dx.doi.org/10.14272/reaction/SA-FUHFF-UHFFFADPSC-ZWKNLRXFUT-UHFFFADPSC-NUHFF-NNUHD-NUHFF-ZZZ.

2.3. Semi-one-pot and one-pot transformation of aldehydes to nitriles

Starting material was switched from **1a** to **1d** (or **2d** for semi-one-pot) due to the relative volatility of benzonitrile **4a** in comparison to **4d** for semi-one-pot, one-pot, and microwave-assisted one-pot transformations.

2.3.1. Semi-one-pot synthesis of nitriles (4-methoxybenzaldehyde oxime **2d** to 4-methoxybenzonitrile **4d**)

4-Methoxybenzaldehyde oxime **2d** (121 mg, 800 µmol, 1.00 equiv.), dry pyridine (95.0 mg, 96.7 µL, 1.20 mmol, 1.50 equiv.), and dry Et_3N (121 mg, 167 µL, 1.20 mmol, 1.50 equiv.) were dissolved in 7.0 mL of dry MeCN in a Schlenk flask under an Ar atmosphere. Subsequently, *N,N*-dimethylcarbamoyl chloride (129 mg, 110 µL, 1.20 mmol, 1.50 equiv.) was injected, and the mixture was stirred for 18 h to obtain 4-methoxybenzaldehyde *O*-dimethylcarbamoyloxime **3d**. The reaction flask was directly immersed in an oil bath at 80 °C after full conversion to **3d** was confirmed by TLC. 2.0 mL of the reaction mixture was removed after 6 and 24 h for reaction control, and all volatiles were removed *in vacuo* separately. The oily residue was dissolved in 5 mL of CH_2Cl_2, washed with water (2×5 mL), and brine (1×5 mL). The organic layer was dried over $MgSO_4$, filtered, and concentrated *in vacuo* to afford an off-white solid **4d** after 24 h (102 mg, 766 µmol, 97% yield). **^{1}H NMR (400 MHz, $CDCl_3$):** δ_H / ppm = 7.60–7.52 (m, 2H), 6.96–6.89 (m, 2H), 3.83 (s, 3H). See Figure S16. **^{13}C NMR (101 MHz, $CDCl_3$):** δ_C / ppm = 162.9, 134.0, 119.3, 114.8, 104.0, 55.6. See Figure S17. **APCI$^+$ HRMS**: *m/z* = 134.0603 (calcd. 134.0600 for MH^+) (*m/z* only reported for 24 h). See Figure S18. Further details, data, and metadata can be found under reaction DOIs: https://dx.doi.org/10.14272/reaction/SA-FUHFF-UHFFFADPSC-SIJYEXCZGU-UHFFFADPSC-NUHFF-NXJJT-NUHFF-ZZZ.3, and https://dx.doi.org/10.14272/reaction/SA-FUHFF-UHFFFADPSC-XDJAAZYHCC-UHFFFADPSC-NUHFF-NUHFF-NUHFF-ZZZ.2.

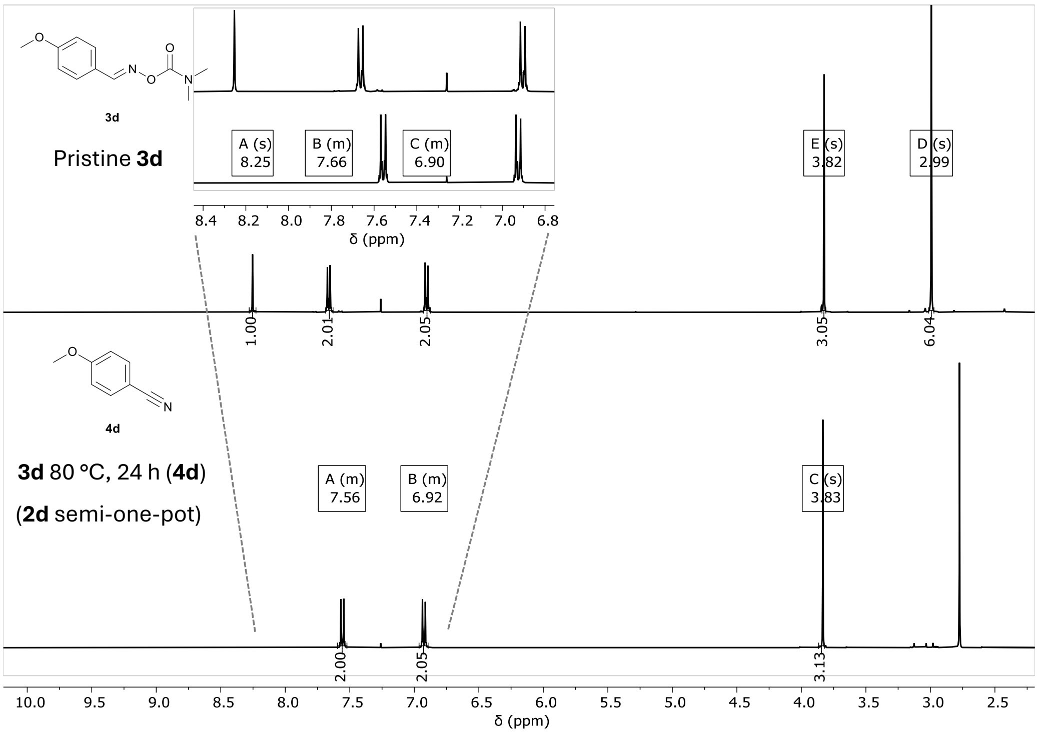

Figure S16. Comparison of ^{1}H NMR spectra of previously synthesized reference 4-methoxybenzaldehyde *O*-dimethylcarbamoyloxime **3d** (top) and 4-methoxybenzonitrile **4d**, obtained by semi-one-pot synthesis from 4-methoxybenzaldehyde oxime **2d** by heating **3d** at 80 °C for 24 h (bottom) (in $CDCl_3$). Analysis DOI: https://dx.doi.org/10.14272/XDJAAZYHCCRJOK-UHFFFAOYSA-N/CHMO0000593.

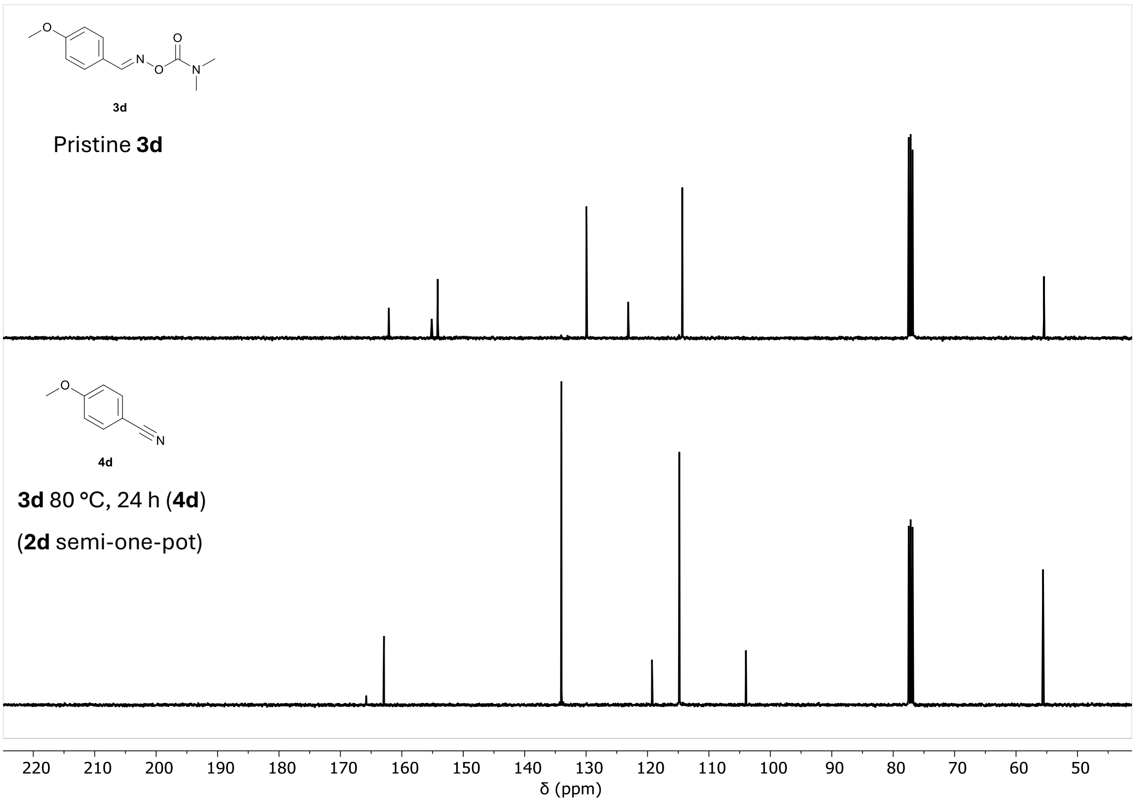

Figure S17. Comparison of ^{13}C NMR spectra of previously synthesized reference 4-methoxybenzaldehyde *O*-dimethylcarbamoyloxime **3d** (top) and 4-methoxybenzonitrile **4d**, obtained by semi-one-pot synthesis from 4-methoxybenzaldehyde oxime **2d** by heating **3d** at 80 °C for 24 h (bottom) (in $CDCl_3$). Analysis DOI: https://dx.doi.org/10.14272/XDJAAZYHCCRJOK-UHFFFAOYSA-N/CHMO0000595.

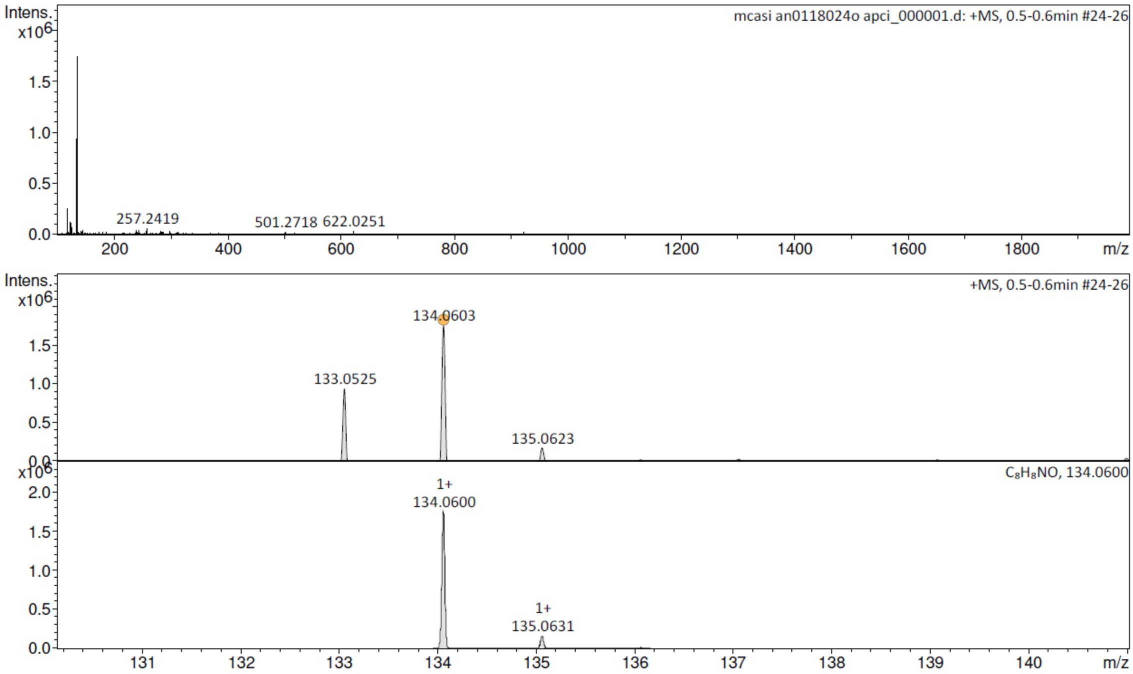

Figure S18. Deconvoluted APCI$^+$ HRMS spectrum (top), along with the comparison between the deconvoluted APCI$^+$ HRMS (top) and the calculated mass spectrum (bottom) of 4-methoxybenzonitrile **4d**, obtained by semi-one-pot synthesis from 4-methoxybenzaldehyde oxime **2d** by heating **3d** at 80 °C for 24 h. Analysis DOI: https://dx.doi.org/10.14272/XDJAAZYHCCRJOK-UHFFFAOYSA-N/CHMO0002763.

2.3.2. One-pot synthesis of nitriles (4-methoxybenzaldehyde **1d** to 4-methoxybenzonitrile **4d**)

4-Methoxybenzaldehyde (204 mg, 1.50 mmol, 1.00 equiv.), hydroxylamine hydrochloride (115 mg, 68.9 µL, 1.65 mmol, 1.10 equiv.), and pyridine (491 mg, 500 µL, 6.21 mmol, 4.14 equiv.) were dissolved in 7.0 mL of MeCN in a Schlenk flask and refluxed for 24 h. 4-Methoxybenzaldehyde oxime **2d** was obtained without further purification. Subsequently, the same reaction medium was treated with the next reaction precursors: pyridine (178 mg, 182 µL, 2.25 mmol, 1.50 equiv.), Et_3N (228 mg, 314 µL, 2.25 mmol, 1.50 equiv.), and *N,N*-dimethylcarbamoyl chloride (242 mg, 207 µL, 2.25 mmol, 1.50 equiv.), and stirred overnight at r.t. to obtain 4-methoxybenzaldehyde *O*-dimethylcarbamoyloxime **3d**. The next day, the reaction flask was directly immersed in an oil bath at 80 °C after full conversion to **3d** was confirmed by TLC. 2.0 mL of the reaction mixtures were removed after 6 and 24 h, separately for the reaction control, and all volatiles were removed *in vacuo*. For both samples removed at 6 and 24 h, the oily residues were dissolved in 5 mL CH_2Cl_2, washed with H_2O (2×5 mL), and brine (1×5 mL). The organic layers were dried over $MgSO_4$, filtered, and concentrated *in vacuo* to afford off-white solid 4-methoxybenzonitrile **4d** after 24 h (140 mg, 1.05 mmol, 70% NMR yield). **^{1}H NMR (400 MHz, $CDCl_3$):** δ_H / ppm = 7.60–7.52 (m, 2H), 6.96–6.89 (m, 2H), 3.83 (s, 3H). See Figure S19. **^{13}C NMR (101 MHz, $CDCl_3$):** δ_C / ppm = 162.9, 134.0, 119.3, 114.8, 104.0, 55.6. See Figure S20. **APCI$^+$ HRMS**: *m/z* = 134.0599 (calcd. 134.0600 for MH^+) (*m/z* only reported for 24 h). See Figure S21. Details of the experiment, molecular characterization data, and metadata can be found under reaction DOIs: https://dx.doi.org/10.14272/reaction/SA-FUHFF-UHFFFADPSC-FXOSHPAYNZ-UHFFFADPSC-NUHFF-NHSZW-NUHFF-ZZZ.1, https://dx.doi.org/10.14272/reaction/SA-FUHFF-UHFFFADPSC-SIJYEXCZGU-UHFFFADPSC-NUHFF-NXJJT-NUHFF-ZZZ.1, and https://dx.doi.org/10.14272/reaction/SA-FUHFF-UHFFFADPSC-XDJAAZYHCC-UHFFFADPSC-NUHFF-NUHFF-NUHFF-ZZZ.

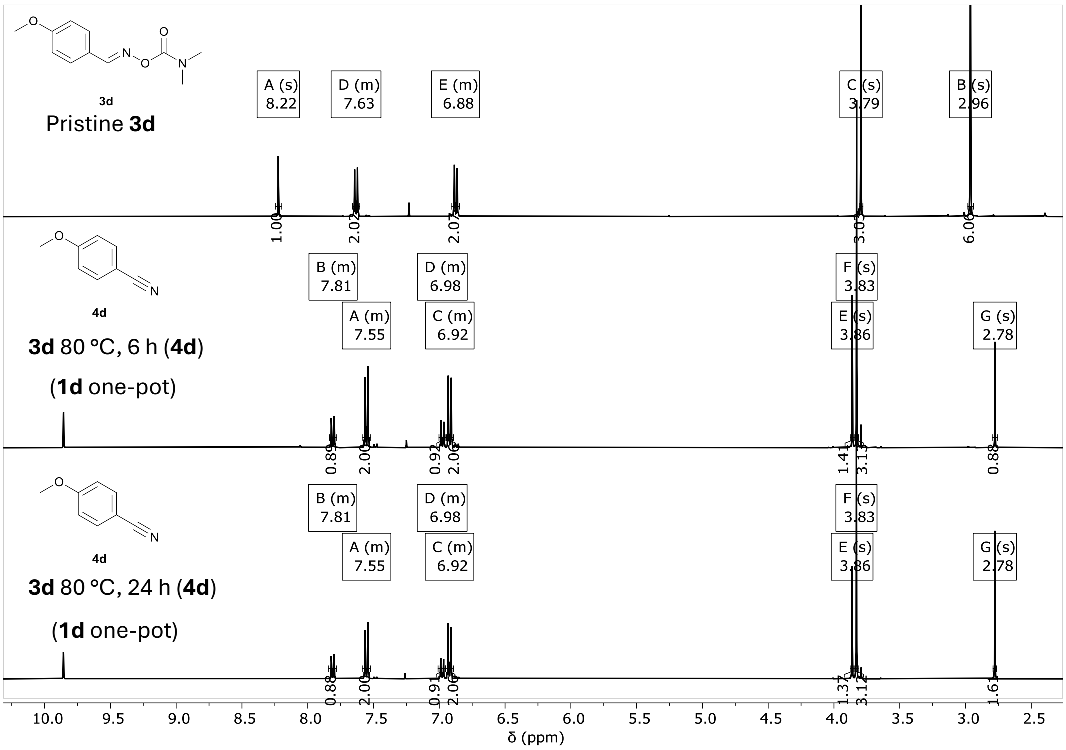

Figure S19. Comparison of ^{1}H NMR spectra of previously synthesized reference 4-methoxybenzaldehyde *O*-dimethylcarbamoyloxime **3d** (top) and 4-methoxybenzonitrile **4d**, obtained by one-pot synthesis from 4-methoxybenzaldehyde **1d** by heating 4-methoxybenzaldehyde *O*-dimethylcarbamoyloxime **3d** at 80 °C for 6 h (middle) and 24 h (bottom) (in $CDCl_3$) (Middle and bottom ^{1}H NMRs consist of 4-methoxybenzaldehyde that remains unreacted further).

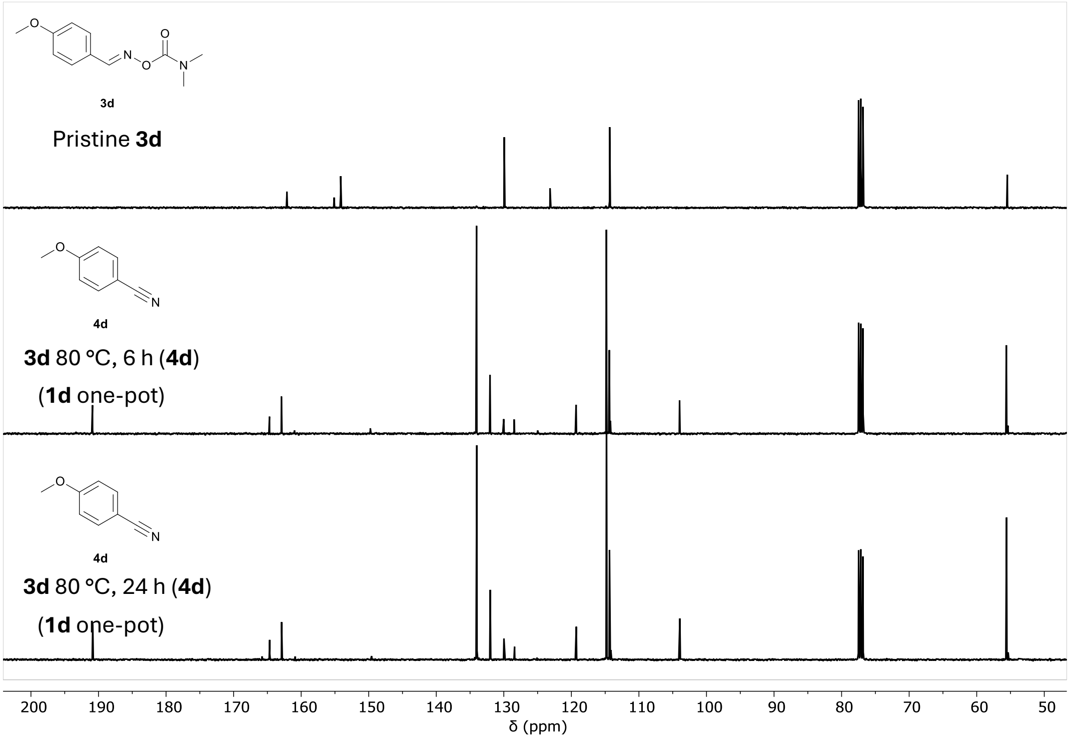

Figure S20. Comparison of ^{13}C NMR spectra of previously synthesized reference 4-methoxybenzaldehyde *O*-dimethylcarbamoyloxime **3d** (top), 4-methoxybenzonitrile **4d**, obtained by one-pot synthesis from 4-methoxybenzaldehyde **1d** by heating 4-methoxybenzaldehyde *O*-dimethylcarbamoyloxime **3d** at 80 °C for 6 h (middle) and 24 h (bottom) (in $CDCl_3$) (Middle and bottom ^{13}C NMRs consist of 4-methoxybenzaldehyde that remains unreacted further).

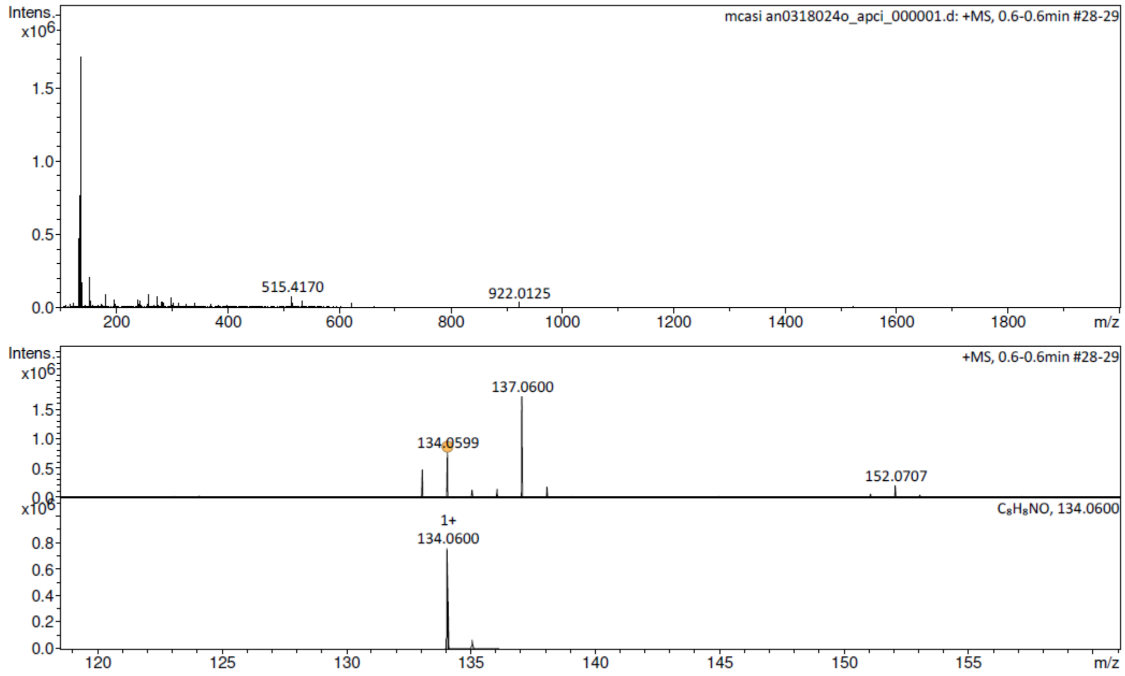

Figure S21. Deconvoluted APCI$^+$ HRMS spectrum (top), along with the comparison between the deconvoluted APCI$^+$ HRMS (top) and the calculated mass spectrum (bottom) of 4-methoxybenzonitrile **4d**, obtained by one-pot synthesis from 4-methoxybenzaldehyde **1d** by heating 4-methoxybenzaldehyde *O*-dimethylcarbamoyloxime **3d** at 80 °C for 24 h. Analysis DOI: https://dx.doi.org/10.14272/SA-FUHFF-UHFFFADPSC-XDJAAZYHCC-UHFFFADPSC-NUHFF-NUHFF-NUHFF-ZZZ/CHMO0002763.

2.4. One-pot formation of nitriles *via* microwave-assisted synthesis

4-Methoxybenzaldehyde **1d** (102 mg, 91.1 µL, 750 µmol, 1.00 equiv.), hydroxylamine hydrochloride (104 mg, 62.3 µL, 1.50 mmol, 2.00 equiv.), and pyridine (178 mg, 181 µL, 2.25 mmol, 3.00 equiv.) were dissolved in 3.5 mL MeCN in a microwave vial, pre-stirred for 30 s in the microwave synthesizer, and microwave-heated by using 150 W of continuous microwave power for 30 min at 80 °C. 4-methoxybenzaldehyde oxime **2d** was obtained without further purification, and full conversion was confirmed by TLC. Subsequently, the same reaction vial was treated with Et_3N (228 mg, 314 µL, 2.25 mmol, 3.00 equiv.) and *N,N*-dimethylcarbamoyl chloride (215 mg, 184 µL, 2.00 mmol, 2.67 equiv.), pre-stirred for 30 s in the microwave synthesizer, and stirred for 80 min at 40 °C to obtain 4-methoxybenzaldehyde *O*-dimethylcarbamoyloxime **3d** (since the lowest temperature provided by the microwave synthesizer was 40 °C, the temperature was set to this degree instead of r.t.). The microwave vial was directly subjected to microwave heating at 80 °C for 60 min after full conversion to **3d** was confirmed by TLC. Later, all volatiles were removed *in vacuo*. The oily residue was dissolved in 5 mL CH_2Cl_2, washed with H_2O (2×5 mL), and brine (1×5 mL). The organic layers were dried over $MgSO_4$, filtered, and concentrated *in vacuo* to afford off-white solid 4-methoxybenzonitrile **4d** (180 mg, 1.35 mmol, 90% yield). **^{1}H NMR (400 MHz, DMSO-*d*$_6$):** δ_H / ppm = 7.60–7.52 (m, 2H), 6.96–6.89 (m, 2H), 3.83 (s, 3H). See Figure S22. **^{13}C NMR (101 MHz, DMSO-*d*$_6$):** δ_C / ppm = 162.7, 134.2, 119.1, 115.1, 102.8, 55.7. See Figure S23. Details of the experiment, molecular characterization data, and metadata can be found under reaction DOIs: https://dx.doi.org/10.14272/reaction/SA-FUHFF-UHFFFADPSC-FXOSHPAYNZ-UHFFFADPSC-NUHFF-NHSZW-NUHFF-ZZZ.2, https://dx.doi.org/10.14272/reaction/SA-FUHFF-UHFFFADPSC-SIJYEXCZGU-UHFFFADPSC-NUHFF-NXJJT-NUHFF-ZZZ.2, and https://dx.doi.org/10.14272/reaction/SA-FUHFF-UHFFFADPSC-XDJAAZYHCC-UHFFFADPSC-NUHFF-NUHFF-NUHFF-ZZZ.1.

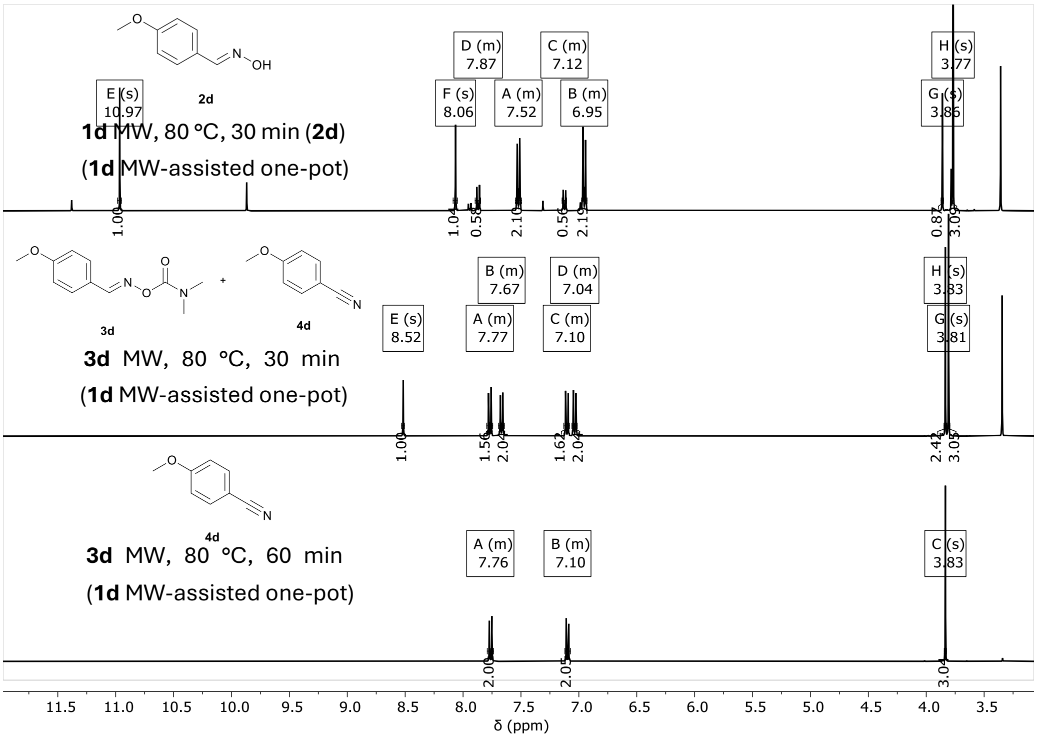

Figure S22. Comparison of ^{1}H NMR spectra of 4-methoxybenzaldehyde oxime **2d** (top) formed from 4-methoxybenzaldehyde **1d** after microwave irradiation at 80 °C for 30 min in MeCN, partial conversion to 4-methoxybenzonitrile **4d** formed by microwave-assisted heating at 80 °C for 30 min in MeCN (middle), and complete conversion after 60 min under identical conditions (bottom) (in DMSO-d_6). ^{1}H NMRs contain residual 4-methoxybenzaldeyde **1d** (top), and also incomplete formation of **4d**, consistent with intermediate **3d** formation (middle). The middle spectrum was reported to show the formation of **3d**.

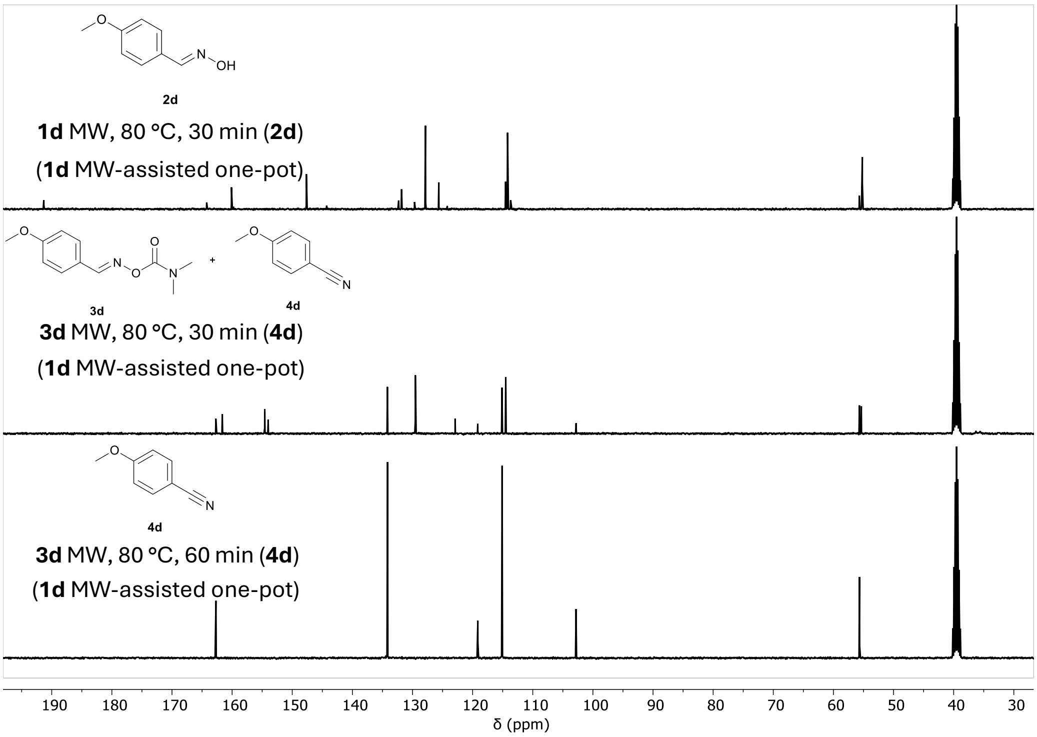

Figure S23. Comparison of ^{13}C NMR spectra of 4-methoxybenzaldehyde oxime **2d** (top) formed from 4-methoxybenzaldehyde **1d** after microwave irradiation at 80 °C for 30 min in MeCN, partial conversion to 4-methoxybenzonitrile **4d** formed by microwave-assisted heating at 80 °C for 30 min in MeCN (middle), and complete conversion after 60 min under identical conditions (bottom) (in DMSO-d_6). ^{1}H NMRs contain residual 4-methoxybenzaldehyde **1d** (top), and also incomplete formation of **4d**, consistent with intermediate **3d** formation (middle). The middle spectrum was reported to show the formation of **3d**.

2.5. Large-scale one-pot formation of nitriles (4-methoxybenzaldehyde **1d** to 4-methoxybenzonitrile **4d**)

4-Methoxybenzaldehyde (10.0 g, 73.4 mmol, 1.00 equiv.), $NH_3OH^+Cl^-$ (7.66 g, 4.59 mL, 110 mmol, 1.50 equiv.), and pyridine (17.4 g, 17.8 mL, 220 mmol, 3.00 equiv.) were dissolved in 300.0 mL of MeCN in a Schlenk flask and refluxed for 24 h. 4-Methoxybenzaldehyde oxime **2d** was obtained without further purification and was confirmed by TLC. Subsequently, the same reaction flask containing crude 4-methoxybenzaldehyde oxime (11.0 g, 72.8 mmol, 1.00 equiv.) was diluted to 450.0 mL of MeCN due to the high amount of $Et_3N \cdot HCl$ formed, and treated with the next reaction precursors; dry pyridine (24.2 g, 24.6 mL, 306 mmol, 4.20 equiv.) and dry Et_3N (30.9 g, 42.6 mL, 306 mmol, 4.20 equiv.). Subsequently, *N,N*-dimethylcarbamoyl chloride (29.0 g, 24.8 mL, 269 mmol, 3.70 equiv.) was injected and stirred overnight at r.t. to obtain 4-methoxybenzaldehyde *O*-dimethylcarbamoyloxime **3d**. Then, the same reaction flask containing crude **3d** (16.2 g, 72.8 mmol, 1.00 equiv.) was immersed in an oil bath at 80 °C after full conversion to **3d** was confirmed by TLC. After completion in 24 h, all volatiles were removed *in vacuo*. The oily residue was dissolved in CH_2Cl_2, washed with H_2O (3×), and brine (1×). The organic layers were combined, dried over $MgSO_4$, filtered, and concentrated *in vacuo* to afford an off-white solid of **4d** (9.69 g, 98% purity, 71.3 mmol, 98% yield). **^{1}H NMR (400 MHz, $CDCl_3$):** δ_H / ppm = 7.60–7.52 (m, 2H), 6.96–6.89 (m, 2H), 3.83 (s, 3H). See Figure S24. **^{13}C NMR (101 MHz, $CDCl_3$):** δ_C / ppm = 162.9, 134.0, 119.3, 114.8, 104.0, 55.6. See Figure S25. **APCI$^+$ HRMS**: *m/z* = 134.0603 (calcd. 134.0600 for MH^+) (*m/z* only reported for 24 h). See Figure S26. Details of the experiment, molecular characterization data, and metadata can be found under reaction DOIs: https://dx.doi.org/10.14272/reaction/SA-FUHFF-UHFFFADPSC-FXOSHPAYNZ-UHFFFADPSC-NUHFF-NHSZW-NUHFF-ZZZ.3, https://dx.doi.org/10.14272/reaction/SA-FUHFF-UHFFFADPSC-SIJYEXCZGU-UHFFFADPSC-NUHFF-NXJJT-NUHFF-ZZZ.4, and https://dx.doi.org/10.14272/reaction/SA-FUHFF-UHFFFADPSC-XDJAAZYHCC-UHFFFADPSC-NUHFF-NUHFF-NUHFF-ZZZ.5.

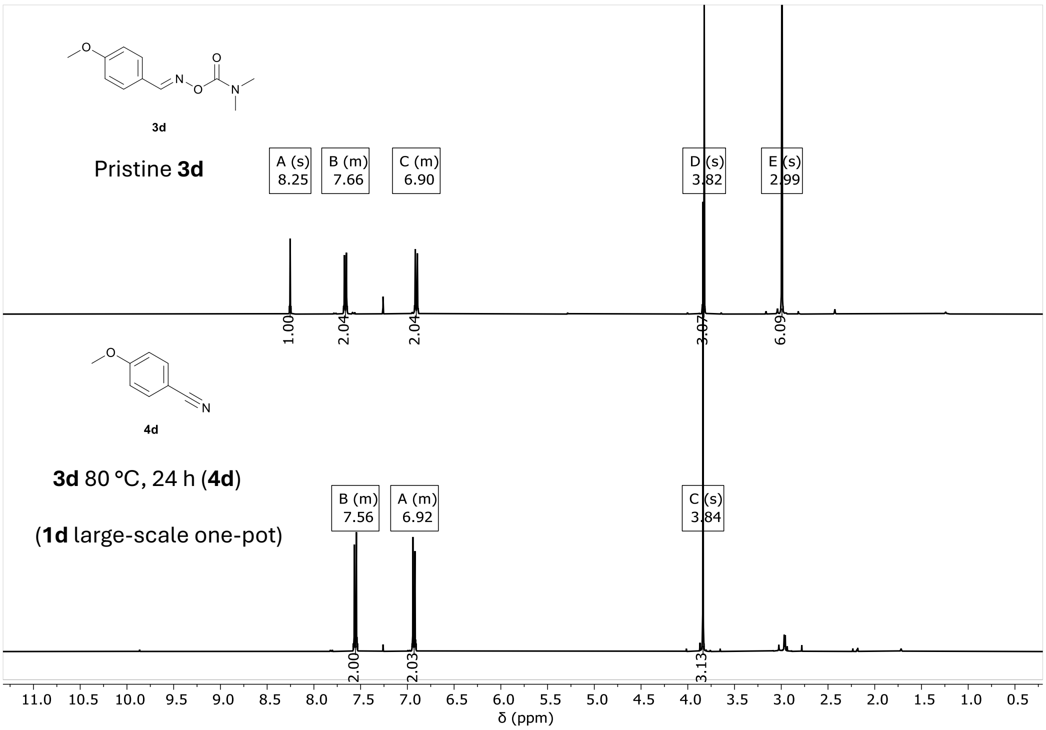

Figure S24. Comparison of ^{1}H NMR spectra of previously synthesized reference 4-methoxybenzaldehyde *O*-dimethylcarbamoyloxime **3d** (top) and 4-methoxybenzonitrile **4d**, obtained by large-scale one-pot synthesis from 4-methoxybenzaldehyde **1d** by heating 4-methoxybenzaldehyde *O*-dimethylcarbamoyloxime **3d** at 80 °C for 24 h (bottom) (in $CDCl_3$). Analysis DOI: https://dx.doi.org/10.14272/XDJAAZYHCCRJOK-UHFFFAOYSA-N/CHMO0000593.2.

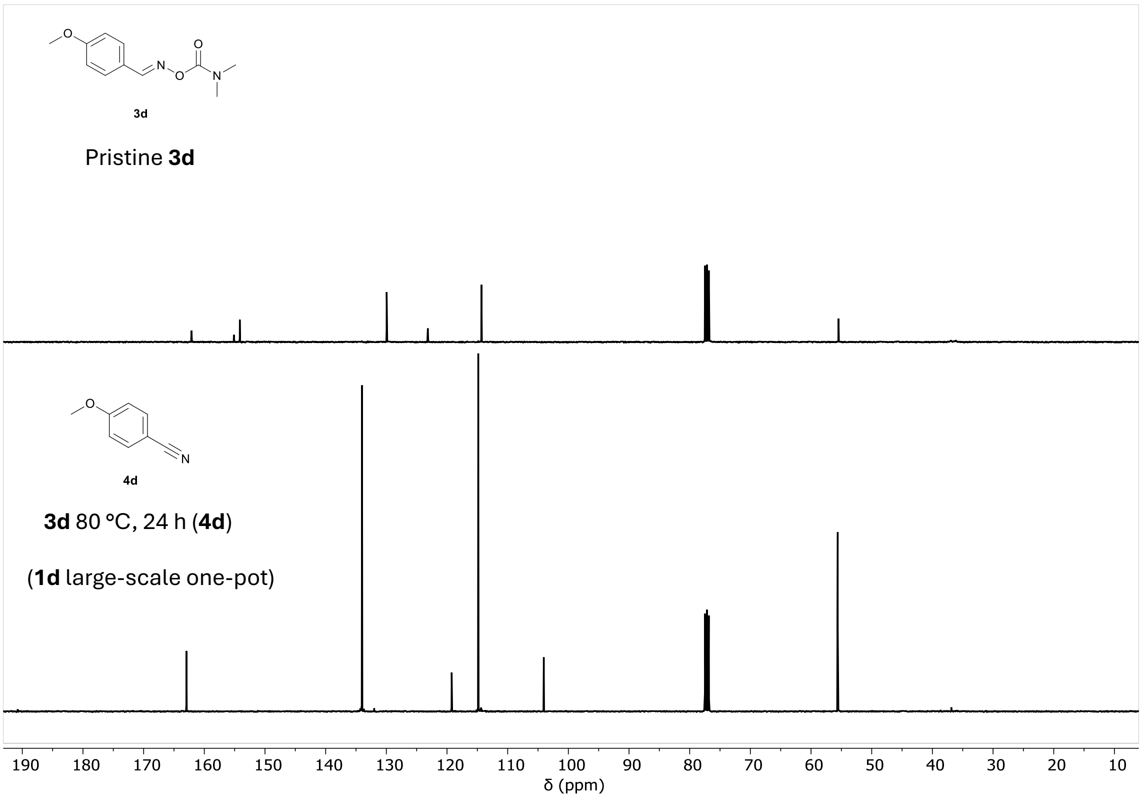

Figure S25. Comparison of ^{13}C NMR spectra of previously synthesized reference 4-methoxybenzaldehyde *O*-dimethylcarbamoyloxime **3d** (top), 4-methoxybenzonitrile **4d**, obtained by large-scale one-pot synthesis from 4-methoxybenzaldehyde **1d** by heating 4-methoxybenzaldehyde *O*-dimethylcarbamoyloxime **3d** at 80 °C for 24 h (bottom) (in $CDCl_3$). Analysis DOI: https://dx.doi.org/10.14272/XDJAAZYHCCRJOK-UHFFFAOYSA-N/CHMO0000595.2.

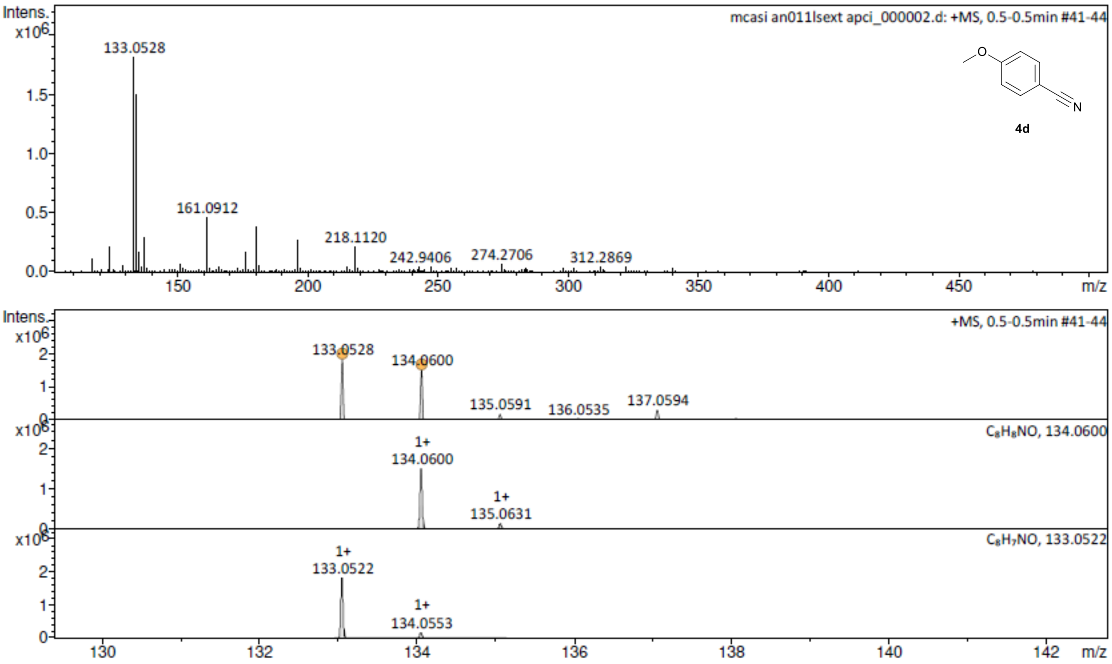

Figure S26. Deconvoluted APCI$^+$ HRMS spectrum (top), along with the comparison between the deconvoluted APCI$^+$ HRMS (top) and the calculated mass spectrum (bottom) of 4-methoxybenzonitrile **4d**, obtained by large-scale one-pot synthesis from 4-methoxybenzaldehyde **1d** by heating 4-methoxybenzaldehyde *O*-dimethylcarbamoyloxime **3d** at 80 °C for 24 h. Analysis DOI: https://dx.doi.org/10.14272/XDJAAZYHCCRJOK-UHFFFAOYSA-N/CHMO0002763.2.

2.6. Rosenmund-von Braun cyanation (4-methoxybenzonitrile **4d**)

A mixture of 1-bromo-4-methoxybenzene (187 mg, 126 µL, 1000 µmol, 1.00 equiv.) and CuCN (107 mg, 36.6 µL, 1.19 mmol, 1.19 equiv.) was refluxed in 10 mL of DMF at 160 °C for 24 h. Afterwards, the resulting suspension was cooled to r.t. and diluted with 10 mL of H_2O. The suspension was extracted with EtOAc (25 mL), and washed with H_2O (3×20 mL), and brine (1×20 mL). The organic layer was dried over $MgSO_4$, filtered, concentrated, and the solvent was removed *in vacuo*. The residue was purified by flash chromatography on silica with (hexane/EtOAc = 9:1) to afford 4-methoxybenzonitrile **4d** as an off-white solid (91.0 mg, 683 µmol, 68% yield). **^{1}H NMR (400 MHz, $CDCl_3$):** δ_H / ppm = 7.60–7.55 (m, 2H), 6.97–6.92 (m, 2H), 3.85 (s, 3H). See Figure S27. **^{13}C NMR (101 MHz, $CDCl_3$):** δ_C / ppm = 163.0, 134.1, 119.3, 114.9, 104.2, 55.7. See Figure S28. **APCI$^+$ HRMS:** m/z = 134.0602 (calcd. 134.0600 for MH^+). See Figure S29. Details of the experiment, molecular characterization data, and metadata can be found under reaction DOIs: https://dx.doi.org/10.14272/reaction/SA-FUHFF-UHFFFADPSC-XDJAAZYHCC-UHFFFADPSC-NUHFF-NUHFF-NUHFF-ZZZ.4.

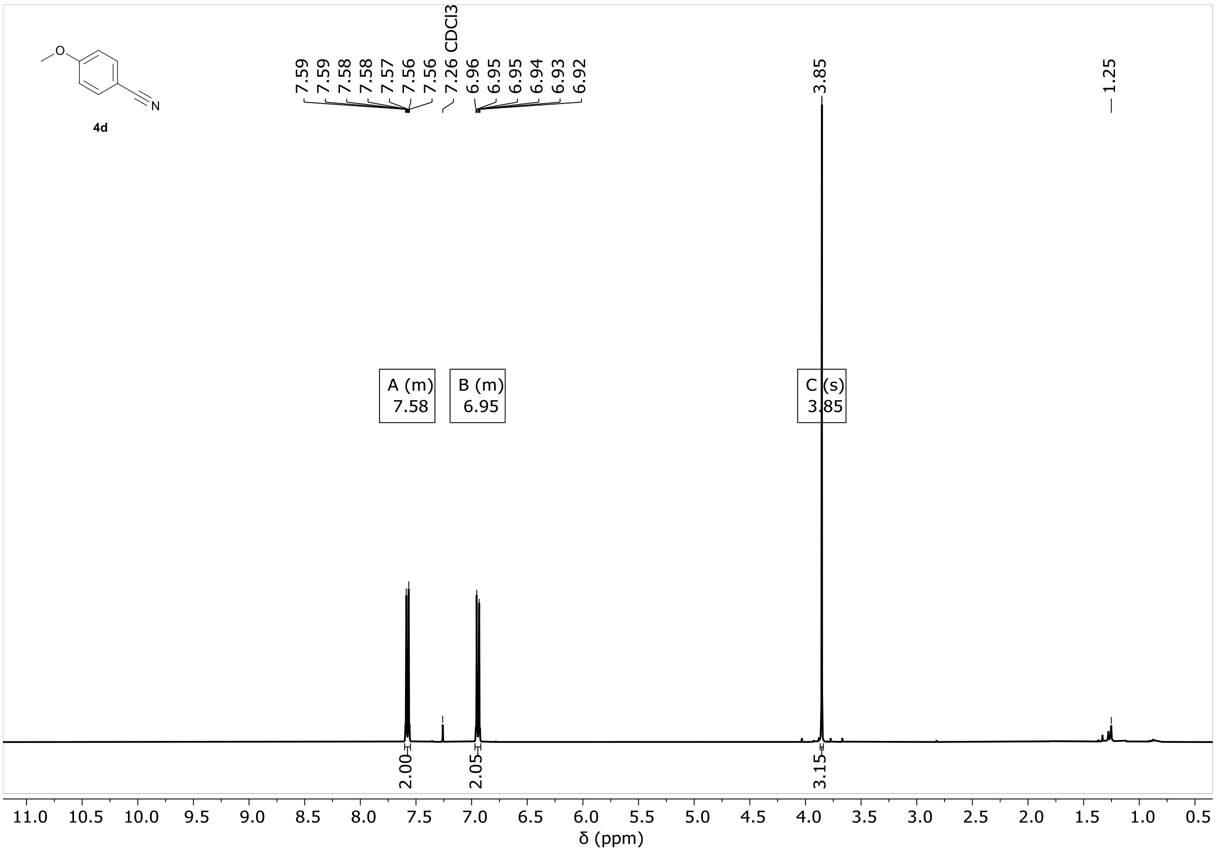

Figure S27. ^{1}H NMR spectrum of 4-methoxybenzonitrile **4d** formed by Rosenmund-von Braun cyanation (in $CDCl_3$). Analysis DOI: https://dx.doi.org/10.14272/XDJAAZYHCCRJOK-UHFFFAOYSA-N/CHMO0000593.1.

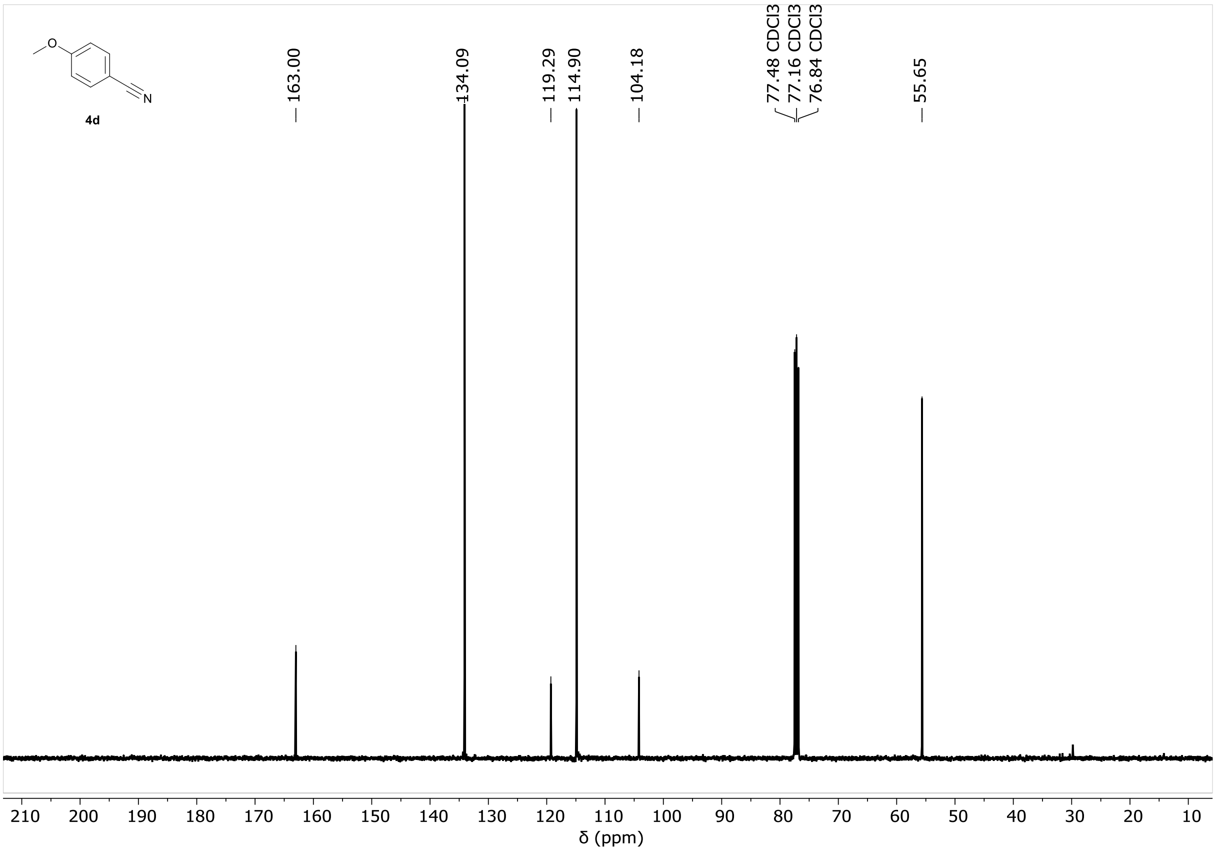

Figure S28. ^{13}C NMR spectrum of 4-methoxybenzonitrile **4d** formed by Rosenmund-von Braun cyanation (in $CDCl_3$). Analysis DOI: https://dx.doi.org/10.14272/XDJAAZYHCCRJOK-UHFFFAOYSA-N/CHMO0000595.1.

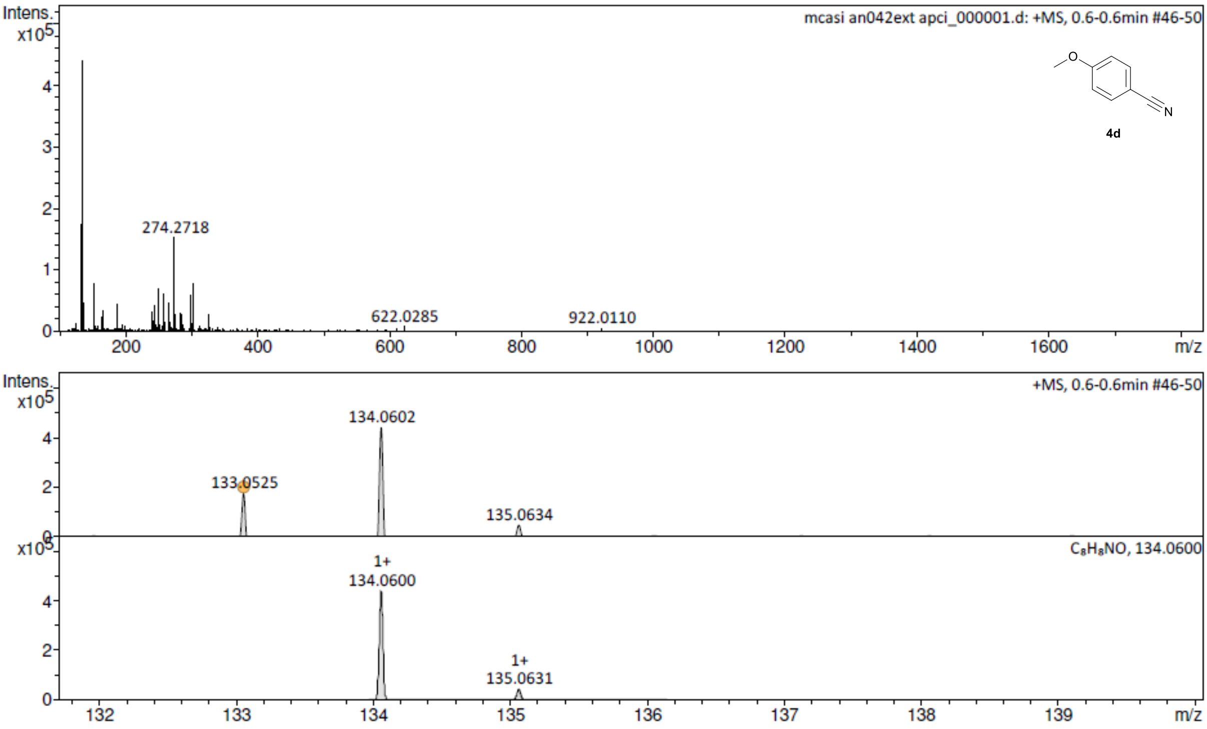

Figure S29. Deconvoluted APCI$^+$ HRMS spectrum of 4-methoxybenzonitrile **4d** formed by Rosenmund-von Braun cyanation. Analysis DOI: https://dx.doi.org/10.14272/XDJAAZYHCCRJOK-UHFFFAOYSA-N/CHMO0002763.1.

2.7. Initial control experiments on the role of the base mixture (pyridine/Et_3N) for the formation of benzaldehyde *O*-dimethylcarbamoyloxime **3a** from benzaldehyde oxime **2a**

2a (103 mg, 0.85 mmol, 1.00 equiv.) and dry Et_3N (172 mg, 237 µL, 1.0 mmol, 2.00 equiv.) were dissolved in dry MeCN (8 mL) in a Schlenk flask under an Ar atmosphere. Subsequently, *N,N*-dimethylcarbamoyl chloride (183 mg, 156 µL, 1.70 mmol, 2.00 equiv.) was injected, and it was stirred at r.t. for 48 h. All volatiles were removed *in vacuo*, and the NMR of the crude mixture was submitted. See Figure S30 and Figure S31.

The same experiment was repeated as follows: **2a** (97 mg, 0.80 mmol, 1.00 equiv.) and dry Et_3N (121 mg, 167 µL, 1.20 mmol, 1.50 equiv.) were dissolved in dry MeCN (8 mL) in a Schlenk flask under an Ar atmosphere. Subsequently, *N,N*-dimethylcarbamoyl chloride (129 mg, 110 µL, 1.20 mmol, 1.50 equiv.) was injected, and it was stirred overnight. The reaction was monitored by TLC, showing no visible formation of **3a**. Afterwards, pyridine (95 mg, 97 µL, 1.50 mmol, 1.50 equiv.) was introduced to the system and stirred at r.t. overnight, whereupon the color of the solution turned from transparent to light yellow. All volatiles were removed *in vacuo*, and the NMR of the crude mixture was submitted. Benzaldehyde *O*-dimethylcarbamoyloxime **3a** was obtained as an off-white solid (145 mg, 0.75 mmol, 94% yield). See Figure S30 and Figure S31.

A similar experiment using pyridine as the sole base was performed; however, the reaction did not reach completion. Here, we only report the stacked ^{1}H NMR of the synthesis of **3a** by using only Et_3N, where a limited conversion is achieved, and the pyridine/Et_3N mixture, where full conversion is observed. Using pyridine was only confirmed no conversion by TLC. Additionally, $Et_3N\cdot HCl$ was present in the ^{1}H NMR spectra since the work-up was not performed for the initial control experiments.

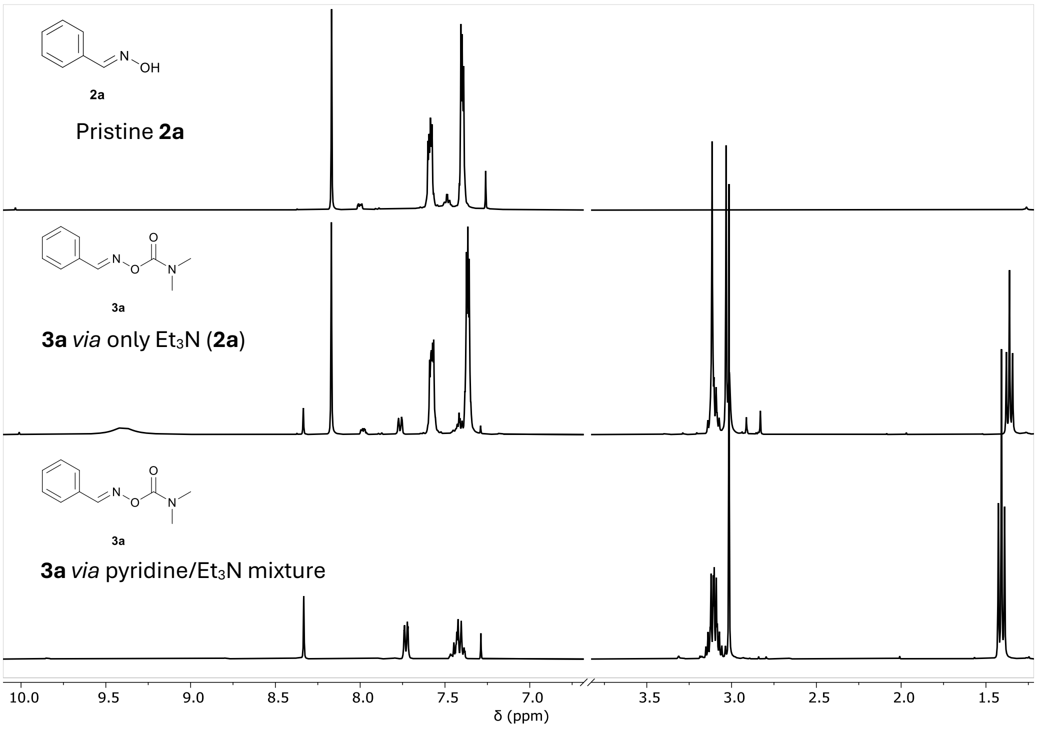

Figure S30. Comparison of ^{1}H NMR spectra of previously synthesized benzaldehyde oxime **2a** (top), formed from benzaldehyde **1a**, the synthesis trial of benzaldehyde *O*-dimethylcarbamoyloxime **3a** from **2a** by using only Et_3N (middle), and pyridine/Et_3N mixture (bottom) as the choice of base (in $CDCl_3$). ^{1}H NMRs contain $Et_3N.HCl$ (middle and bottom), and also the majorly incomplete conversion of **3a**, consistent with the presence of the starting material **2a** (middle). The top spectrum was reported to show the signal changing between **2a** and **3a**.

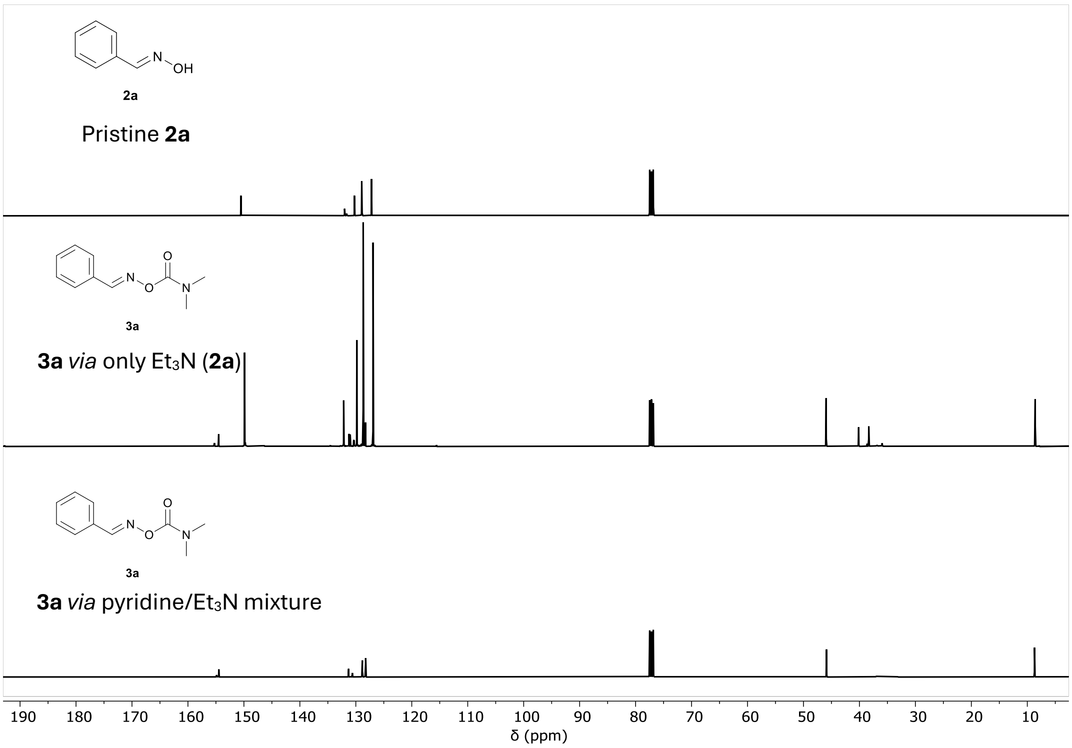

Figure S31. Comparison of ^{13}C NMR spectra of previously synthesized benzaldehyde oxime **2a** (top), formed from benzaldehyde **1a**, the synthesis trial of benzaldehyde *O*-dimethylcarbamoyloxime **3a** from **2a** by using only Et_3N (middle), and pyridine/Et_3N mixture (bottom) as the choice of base (in $CDCl_3$). ^{13}C NMRs contain $Et_3N.HCl$ (middle and bottom), and also the majorly incomplete conversion of **3a**, consistent with the presence of the starting material **2a** (middle). The top spectrum was reported to show the signal changing between **2a** and **3a**.

3. Molecular characterization data

3.1. Characterization data of products and intermediates

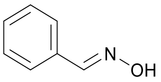

Benzaldehyde oxime (2a): Colorless liquid (400 mg, 83% yield) (R_f = 0.84, CH_2Cl_2/MeOH = 95:5). **^{1}H NMR (400 MHz, $CDCl_3$):** δ_H / ppm = 8.17 (s, 1H), 7.58 (ddd, J = 6.8, 5.0, 3.3 Hz, 2H), 7.43–7.37 (m, 3H). See Figure S32. **^{13}C NMR (101 MHz, $CDCl_3$):** δ_C / ppm = 150.4, 131.9, 130.1, 128.8, 127.0. See Figure S33. **ESI$^+$ HRMS**: m/z = 122.0612 (calcd. 122.0600 for MH^+). See Figure S34. Details of this experimental procedure, molecular characterization data, and metadata are available at the reaction DOI: https://dx.doi.org/10.14272/reaction/SA-FUHFF-UHFFFADPSC-VTWKXBJHBH-UHFFFADPSC-NUHFF-NCGTV-NUHFF-ZZZ.

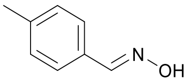

4-Methylbenzaldehyde oxime (2b): Colorless solid (520 mg, 92% purity, 88% yield) (R_f = 0.68, CH_2Cl_2/MeOH = 98:2). **^{1}H NMR (400 MHz, $CDCl_3$):** δ_H / ppm = 8.14 (s, 1H), 7.51–7.42 (m, 2H), 7.23–7.16 (m, 2H), 2.38 (s, 3H). See Figure S35. **^{13}C NMR (101 MHz, $CDCl_3$):** δ_C / ppm = 150.5, 140.5, 129.7, 129.2, 127.1, 21.6. See Figure S36. **ESI$^+$ HRMS**: m/z = 136.0767 (calcd. 136.0757 for MH^+). See Figure S37. Details of this experimental procedure, molecular characterization data, and metadata are available at the reaction DOI: https://dx.doi.org/10.14272/reaction/SA-FUHFF-UHFFFADPSC-SRNDYVBEUZ-UHFFFADPSC-NUHFF-NHSZW-NUHFF-ZZZ.

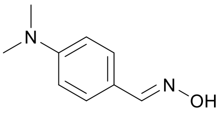

4-(Dimethylamino)benzaldehyde oxime (2c): Off-white solid (280 mg, 85% purity, 72% yield) (R_f = 0.88, EtOAc). **^{1}H NMR (400 MHz, DMSO-d_6):** δ_H / ppm = 10.66 (s, 1H, OH), 7.97 (s, 1H), 7.41–7.37 (m, 2H), 6.72–6.68 (m, 2H), 2.92 (s, 6H). See Figure S38. **^{13}C NMR (101 MHz, DMSO-d_6):** δ_C / ppm = 150.9, 148.1, 127.5, 120.6, 111.9, 39.8. See Figure S39. **ESI$^+$ HRMS**: m/z = 187.0842 (calcd. 187.0842 for MNa^+). See Figure S40. Details of this experimental procedure, molecular characterization data, and metadata are available at the reaction DOI: https://dx.doi.org/10.14272/reaction/SA-FUHFF-UHFFFADPSC-UJYKADYTQM-UHFFFADPSC-NUHFF-NUSVJ-NUHFF-ZZZ.

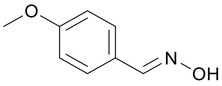

4-Methoxybenzaldehyde oxime (2d): Colorless crystal-like solid (1030 mg, 85% yield) (R_f = 0.61, CH_2Cl_2/MeOH = 98:2). **^{1}H NMR (400 MHz, DMSO-d_6):** δ_H / ppm = 10.97 (s, 1H, OH), 8.07 (s, 1H), 7.54–7.50 (m, 2H), 6.97–6.93 (m, 2H), 3.77 (s, 3H). See Figure S41. **^{13}C NMR (101 MHz, DMSO-d_6):** δ_C / ppm = 160.1, 147.7, 127.9, 125.6, 114.2, 55.2. See Figure S42. **ESI$^+$ HRMS**: m/z = 152.0694 (calcd. 152.0706 for MH^+). See Figure S43. Details of this experimental procedure, molecular characterization data, and metadata are available at the reaction DOI: https://dx.doi.org/10.14272/reaction/SA-FUHFF-UHFFFADPSC-FXOSHPAYNZ-UHFFFADPSC-NUHFF-NHSZW-NUHFF-ZZZ.

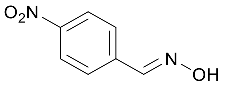

4-Nitrobenzaldehyde oxime (2e): Yellow solid (650 mg, 93% purity, 91% yield) (R_f = 0.80, CH_2Cl_2/MeOH = 98:2). **^{1}H NMR (400 MHz, DMSO-*d*$_6$):** δ_H / ppm = 11.85 (s, 1H, OH), 8.30 (s, 1H), 8.27–8.23 (m, 2H), 7.88–7.83 (m, 2H). See Figure S44. **^{13}C NMR (101 MHz, DMSO-*d*$_6$):** δ_C / ppm = 147.5, 146.9, 139.5, 127.4, 124.0. See Figure S45. **ESI$^+$ HRMS**: *m/z* = 167.0455 (calcd. 167.0451 for MH$^+$). See Figure S46. Details of this experimental procedure, molecular characterization data, and metadata are available at the reaction DOI: https://dx.doi.org/10.14272/reaction/SA-FUHFF-UHFFFADPSC-WTLPAVBACR-UHFFFADPSC-NUHFF-NNFXF-NUHFF-ZZZ.

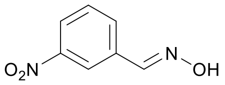

3-Nitrobenzaldehyde oxime (2f): Purified by flash silica gel column chromatography (CH_2Cl_2/MeOH = 98:2 to 95:5, v/v). Off-white solid (1200 mg, 80% yield) (R_f = 0.72, CH_2Cl_2/MeOH = 98:2). **^{1}H NMR (400 MHz, DMSO-*d*$_6$):** δ_H / ppm = 11.65 (s, 1H, OH), 8.40 (dd, J = 2.2, 1.6 Hz, 1H), 8.31 (s, 1H), 8.20 (ddd, J = 8.2, 2.4, 1.1 Hz, 1H), 8.03 (dt, J = 7.7, 1.3 Hz, 1H), 7.68 (t, J = 8.0 Hz, 1H). See Figure S47. **^{13}C NMR (101 MHz, DMSO-*d*$_6$):** δ_C / ppm = 148.1, 146.6, 134.9, 132.3, 130.3, 123.7, 120.8. See Figure S48. **ESI$^-$ HRMS**: *m/z* = 165.0307 (calcd. 165.0306 for M$^-$). See Figure S49. Details of this experimental procedure, molecular characterization data, and metadata are available at the reaction DOI: https://dx.doi.org/10.14272/reaction/SA-FUHFF-UHFFFADPSC-GQMMRLBWXC-UHFFFADPSC-NUHFF-NNFXF-NUHFF-ZZZ.

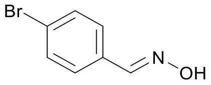

4-Bromobenzaldehyde oxime (2g): Colorless solid (570 mg, 94% purity, 89% yield) (R_f = 0.67, CH_2Cl_2/MeOH = 98:2). **^{1}H NMR (400 MHz, DMSO-*d*$_6$):** δ_H / ppm = 11.37 (s, 1H, OH), 8.13 (s, 1H), 7.62–7.57 (m, 2H), 7.56–7.52 (m, 2H). See Figure S50. **^{13}C NMR (101 MHz, DMSO-*d*$_6$):** δ_C / ppm = 147.2, 132.4, 131.7, 128.3, 122.4. See Figure S51. **ESI$^+$ HRMS**: *m/z* = 199.9699 (calcd. 199.9706 for MH$^+$). See Figure S52. Details of this experimental procedure, molecular characterization data, and metadata are available at the reaction DOI: https://dx.doi.org/10.14272/reaction/SA-FUHFF-UHFFFADPSC-UIIZGAXKZZ-UHFFFADPSC-NUHFF-NNXWJ-NUHFF-ZZZ.

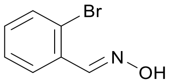

2-Bromobenzaldehyde oxime (2h): Colorless solid (990 mg, 87% yield) (R_f = 0.70, CH_2Cl_2/MeOH = 98:2). **^{1}H NMR (400 MHz, DMSO-*d*$_6$):** δ_H / ppm = 11.69 (s, 1H, OH), 8.32 (s, 1H), 7.79 (dd, J = 7.7, 1.8 Hz, 1H), 7.66 (dd, J = 7.9, 1.3 Hz, 1H), 7.41 (td, J = 7.6, 1.5 Hz, 1H), 7.36–7.30 (m, 1H). See Figure S53. **^{13}C NMR (101 MHz, DMSO-*d*$_6$):** δ_C / ppm = 146.8, 133.0, 131.8, 131.2, 128.1, 127.1, 122.5. See Figure S54. **ESI$^-$ HRMS**: *m/z* = 197.9559 (calcd. 197.9560 for MH$^-$). See Figure S55. Details of this experimental procedure, molecular characterization data, and metadata are available at the reaction DOI: https://dx.doi.org/10.14272/reaction/SA-FUHFF-UHFFFADPSC-PSIRFUPZHP-UHFFFADPSC-NUHFF-NNXWJ-NUHFF-ZZZ.

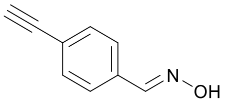

4-Ethynylbenzaldehyde oxime (2i): Orange solid (550 mg, 96% purity, 91% yield) (R_f = 0.84, CH_2Cl_2/MeOH = 98:2). **^{1}H NMR (400 MHz, DMSO-*d*$_6$):** δ_H / ppm = 11.43 (s, 1H, OH), 8.16 (s, 1H), 7.62–7.58 (m, 2H), 7.52–7.47 (m, 2H), 4.28 (s, 1H). See Figure S56. **^{13}C NMR (101 MHz, DMSO-*d*$_6$):** δ_C / ppm = 147.5, 133.5, 132.1, 126.5, 122.3, 83.29, 82.0. See Figure S57. **ESI$^-$ HRMS**: *m/z* = 144.0454 (calcd. 144.0455 for M$^-$). See Figure S58. Details of this experimental procedure, molecular characterization data, and metadata are available at the reaction DOI: https://dx.doi.org/10.14272/reaction/SA-FUHFF-UHFFFADPSC-ZHXYVOBALG-UHFFFADPSC-NUHFF-NUSVJ-NUHFF-ZZZ.

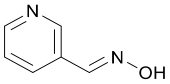

Nicotinaldehyde oxime (2j): Colorless solid (1700 mg, 87% yield) (R_f = 0.18, CH_2Cl_2/MeOH = 98:2). **^{1}H NMR (400 MHz, DMSO-*d*$_6$):** δ_H / ppm = 11.53 (s, 1H, OH), 8.75 (dd, J = 2.2, 0.8 Hz, 1H), 8.56 (dd, J = 4.8, 1.6 Hz, 1H), 8.20 (s, 1H), 7.98 (dt, J = 8.0, 2.0 Hz, 1H), 7.42 (ddd, J = 8.0, 4.8, 0.9 Hz, 1H). See Figure S59. **^{13}C NMR (101 MHz, DMSO-*d*$_6$):** δ_C / ppm = 150.1, 147.9, 145.7, 133.1, 129.0, 123.9. See Figure S60. **ESI$^-$ HRMS**: *m/z* = 121.0410 (calcd. 121.0407 for M$^-$). See Figure S61. See Figure S58. Details of this experimental procedure, molecular characterization data, and metadata are available at the reaction DOI: https://dx.doi.org/10.14272/reaction/SA-FUHFF-UHFFFADPSC-YBKOPFQCLS-UHFFFADPSC-NUHFF-NNFXF-NUHFF-ZZZ.

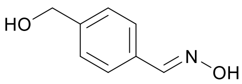

4-(Hydroxymethyl)benzaldehyde oxime (2k): Colorless solid (1020 mg, 95% purity, 80% yield) (R_f = 0.97, CH_2Cl_2). **^{1}H NMR (400 MHz, DMSO-*d*$_6$):** δ_H / ppm = 11.14 (s, 1H, OH), 8.11 (s, 1H), 7.57–7.52 (m, 2H), 7.35–7.31 (m, 2H), 5.23 (t, 1H), 4.50 (d, 2H). See Figure S62. **^{13}C NMR (151 MHz, DMSO-*d*$_6$):** δ_C / ppm = 148.0, 143.8, 131.5, 126.6, 126.2, 62.6. See Figure S63. **ESI$^-$ HRMS**: *m/z* = 150.0561 (calcd. 150.0561 for MH$^-$). See Figure S64. Details of this experimental procedure, molecular characterization data, and metadata are available at the reaction DOI: https://dx.doi.org/10.14272/reaction/SA-FUHFF-UHFFFADPSC-DTMHHXCNEA-UHFFFADPSC-NUHFF-NNXWJ-NUHFF-ZZZ.

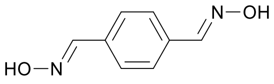

Terephthalaldehyde dioxime (2l): Colorless solid (1450 mg, 98% yield) (R_f = 0.41, CH_2Cl_2/MeOH = 95:5). **^{1}H NMR (400 MHz, DMSO-*d*$_6$):** δ_H / ppm = 11.34 (s, 2H, OH), 8.14 (s, 2H), 7.61 (s, 4H). See Figure S65. **^{13}C NMR (101 MHz, DMSO-*d*$_6$):** δ_C / ppm = 147.8, 133.8, 126.7. Figure S66. **ESI$^-$ HRMS**: *m/z* = 163.0514 (calcd. 163.0513 for M$^-$). See Figure S67. Details of this experimental procedure, molecular characterization data, and metadata are available at the reaction DOI: https://dx.doi.org/10.14272/reaction/SA-FUHFF-UHFFFADPSC-UFJKQCPYFK-UHFFFADPSC-NUHFF-NOETL-NUHFF-ZZZ.

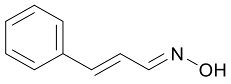

Cinnamaldehyde oxime (2m): Off-white solid (1060 mg, 94% purity, 85% yield) (R_f = 0.55, CH_2Cl_2/MeOH = 98:2). **^{1}H NMR (400 MHz, DMSO-*d*$_6$):** δ_H / ppm = 11.12 (s, 1H, OH), 7.91 (dd, J = 7.2, 1.8 Hz, 1H), 7.56–7.52 (m, 2H), 7.39–7.34 (m, 2H), 7.32–7.27 (m, 1H), 6.96–6.88 (m, 2H). See Figure S68. **^{13}C NMR (101 MHz, DMSO-*d*$_6$):** δ_C / ppm = 150.3, 136.6, 136.1, 128.8, 128.4, 126.8, 123.1. See Figure S69. **ESI$^+$ HRMS**: *m/z* = 170.0579 (calcd. 170.0576 for MNa^+). See Figure S70. Details of this experimental procedure, molecular characterization data, and metadata are available at the reaction DOI: https://dx.doi.org/10.14272/reaction/SA-FUHFF-UHFFFADPSC-RUQDOYIAKH-UHFFFADPSC-NUHFF-NPMLY-NUHFF-ZZZ.

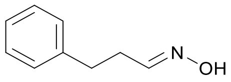

3-Phenylpropanal oxime (2n): Colorless solid (1300 mg, 93% purity, 90% yield) (R_f = 0.38, CH_2Cl_2/MeOH = 98:2). **^{1}H NMR (400 MHz, DMSO-*d*$_6$):** δ_H / ppm = 10.83 (s, 1H, OH), 7.31–7.26 (m, 2H), 7.24–7.17 (m, 3H), 6.67 (t, J = 5.2 Hz, 1H), 2.74 (t, J = 7.6 Hz, 2H), 2.57–2.51 (m, 2H). See Figure S71. **^{13}C NMR (101 MHz, DMSO-*d*$_6$):** δ_C / ppm = 149.4, 141.0, 128.2, 128.0, 125.8, 31.2, 26.0. See Figure S72. **ESI$^+$ HRMS**: *m/z* = 172.0731 (calcd. 172.0731 for MNa^+). See Figure S73. Details of this experimental procedure, molecular characterization data, and metadata are available at the reaction DOI: https://dx.doi.org/10.14272/reaction/SA-FUHFF-UHFFFADPSC-WSTRHGOVAO-UHFFFADPSC-NUHFF-NCIIM-NUHFF-ZZZ.

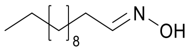

Dodecanal oxime (2o): Colorless crystal-like solid (1190 mg, 99% yield) (R_f = 0.44, CH_2Cl_2). **^{1}H NMR (400 MHz, DMSO-*d*$_6$):** δ_H / ppm = 10.70 (s, 1H, OH), 6.62 (t, J = 5.3 Hz, 1H), 2.21 (t, J = 5.4 Hz, 2H), 1.39 (p, J = 7.3 Hz, 2H), 1.33–1.19 (m, 16H), 0.85 (t, 3H). See Figure S74. **^{13}C NMR (101 MHz, DMSO-*d*$_6$):** δ_C / ppm = 150.3, 31.3, 29.0, 29.0, 28.9, 28.9, 28.8, 28.7, 25.6, 24.5, 22.1, 13.9. See Figure S75. **APCI$^+$ HRMS**: *m/z* = 200.2012 (calcd. 200.2009 for MH^+). See Figure S76. Details of this experimental procedure, molecular characterization data, and metadata are available at the reaction DOI: https://dx.doi.org/10.14272/reaction/SA-FUHFF-UHFFFADPSC-GKHOHOBTPP-UHFFFADPSC-NUHFF-NZGMW-NUHFF-ZZZ.

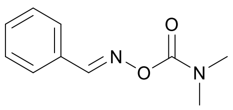

Benzaldehyde *O*-dimethylcarbamoyloxime (3a): Off-white solid (720 mg, 94% yield) (R_f = 0.50, CH_2Cl_2/MeOH = 98:2). **^{1}H NMR (400 MHz, DMSO-*d*$_6$):** δ_H / ppm = 8.62 (s, 1H), 7.77–7.68 (m, 2H), 7.55–7.44 (m, 3H), 2.95 (s, 3H), 2.92 (s, 3H). See Figure S77. **^{13}C NMR (101 MHz, DMSO-*d*$_6$):** δ_C / ppm = 155.0, 153.8, 131.3, 130.6, 129.0, 127.8, 36.4, 35.6. See Figure S78. **ESI$^+$ HRMS**: *m/z* = 215.0799 (calcd. 215.0791 for MNa^+). See Figure S79. Details of this experimental procedure, molecular characterization data, and metadata are available at the reaction DOI: https://dx.doi.org/10.14272/reaction/SA-FUHFF-UHFFFADPSC-JFDJXXRTHC-UHFFFADPSC-NUHFF-NFMKG-NUHFF-ZZZ.

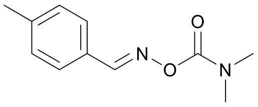

4-Methylbenzaldehyde *O*-dimethylcarbamoyloxime (3b): Off-white solid (150 mg, 92% yield) (R_f = 0.62, CH_2Cl_2/MeOH = 98:2). **^{1}H NMR (400 MHz, DMSO-d_6):** δ_H / ppm = 8.56 (s, 1H), 7.64–7.59 (m, 2H), 7.32–7.26 (m, 2H), 2.94 (s, 3H), 2.92 (s, 3H), 2.35 (s, 3H). See Figure S80. **^{13}C NMR (101 MHz, DMSO-d_6):** δ_C / ppm = 154.9, 153.9, 141.3, 129.6, 127.8, 127.8, 36.3, 35.6, 21.1. See Figure S81. **ESI$^+$ HRMS**: *m/z* = 229.0952 (calcd. 229.0947 for MNa$^+$). See Figure S82. Details of this experimental procedure, molecular characterization data, and metadata are available at the reaction DOI: https://dx.doi.org/10.14272/reaction/SA-FUHFF-UHFFFADPSC-OMJKKBOLQU-UHFFFADPSC-NUHFF-NXJJT-NUHFF-ZZZ

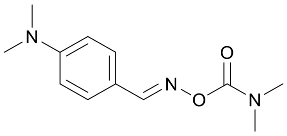

4-(Dimethylamino)benzaldehyde *O*-dimethylcarbamoyloxime (3c): Off-white solid (210 mg, 90% yield) (R_f = 0.55, EtOAc). **^{1}H NMR (400 MHz, $CDCl_3$):** δ_H / ppm = 8.20 (s, 1H), 7.61–7.56 (m, 2H), 6.68–6.63 (m, 2H), 3.00 (s, 6H), 2.99 (s, 6H). See Figure S83. **^{13}C NMR (101 MHz, $CDCl_3$):** δ_C / ppm = 155.4, 154.9, 152.3, 129.8, 117.8, 111.7, 40.2, 36.2. See Figure S84. **ESI$^+$ HRMS**: *m/z* = 258.0961 (calcd. 258.1213 for MNa$^+$). See Figure S85. Details of this experimental procedure, molecular characterization data, and metadata are available at the reaction DOI: https://dx.doi.org/10.14272/reaction/SA-FUHFF-UHFFFADPSC-IMIYGBBGUP-UHFFFADPSC-NUHFF-NKGKN-NUHFF-ZZZ.

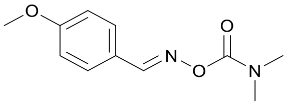

4-Methoxybenzaldehyde *O*-dimethylcarbamoyloxime (3d): Colorless solid (300 mg, 90% yield) (R_f = 0.46, CH_2Cl_2/MeOH = 98:2). **^{1}H NMR (400 MHz, DMSO-d_6):** δ_H / ppm = 8.52 (s, 1H), 7.71–7.63 (m, 2H), 7.07–6.99 (m, 2H), 3.81 (s, 3H), 2.91 (s, 6H). See Figure S86. **^{13}C NMR (101 MHz, DMSO-d_6):** δ_C / ppm = 161.6, 154.6, 154.0, 129.5, 122.9, 114.5, 55.4, 36.3, 36.0. See Figure S87. **ESI$^+$ HRMS**: *m/z* = 245.0919 (calcd. 245.0897 for MNa$^+$). See Figure S88. Details of this experimental procedure, molecular characterization data, and metadata are available at the reaction DOI: https://dx.doi.org/10.14272/reaction/SA-FUHFF-UHFFFADPSC-SIJYEXCZGU-UHFFFADPSC-NUHFF-NXJJT-NUHFF-ZZZ.

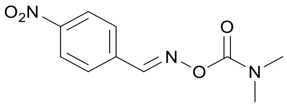

4-Nitrobenzaldehyde *O*-dimethylcarbamoyloxime (3e): Pale yellow solid (215 mg, 91% yield) (R_f = 0.81, CH_2Cl_2/MeOH = 98:2). **^{1}H NMR (400 MHz, DMSO-d_6):** δ_H / ppm = 8.80 (s, 1H), 8.36–8.31 (m, 2H), 8.02–7.97 (m, 2H), 2.97 (s, 3H), 2.92 (s, 3H). See Figure S89. **^{13}C NMR (101 MHz, DMSO-d_6):** δ_C / ppm = 153.4, 148.8, 136.9, 129.0, 124.2, 36.4, 35.6. See Figure S90. **ESI$^+$ HRMS**: *m/z* = 260.0661 (calcd. 260.0642 for MNa$^+$). See Figure S91. Details of this experimental procedure, molecular characterization data, and metadata are available at the reaction DOI: https://dx.doi.org/10.14272/reaction/SA-FUHFF-UHFFFADPSC-HRGJLBWBLQ-UHFFFADPSC-NUHFF-NHCNC-NUHFF-ZZZ.

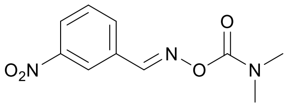

3-Nitrobenzaldehyde *O*-dimethylcarbamoyloxime (3f): Pale yellow solid (810 mg, 85% yield) (R_f = 0.92, CH_2Cl_2). **^{1}H NMR (400 MHz, DMSO-*d*$_6$):** δ_H / ppm = 8.79 (s, 1H), 8.53 (t, J = 2.0 Hz, 1H), 8.34 (ddd, J = 8.2, 2.4, 1.0 Hz, 1H), 8.16 (dt, J = 7.7, 1.3 Hz, 1H), 7.79 (t, J = 8.0 Hz, 1H), 2.97 (s, 3H), 2.92 (s, 3H). See Figure S92. **^{13}C NMR (101 MHz, DMSO-*d*$_6$):** δ_C / ppm = 153.5, 153.3, 148.1, 133.7, 132.4, 130.7, 125.5, 122.2, 36.4, 35.6. See Figure S93. **ESI$^+$ HRMS**: *m/z* = 260.0647 (calcd. 260.0642 for MNa^+). See Figure S94. Details of this experimental procedure, molecular characterization data, and metadata are available at the reaction DOI: https://dx.doi.org/10.14272/reaction/SA-FUHFF-UHFFFADPSC-PALBVJICMH-UHFFFADPSC-NUHFF-NHCNC-NUHFF-ZZZ.

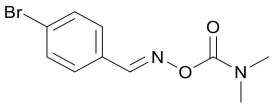

4-Bromobenzaldehyde *O*-dimethylcarbamoyloxime (3g): Yellow solid (250 mg, 92% yield) (R_f = 0.89, CH_2Cl_2/MeOH = 98:2). **^{1}H NMR (400 MHz, DMSO-*d*$_6$):** δ_H / ppm = 8.62 (s, 1H), 7.72–7.66 (m, 4H), 2.95 (s, 3H), 2.92 (s, 3H). See Figure S95. **^{13}C NMR (101 MHz, DMSO-*d*$_6$):** δ_C / ppm = 154.1, 153.7, 132.1, 129.9, 129.7, 124.8, 36.4, 35.6. See Figure S96. **ESI$^+$ HRMS**: *m/z* = 292.9880 (calcd. 292.9896 for MNa^+). See Figure S97. Details of this experimental procedure, molecular characterization data, and metadata are available at the reaction DOI: https://dx.doi.org/10.14272/reaction/SA-FUHFF-UHFFFADPSC-IETXEXJWMC-UHFFFADPSC-NUHFF-NIDTW-NUHFF-ZZZ.

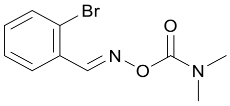

2-Bromobenzaldehyde *O*-dimethylcarbamoyloxime (3h): Orange liquid (700 mg, 80% yield) (R_f = 0.79, CH_2Cl_2/MeOH = 98:2). **^{1}H NMR (400 MHz, DMSO-*d*$_6$):** δ_H / ppm = 8.72 (s, 1H), 7.86 (dd, J = 7.4, 2.2 Hz, 1H), 7.78–7.73 (m, 1H), 7.53–7.42 (m, 2H), 2.97 (s, 3H), 2.91 (s, 3H). See Figure S98. **^{13}C NMR (101 MHz, DMSO-*d*$_6$):** δ_C / ppm = 153.5, 133.3, 133.0, 132.4, 130.4, 129.8, 128.3, 118.3, 36.4, 35.7. See Figure S99. **ESI$^+$ HRMS**: *m/z* = 292.9892 (calcd. 292.9896 for MNa^+). See Figure S100. Details of this experimental procedure, molecular characterization data, and metadata are available at the reaction DOI: https://dx.doi.org/10.14272/reaction/SA-FUHFF-UHFFFADPSC-XHSCOJMVTU-UHFFFADPSC-NUHFF-NIDTW-NUHFF-ZZZ.

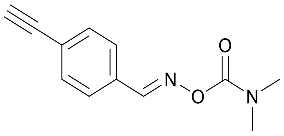

4-Ethynylbenzaldehyde *O*-dimethylcarbamoyloxime (3i): Pale orange solid (580 mg, 90% yield) (R_f = 0.96 CH_2Cl_2/MeOH = 98:2). **^{1}H NMR (400 MHz, DMSO-*d*$_6$):** δ_H / ppm = 8.64 (s, 1H), 7.76–7.71 (m, 2H), 7.60–7.56 (m, 2H), 4.40 (s, 1H), 2.94 (s, 3H), 2.91 (s, 3H). See Figure S101. **^{13}C NMR (101 MHz, DMSO-*d*$_6$):** δ_C / ppm = 154.2, 153.7, 132.3, 131.0, 128.0, 124.3, 83.1, 82.9, 36.4, 35.6. See Figure S102. **ESI$^+$ HRMS**: *m/z* = 239.0793 (calcd. 239.0791 for MNa^+). See Figure S103. Details of this experimental procedure, molecular characterization data, and metadata are available at the reaction DOI: https://dx.doi.org/10.14272/reaction/SA-FUHFF-UHFFFADPSC-BIJWBGUJDO-UHFFFADPSC-NUHFF-NKGKN-NUHFF-ZZZ.

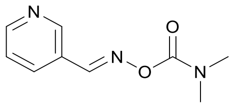

Nicotinaldehyde *O*-dimethylcarbamoyloxime (3j): Orange solid (990 mg, 99% yield) (R_f = 0.33, CH_2Cl_2/MeOH = 98:2). **^{1}H NMR (400 MHz, DMSO-d_6):** δ_H / ppm = 8.86 (dd, J = 2.3, 0.9 Hz, 1H), 8.70 (s, 1H), 8.69 (dd, J = 4.8, 1.7 Hz, 1H), 8.13 (dt, J = 8.0, 2.0 Hz, 1H), 7.52 (ddd, J = 8.0, 4.8, 0.9 Hz, 1H), 2.96 (s, 3H), 2.91 (s, 3H). See Figure S104. **^{13}C NMR (101 MHz, DMSO-d_6):** δ_C / ppm = 153.6, 152.9, 151.9, 149.2, 134.3, 126.8, 124.1, 36.4, 35.6. See Figure S105. **ESI$^+$ HRMS**: *m/z* = 216.0739 (calcd. 216.0743 for MNa^+). See Figure S106. Details of this experimental procedure, molecular characterization data, and metadata are available at the reaction DOI: https://dx.doi.org/10.14272/reaction/SA-FUHFF-UHFFFADPSC-ZIFBKQWXTN-UHFFFADPSC-NUHFF-NHCNC-NUHFF-ZZZ.

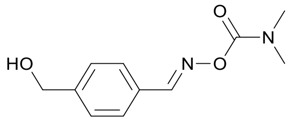

4-(Hydroxymethyl)benzaldehyde *O*-dimethylcarbamoyloxime (3k): Colorless solid (1000 mg, 90% yield) (R_f = 0.18, CH_2Cl_2/MeOH = 98:2). **^{1}H NMR (400 MHz, DMSO-d_6):** δ_H / ppm = 8.58 (s, 1H), 7.72–7.66 (m, 2H), 7.46–7.39 (m, 2H), 5.32 (t, *J* = 5.7 Hz, 1H), 4.55 (d, *J* = 5.6 Hz, 2H), 2.93 (s, 6H). See Figure S107. **^{13}C NMR (101 MHz, DMSO-d_6):** δ_C / ppm = 154.9, 153.9, 146.1, 128.9, 127.7, 126.8, 62.5, 36.3, 35.6. See Figure S108. **ESI$^+$ HRMS**: *m/z* = 245.0896 (calcd. 245.0897 for MNa^+). See Figure S109. Details of this experimental procedure, molecular characterization data, and metadata are available at the reaction DOI: https://dx.doi.org/10.14272/reaction/SA-FUHFF-UHFFFADPSC-PEOMWEHVPD-UHFFFADPSC-NUHFF-NIDTW-NUHFF-ZZZ.

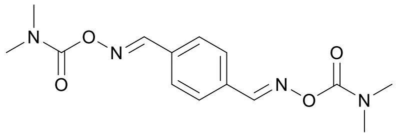

Terephthalaldehyde *O,O*-didimethylcarbamoyl dioxime (3l): Colorless solid (1170 mg, 95% yield) (R_f = 0.37, EtOAc/MeOH = 95:5). **^{1}H NMR (400 MHz, $CDCl_3$):** δ_H / ppm = 8.33 (s, 2H), 7.78 (s, 4H), 3.02 (s, 12H). See Figure S110. **^{13}C NMR (101 MHz, $CDCl_3$):** δ_C / ppm = 154.8, 153.6, 133.3, 128.6, 36.2. See Figure S111. **ESI$^+$ HRMS**: *m/z* = 329.1223 (calcd. 329.1220 for MNa^+). See Figure S112. Details of this experimental procedure, molecular characterization data, and metadata are available at the reaction DOI: https://dx.doi.org/10.14272/reaction/SA-FUHFF-UHFFFADPSC-MCZIYKJYNV-UHFFFADPSC-NUHFF-NOJJC-NUHFF-ZZZ

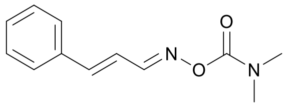

Cinnamaldehyde *O*-dimethylcarbamoyloxime (3m): Colorless solid (750 mg, 86% yield) (R_f = 0.94, CH_2Cl_2/MeOH = 98:2). **^{1}H NMR (400 MHz, $CDCl_3$):** δ_H / ppm = 8.10 (d, J = 9.5 Hz, 1H), 7.48–7.44 (m, 2H), 7.39–7.31 (m, 3H), 7.04 (dd, J = 16.1, 9.5 Hz, 1H), 6.94 (d, J = 16.0 Hz, 1H), 2.99 (s, 6H). See Figure S113. **^{13}C NMR (101 MHz, $CDCl_3$):** δ_C / ppm = 155.6, 154.9, 142.3, 135.5, 129.7, 129.0, 127.4, 121.0, 37.0, 36.1. See Figure S114. **ESI$^+$ HRMS**: *m/z* = 241.0948 (calcd. 241.0947 for MNa^+). See Figure S115. Details of this experimental procedure, molecular characterization data, and metadata are available at the reaction DOI: https://dx.doi.org/10.14272/reaction/SA-FUHFF-UHFFFADPSC-OIXDETWCKL-UHFFFADPSC-NUHFF-NCUKA-NUHFF-ZZZ.

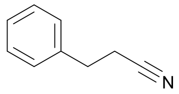

3-phenylpropanenitrile (4n): Purified by vacuum distillation starting from 50 °C to 90 °C. Colorless liquid (Yield is not applicable since the total amount could not be recovered during the distillation; however, full conversion is achieved after 90 °C) (R_f =0.94, CH_2Cl_2/MeOH = 98:2). **^{1}H NMR (400 MHz, $CDCl_3$):** δ_H / ppm = 7.37 (m, 2H), 7.31 (m, 1H), 7.26 (dd, 2H), 2.95 (t, 2H), 2.61 (t, 2H). See Figure S116. **^{13}C NMR (101 MHz, $CDCl_3$):** δ_C / ppm = 138.1, 128.8, 128.2, 127.1, 119.2, 31.4, 19.2. See Figure S117. The spectroscopic data obtained were in accordance with the literature.[1] Details of this experimental procedure, molecular characterization data, and metadata are available at the reaction DOI: https://dx.doi.org/10.14272/reaction/SA-FUHFF-UHFFFADPSC-CDESRRHCBJ-UHFFFADPSC-NUHFF-NXEPH-NUHFF-ZZZ.

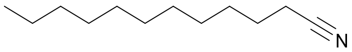

Dodecanenitrile (4o): Purified by vacuum distillation starting from 50 °C to 90 °C. Colorless liquid (Yield is not applicable since the total amount could not be recovered during the distillation; however, full conversion is achieved after 90 °C (R_f = 0.91, CH_2Cl_2/MeOH = 98:2). **^{1}H NMR (400 MHz, $CDCl_3$):** δ_H / ppm = 2.30 (t, J = 7.1 Hz, 2H), 1.62 (p, J = 7.2 Hz, 2H), 1.41 (p, J = 7.3 Hz, 2H), 1.32–1.20 (m, 14H), 0.85 (t, 3H). See Figure S118. **^{13}C NMR (101 MHz, $CDCl_3$):** δ_C / ppm = 119.8, 31.9, 29.6, 29.5, 29.3, 28.8, 28.7, 25.4, 22.7, 17.1, 14.1. See Figure S119. MS data with different ionization techniques was not obtainable due to the sample's volatility. The spectroscopic data obtained were in accordance with the literature.[2] Details of this experimental procedure, molecular characterization data, and metadata are available at the reaction DOI: https://dx.doi.org/10.14272/reaction/SA-FUHFF-UHFFFADPSC-NAIMFYDRSL-UHFFFADPSC-NUHFF-NBVIN-NUHFF-ZZZ.

3.2. ^{1}H NMR, ^{13}C NMR, and mass spectra

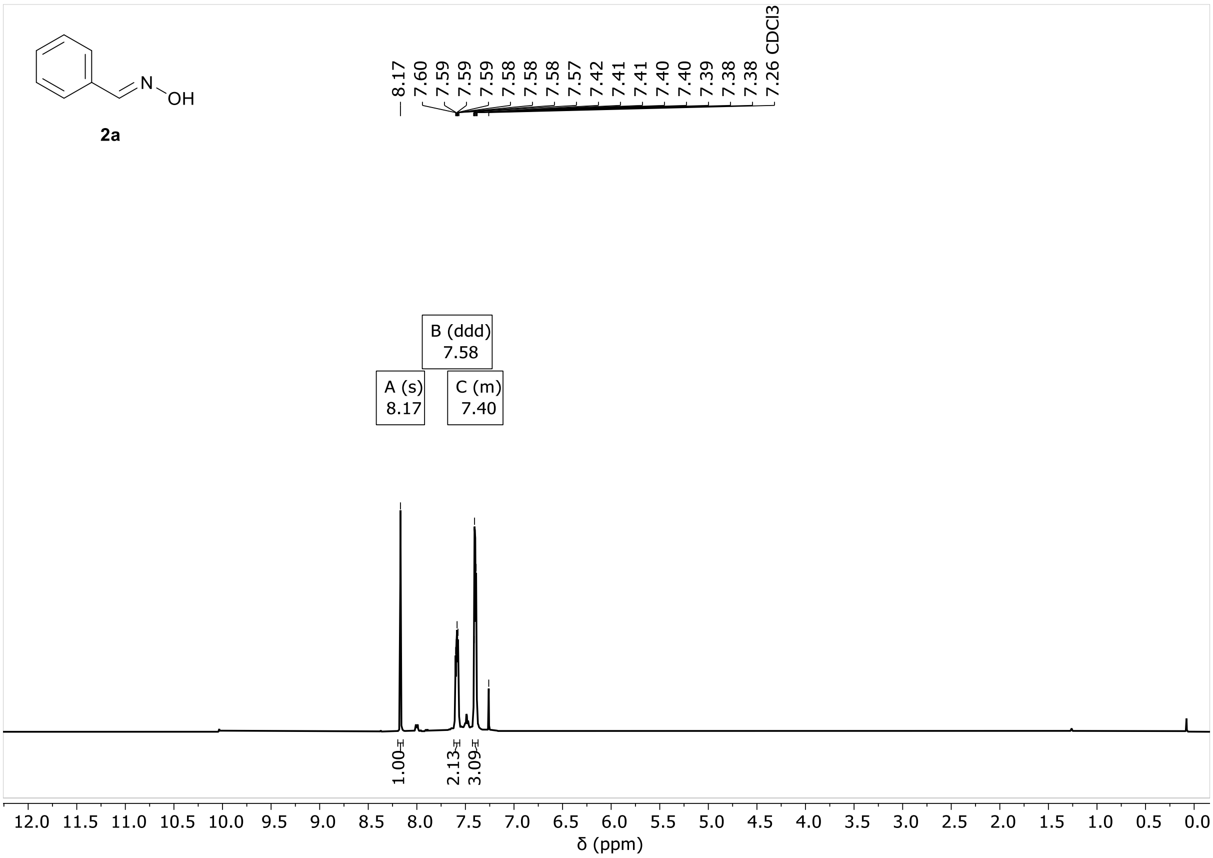

Figure S32. ^{1}H NMR spectrum of benzaldehyde oxime **2a** in $CDCl_3$.

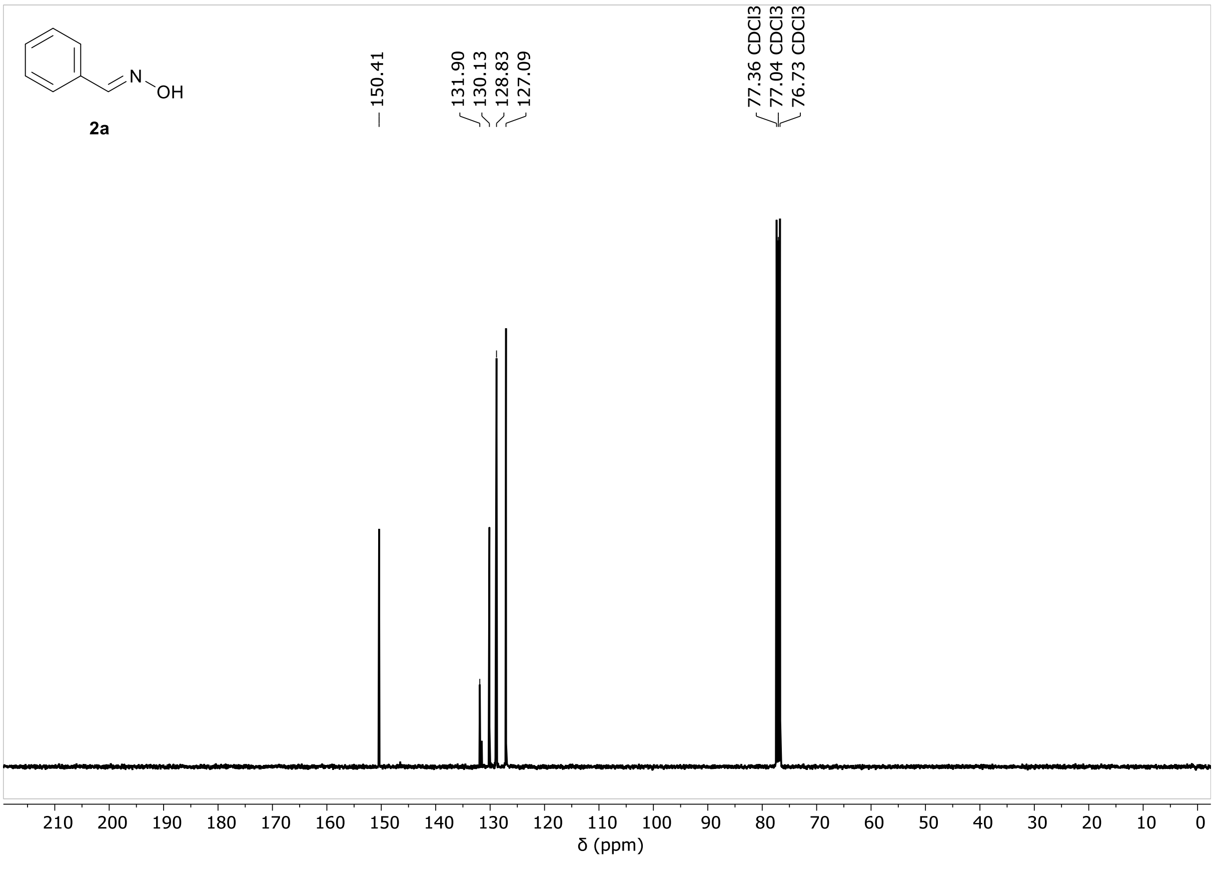

Figure S33. ^{13}C NMR spectrum of benzaldehyde oxime **2a** in $CDCl_3$.

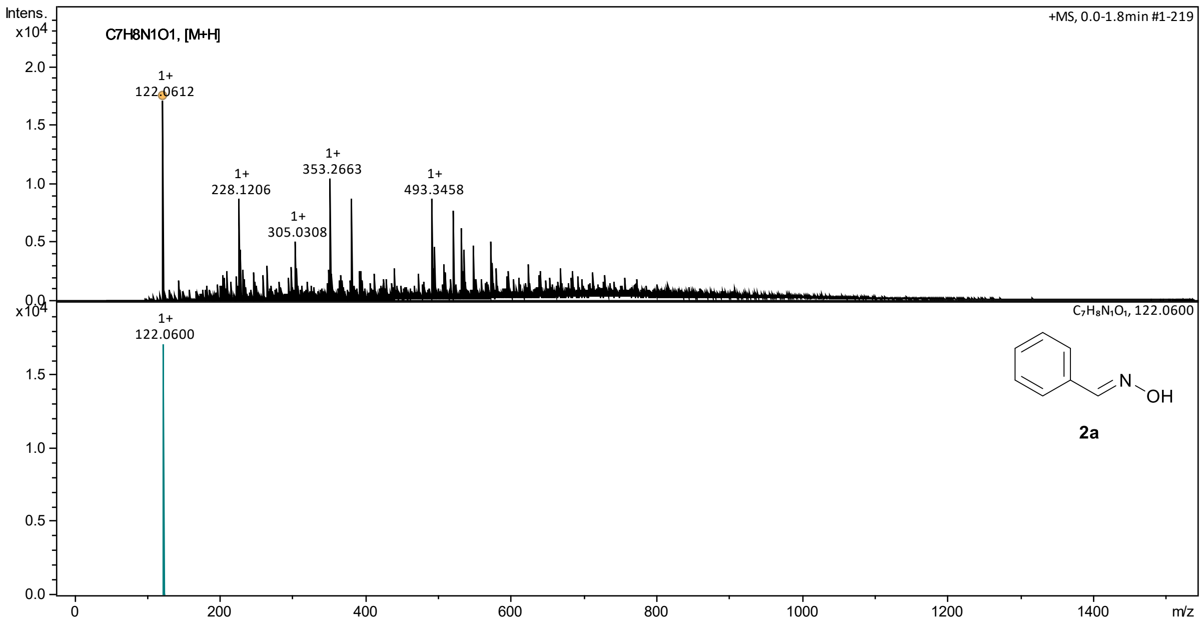

Figure S34. Deconvoluted ESI$^+$ HRMS (top) and calculated mass (bottom) spectra of benzaldehyde oxime **2a.**

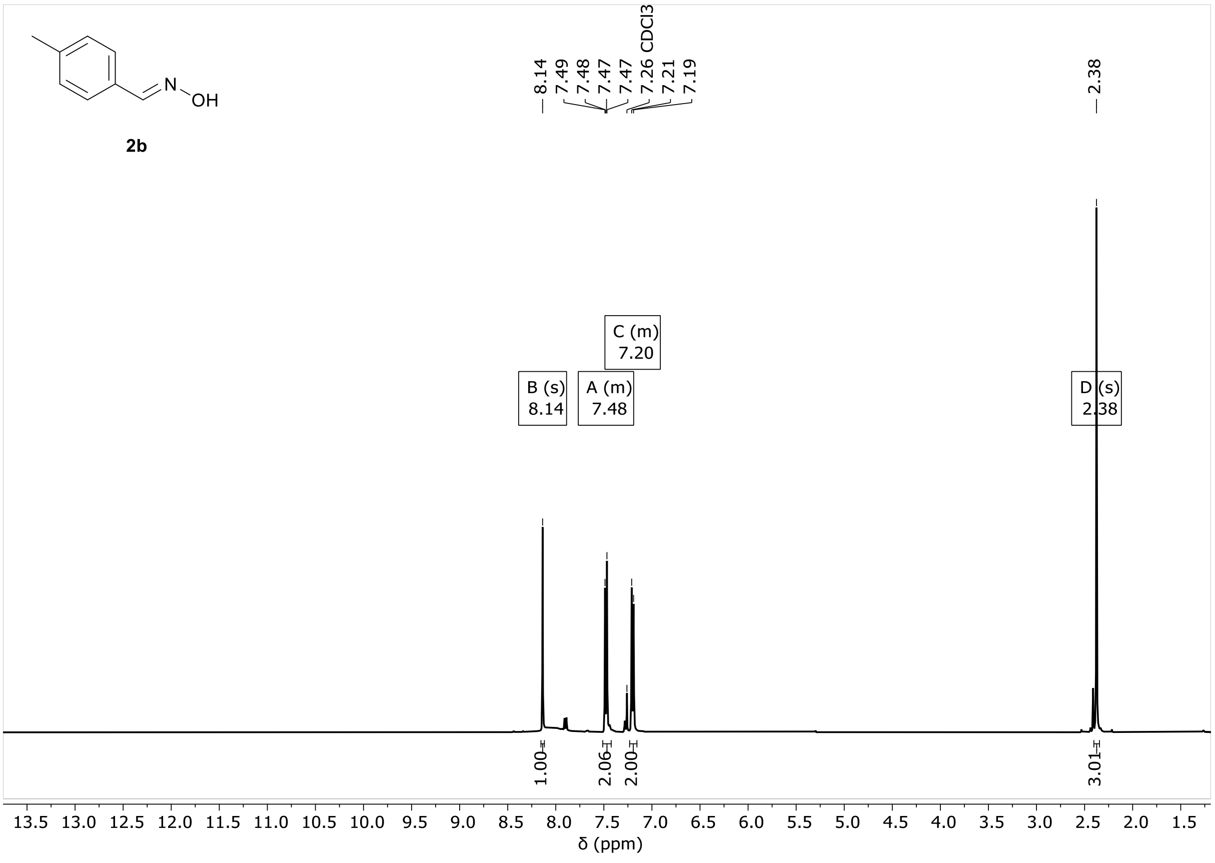

Figure S35. ^{1}H NMR spectrum of 4-methylbenzaldehyde oxime **2b** in $CDCl_3$.

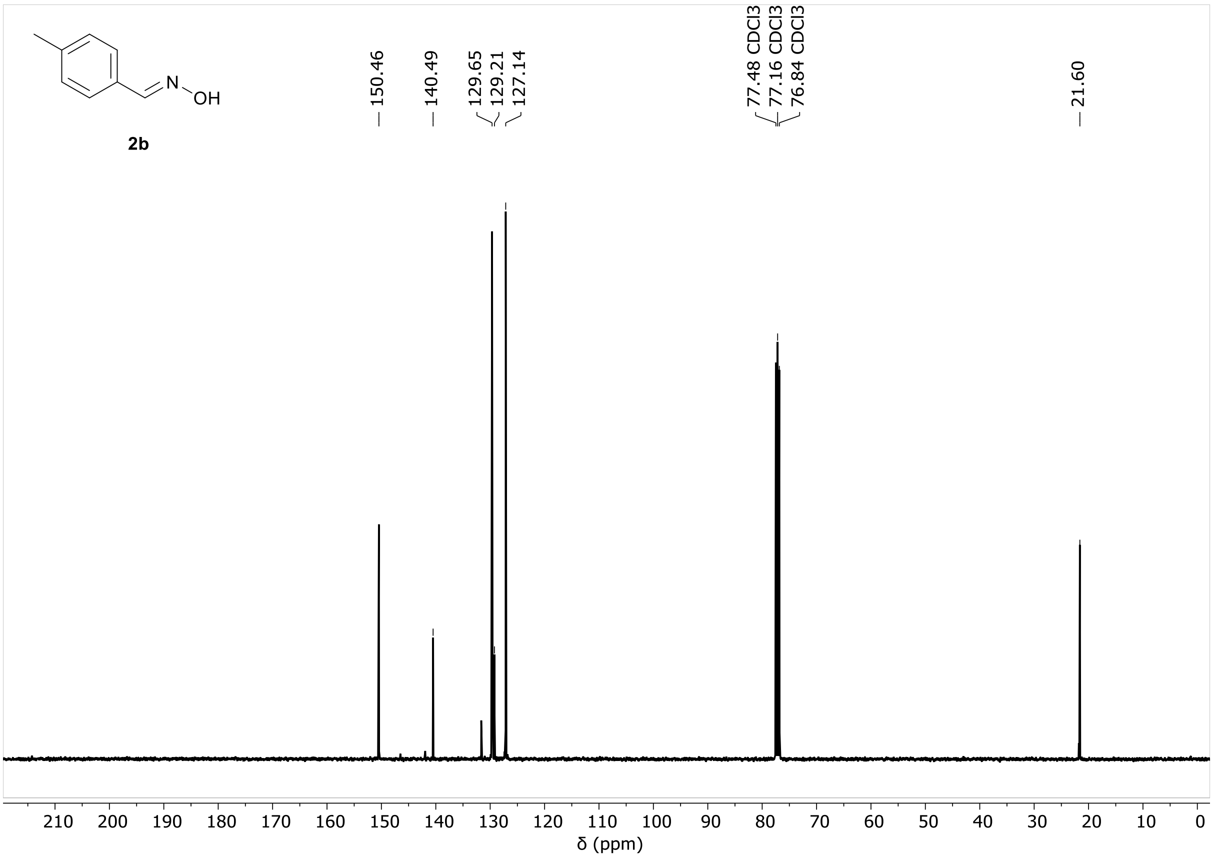

Figure S36. ^{13}C NMR spectrum of 4-methylbenzaldehyde oxime **2b** in $CDCl_3$.

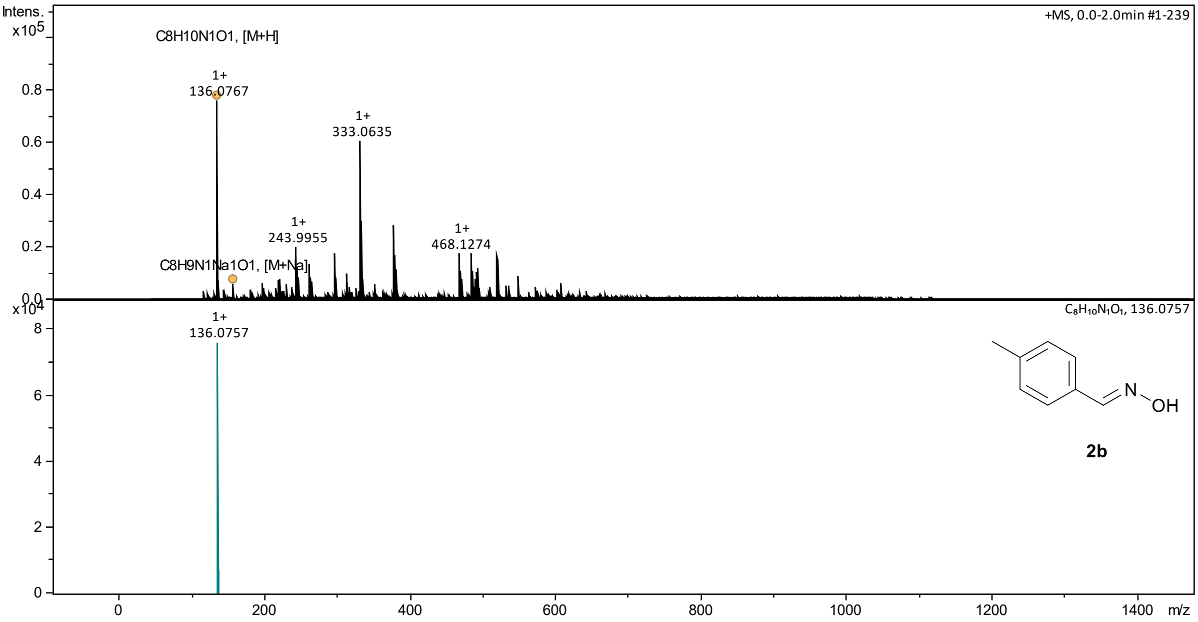

Figure S37. Deconvoluted ESI$^+$ HRMS (top) and calculated mass (bottom) spectra of 4-methylbenzaldehyde oxime **2b**.

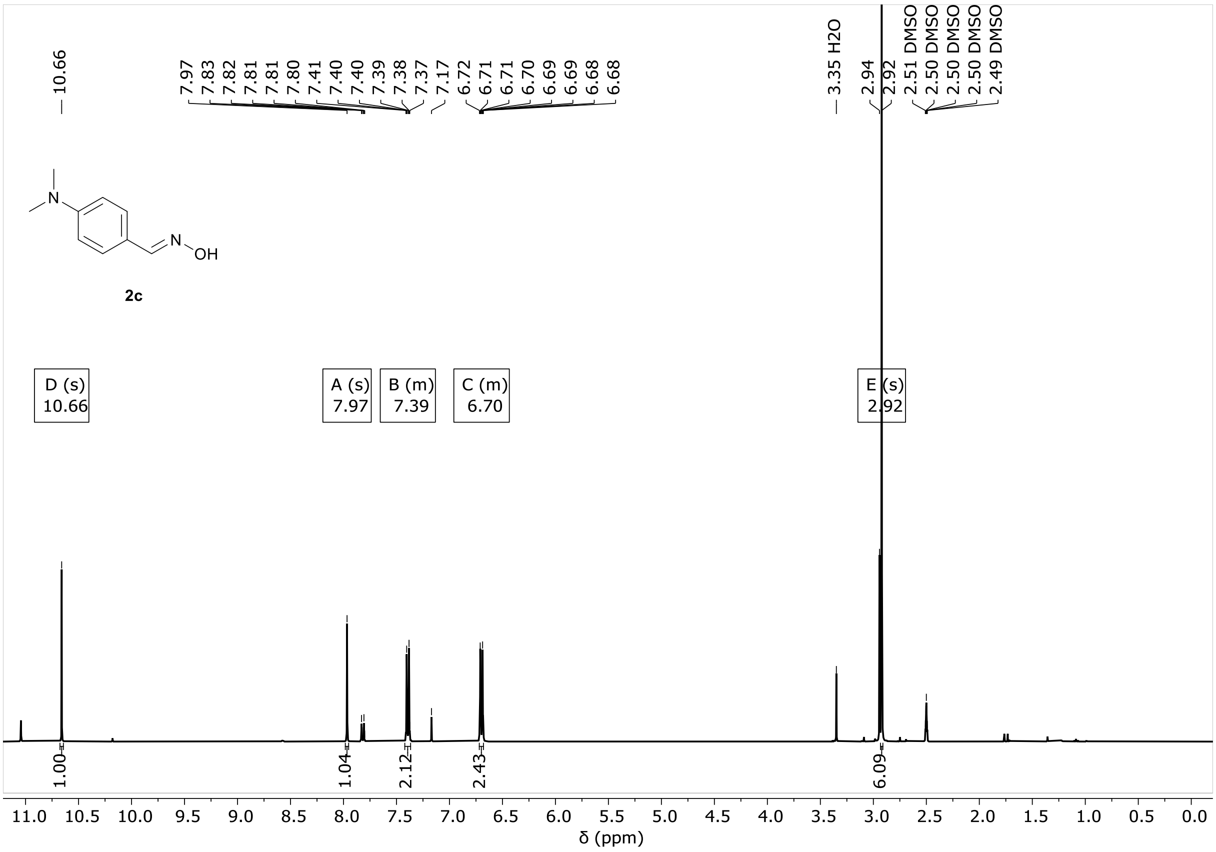

Figure S38. ^{1}H NMR spectrum of 4-(dimethylamino)benzaldehyde oxime **2c** in DMSO-d_6.

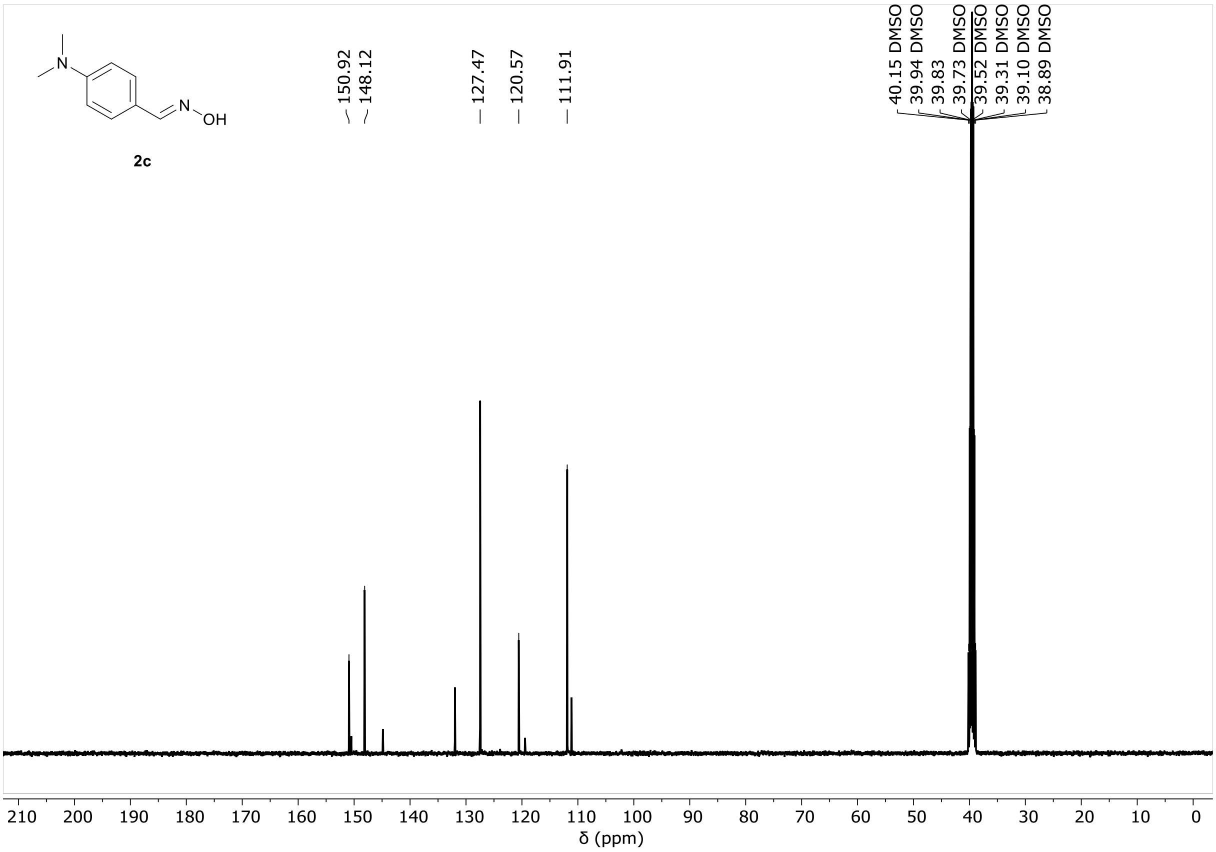

Figure S39. ^{13}C NMR spectrum of 4-(dimethylamino)benzaldehyde oxime **2c** in DMSO-d_6.

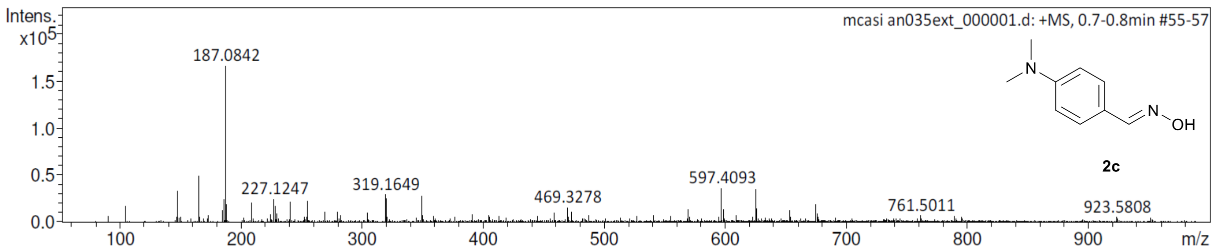

Figure S40. Deconvoluted ESI$^+$ HRMS spectrum of 4-(dimethylamino)benzaldehyde oxime **2c**.

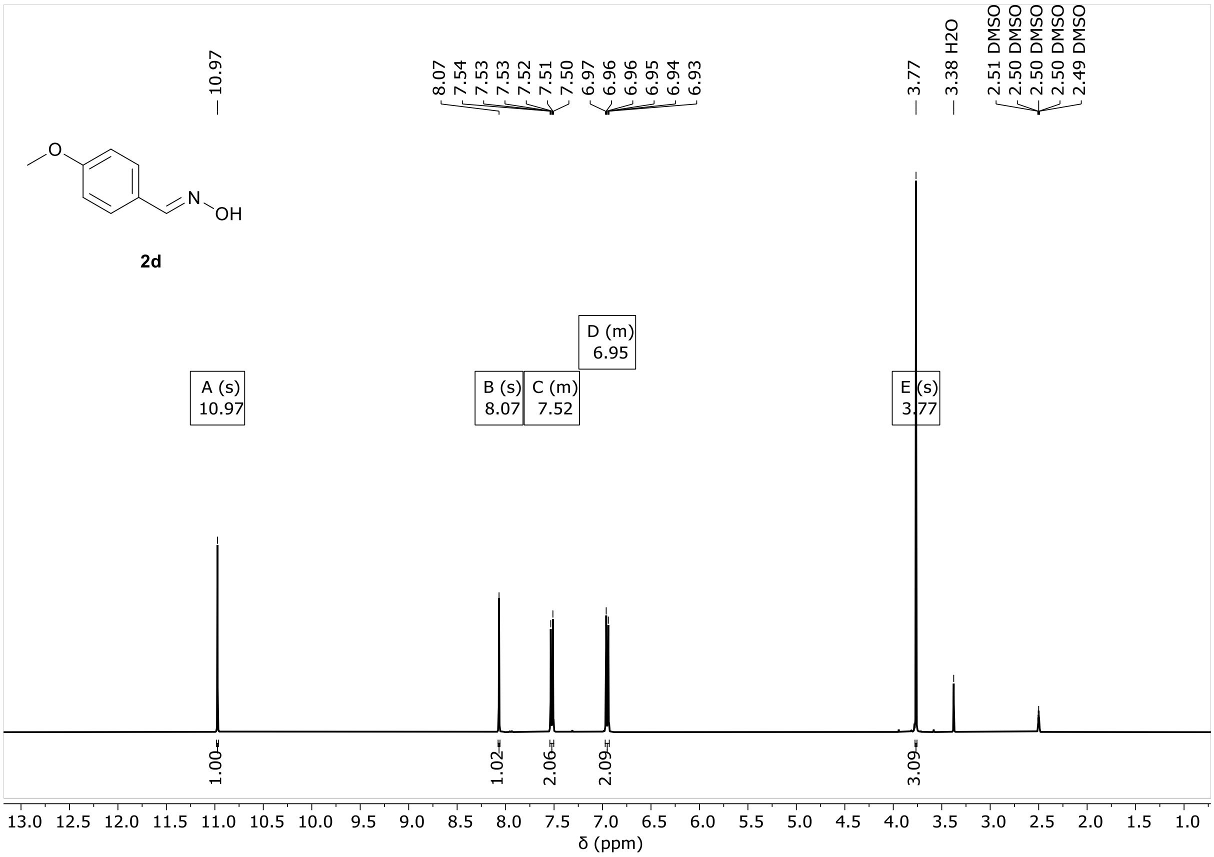

Figure S41. ^{1}H NMR spectrum of 4-methoxybenzaldehyde oxime **2d** in DMSO-d_6.

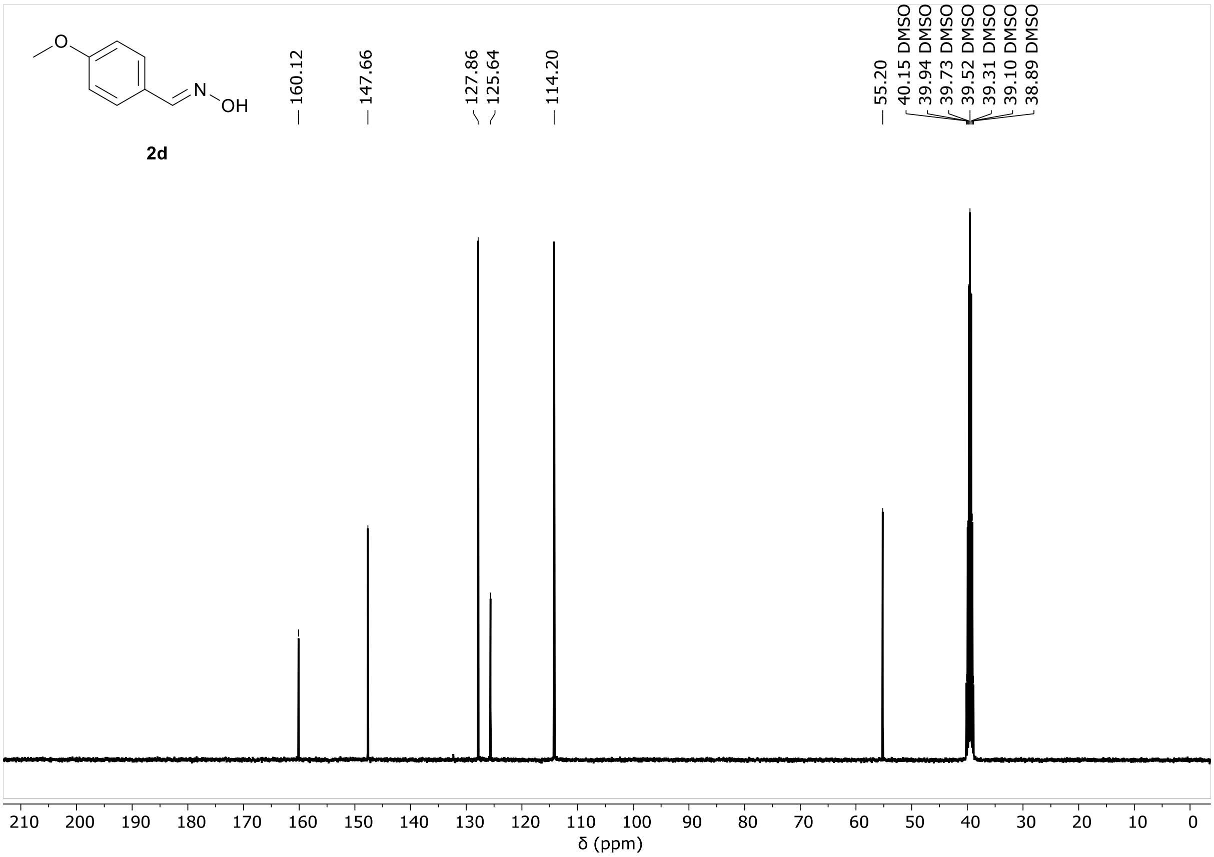

Figure S42. ^{13}C NMR spectrum of 4-methoxybenzaldehyde oxime **2d** in DMSO-d_6.

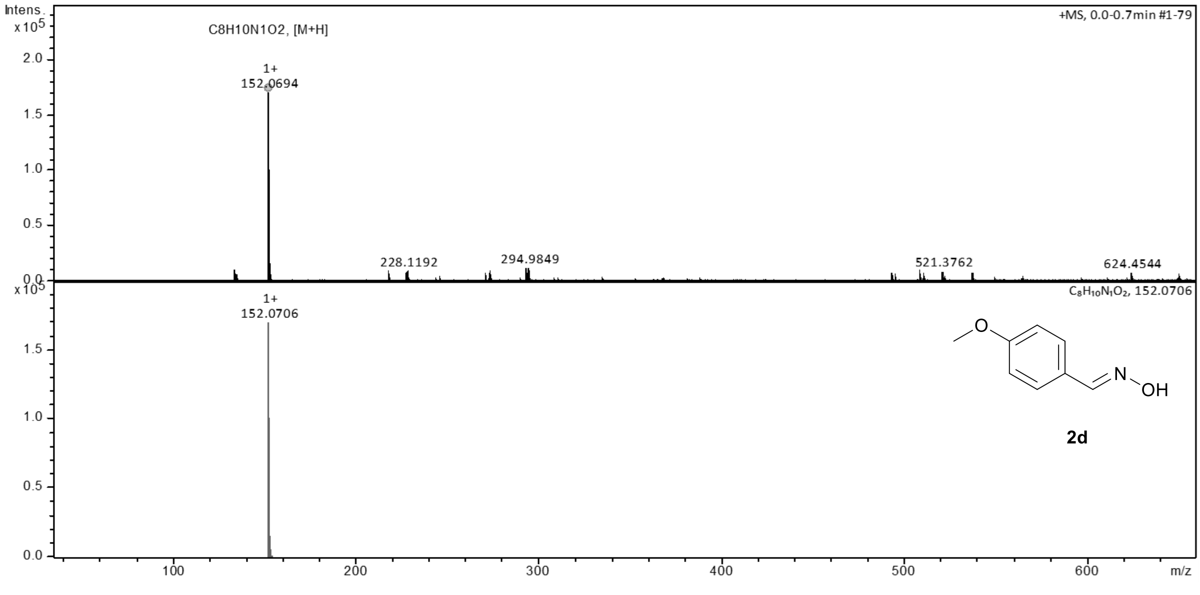

Figure S43. Deconvoluted ESI⁺ HRMS (top) and calculated mass (bottom) spectra of 4-methoxybenzaldehyde oxime **2d.**

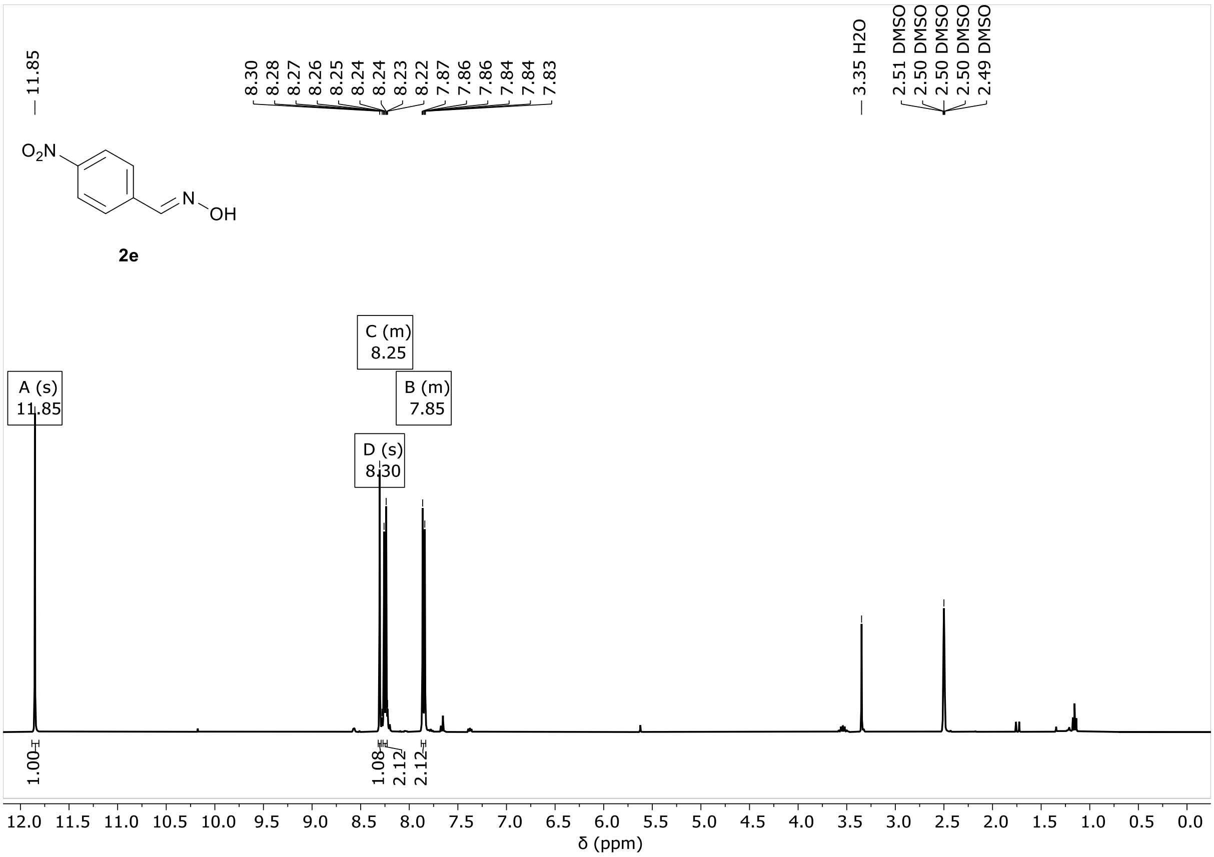

Figure S44. ^{1}H NMR spectrum of 4-nitrobenzaldehyde oxime **2e** in DMSO-d_6.

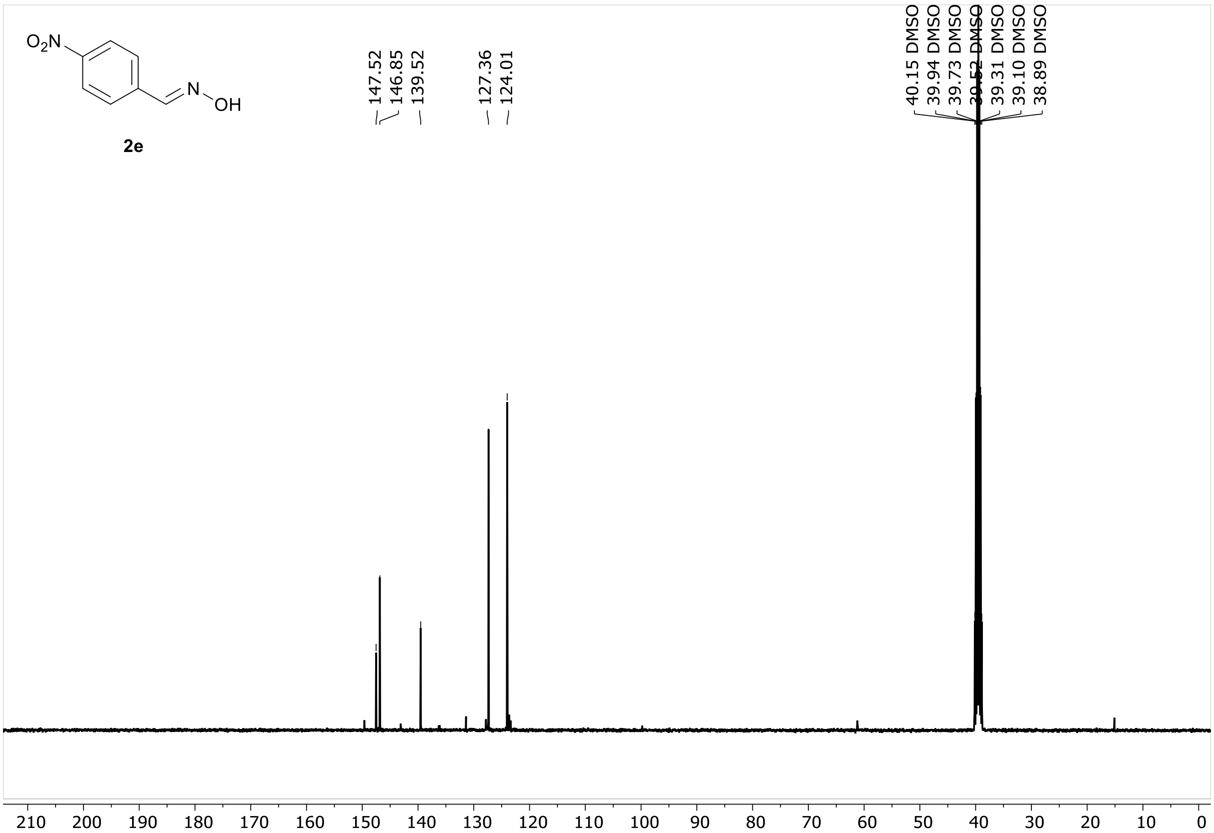

Figure S45. ^{13}C NMR spectrum of 4-nitrobenzaldehyde oxime **2e** in DMSO-d_6.

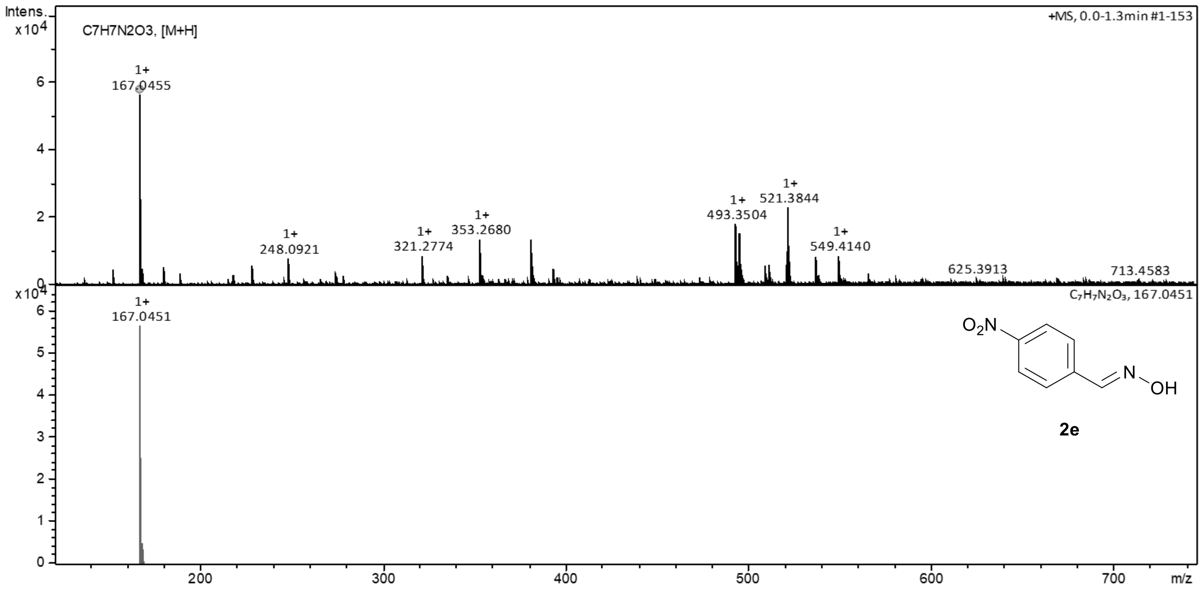

Figure S46. Deconvoluted ESI⁺ HRMS (top) and calculated mass (bottom) spectra of 4-nitrobenzaldehyde oxime **2e.**

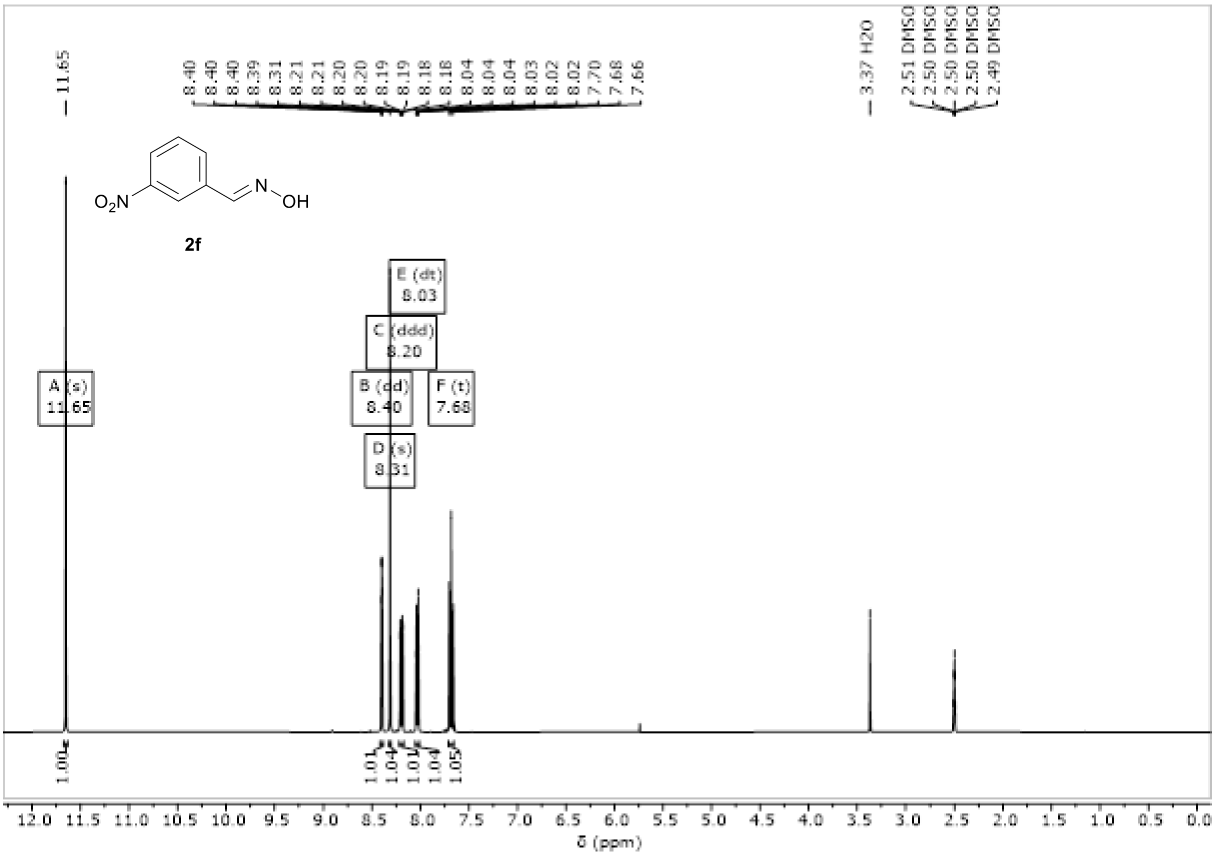

Figure S47. ^{1}H NMR spectrum of 3-nitrobenzaldehyde oxime **2f** in DMSO-d_6.

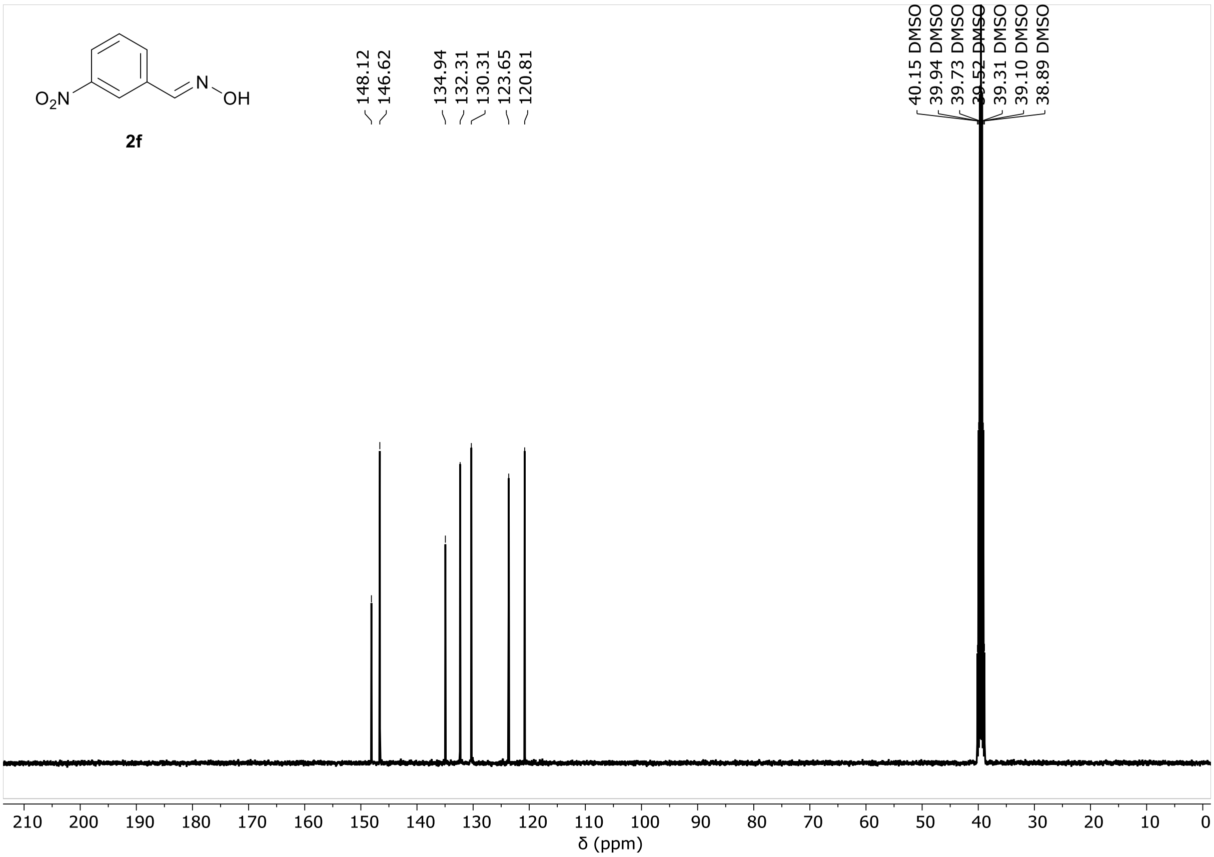

Figure S48. ^{13}C NMR spectrum of 3-nitrobenzaldehyde oxime **2f** in DMSO-d_6.

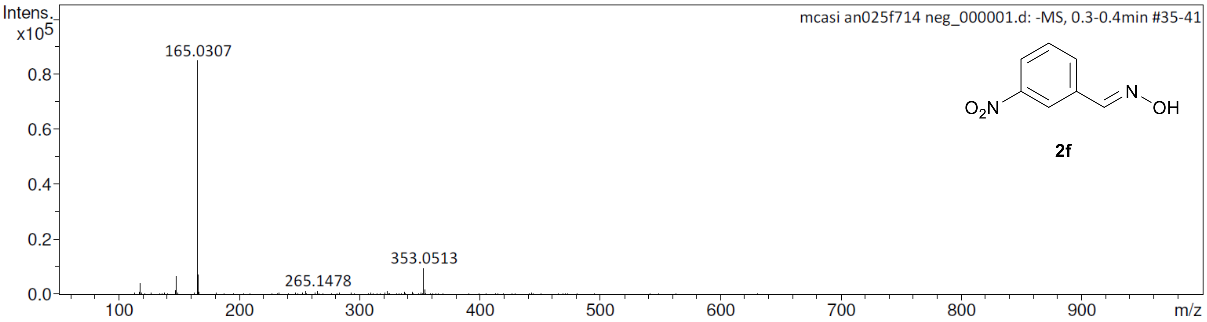

Figure S49. Deconvoluted ESI$^-$ HRMS spectrum of 3-nitrobenzaldehyde oxime **2f**.

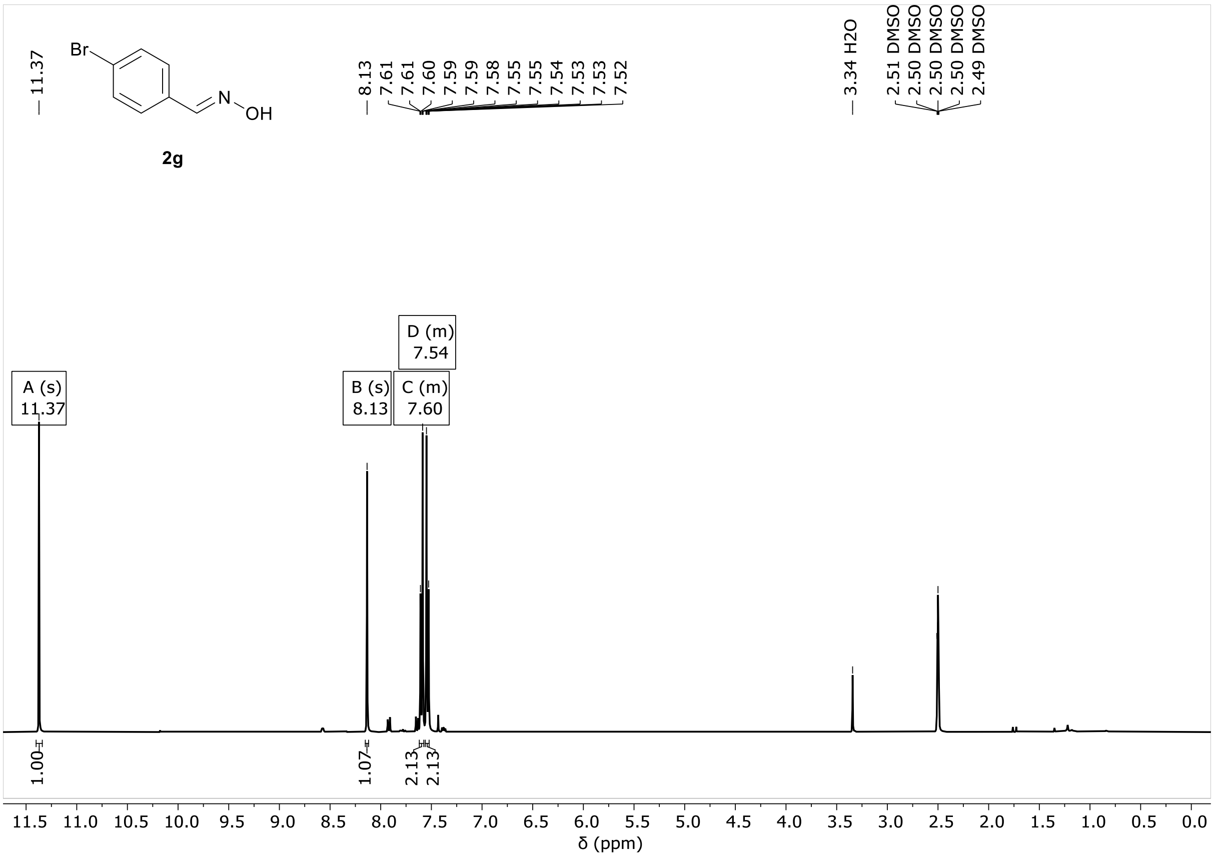

Figure S50. ^{1}H NMR spectrum of 4-bromobenzaldehyde oxime **2g** in DMSO-d_6.

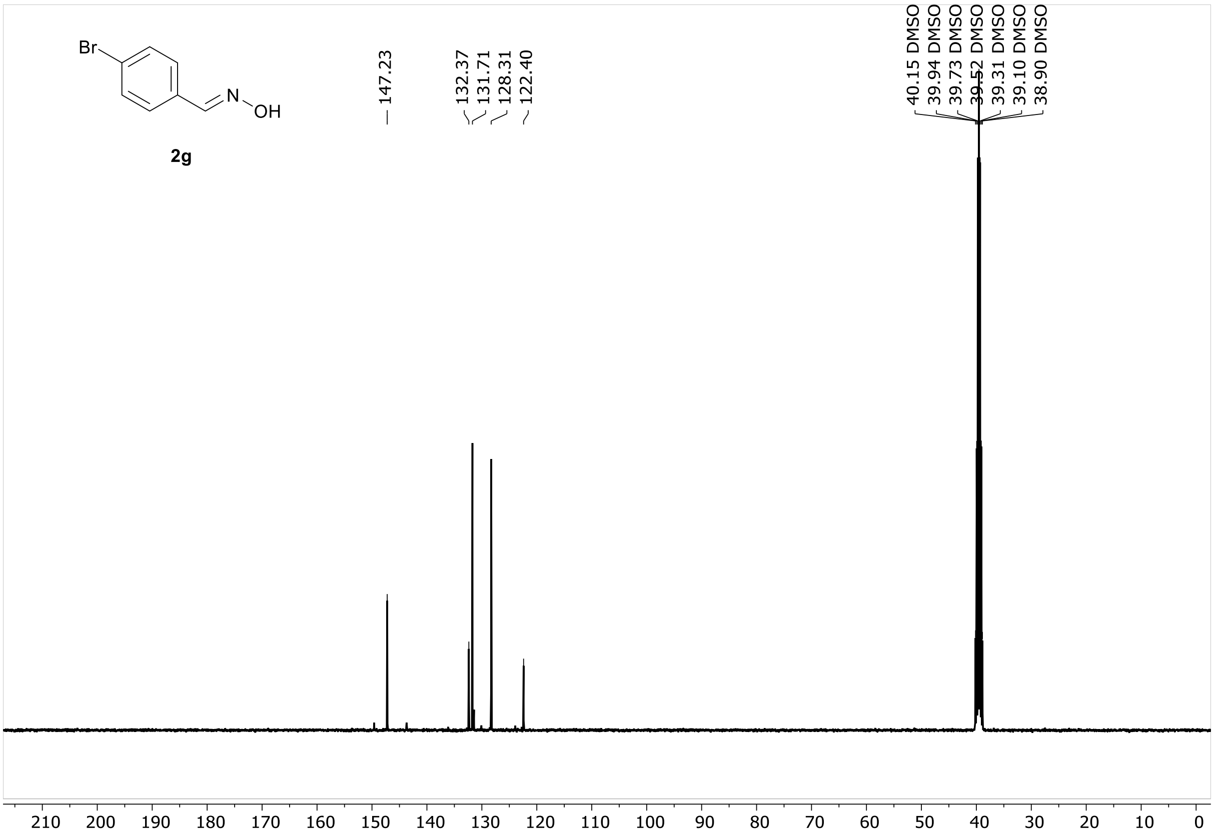

Figure S51. ^{13}C NMR spectrum of 4-bromobenzaldehyde oxime **2g** in DMSO-d_6.

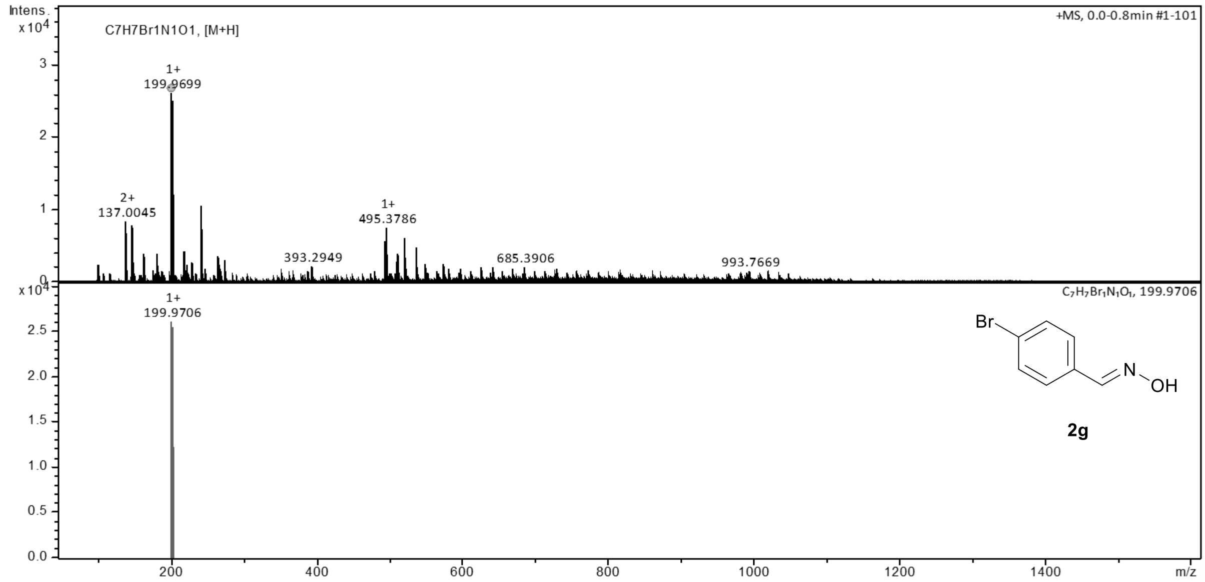

Figure S52. Deconvoluted ESI⁺ HRMS (top) and calculated mass (bottom) spectra of 4-bromobenzaldehyde oxime **2g**.

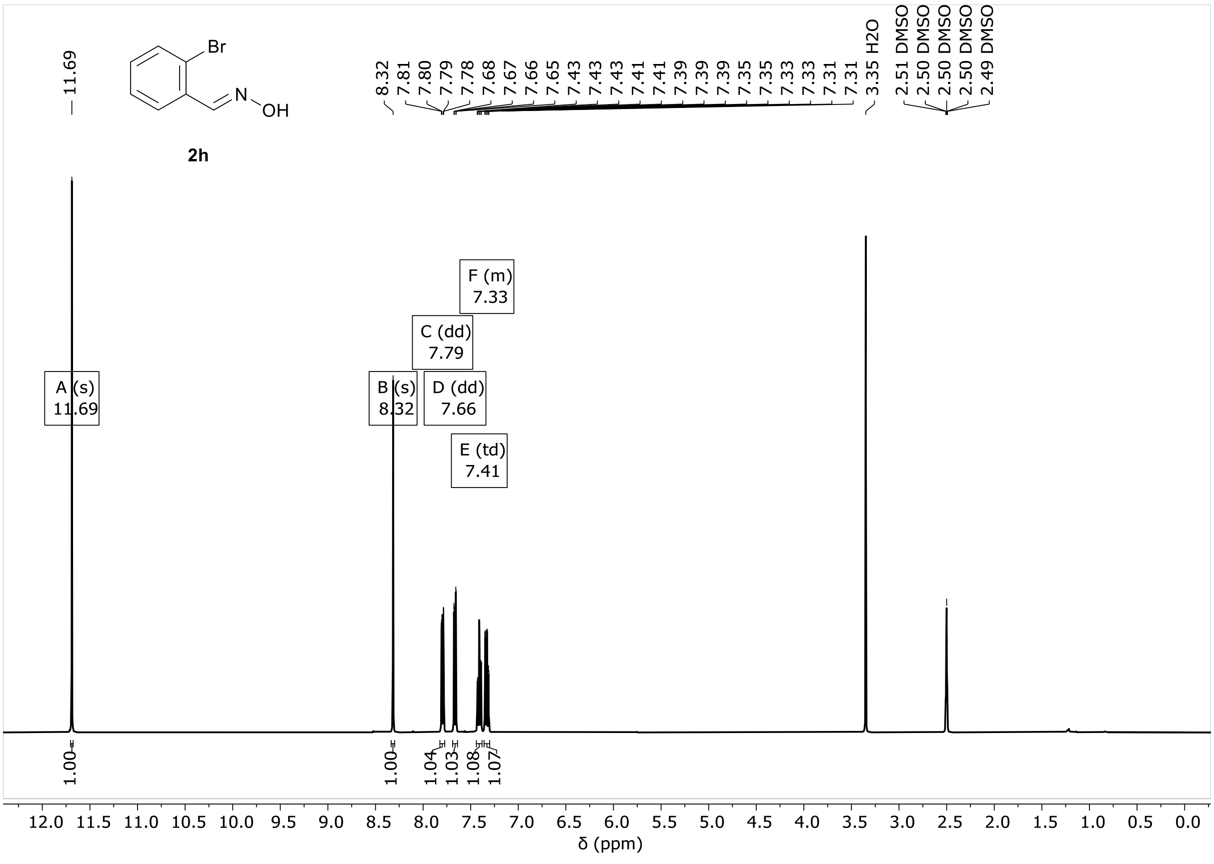

Figure S53. ^{1}H NMR spectrum of 2-bromobenzaldehyde oxime **2h** in DMSO-d_6.

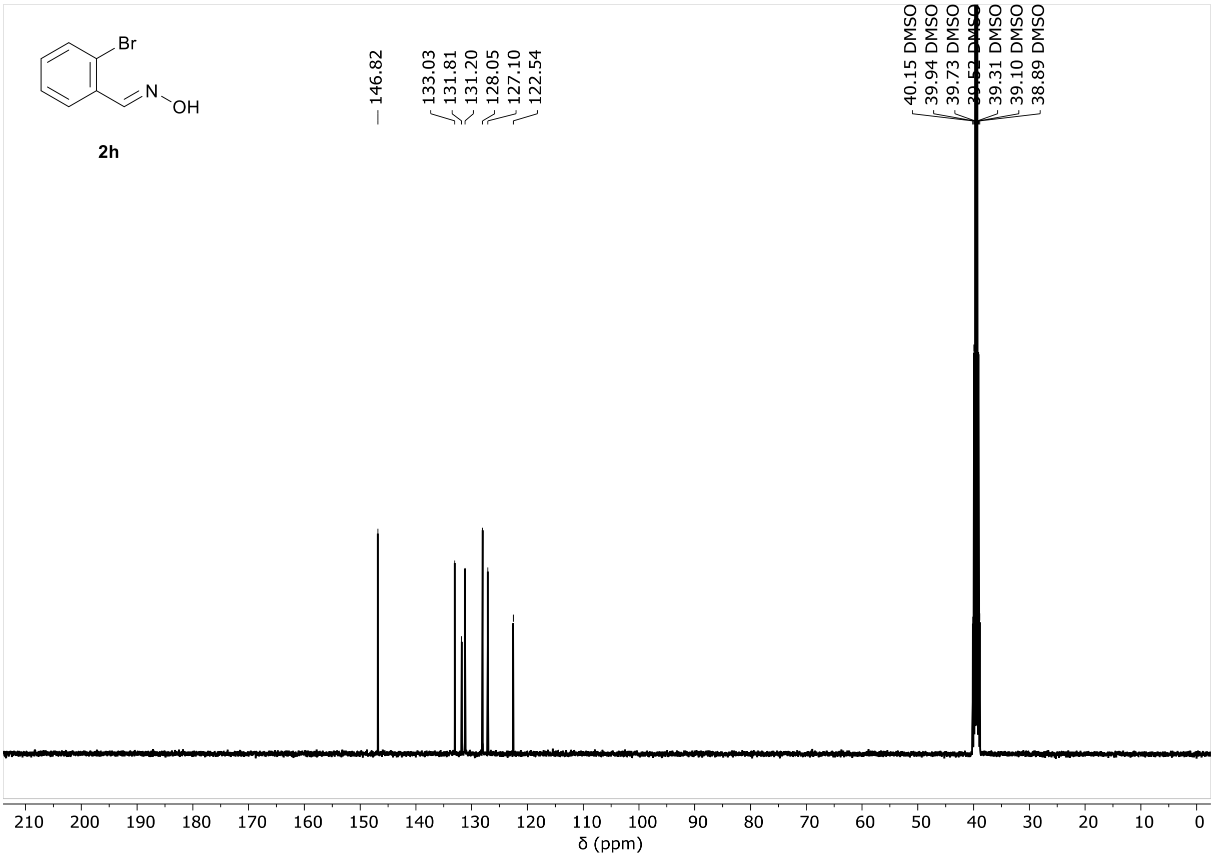

Figure S54. ^{13}C NMR spectrum of 2-bromobenzaldehyde oxime **2h** in DMSO-d_6.

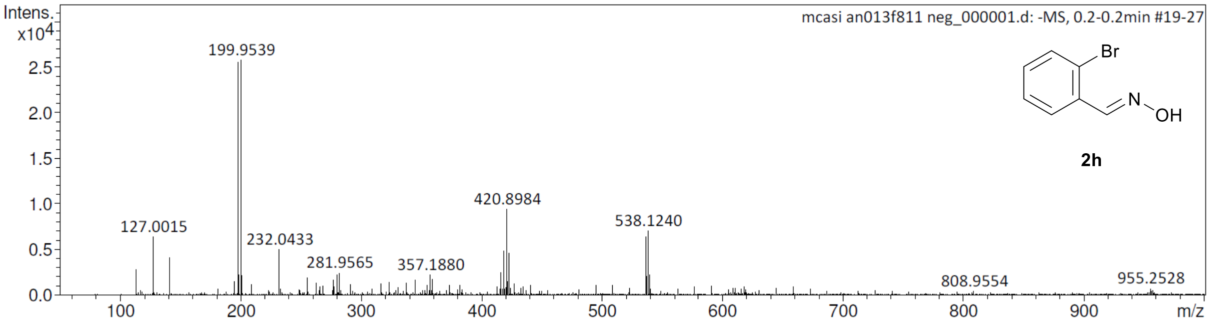

Figure S55. Deconvoluted ESI$^-$ HRMS spectrum of 2-bromobenzaldehyde oxime **2h**.

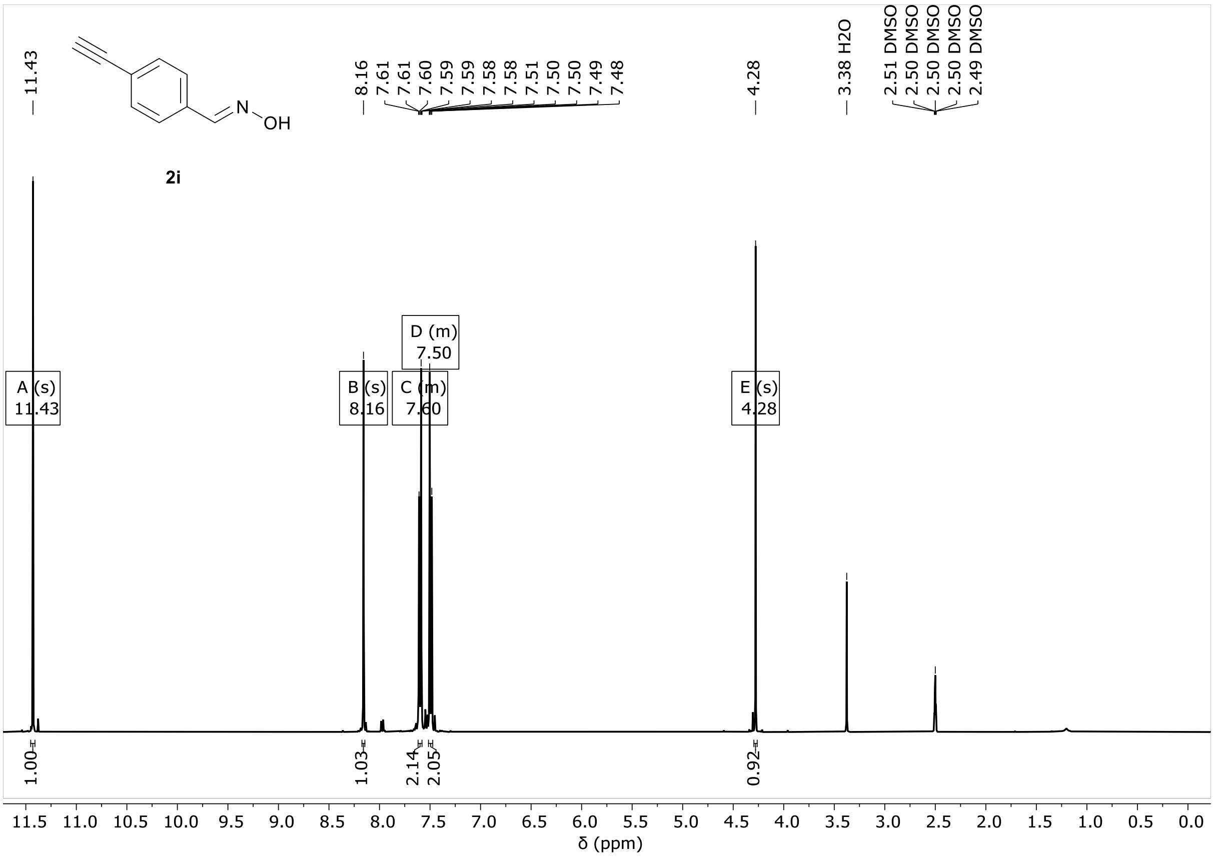

Figure S56. ^{1}H NMR spectrum of 4-ethynylbenzaldehyde oxime **2i** in DMSO-d_6.

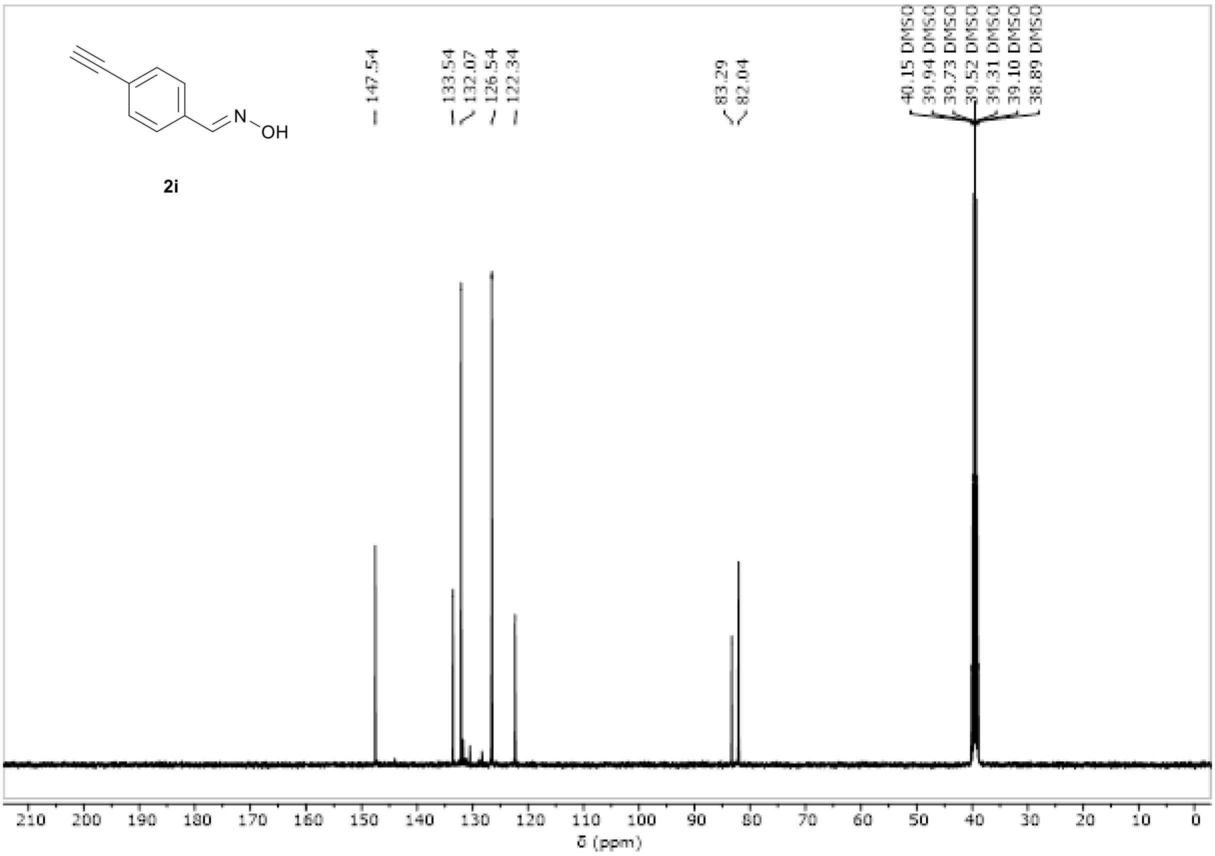

Figure S57. ^{13}C NMR spectrum of 4-ethynylbenzaldehyde oxime **2i** in DMSO-d_6.

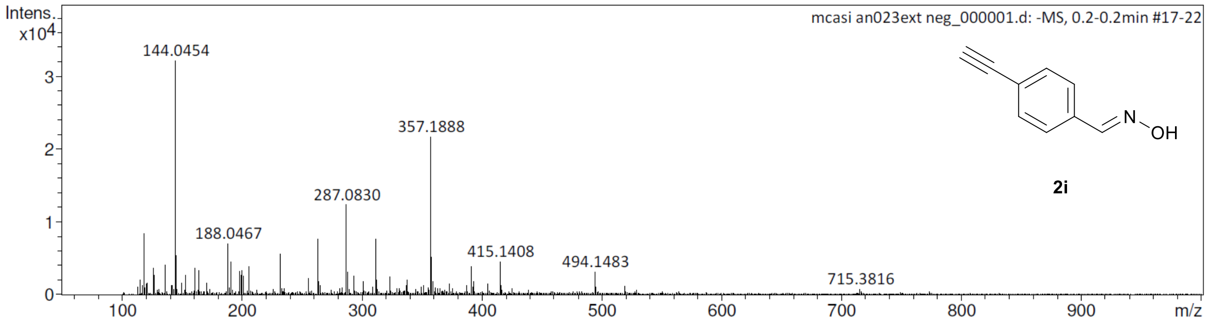

Figure S58. Deconvoluted ESI⁻ HRMS spectrum of 4-ethynylbenzaldehyde oxime **2i**.

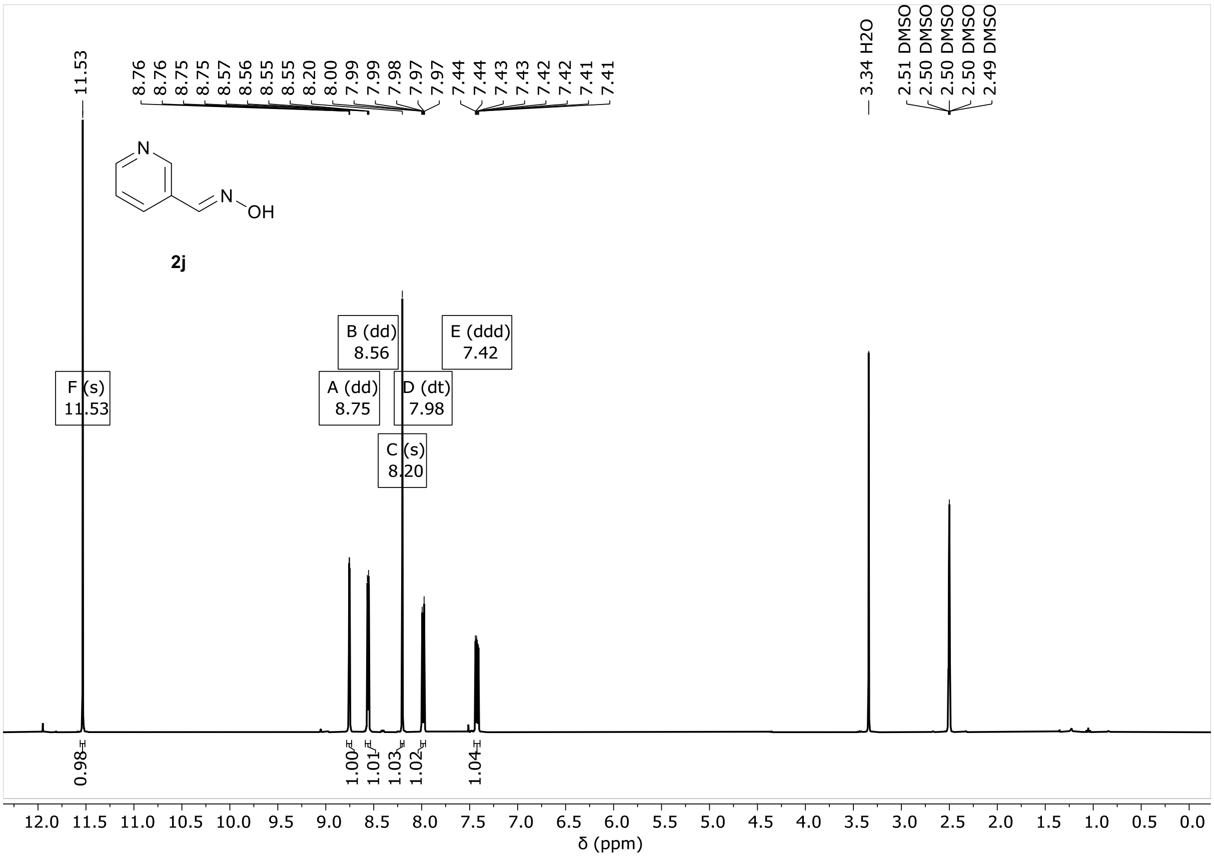

Figure S59. ^{1}H NMR spectrum of nicotinaldehyde oxime **2j** in DMSO-d_6.

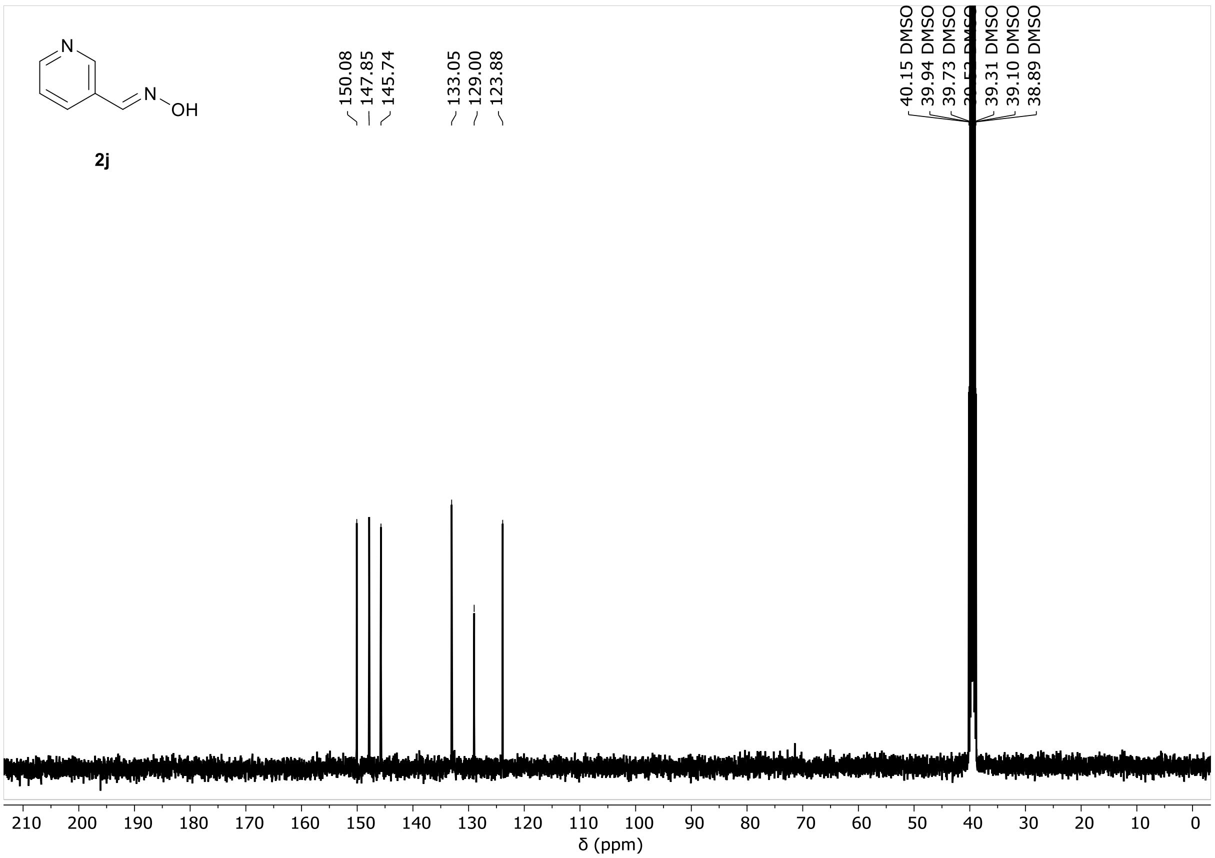

Figure S60. ^{13}C NMR spectrum of nicotinaldehyde oxime **2j** in DMSO-d_6.

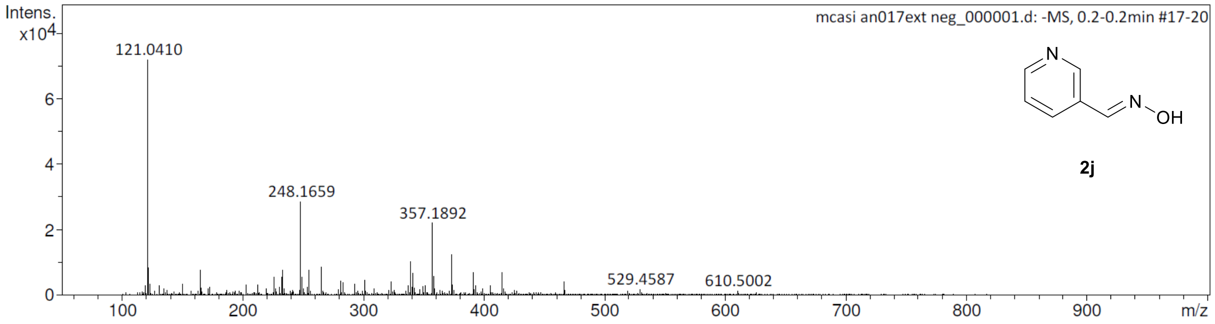

Figure S61. Deconvoluted ESI⁻ HRMS spectrum of nicotinaldehyde oxime **2j**.

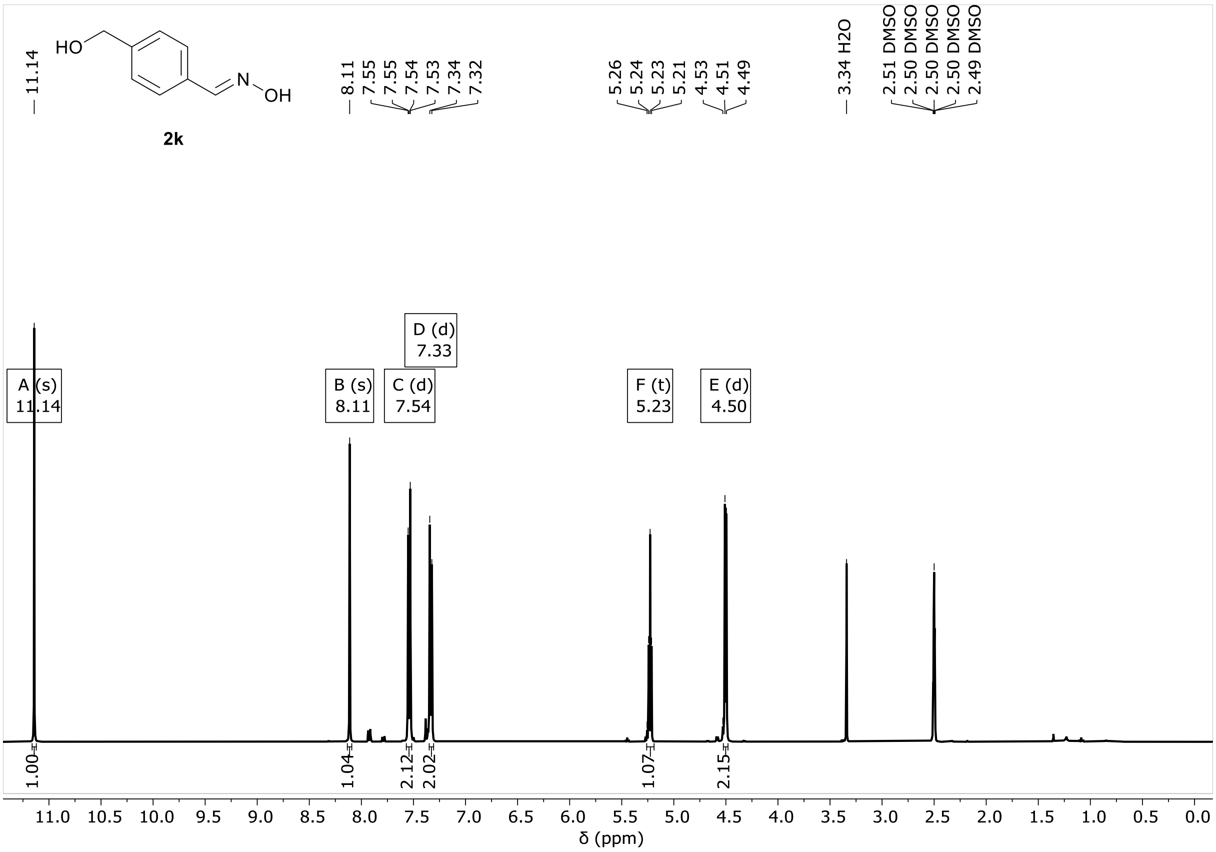

Figure S62. ^{1}H NMR spectrum of 4-(hydroxymethyl)benzaldehyde oxime **2k** in DMSO-d_6.

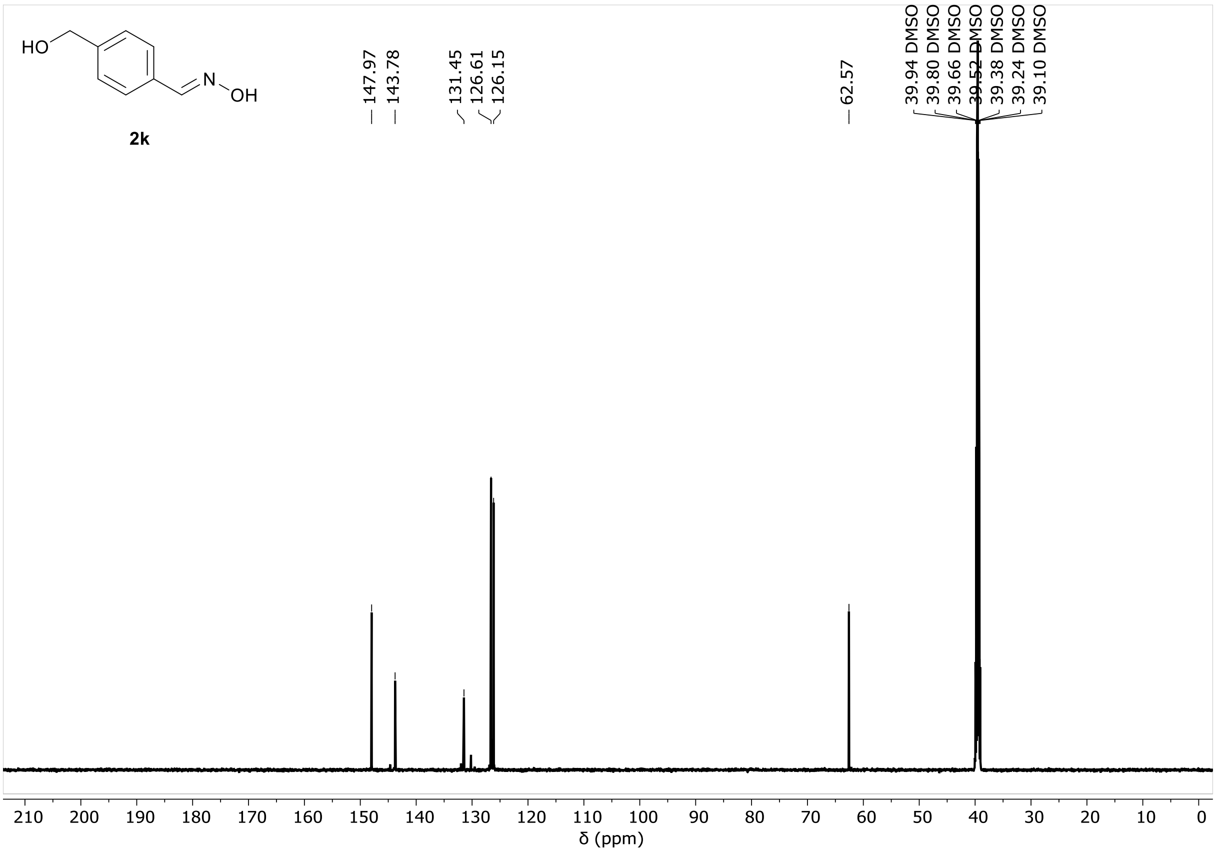

Figure S63. ^{13}C NMR spectrum of 4-(hydroxymethyl)benzaldehyde oxime **2k** in DMSO-d_6.

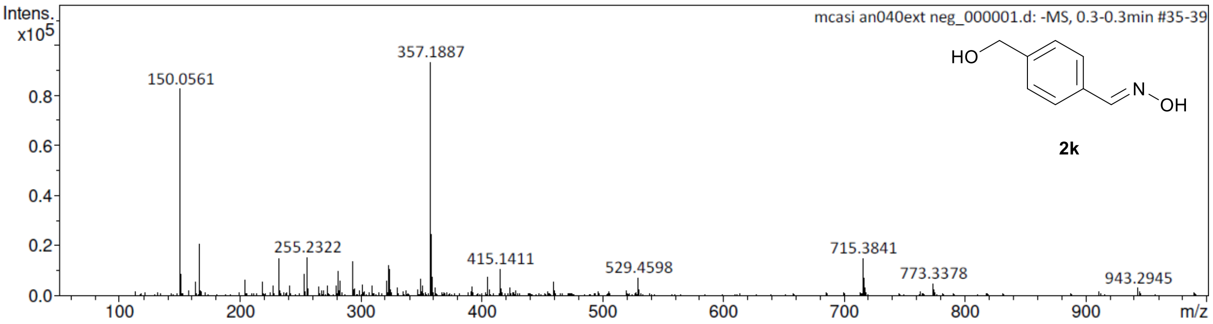

Figure S64. Deconvoluted ESI$^-$ HRMS spectrum of 4-(hydroxymethyl)benzaldehyde oxime **2k** in DMSO-d_6.

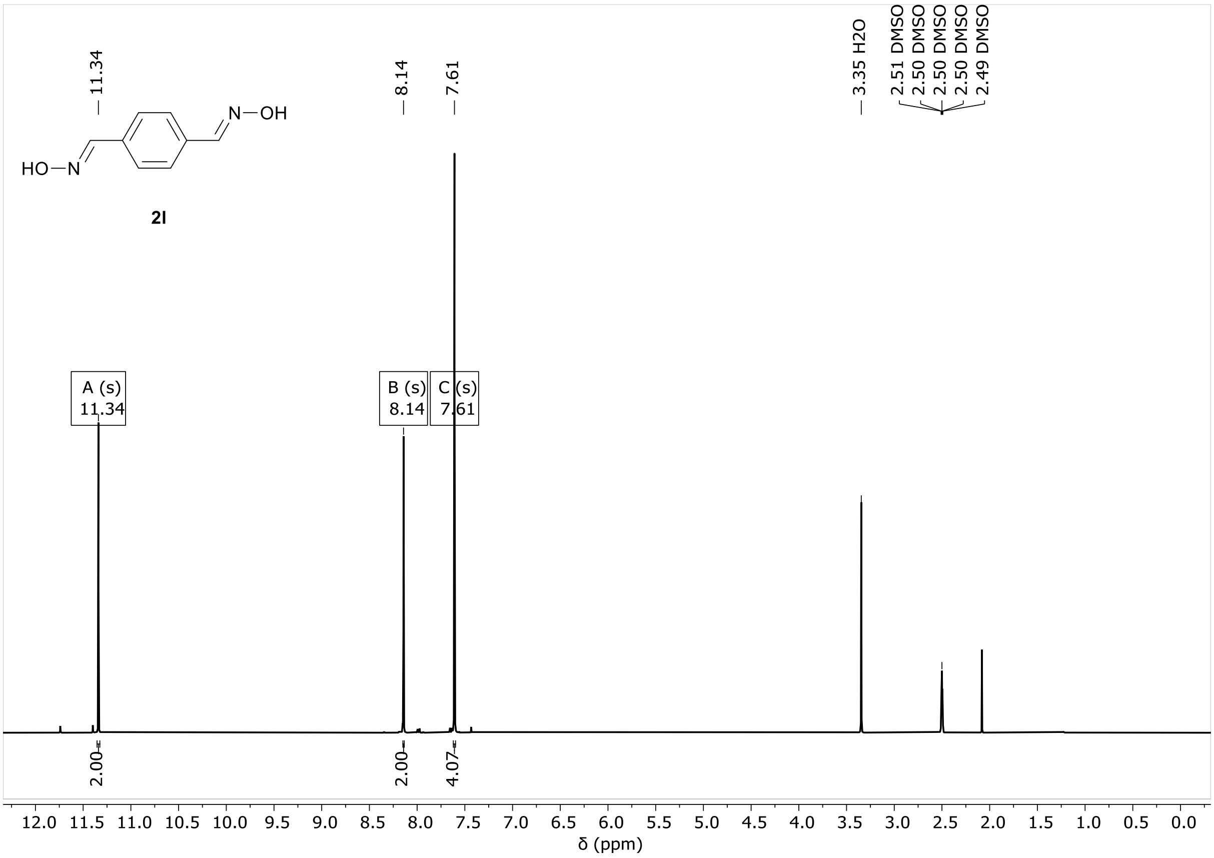

Figure S65. ^{1}H NMR spectrum of terephthalaldehyde dioxime **2l** in DMSO-d_6.

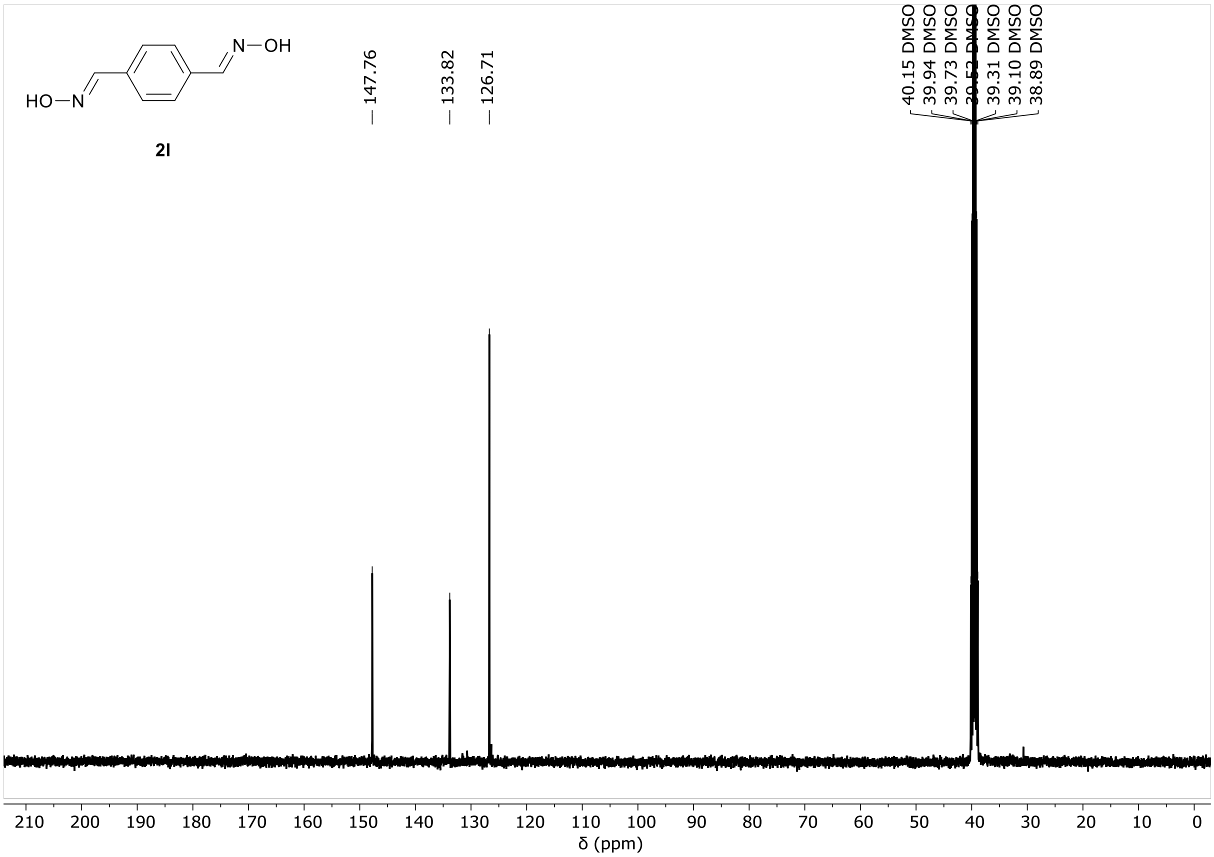

Figure S66. ^{13}C NMR spectrum of terephthalaldehyde dioxime **2l** in DMSO-d_6.

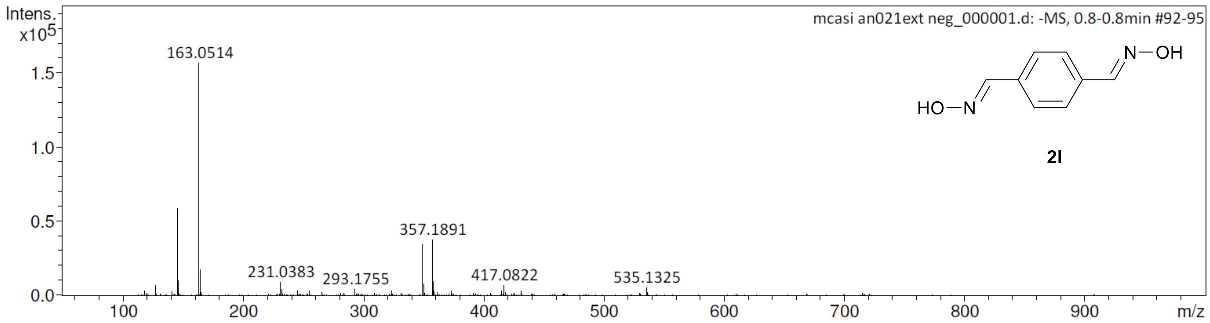

Figure S67. Deconvoluted ESI$^-$ HRMS spectrum of terephthalaldehyde dioxime **2l**.

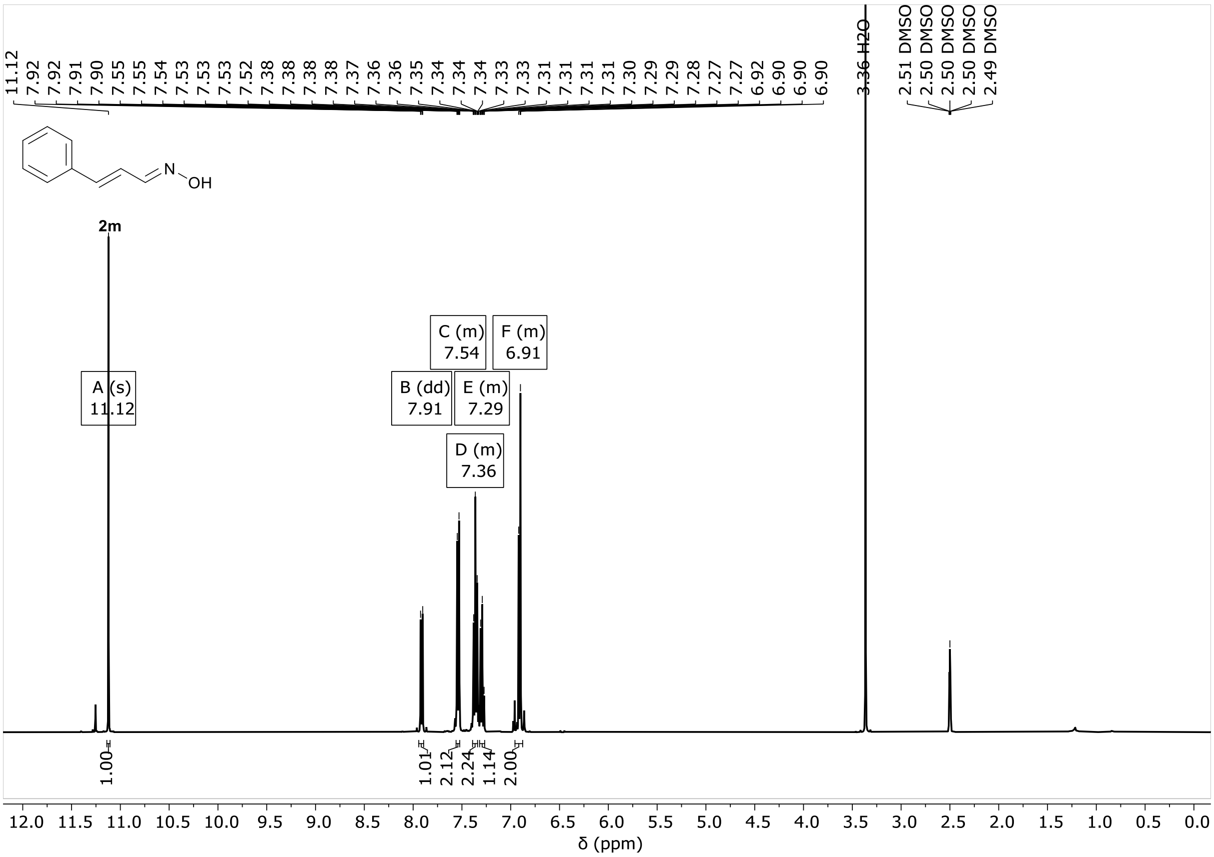

Figure S68. ^{1}H NMR spectrum of cinnamaldehyde oxime **2m** in DMSO-d_6.

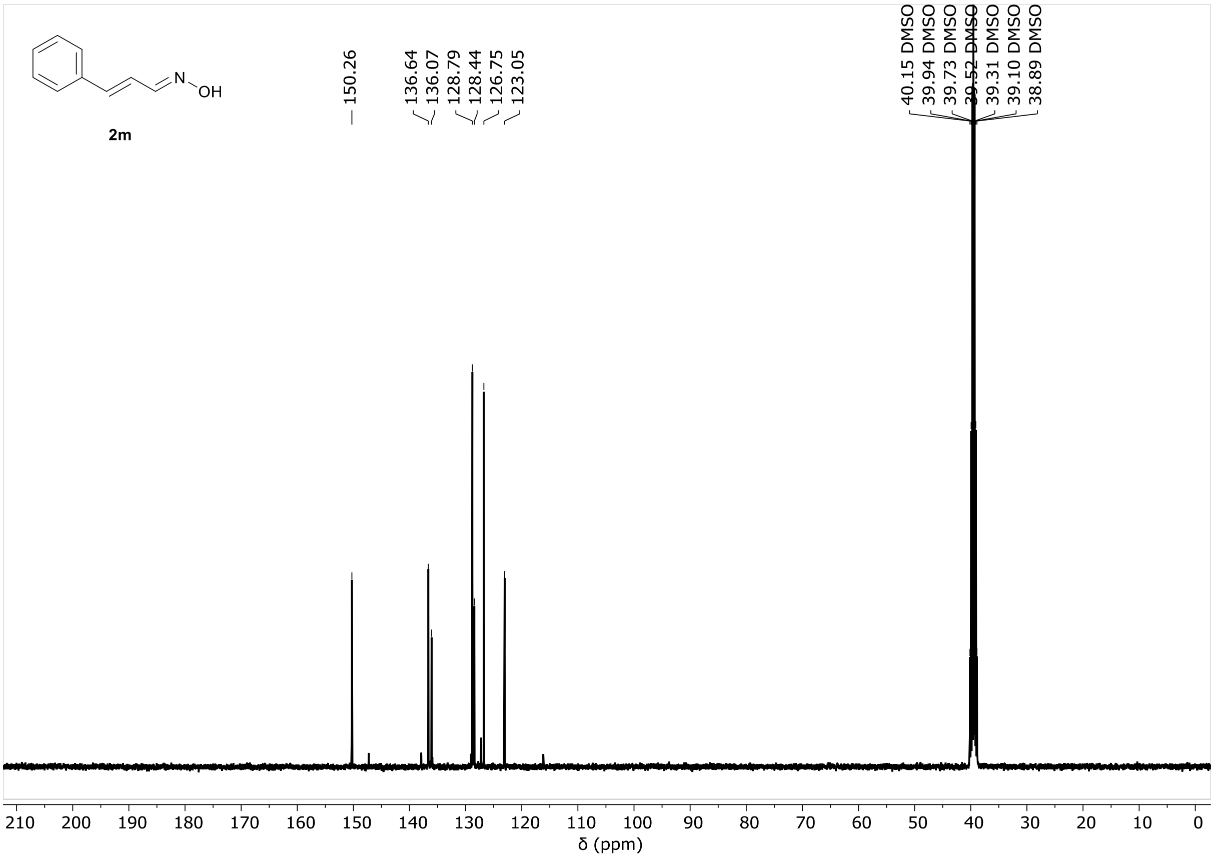

Figure S69. ^{13}C NMR spectrum of cinnamaldehyde oxime **2m** in DMSO-d_6.

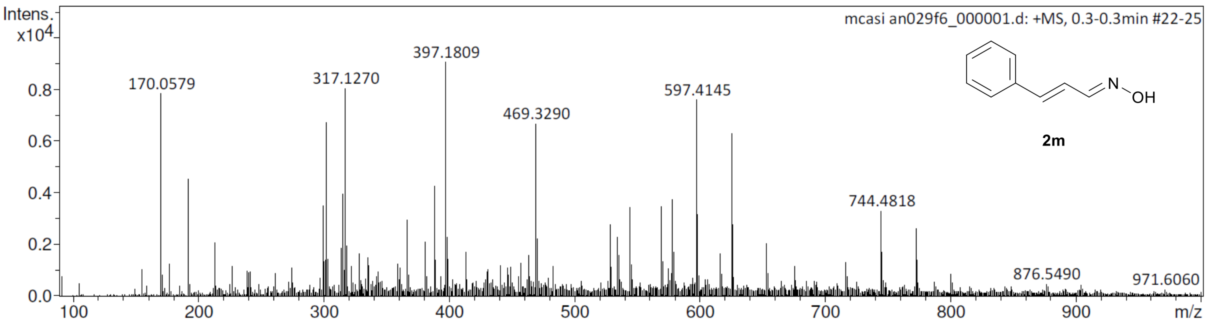

Figure S70. Deconvoluted ESI⁺ HRMS spectrum of cinnamaldehyde oxime **2m.**

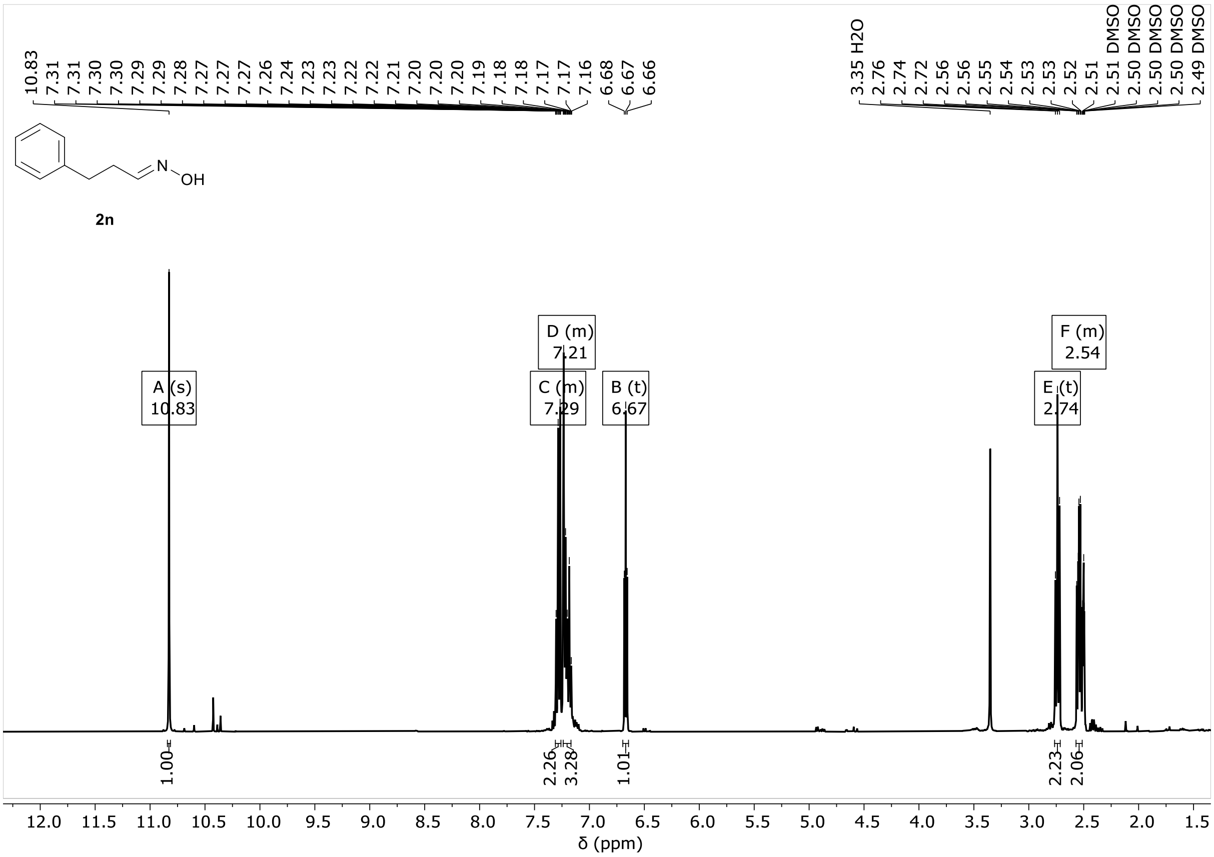

Figure S71. ^{1}H NMR spectrum of 3-phenylpropanal oxime **2n** in DMSO-d_6.

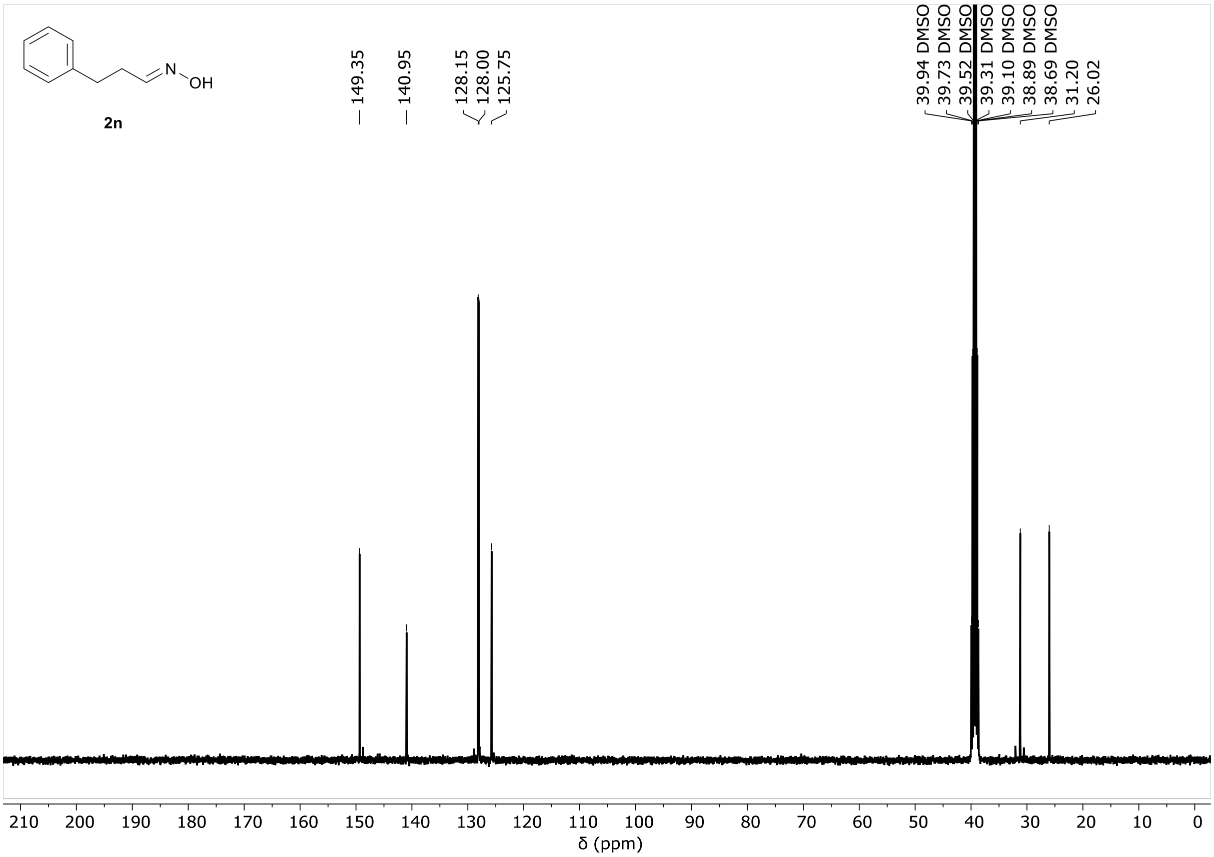

Figure S72. ^{13}C NMR spectrum of 3-phenylpropanal oxime **2n** in DMSO-d_6.

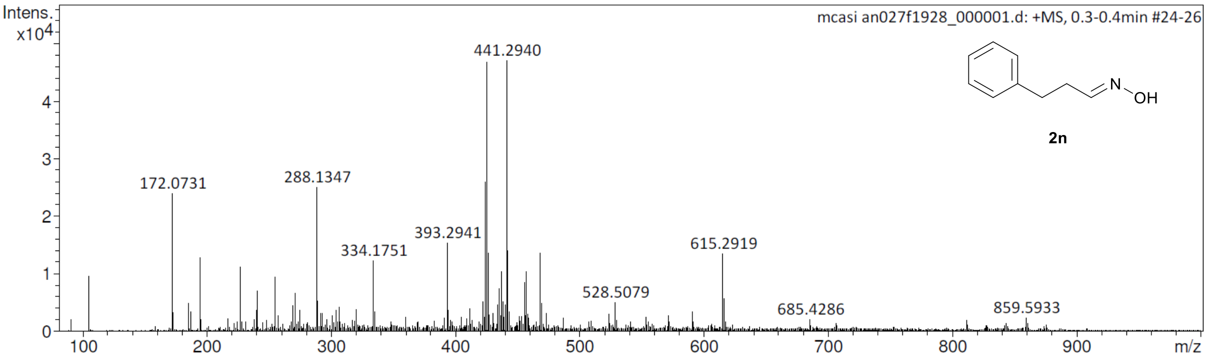

Figure S73. Deconvoluted ESI⁺ HRMS spectrum of 3-phenylpropanal oxime **2n**.

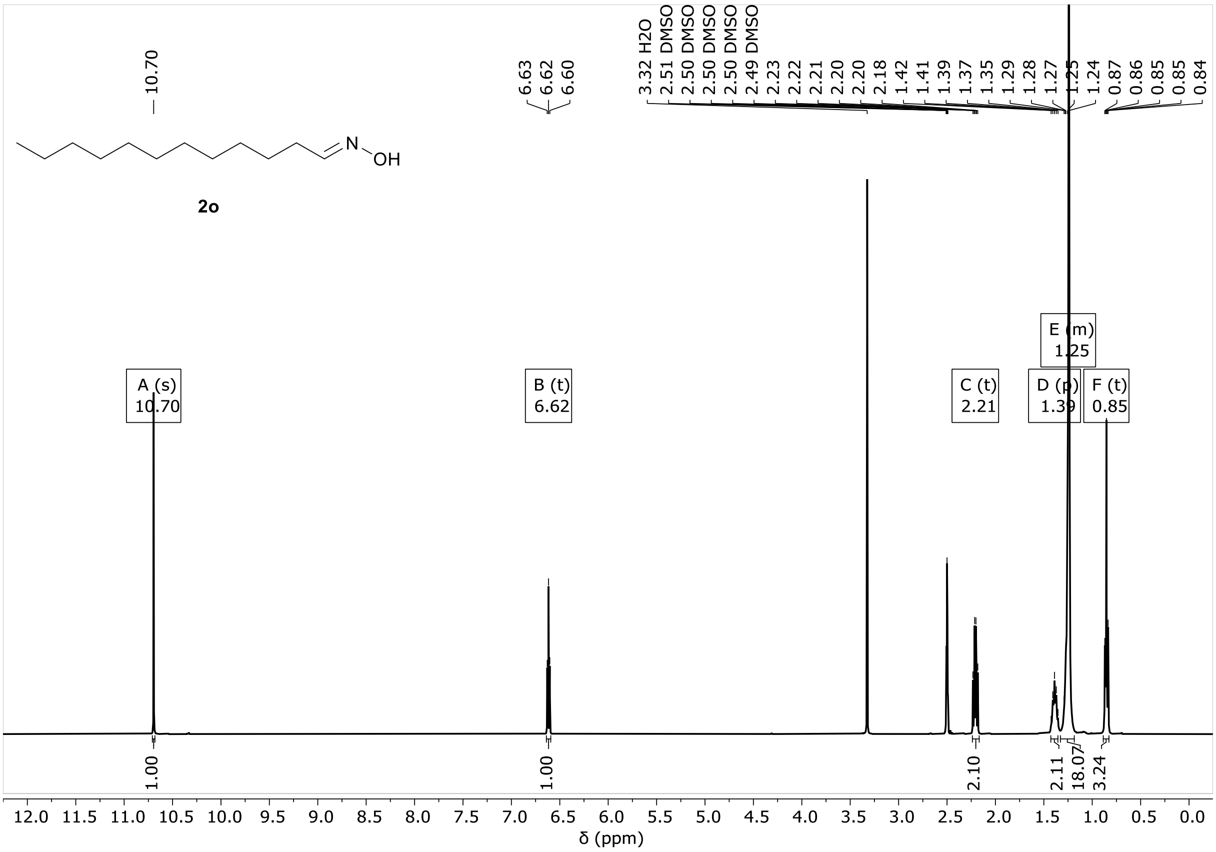

Figure S74. ^{1}H NMR spectrum of dodecanal oxime **2o** in DMSO-d_6.

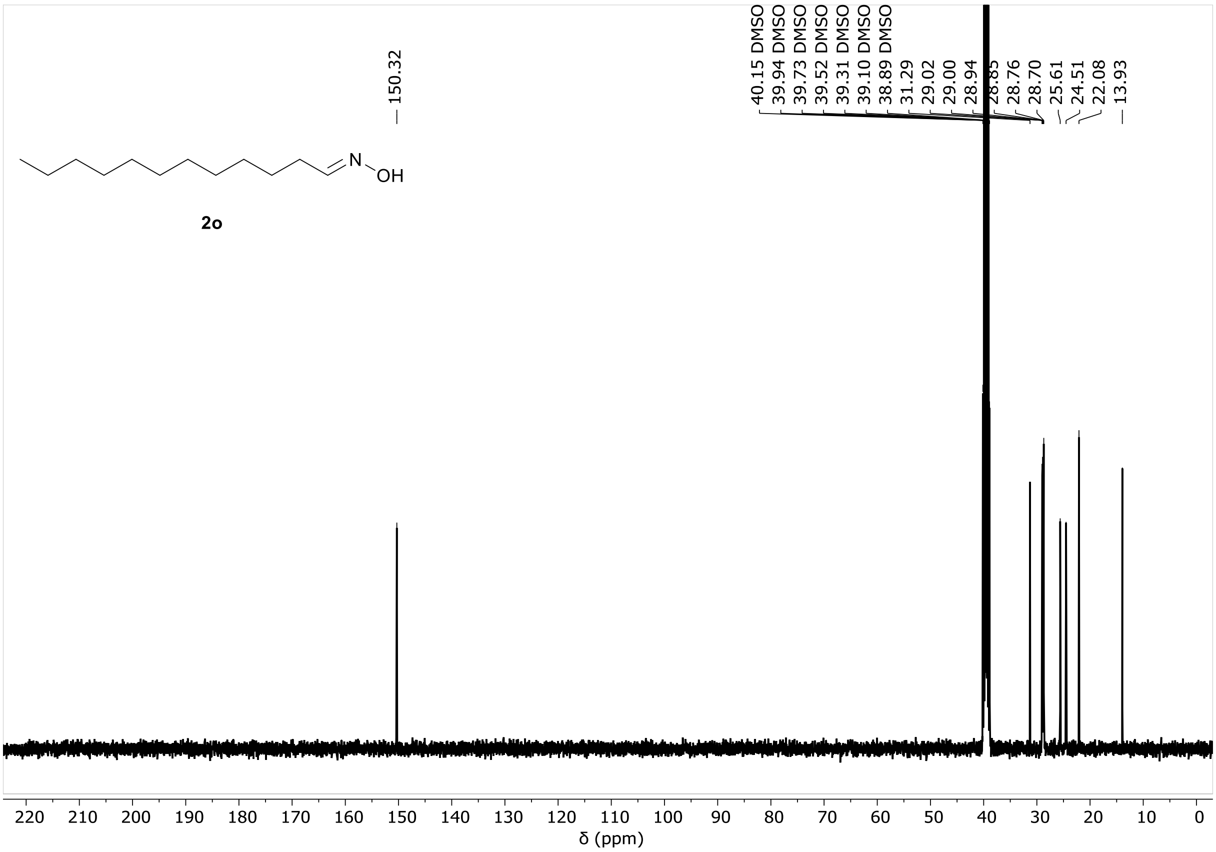

Figure S75. ^{13}C NMR spectrum of dodecanal oxime **2o** in DMSO-d_6.

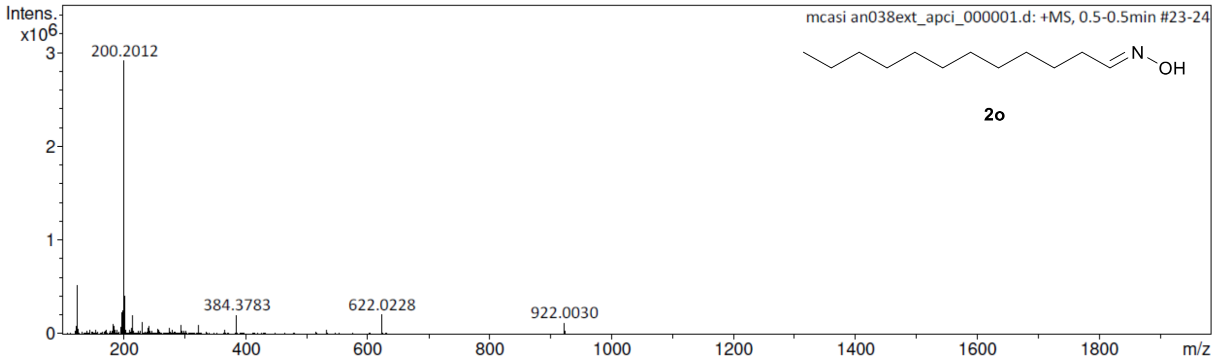

Figure S76. Deconvoluted APCI⁺ HRMS spectrum of dodecanal oxime **2o.**

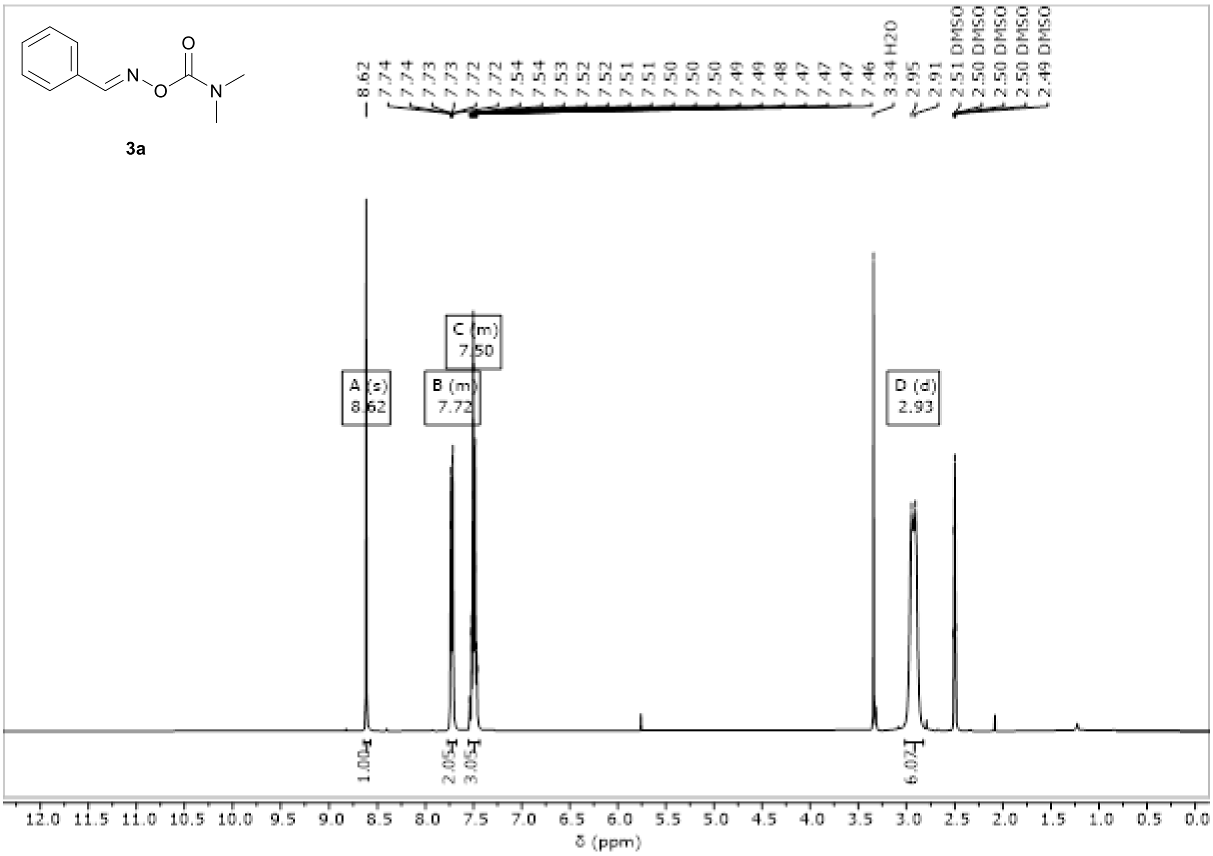

Figure S77. ^{1}H NMR spectrum of benzaldehyde *O*-dimethylcarbamoyloxime **3a** in DMSO-d_6.

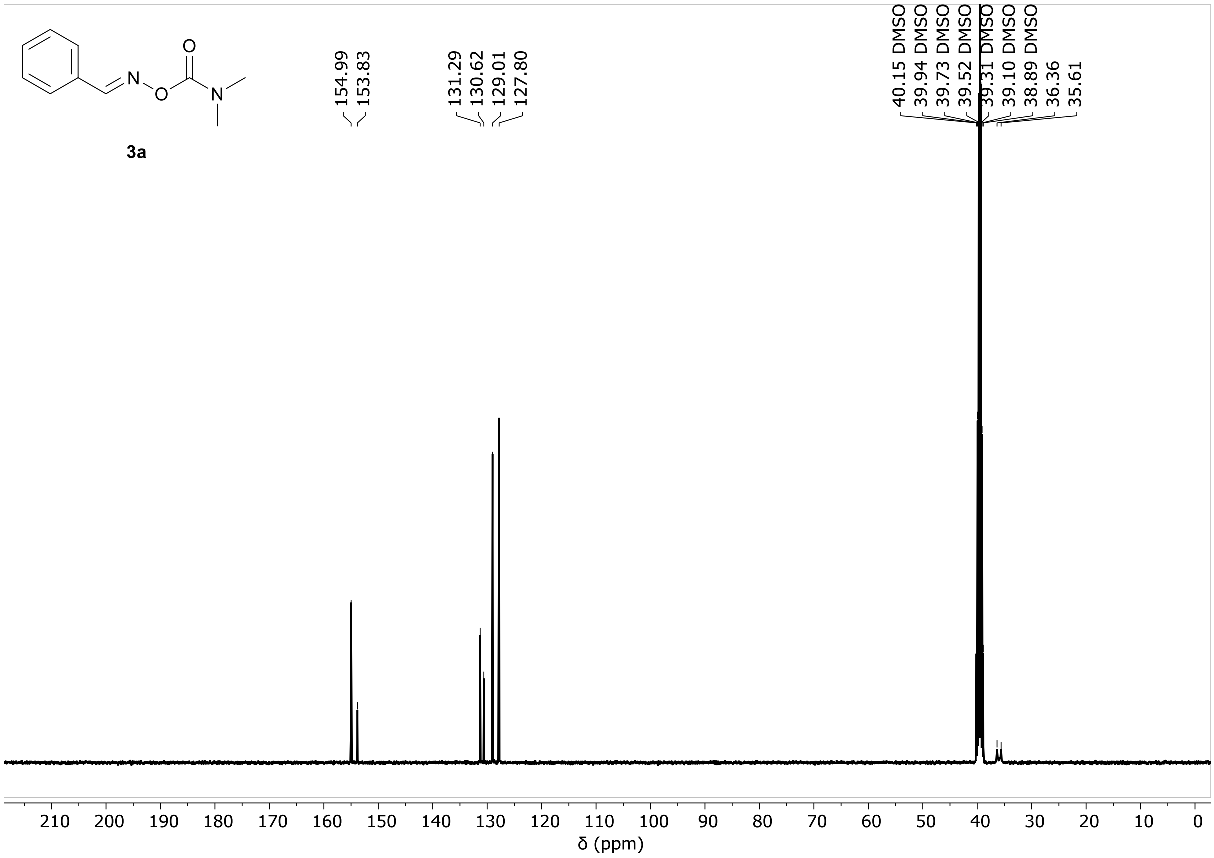

Figure S78. ^{13}C NMR spectrum of benzaldehyde *O*-dimethylcarbamoyloxime **3a** in DMSO-d_6.

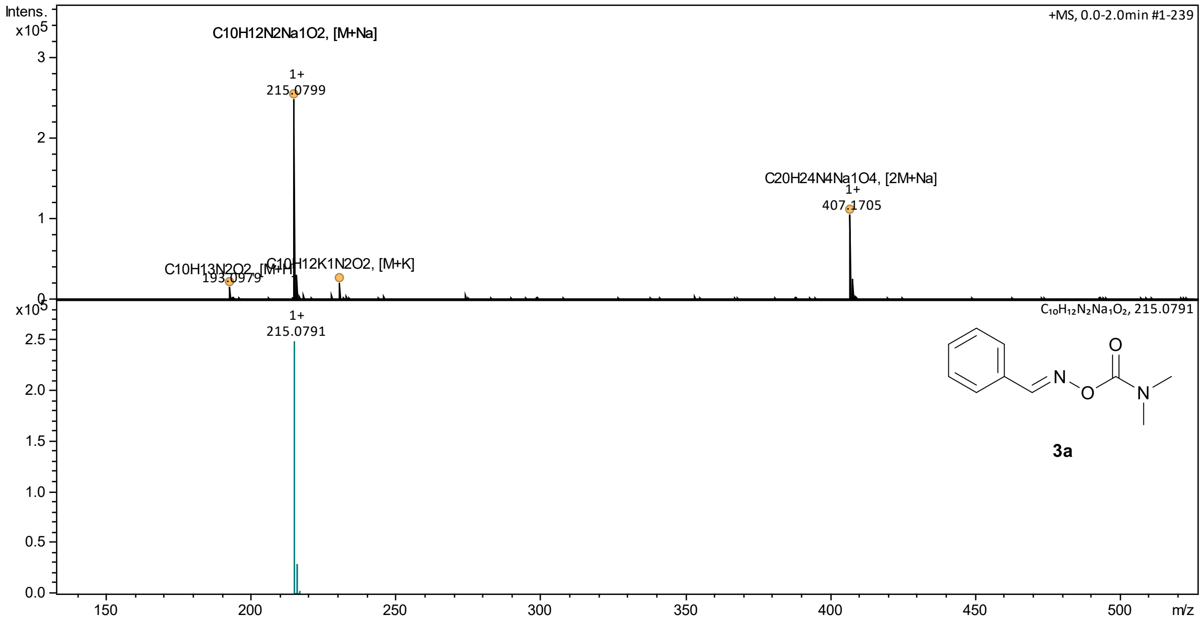

Figure S79. Deconvoluted ESI$^+$ HRMS (top) and calculated mass (bottom) spectra of benzaldehyde *O*-dimethylcarbamoyloxime **3a**.

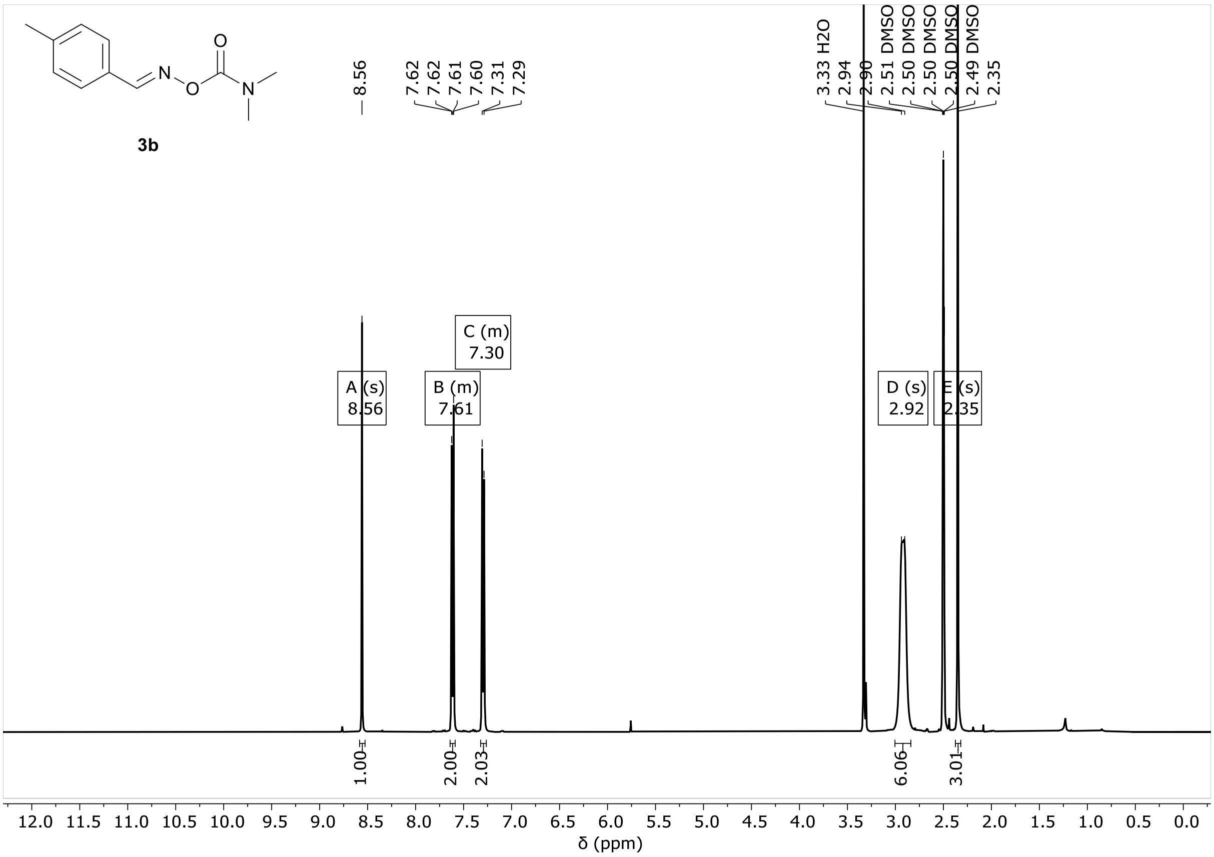

Figure S80. ^{1}H NMR spectrum of 4-methylbenzaldehyde *O*-dimethylcarbamoyloxime **3b** in DMSO-d_6.

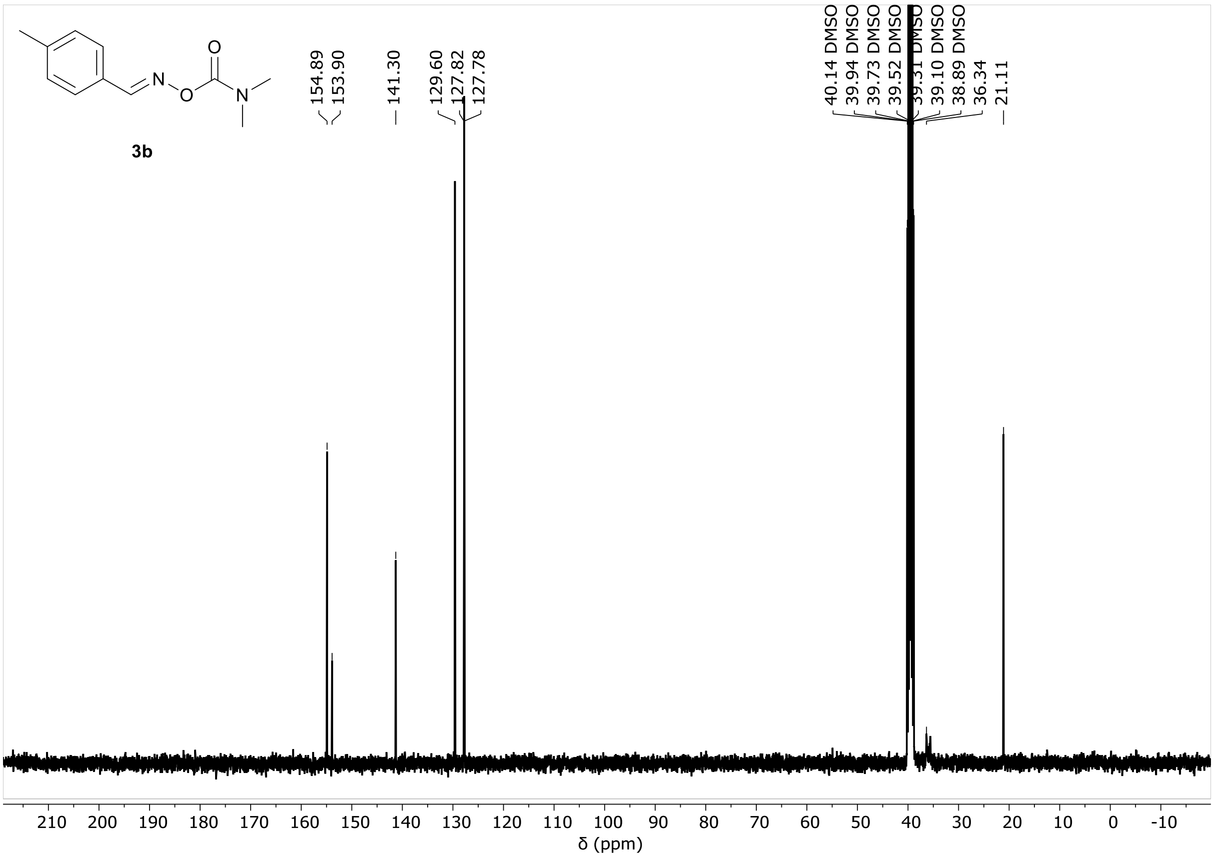

Figure S81. ^{13}C NMR spectrum of 4-methylbenzaldehyde *O*-dimethylcarbamoyloxime **3b** in DMSO-d_6.

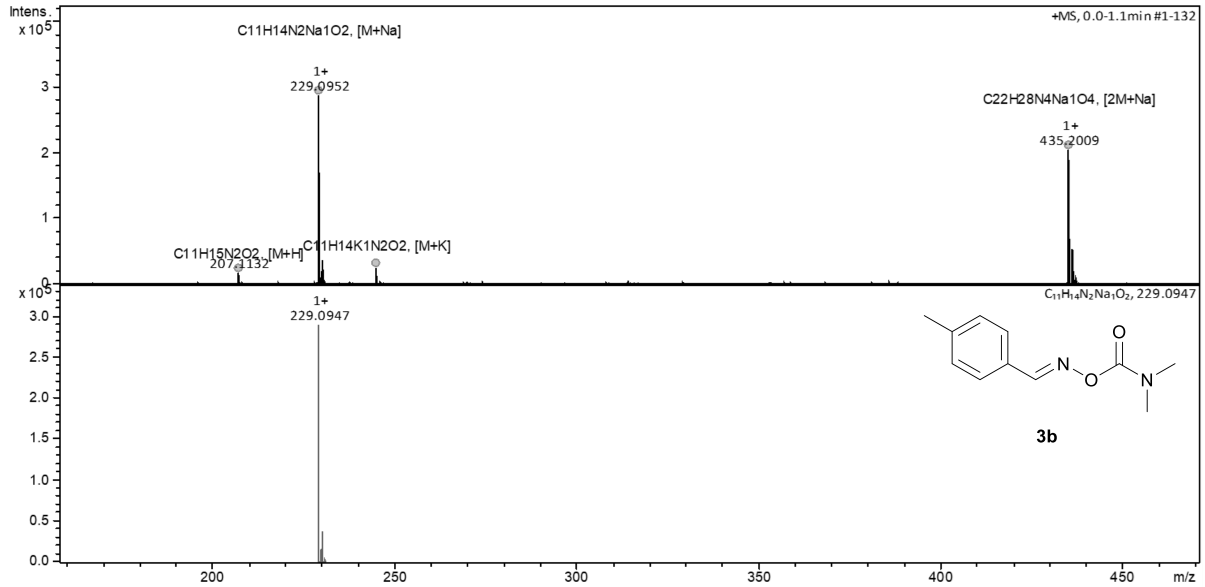

Figure S82. Deconvoluted ESI⁺ HRMS (top) and calculated mass (bottom) spectra of 4-methylbenzaldehyde *O*-dimethylcarbamoyloxime **3b**.

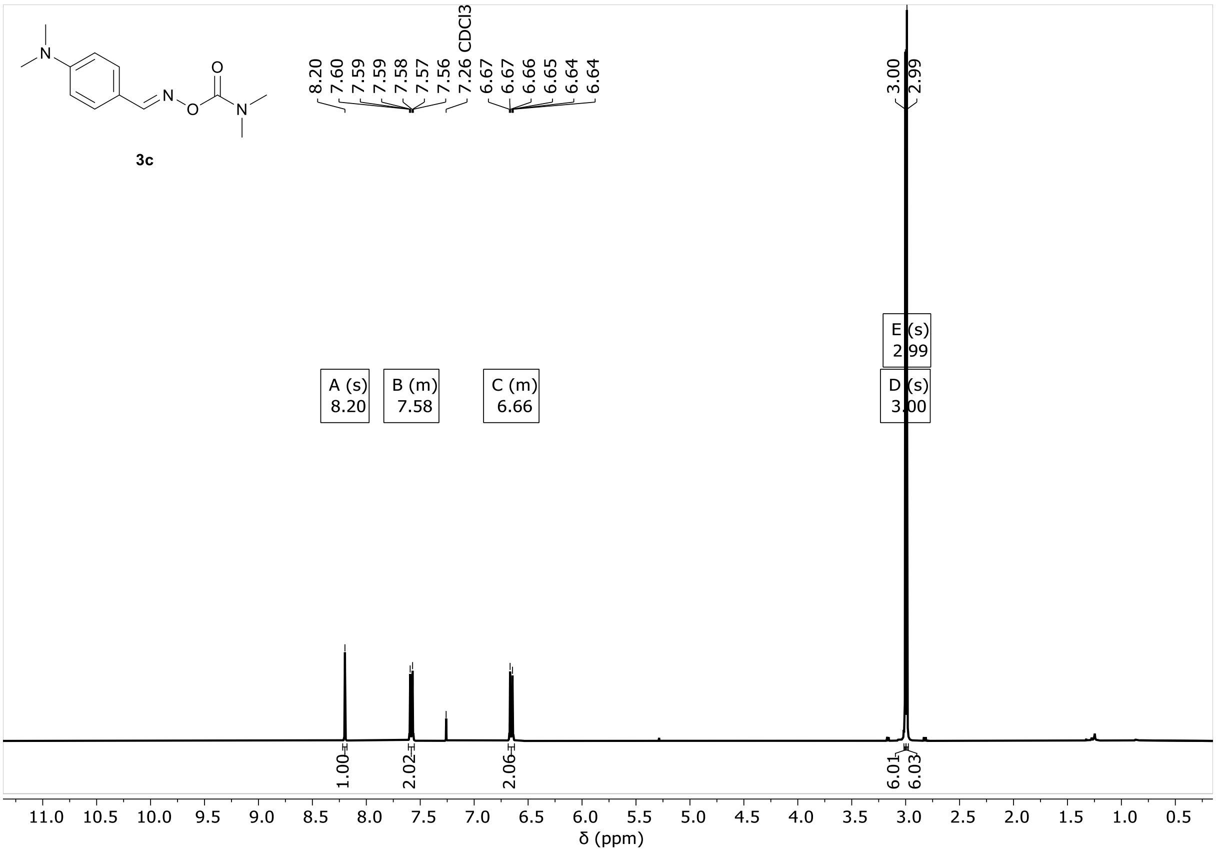

Figure S83. ^{1}H NMR spectrum of 4-(dimethylamino)benzaldehyde *O*-dimethylcarbamoyloxime **3c** in $CDCl_3$.

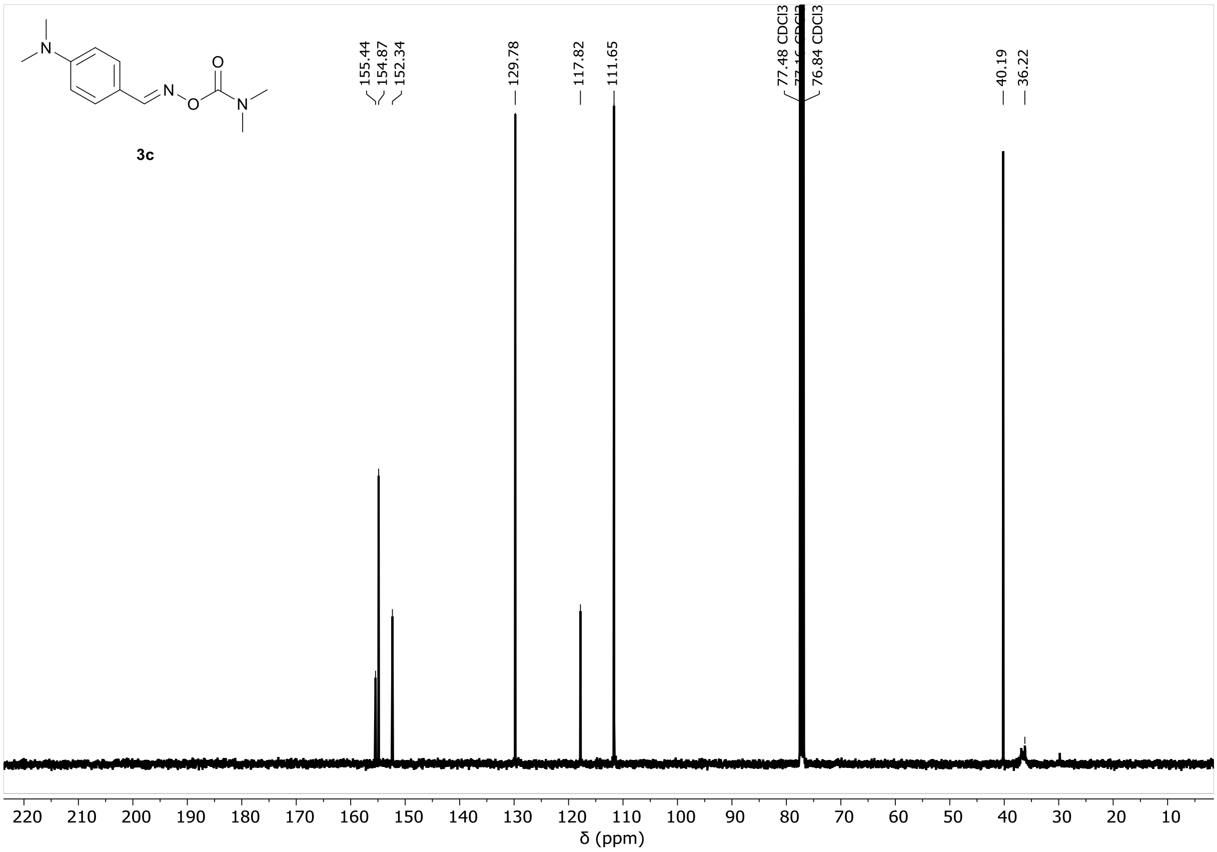

Figure S84. ^{13}C NMR spectrum of 4-hydroxybenzaldehyde *O*-dimethylcarbamoyloxime **3c** in $CDCl_3$.

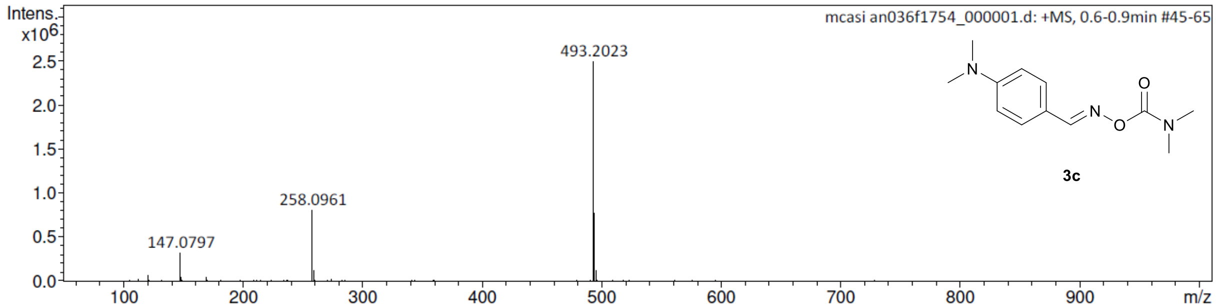

Figure S85. Deconvoluted ESI⁺ HRMS spectrum of 4-(dimethylamino)benzaldehyde *O*-dimethylcarbamoyloxime **3c**.

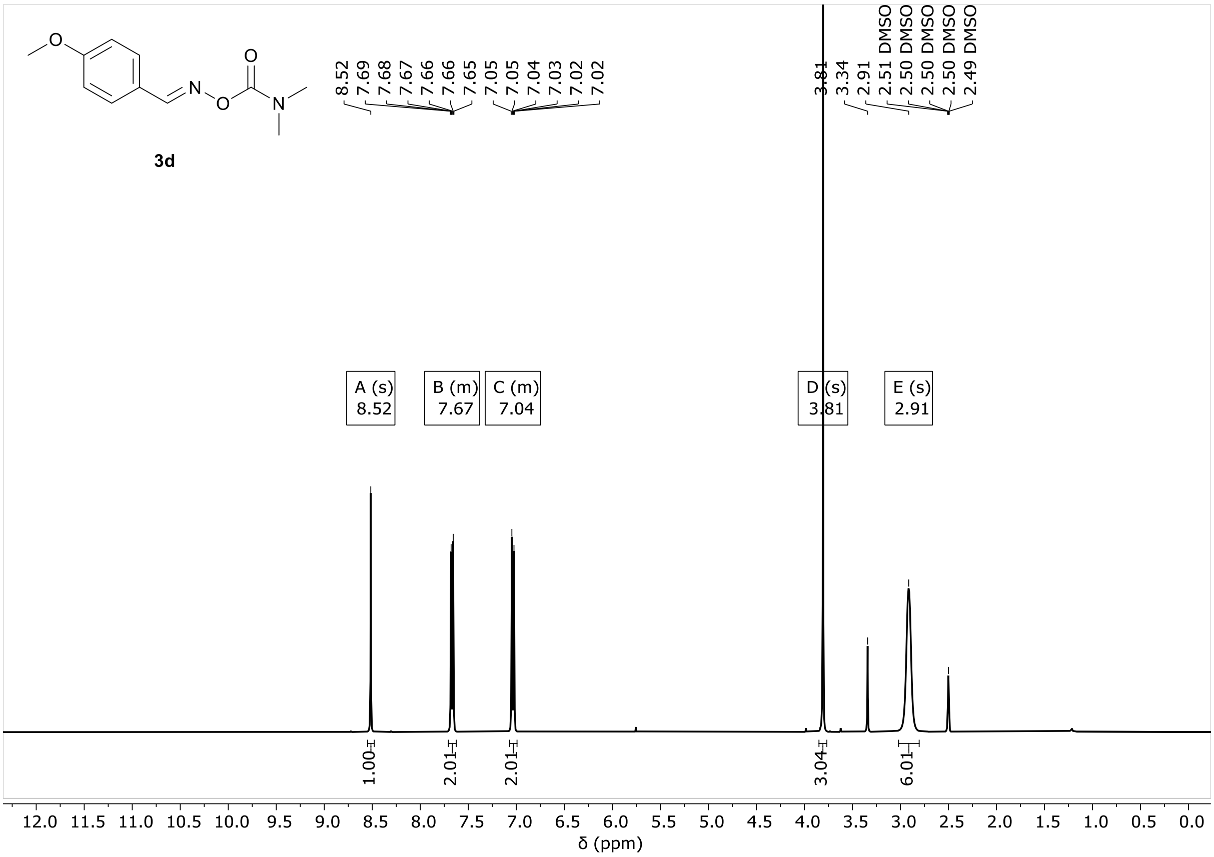

Figure S86. ^{1}H NMR spectrum of 4-methoxybenzaldehyde *O*-dimethylcarbamoyloxime **3d** in DMSO-d_6.

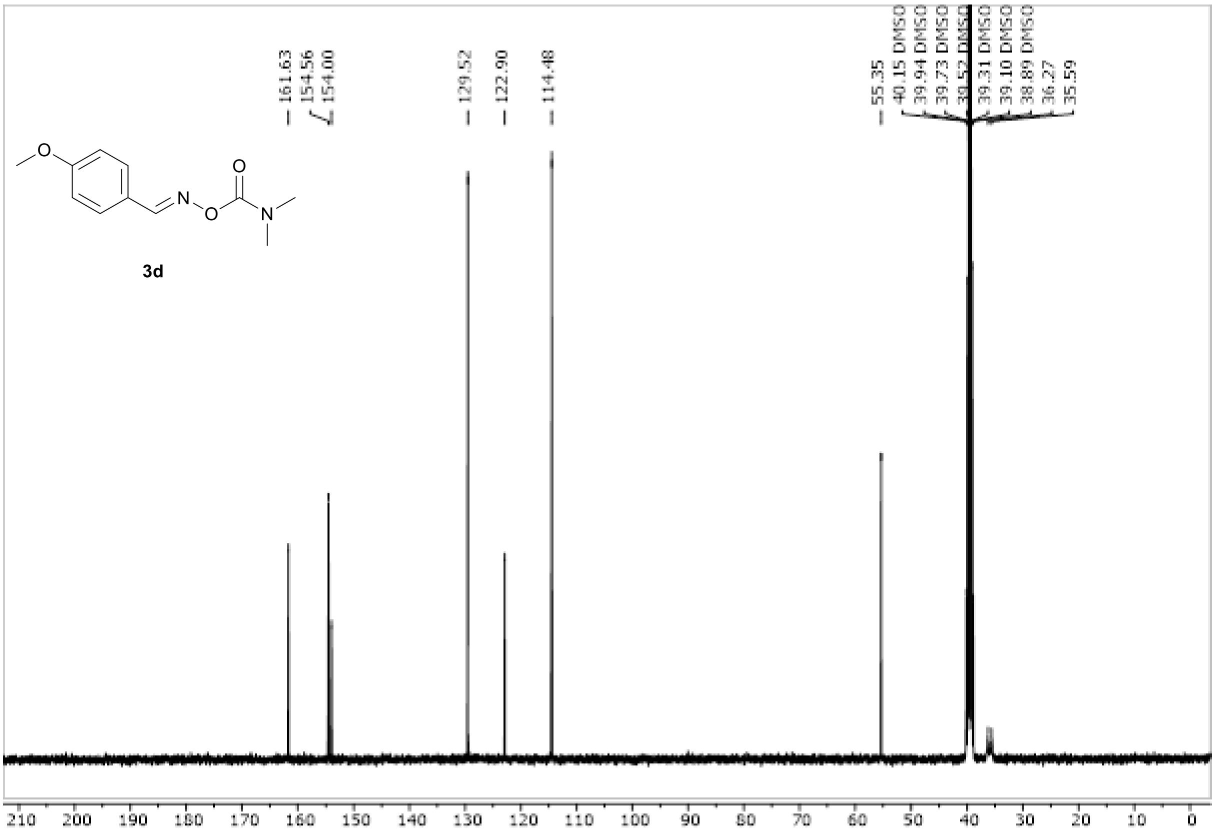

Figure S87. ^{13}C NMR spectrum of 4-methoxybenzaldehyde *O*-dimethylcarbamoyloxime **3d** in DMSO-d_6.

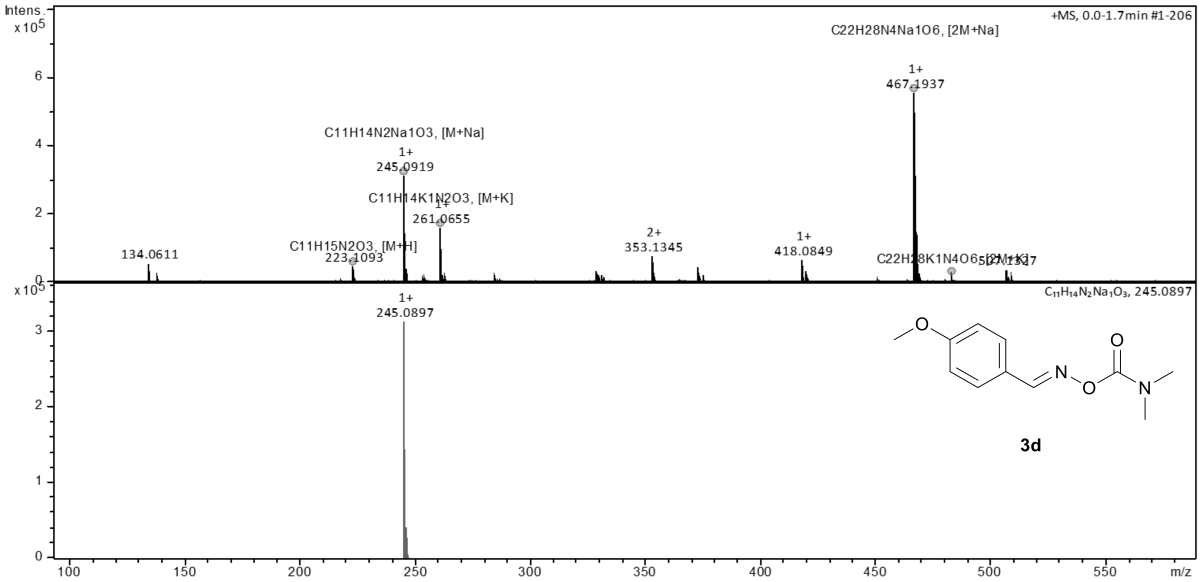

Figure S88. Deconvoluted ESI+ HRMS (top) and calculated mass (bottom) spectra of 4-methoxybenzaldehyde *O*-dimethylcarbamoyloxime **3d**.

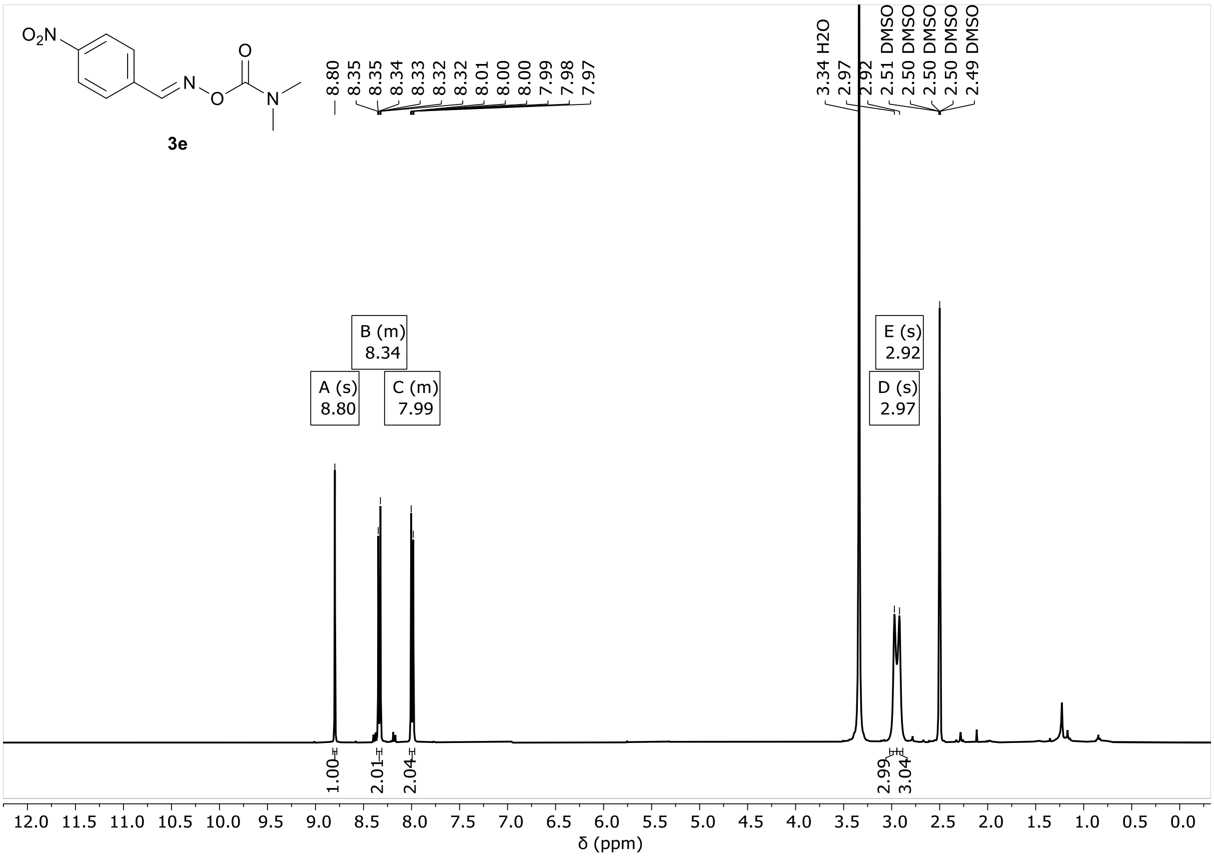

Figure S89. ^{1}H NMR spectrum of 4-nitrobenzaldehyde *O*-dimethylcarbamoyloxime **3e** in DMSO-d_6.

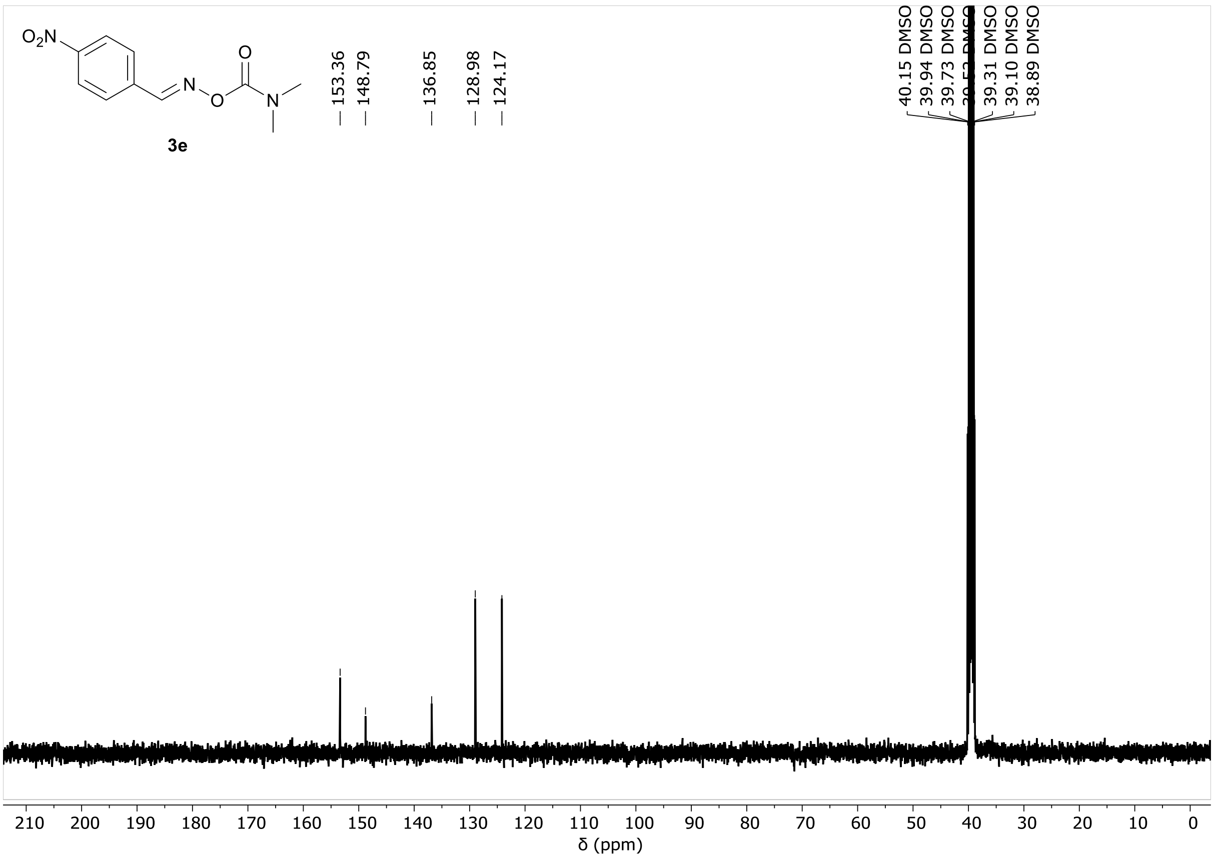

Figure S90.^{13}C NMR spectrum of 4-nitrobenzaldehyde *O*-dimethylcarbamoyloxime **3e** in DMSO-d_6 (NMR sample was prepared with low concentration; therefore, the two methyl peaks usually appearing around 30.0 ppm are not visible).

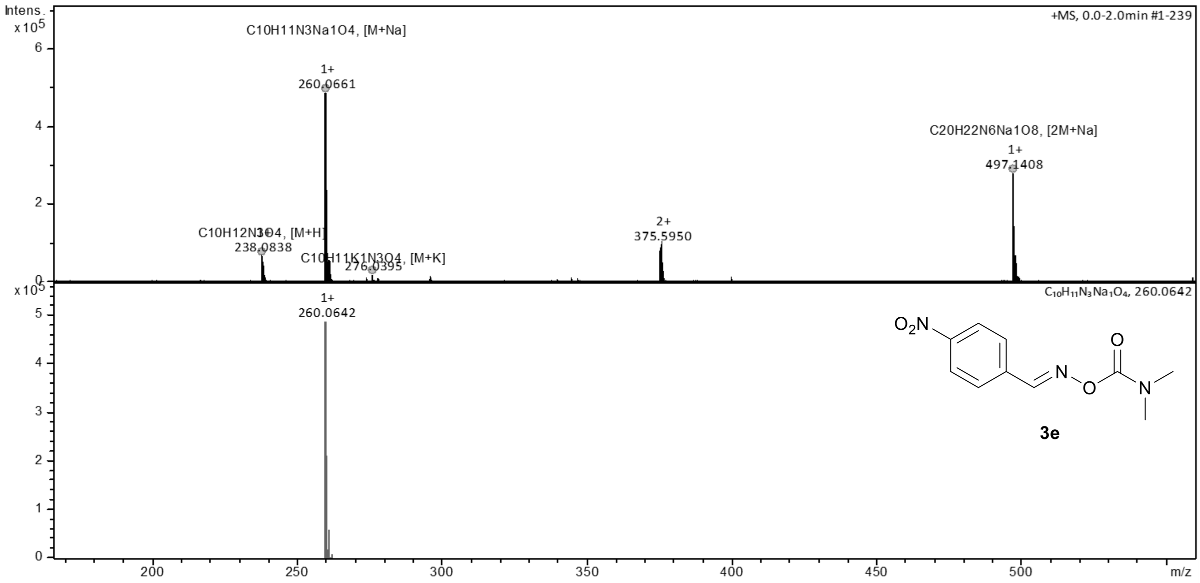

Figure S91. Deconvoluted ESI$^+$ HRMS (top) and calculated mass (bottom) spectra of 4-nitrobenzaldehyde *O*-dimethylcarbamoyloxime **3e**.

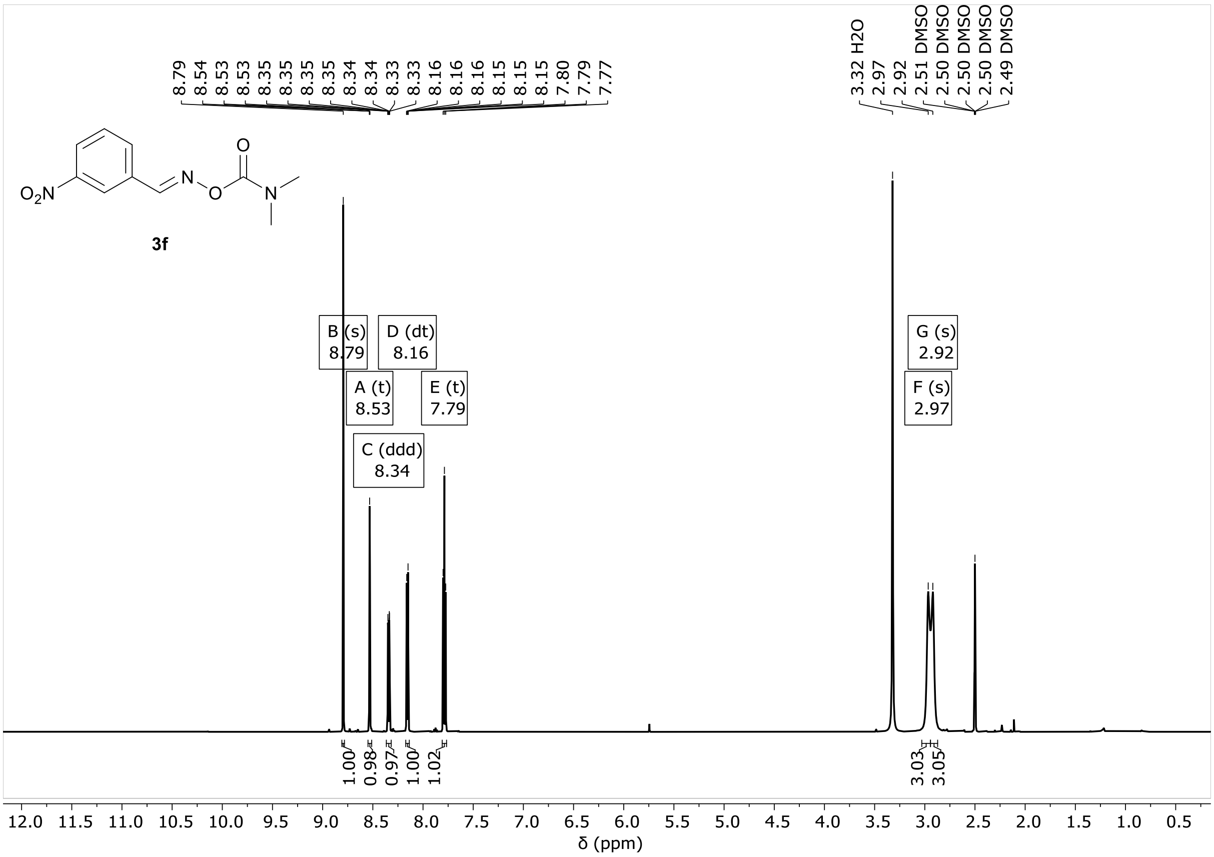

Figure S92. ^{1}H NMR spectrum of 3-nitrobenzaldehyde *O*-dimethylcarbamoyloxime **3f** in DMSO-d_6.

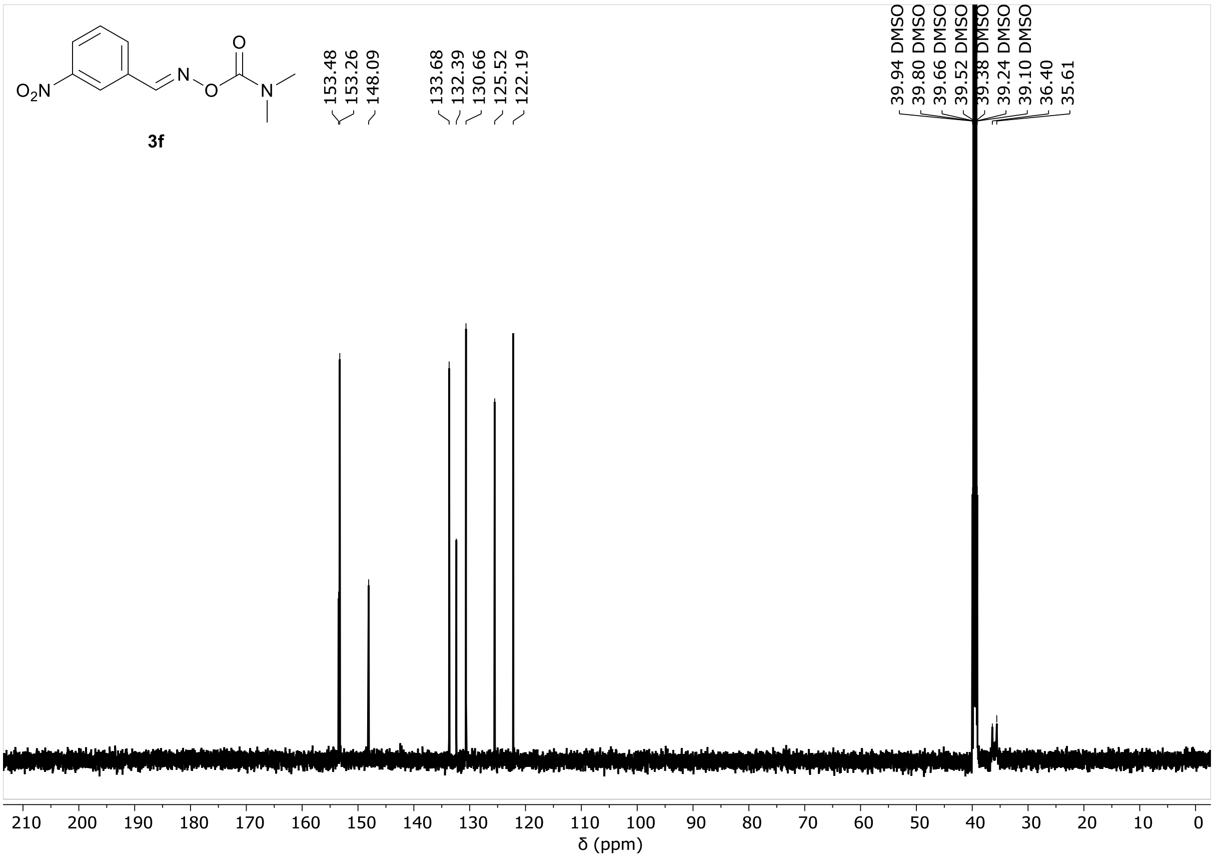

Figure S93. ^{13}C NMR spectrum of 3-nitrobenzaldehyde *O*-dimethylcarbamoyloxime **3f** in DMSO-d_6.

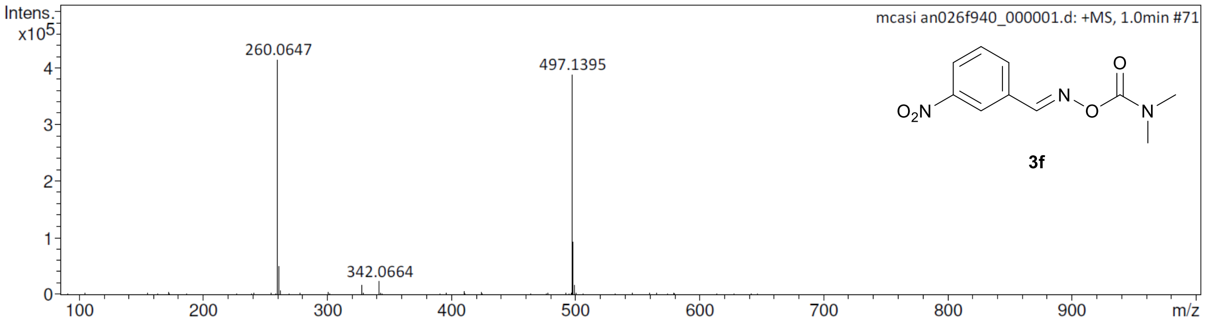

Figure S94. Deconvoluted ESI$^+$ HRMS spectrum of 3-nitrobenzaldehyde *O*-dimethylcarbamoyloxime **3f**.

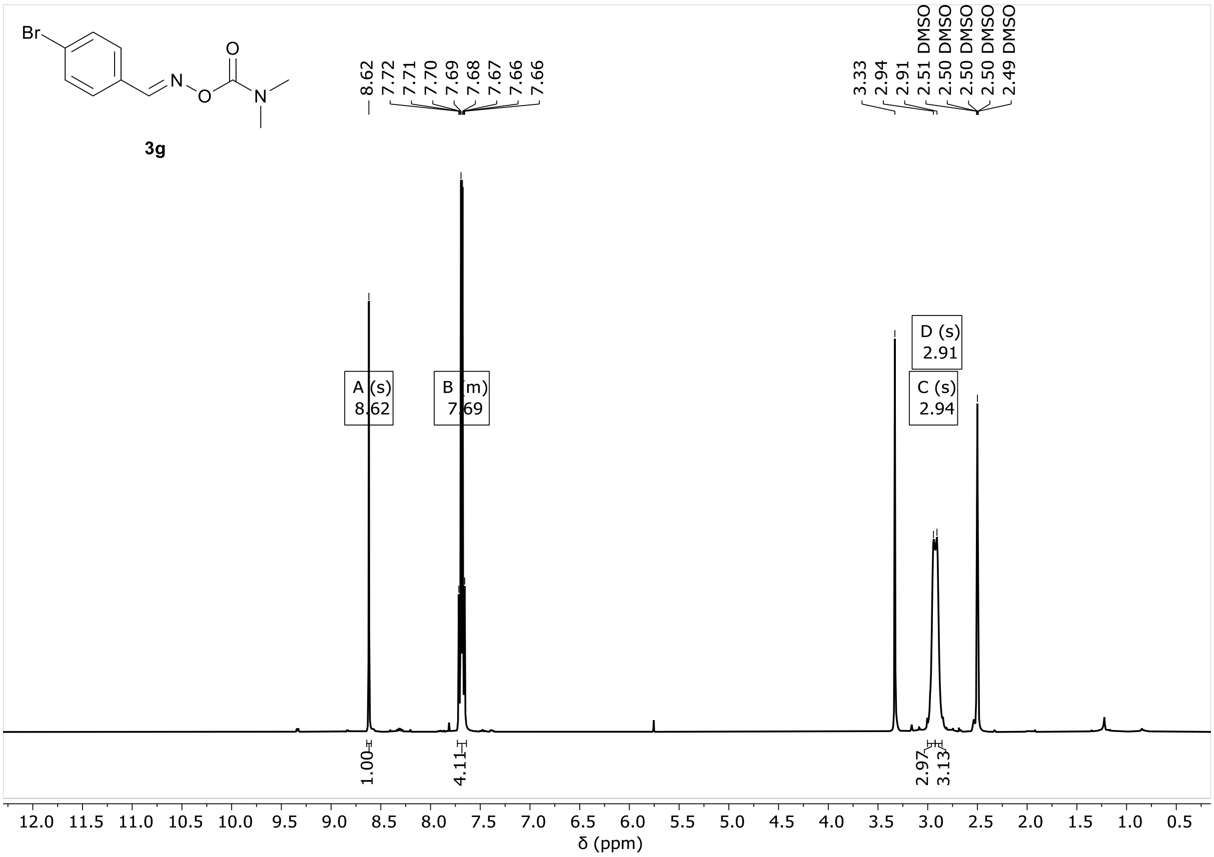

Figure S95. ^{1}H NMR spectrum of 4-bromobenzaldehyde *O*-dimethylcarbamoyloxime **3g** in DMSO-d_6.

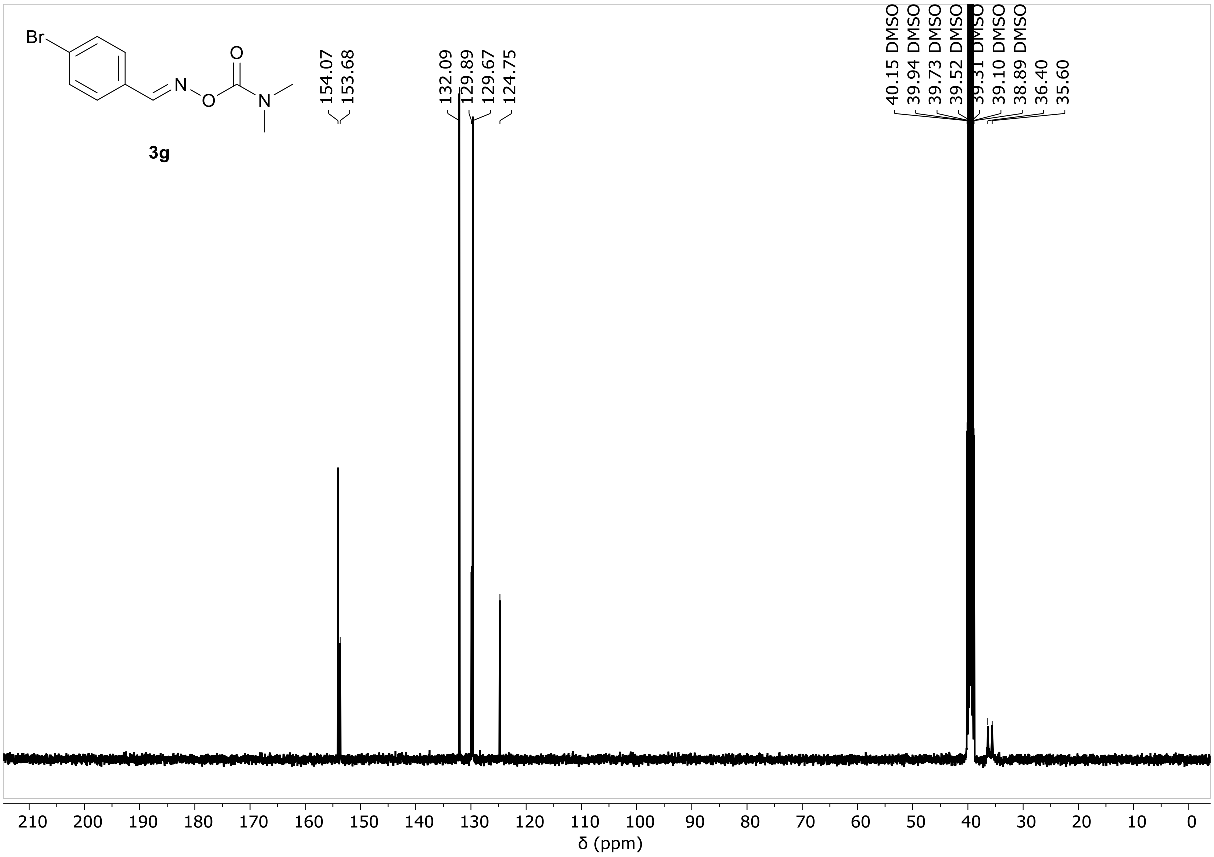

Figure S96. ^{13}C NMR spectrum of 4-bromobenzaldehyde *O*-dimethylcarbamoyloxime **3g** in DMSO-d_6.

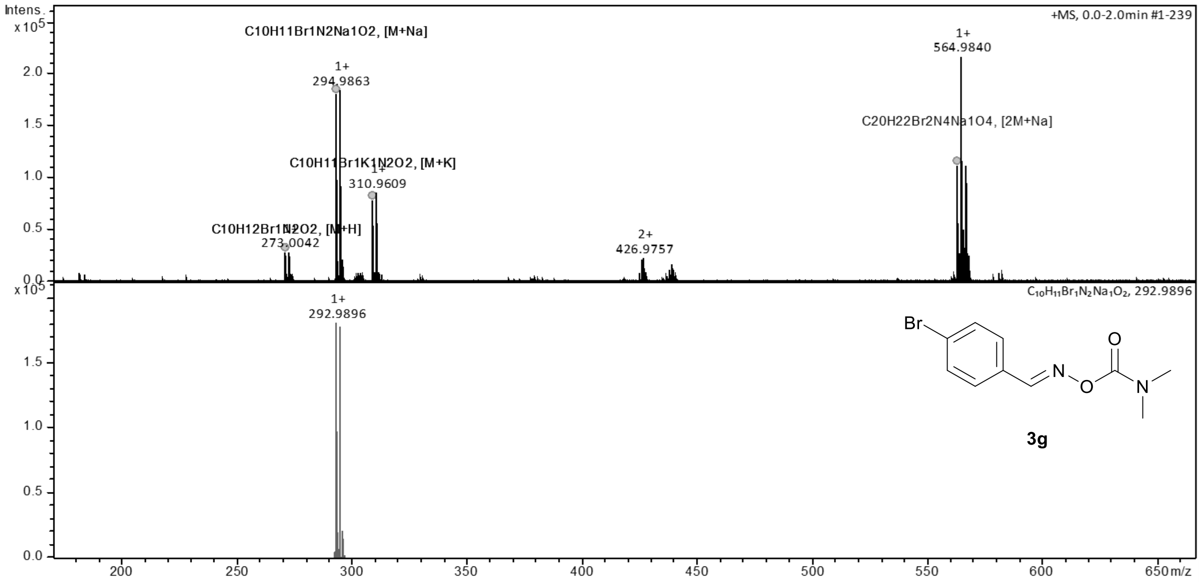

Figure S97. Deconvoluted ESI$^+$ HRMS (top) and calculated mass (bottom) spectra of 4-bromobenzaldehyde *O*-dimethylcarbamoyloxime **3g**.

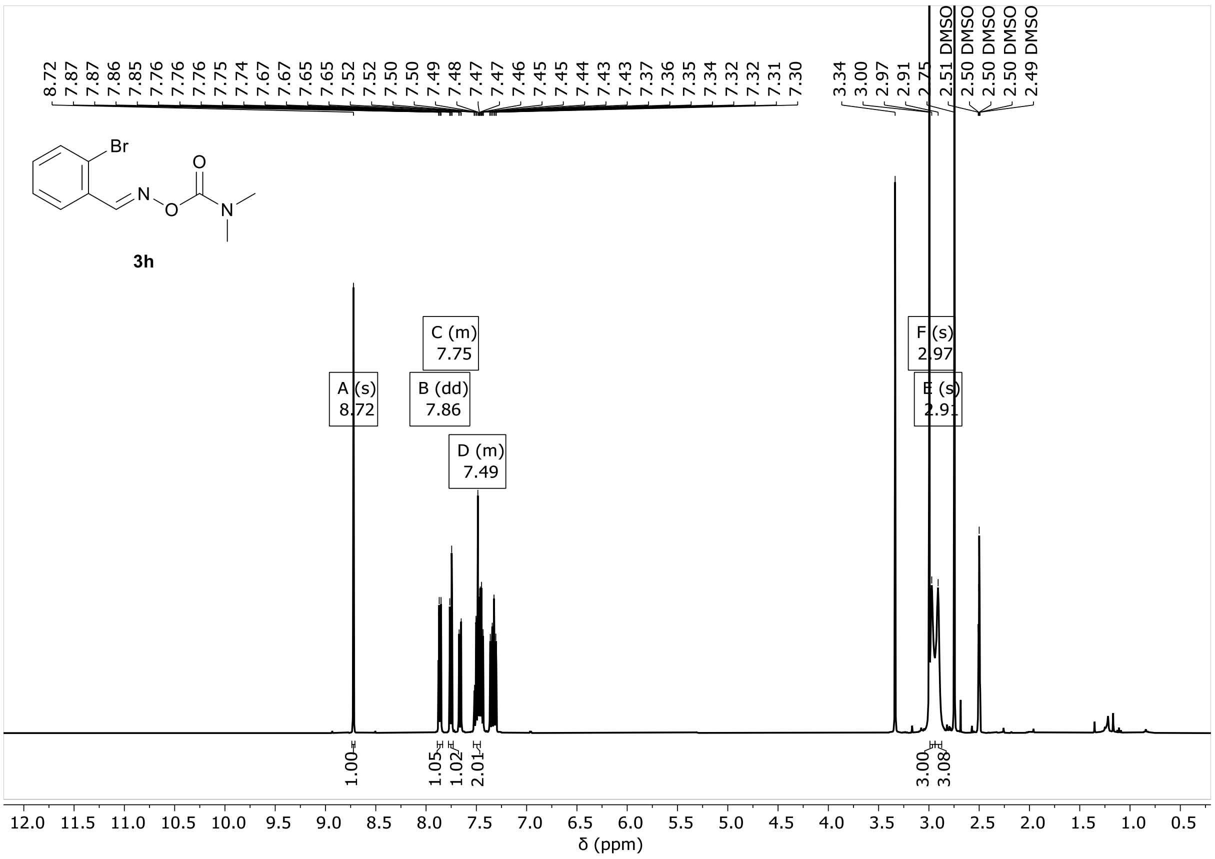

Figure S98. ^{1}H NMR spectrum of 2-bromobenzaldehyde *O*-dimethylcarbamoyloxime **3h** in DMSO-d_6.

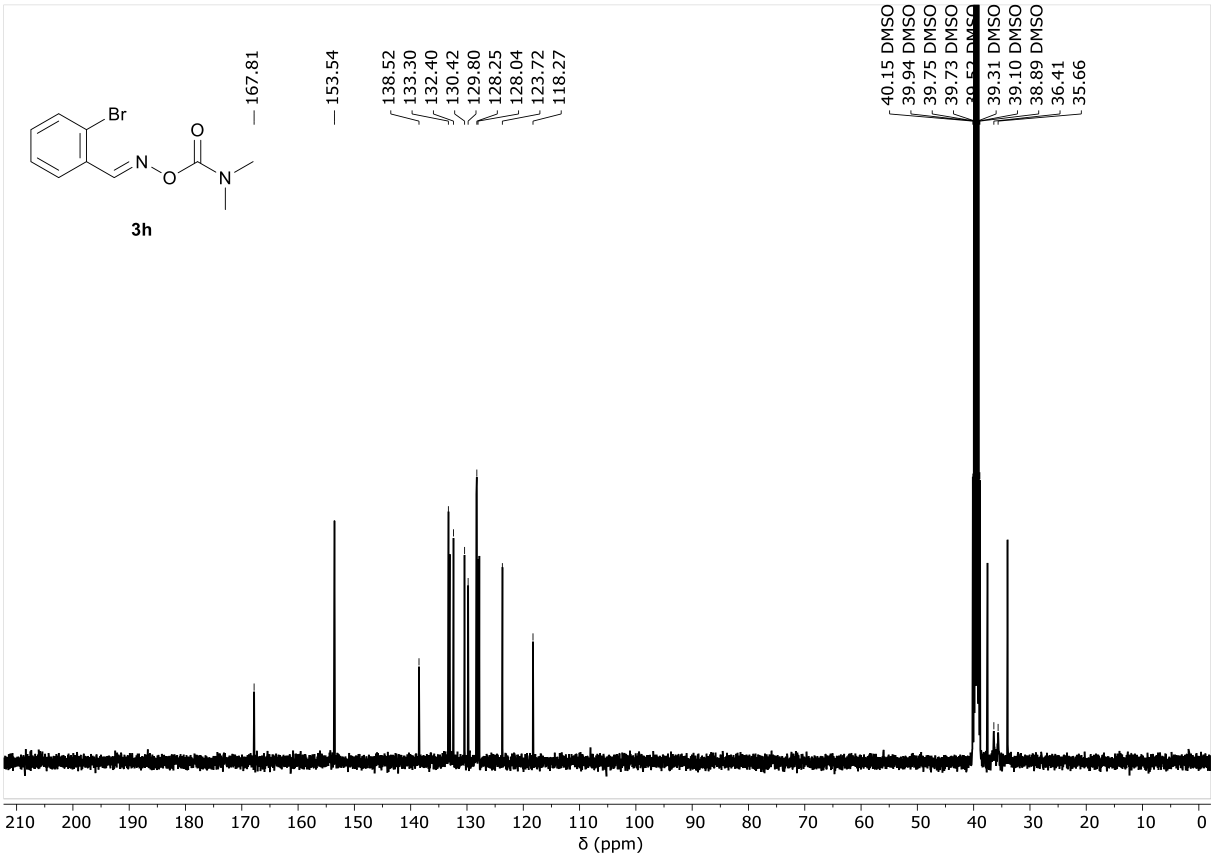

Figure S99. ^{13}C NMR spectrum of 2-bromobenzaldehyde *O*-dimethylcarbamoyloxime **3h** in DMSO-d_6.

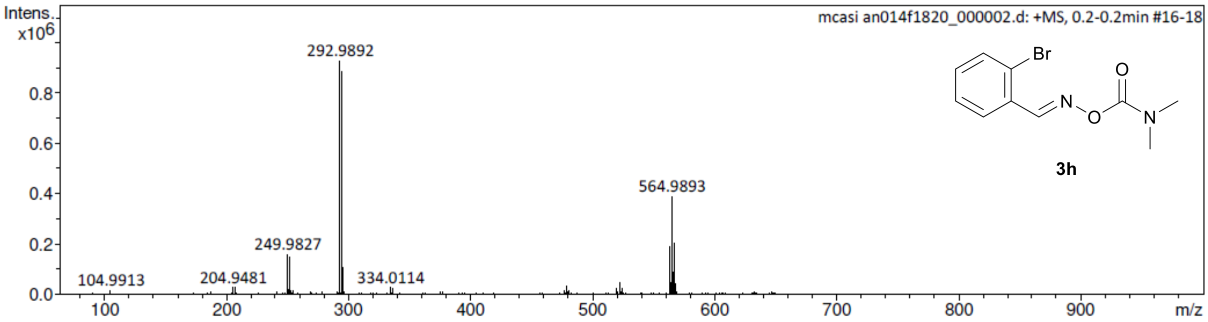

Figure S100. Deconvoluted ESI⁺ HRMS spectrum of 2-bromobenzaldehyde *O*-dimethylcarbamoyloxime **3h**.

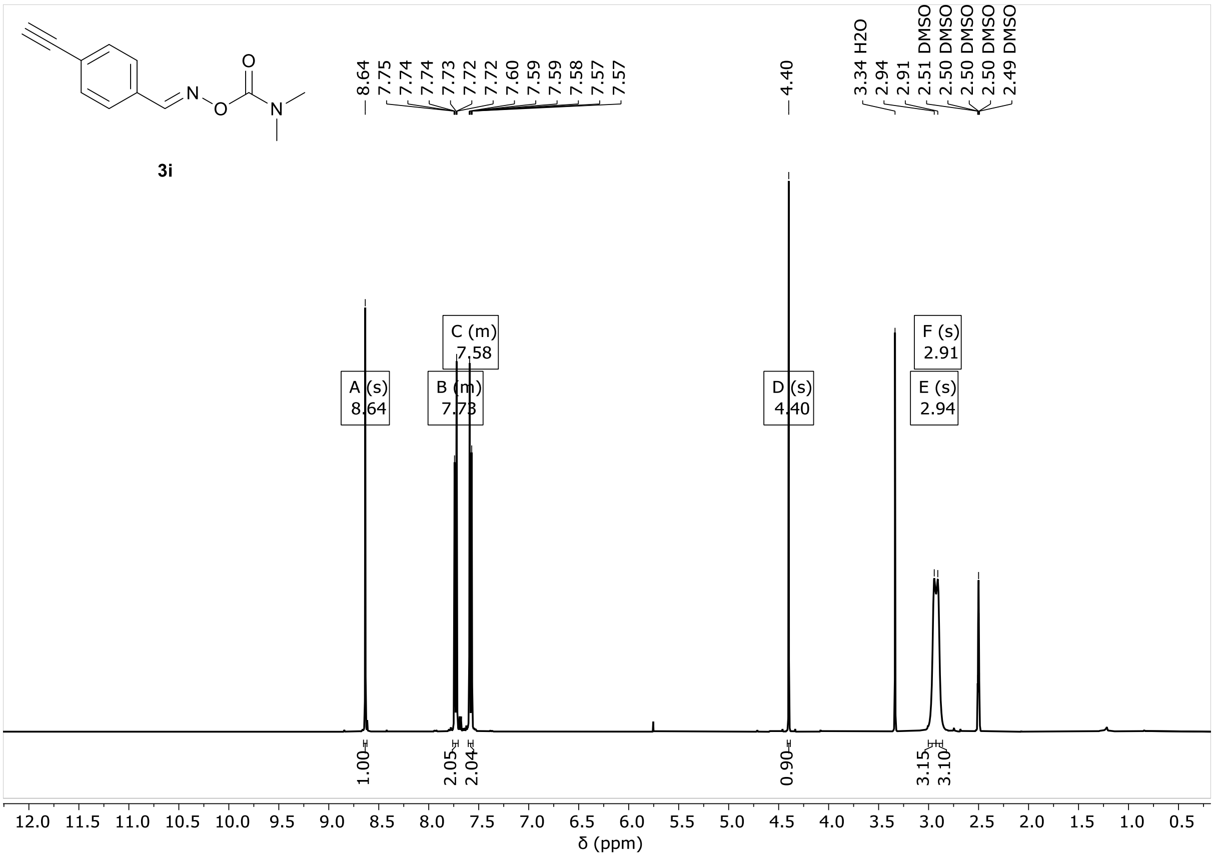

Figure S101. ^{1}H NMR spectrum of 4-ethynylbenzaldehyde *O*-dimethylcarbamoyloxime **3i** in DMSO-d_6.

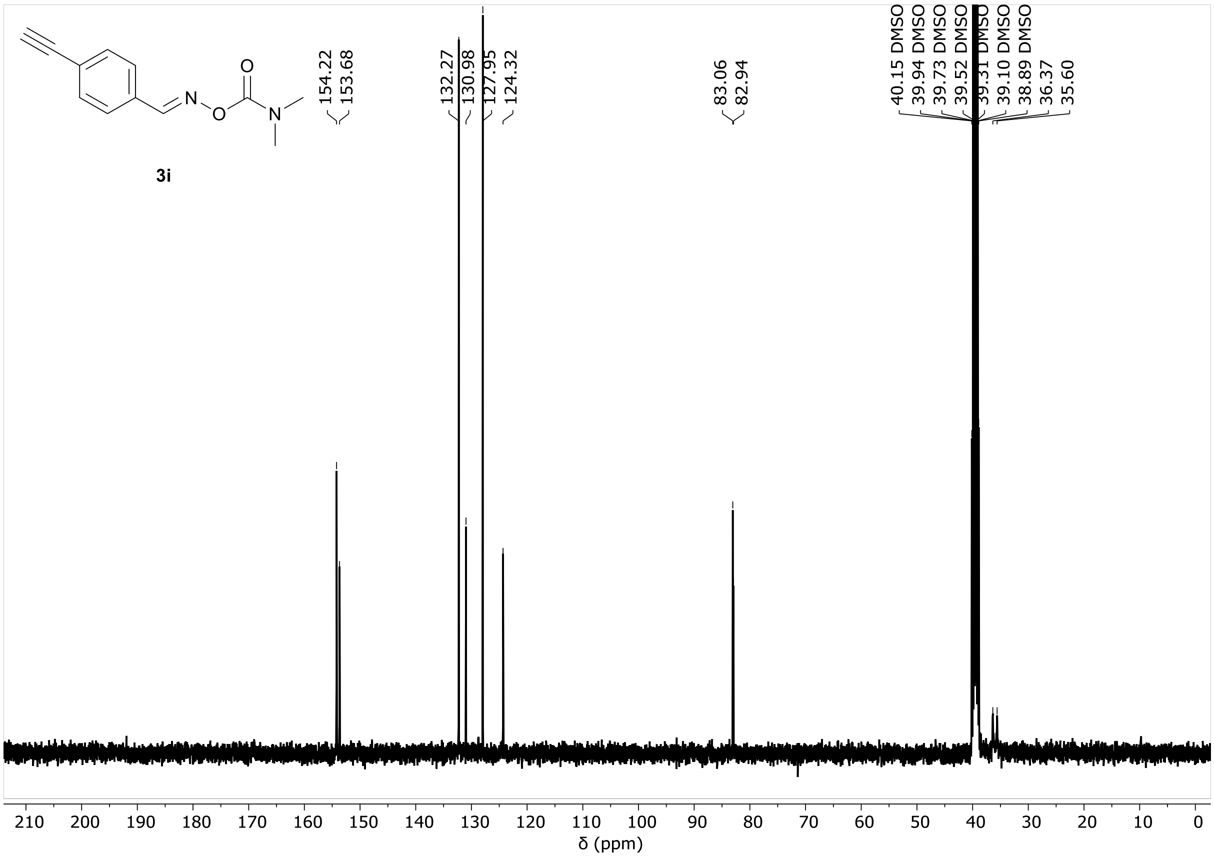

Figure S102. ^{13}C NMR spectrum of 4-ethynylbenzaldehyde *O*-dimethylcarbamoyloxime **3i** in DMSO-d_6.

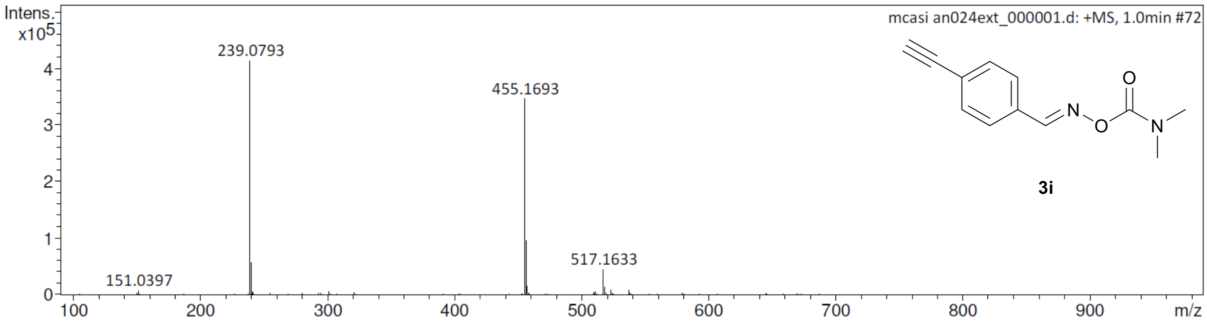

Figure S103. Deconvoluted ESI$^+$ HRMS spectrum of 4-ethynylbenzaldehyde *O*-dimethylcarbamoyloxime **3i**.

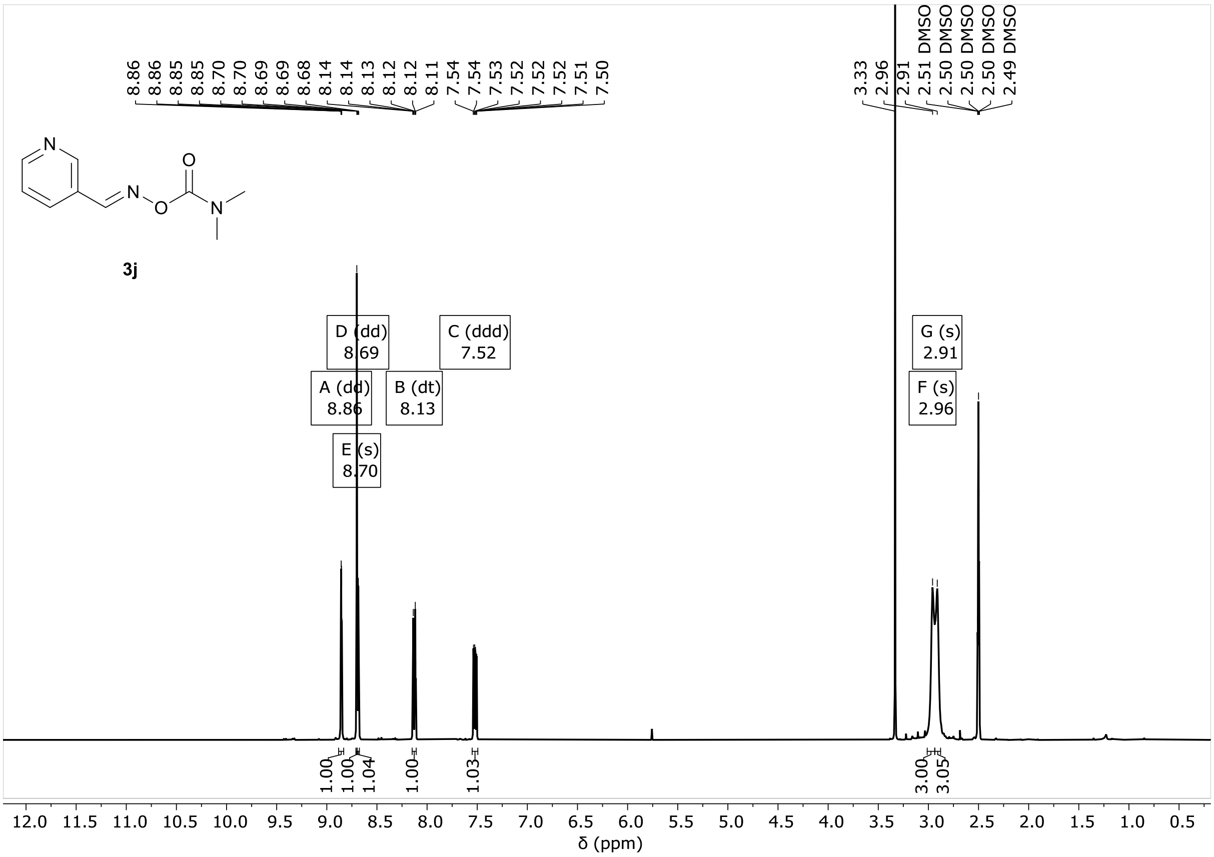

Figure S104. ^{1}H NMR spectrum of nicotinaldehyde *O*-dimethylcarbamoyloxime **3j** in DMSO-d_6.

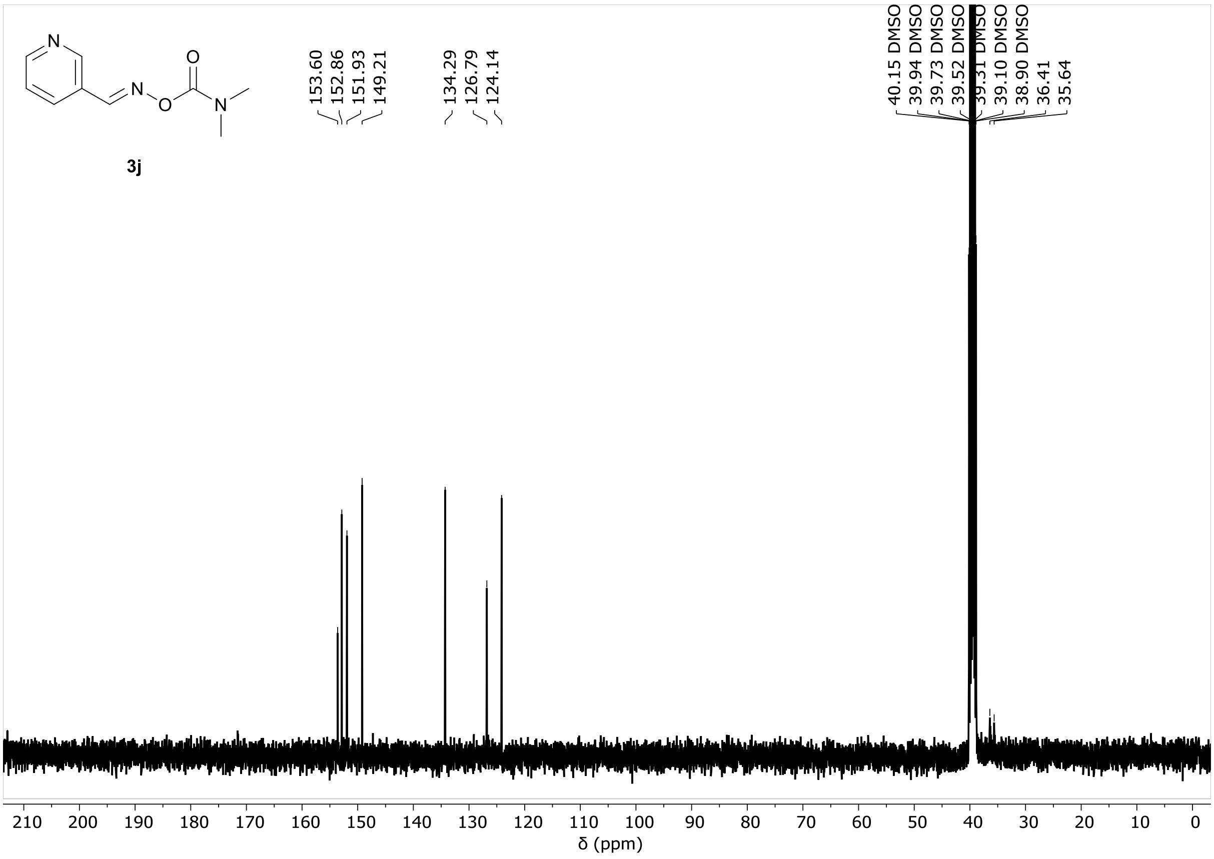

Figure S105. ^{13}C NMR spectrum of nicotinaldehyde *O*-dimethylcarbamoyloxime **3j** in DMSO-d_6.

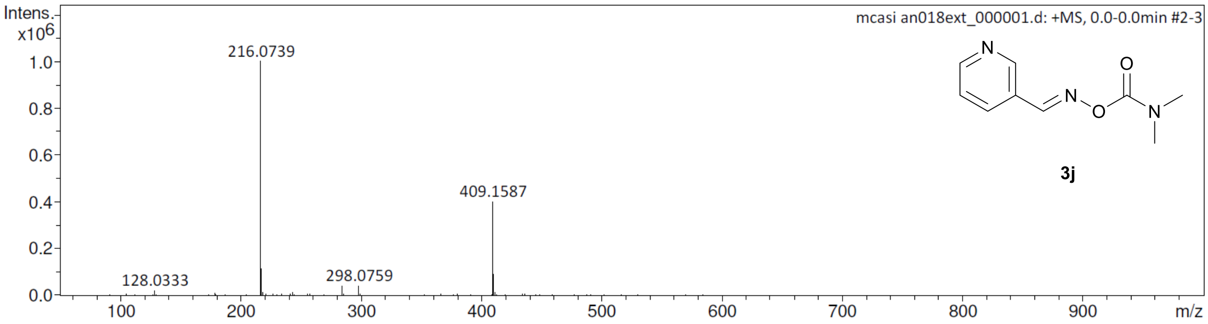

Figure S106. Deconvoluted ESI$^+$ HRMS spectrum of nicotinaldehyde *O*-dimethylcarbamoyloxime **3j**.

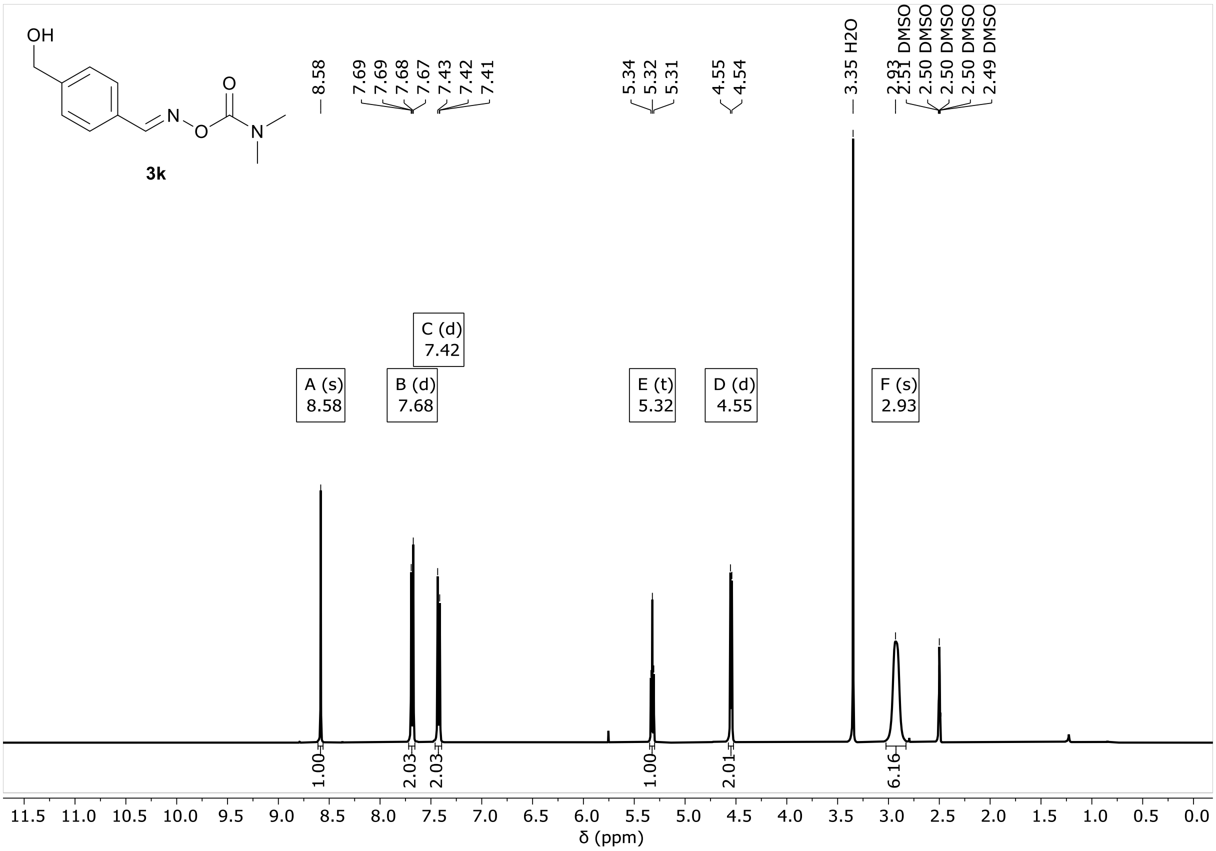

Figure S107. ^{1}H NMR spectrum of 4-(hydroxymethyl)benzaldehyde *O*-dimethylcarbamoyloxime **3k** in DMSO-d_6.

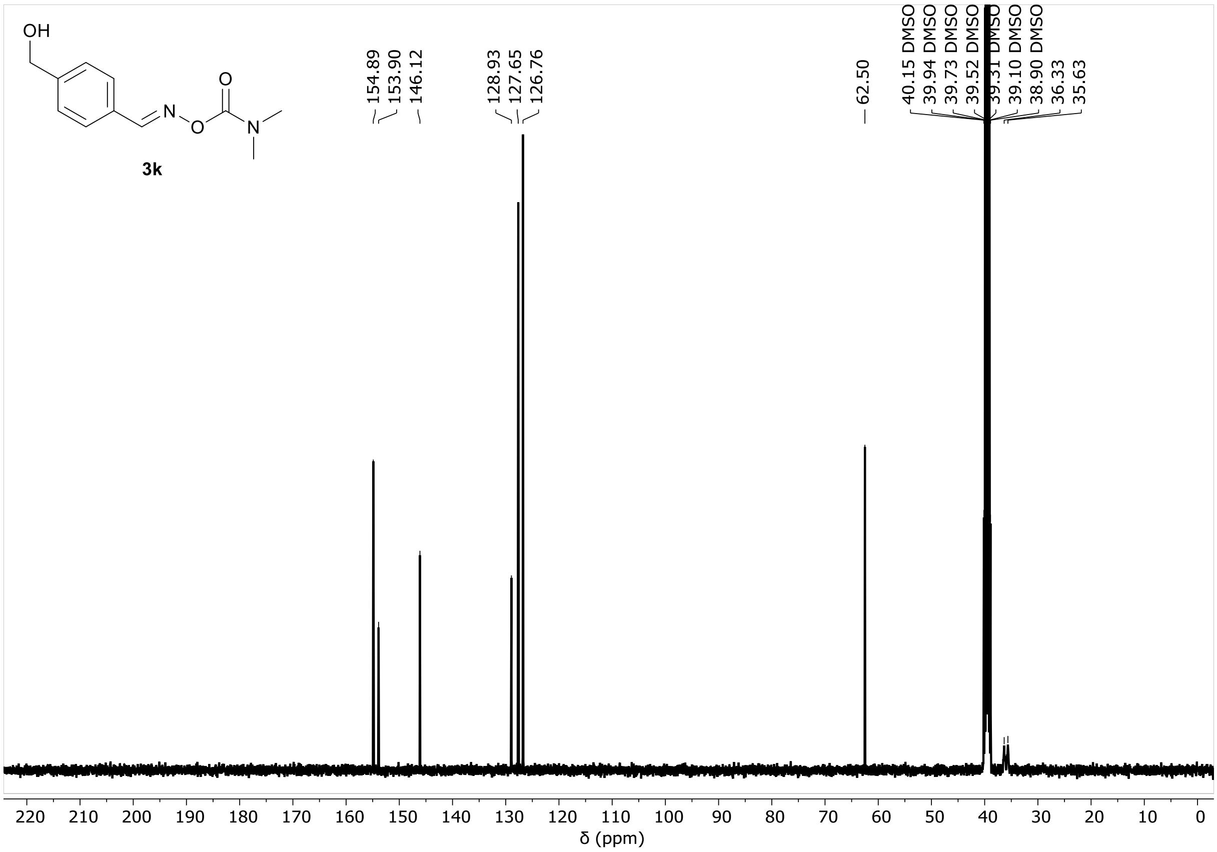

Figure S108. ^{13}C NMR spectrum of 4-(hydroxymethyl)benzaldehyde *O*-dimethylcarbamoyloxime **3k** in DMSO-d_6.

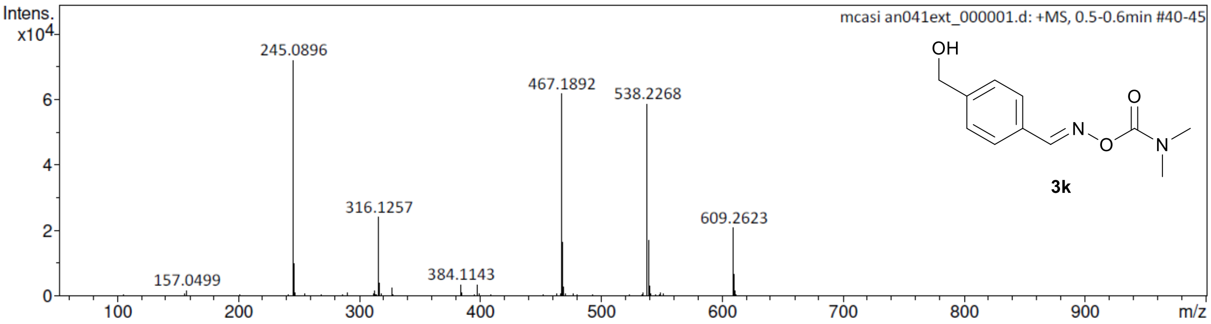

Figure S109. Deconvoluted ESI⁺ HRMS spectrum of 4-(hydroxymethyl)benzaldehyde *O*-dimethylcarbamoyloxime **3k**.

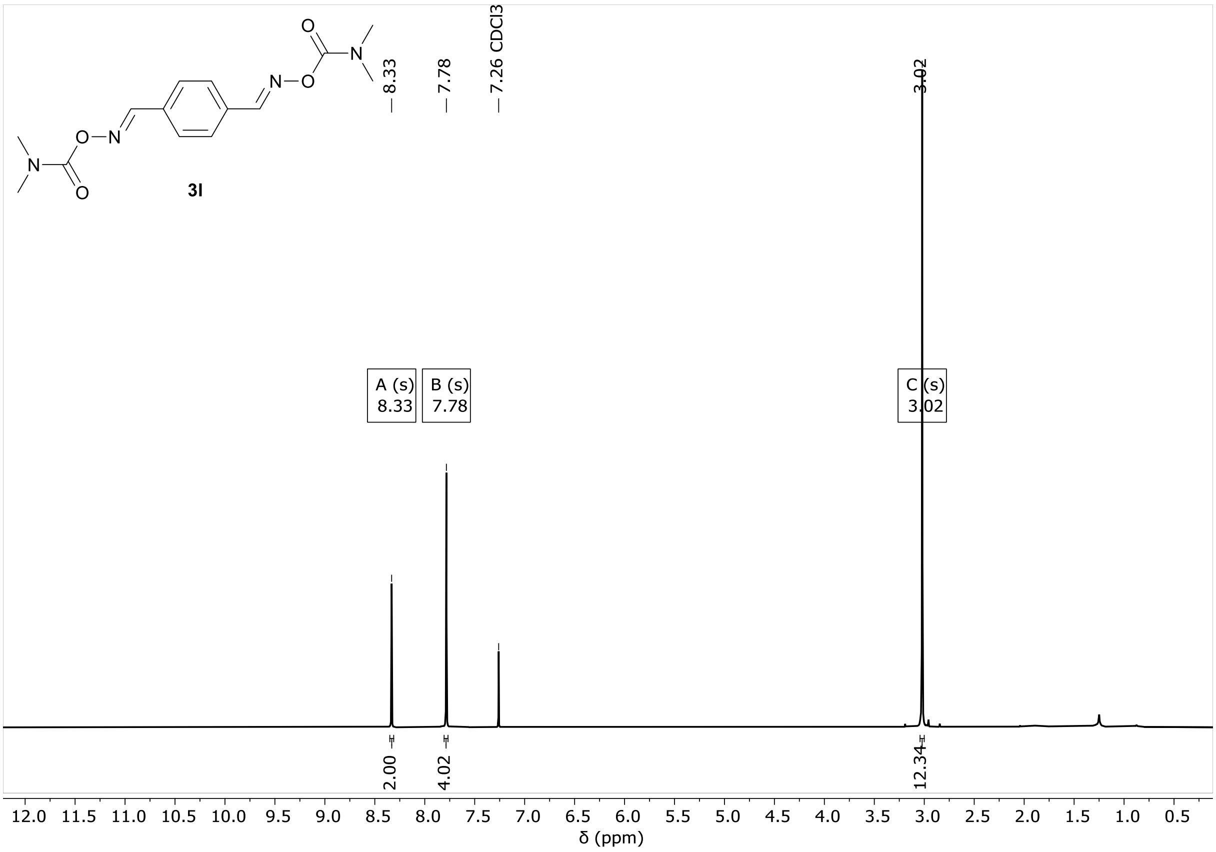

Figure S110. ^{1}H NMR spectrum of terephthalaldehyde *O,O*-didimethylcarbamoyl dioxime **3l** in $CDCl_3$.

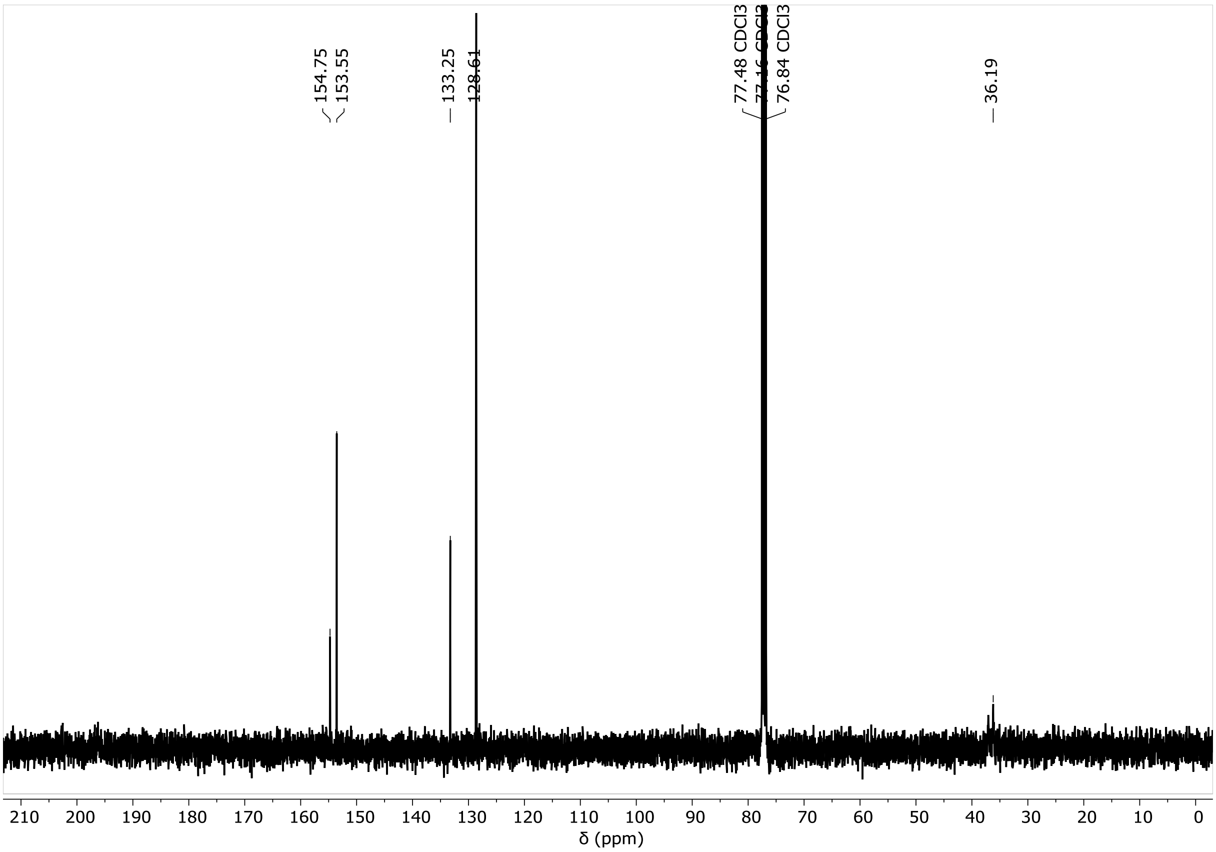

Figure S111. ^{13}C NMR spectrum of terephthalaldehyde *O,O*-didimethylcarbamoyl dioxime **3l** in $CDCl_3$.

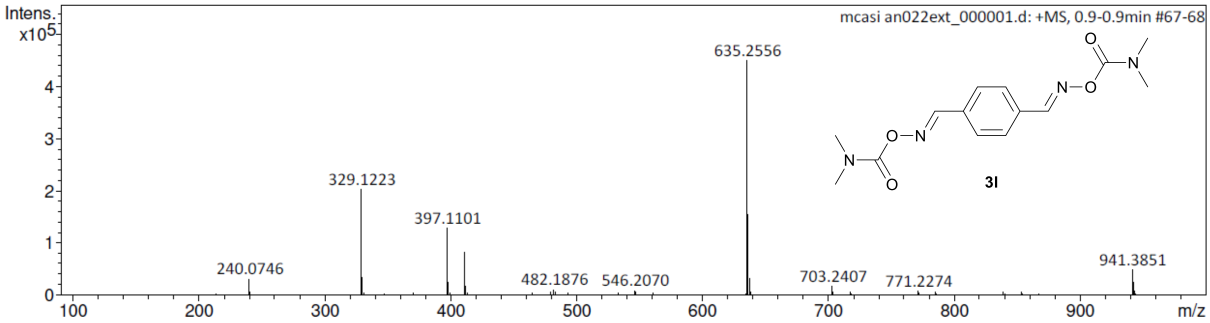

Figure S112. Deconvoluted ESI$^-$ HRMS spectrum of terephthalaldehyde *O,O*-didimethylcarbamoyl dioxime **3l**.

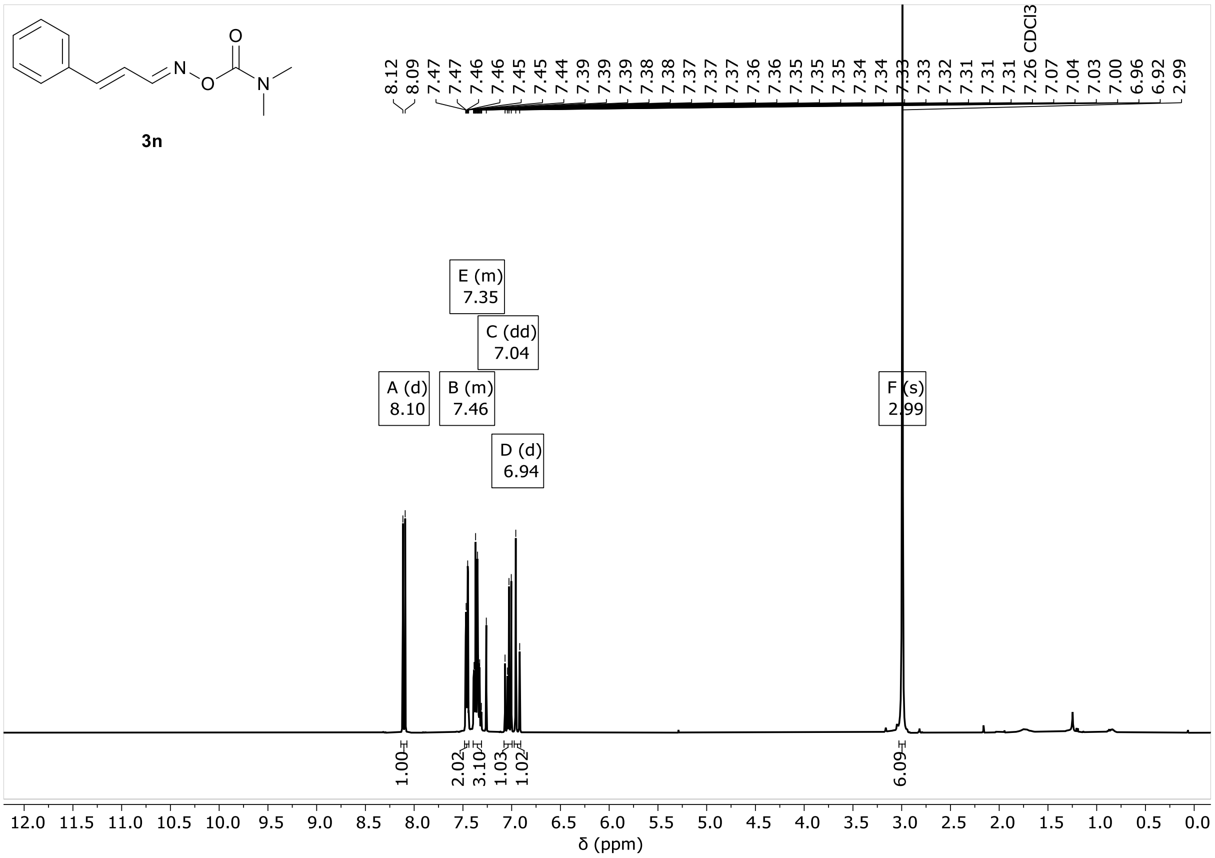

Figure S113. ^{1}H NMR spectrum of cinnamaldehyde *O*-dimethylcarbamoyloxime **3m** in $CDCl_3$.

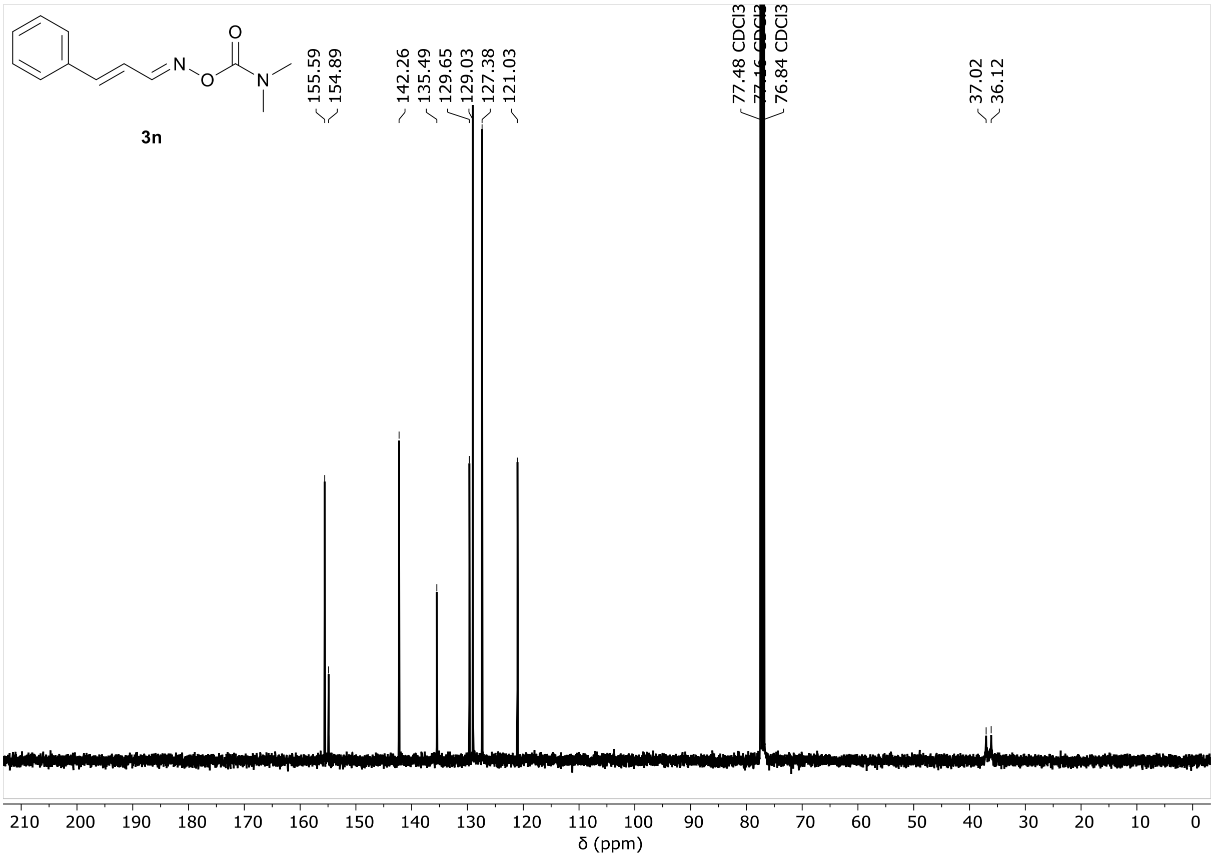

Figure S114. ^{13}C NMR spectrum of cinnamaldehyde *O*-dimethylcarbamoyloxime **3m** in $CDCl_3$.

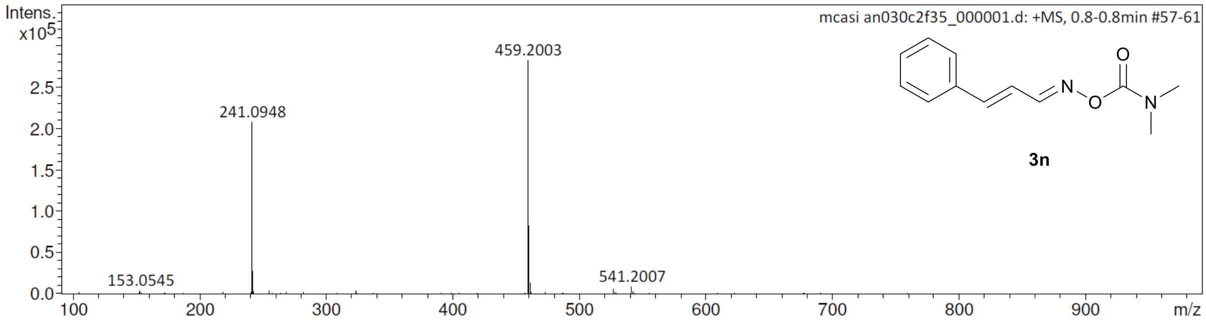

Figure S115. Deconvoluted ESI⁺ HRMS spectrum of cinnamaldehyde *O*-dimethylcarbamoyloxime **3m.**

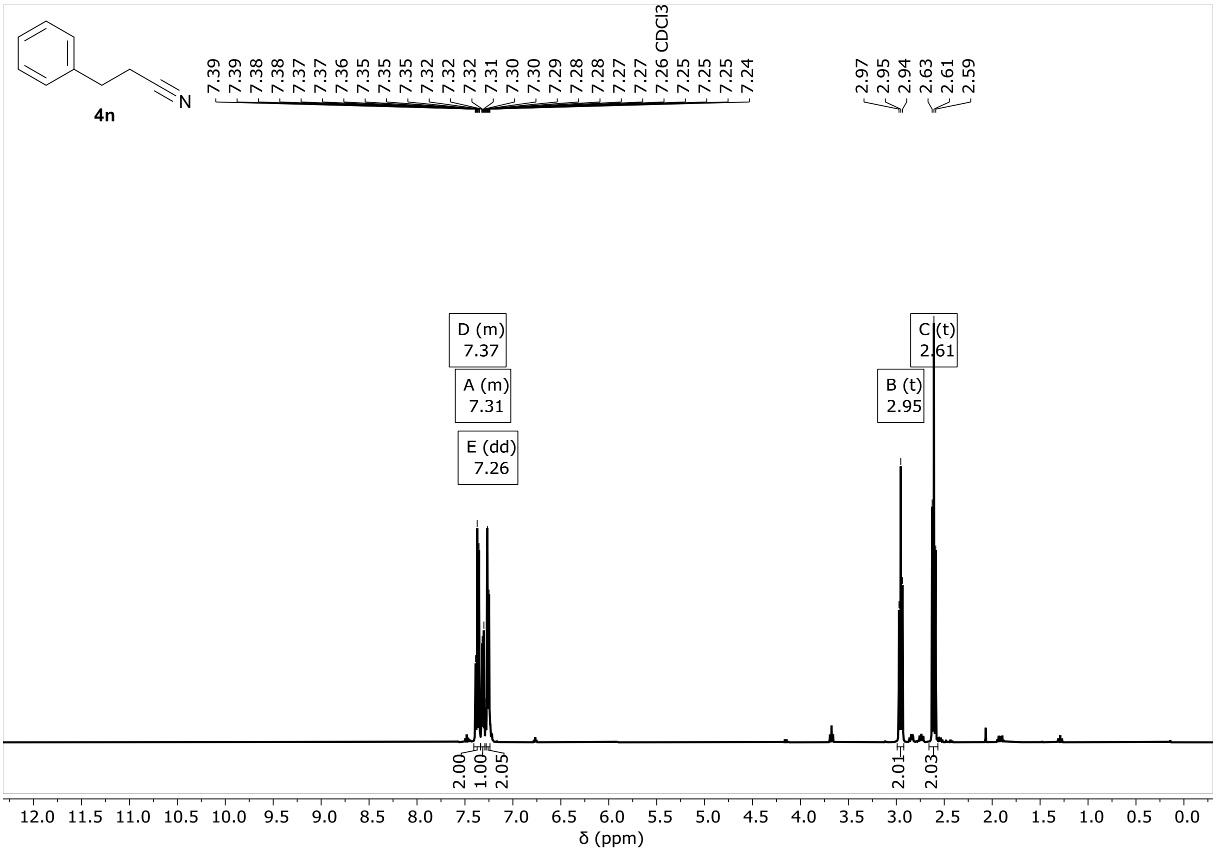

Figure S116. ^{1}H NMR spectrum of 3-phenylpropanenitrile **4n** in $CDCl_3$.

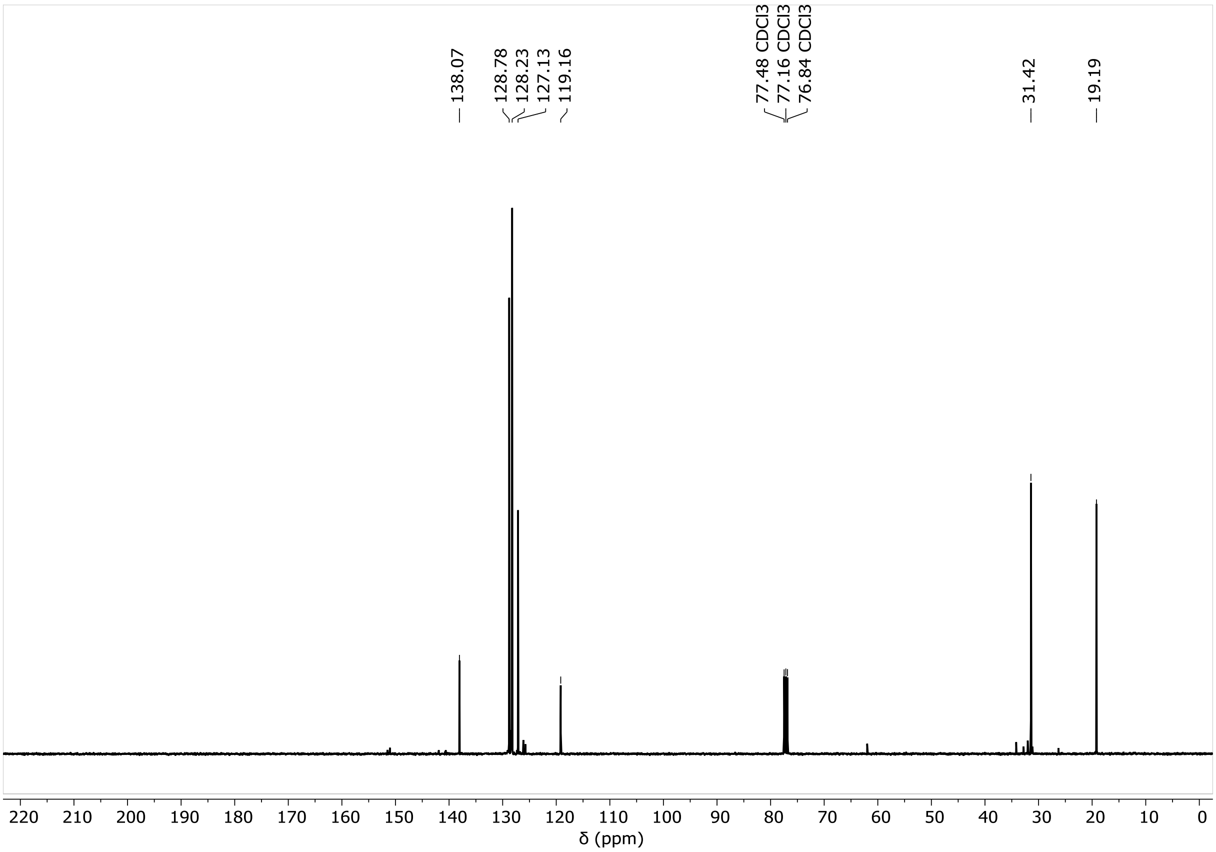

Figure S117. ^{13}C NMR spectrum of 3-phenylpropanenitrile **4n** in $CDCl_3$.

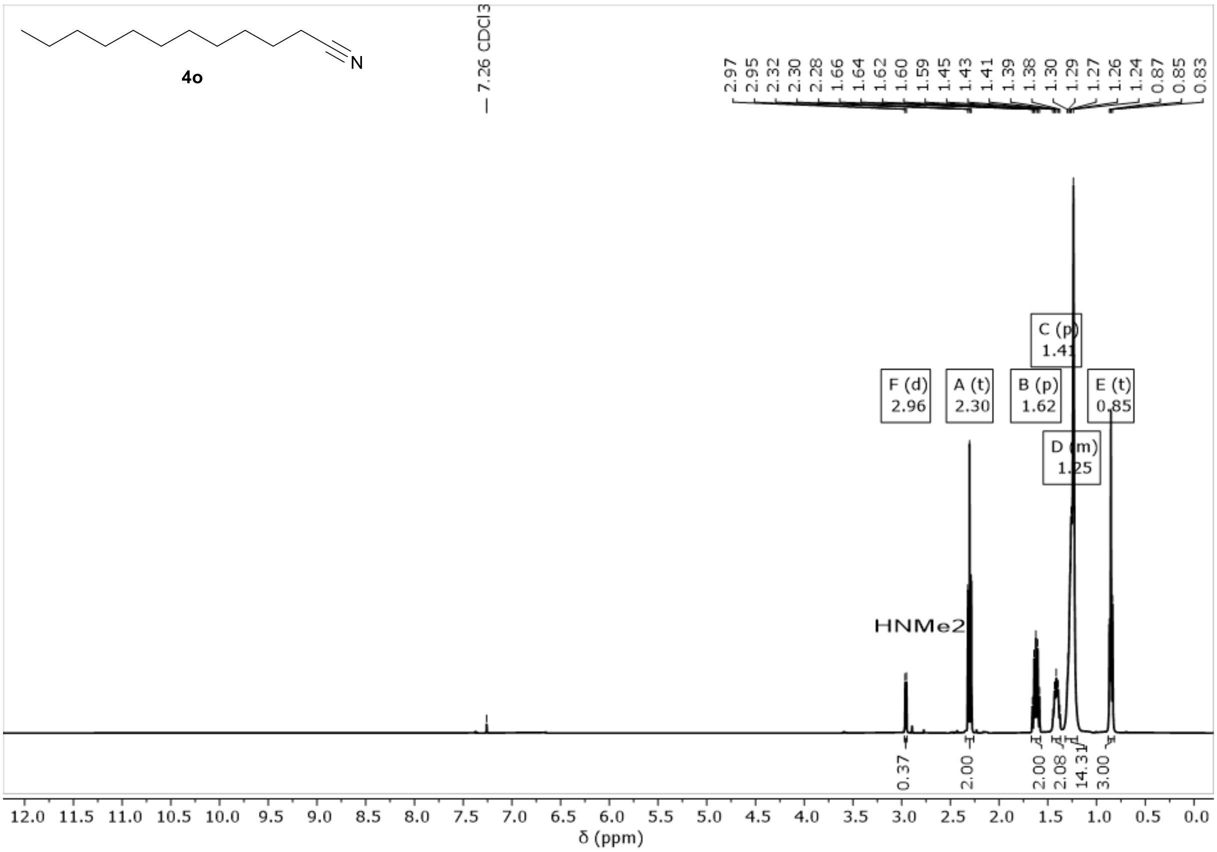

Figure S118. ^{1}H NMR spectrum of dodecanenitrile **4o** in $CDCl_3$.

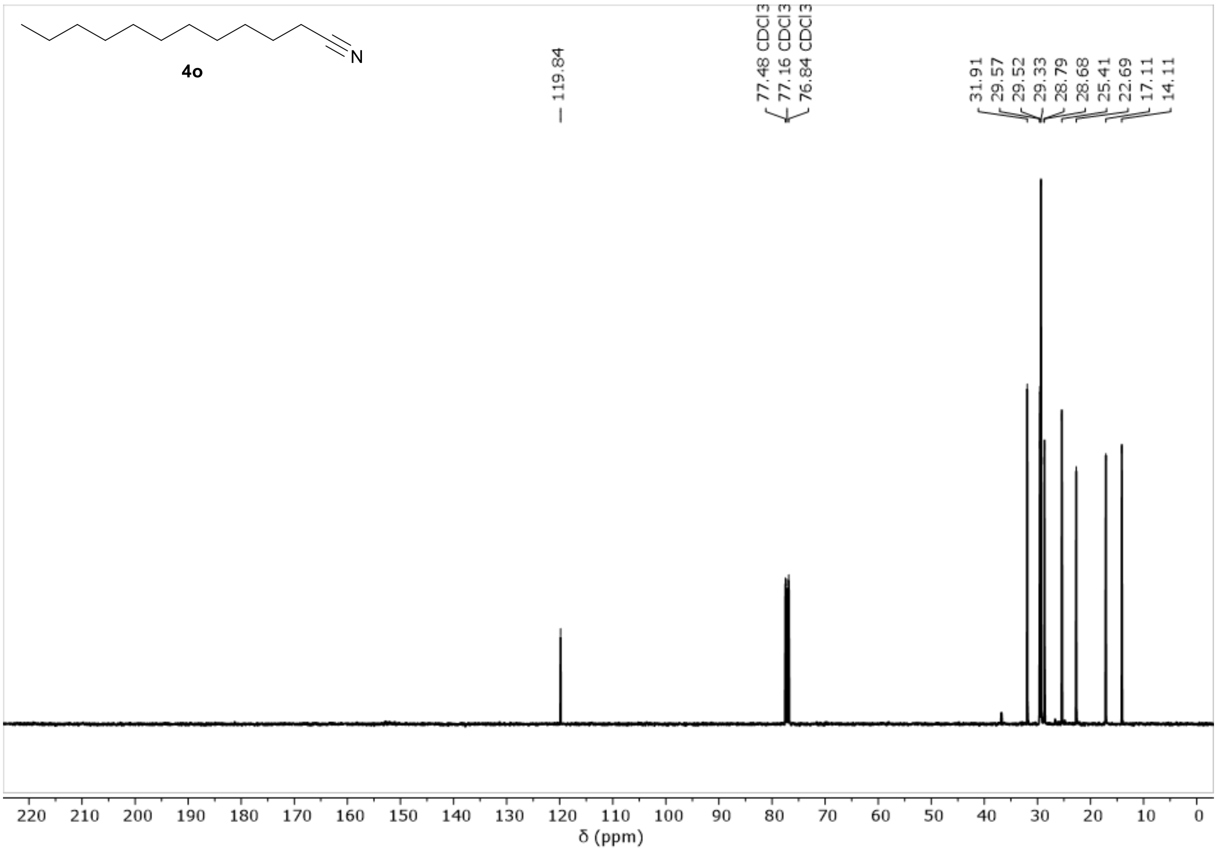

Figure S119. ^{13}C NMR spectrum of dodecanenitrile **4o** in $CDCl_3$.

4. Supporting references

[1] S. Laulhé, S. S. Gori, M. H. Nantz, *J. Org. Chem.* **2012**, *77*, 9334–9337.

[2] X.-Y. Ma, Y. He, T.-T. Lu, M. Lu, *Tetrahedron* **2013**, *69*, 2560–2564.
